# Asymmetric synthesis of metallocenes with planar and central chirality by rhodium-catalyzed desymmetrization reactions†

**DOI:** 10.1039/d5sc00158g

**Published:** 2025-02-26

**Authors:** Nan-Nan Hang, En-Guang Tong, Ting Qi, Chao Sun, Jialin Ming

**Affiliations:** a Natural Products Chem-Bio Innovation Center, College of Food and Biological Engineering, College of Chemistry and Chemical Engineering, Chengdu University Chengdu 610016 China jialin.ming@icloud.com; b Inner Mongolia Key Laboratory of Low Carbon Catalysis, College of Chemistry and Chemical Engineering, Inner Mongolia University 235 West University Street Hohhot 010021 China; c School of Pharmacy, Chengdu University Chengdu 610106 China

## Abstract

Metallocenes with planar and central chirality have emerged as a privileged skeleton for chiral ligand design, and such ligands have exhibited tremendous success in various asymmetric catalysis protocols. Herein, we report a rhodium/chiral diene-catalyzed asymmetric desymmetrization of 1,2-diformylmetallocenes with aryl/alkenylboronic acids to give enantio-enriched formylmetallocenes, which are diastereoisomers of Ugi-type products. This catalytic system also enables the kinetic resolution of 2-substituted 1-formylferrocene with a selectivity factor (*s*) of up to 4331. Compared with traditional synthesis methods, our method has the following advantages: (1) opposite diastereoselectivity; (2) catalytic asymmetric synthesis; (3) single-step construction of planar and central chirality. The synthetic utility of the present method is demonstrated by the asymmetric synthesis of a series of chiral phosphine ligands, including Josiphos- and PPFA-type ligands.

## Abstract 2

Herein, we report a rhodium/chiral diene-catalyzed asymmetric desymmetrization of 1,2-diformylmetallocenes with aryl/alkenylboronic acids to give enantio-enriched formylmetallocenes, which are diastereoisomers of Ugi-type products.

## Graphical abstract

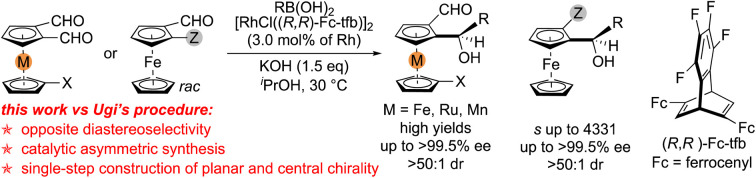

## Introduction

**Scheme 1:**
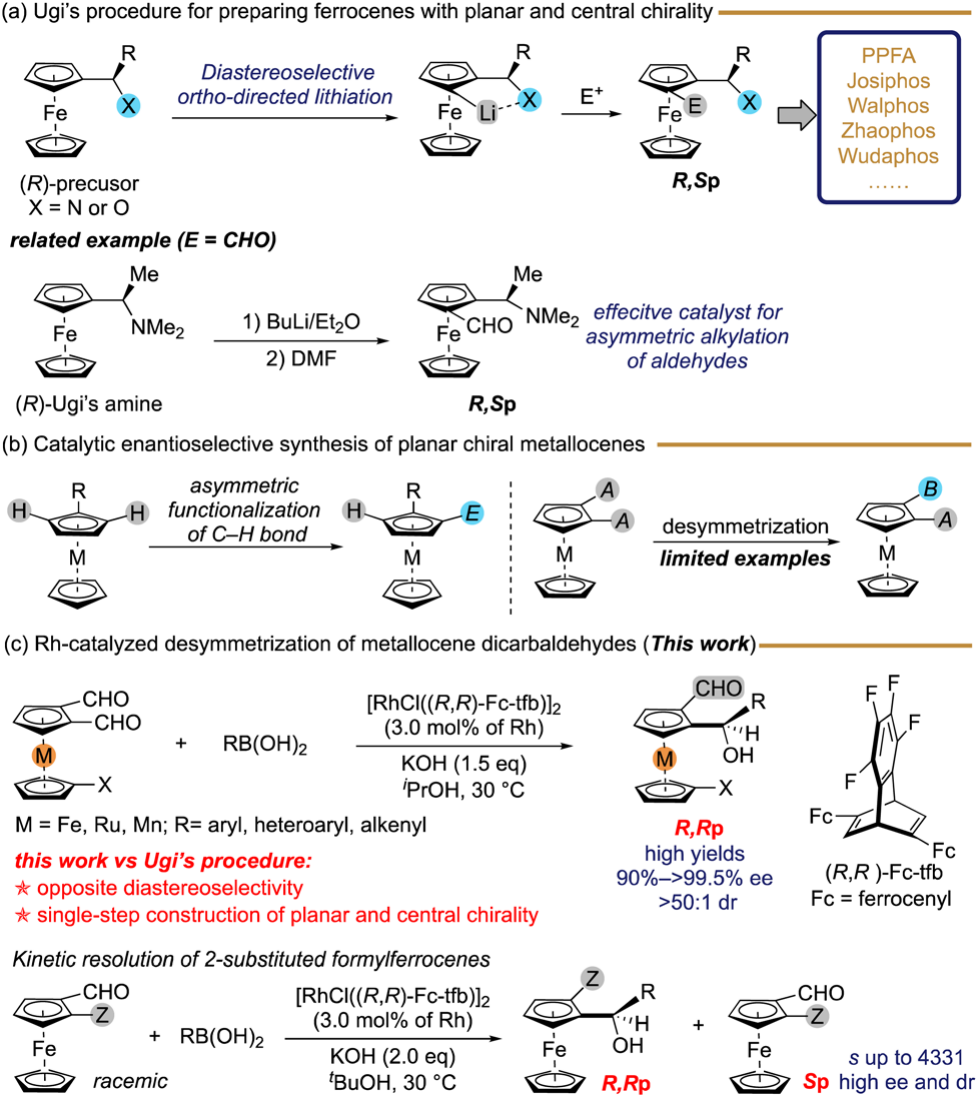
Asymmetric synthesis of metallocenes with planar and central chirality.

Metallocenes possessing planar chirality are of great significance in organic synthesis, materials science, and medicinal chemistry.^1^ Compounds such as PPFA,^2^ TRAP,^3^ Josiphos,^4^ BoPhoz,^5^ Walphos,^6^ Taniaphos,^7^ Zhaophos,^8^ and Wudaphos,^9^ which possess both planar and central chirality, are extensively used in asymmetric catalysis. These ligands are typically prepared according to Ugi's procedure, which introduces additional planar chirality besides their inherent central chirality through diastereoselective *ortho*-lithiation (Scheme 1a).^10^ One example of this is the synthesis of (*R*,*S*p)-ferrocenecarbaldehyde, which has proved successful as a catalyst for the asymmetric alkylation of aldehydes with dialkylzinc reagents, through the *ortho*-lithiation of (*R*)-(2-dimethylaminoethyl)ferrocene (Ugi's amine).^11*b*^ Chiral Ugi's amine is typically prepared by a conventional method that relies heavily on the optical resolution of its racemate with the aid of resolving agents in specific proportions.^10*a*^ As a result, the development of highly effective, simple, and reliable techniques for producing metallocenes with both central and planar chirality is seen as a significant pursuit in the realm of synthetic chemistry. Transition-metal-catalyzed enantioselective C–H functionalization of metallocenes has been the subject of extensive research in relation to catalytic asymmetric synthesis (Scheme 1b).^12^ There have been limited studies on the preparation of enantio-enriched planar chiral metallocenes through catalytic desymmetrization of prochiral compounds.^13^ However, the simultaneous introduction of planar and central chirality in metallocenes in a single step with high diastereo- and enantioselectivity has seldom been reported.^14^

Planar chiral formylferrocenes are an important class of platform molecules, with the potential to be transformed and applied in the synthesis of chiral ligands and catalysts.^15^ Herein, we describe a rhodium-catalyzed desymmetric addition^16^ of organoboronic acids to 1,2-diformylmetallocenes, leading to the formation of chiral metalloceneformaldehydes with both planar and central chirality in high yields with high diastereo- and enantioselectivity (Scheme 1c). Furthermore, we successfully achieved the kinetic resolution of planar chiral 2-substituted formylferrocenes using the same catalytic system. In previous work, the diastereomer of Ugi amine was prepared by multiple-step protection and deprotection, leading to low synthesis efficiency.^17^ Our method offers several advantages over Ugi's procedure, including: (1) catalytic asymmetric synthesis; (2) simultaneous generation of planar and central chirality; (3) opposite planar diastereoselectivity; (4) simple and mild reaction conditions. We have demonstrated the practical application of our approach by synthesizing various chiral phosphine ligands, such as Josiphos- and PPFA-type ligands, in an asymmetric manner.

## Results and discussion

As a model reaction, the addition of PhB(OH)_2_ (2a, 1.0 equiv. with respect to 1a) to 1,2-diformylferrocene (1a) was performed in the presence of 3.0 mol% of rhodium catalysts bearing several types of chiral diene^16*e*^ and bisphosphine ligands and 1.5 equiv. of KOH in 2-propanol at 30 °C for 10 h (Table 1). The reaction with (*R*,*R*)-Fc-tfb^18^ as a ligand gave a 93% yield of 3aa with >99.5% ee, and its diastereoisomer 4aa was not detected by ^1^H NMR spectroscopy (entry 1). Another commonly used chiral diene ligand, (*R*,*R*)-Ph-bod, gave a moderate yield of a mixture of 3aa and 4aa with much lower enantioselectivity (entry 2). Other ligands, namely (*R*)-Segphos, (*R*)-Binap, and (*R*)-Ph-Phox, gave low yields of 3aa with low stereoselectivity, and the formation of side product 5, which was produced by hydrogenation of 1a, was detected in these reactions (entries 3–5). Reactions in other protic solvents, namely dioxane/H_2_O and MeOH, gave 3aa with moderate enantioselectivity (entries 6 and 7). The reaction in ^*t*^BuOH also gave 3aa in high yield with high ee and dr (entry 8). The use of PhZnCl in place of PhB(OH)_2_ gave a trace amount of 3aa (entry 9).

**Table 1:** Optimization of catalytic conditions^a^

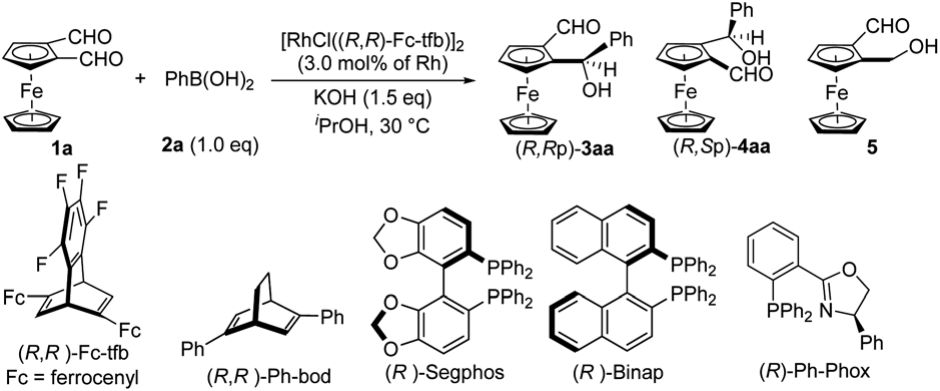				
Entry	Variations from standard conditions (shown above)	Yield^b^ (%) 3aa	dr^c^ (3 : 4)	% ee^d^3aa
1	None	93	>50 : 1	>99.5
2	[RhCl((*R*,*R*)-Ph-bod)]_2_	44	67 : 33	51
3	[RhCl(coe)_2_]_2_ + (*R*)-Segphos	<5	—	—
4	[RhCl(coe)_2_]_2_ + (*R*)-Binap	11	63 : 37	24
5	[RhCl(coe)_2_]_2_ + (*R*)-Ph-Phox	10	80 : 20	0
6	Dioxane/H_2_O (10/1) instead of ^*i*^PrOH	90	>50 : 1	79
7	MeOH instead of ^*i*^PrOH	20	>50 : 1	79
8	^*t*^BuOH instead of ^*i*^PrOH	94	>50 : 1	96
9^e^	PhZnCl instead of PhB(OH)_2_	<5	—	—

a Reaction conditions: 1a (0.10 mmol), 2a (0.10 mmol), KOH (0.15 mmol), [RhCl((*R*,*R*)-Fc-tfb)]_2_ (3.0 mol% Rh), and 2-propanol (1.0 mL) at 30 °C for 10 h.

b Isolated yield.

c dr (diastereomeric ratio) was determined by ^1^H NMR spectroscopy of the crude reaction mixture. Considering the accuracy of ^1^H NMR spectroscopy, the dr is >50 : 1.

d % ee was determined by HPLC on a chiral stationary phase column.

e Reaction conditions: 1a (0.10 mmol), PhZnCl (0.20 mmol), [RhCl((*R*,*R*)-Fc-tfb)]_2_ (3.0 mol% Rh), and THF (1.0 mL) at 30 °C for 10 h.

**Scheme 2:**
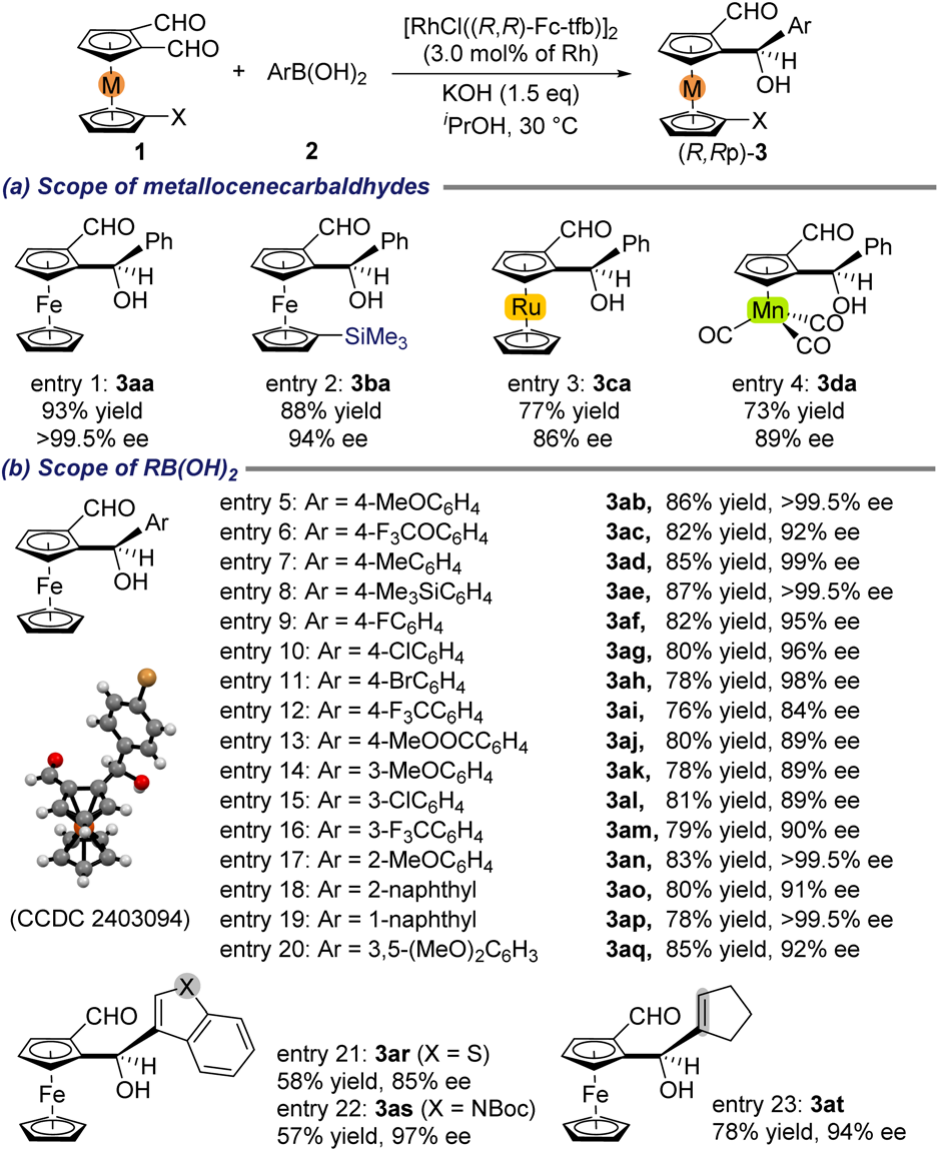
Rh-catalyzed desymmetric addition of organoboronic acids to metallocene dicarbaldehydes: substrate scope. Reaction conditions: 1 (0.10 mmol), 2 (0.10 mmol), KOH (0.15 mmol), [RhCl((*R*,*R*)-Fc-tfb)]_2_ (3.0 mol% Rh), and 2-propanol (1.0 mL) at 30 °C for 10 h. Isolated yield. dr (diastereomeric ratio) was determined by ^1^H NMR spectroscopy of the crude reaction mixture. Considering the accuracy of ^1^H NMR spectroscopy, the dr is >50 : 1. % ee was determined by HPLC on a chiral stationary phase column. The absolute configuration of 3ah was determined to be *R*,*R*_P_ by X-ray crystallographic analysis (CCDC 2403094).

Having established the optimal reaction conditions (Table 1, entry 1), the substrate scope of this desymmetrization reaction of various 1,2-diformylmetallocenes with PhB(OH)_2_ (2a) was examined, and the results are summarized in Scheme 2a. Desymmetrization of 1b, with a Me_3_Si- group at the 1′-position of the ferrocene cyclopentadiene moiety, proceeded well to give the corresponding product 3ba with high diastereo- and enantioselectivity (entry 2). Using the present method, chiral ruthenocene derivative 3ca and tricarbonylcyclopentadienyl manganese derivative 3da were successfully obtained in high yields with excellent dr and high ee values under the standard conditions (entries 3 and 4).

Scheme 2b provides an overview of the results obtained for reactions of 1,2-diformylferrocene (1a) with diversely functionalized organoboronic acids 2, including aryl-, heteroaryl-, and alkenylboronic acids, under the standard conditions. The desymmetric addition proceeded smoothly for ArB(OH)_2_2b–2j, which are aromatic groups consisting of aryl moieties with methoxy, trifluoromethoxy, methyl, trimethylsilyl, halo, trifluoromethyl, and ester substituents at the *para* position (entries 5–13). The corresponding products 3ab–3aj were obtained in high yields with high diastereo- and enantioselectivity (>50 : 1 dr, 84 ≥ 99.5% ee). However, the presence of electron-withdrawing groups generally decreased the enantioselectivity. Arylboronic acids 2k–2m, with electron-donating and -withdrawing substituents at their *meta* position, gave high yields of the corresponding products 3ak–3am with enantioselectivity of 89–90% ee (entries 14–16). The reaction of *ortho*-substituted arylboronic acid 2n also took place, giving the corresponding product 3an in high yield with excellent diastereo- and enantioselectivity (entry 17). The desymmetric addition of 1a also proceeded smoothly for polysubstituted arylboronic acids 2o–2q to give the corresponding products 3ao–3aq in high yields with both high diastereo- and enantioselectivity (entries 18–20). The desymmetric addition of heteroarylboronic acids 2r and 2s gave moderate yields of the corresponding products 3ar and 3as with both high dr and ee (entries 21 and 22). Desymmetric alkenylation of 1a with 1-cyclopentenylboronic acid (2t) also gave a high yield of the corresponding product 3at with high dr and ee (>50 : 1 dr, 94% ee, entry 23).

**Scheme 3:**
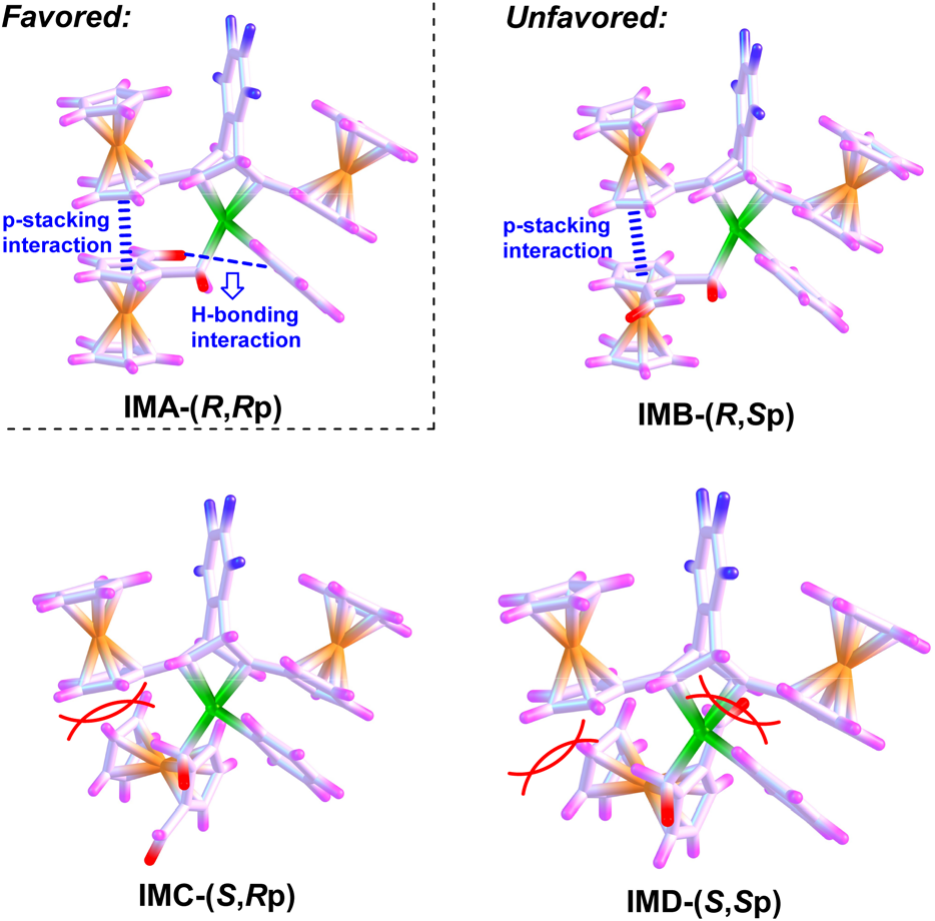
Proposed stereochemical pathway.

The (*R*,*R*p)-configuration for compound 3ah can be rationalized by the stereochemical pathway shown in Scheme 3. The steric match among the ferrocenyl and aldehyde moieties of 1a, Rh-Ph fragment, and the chiral (*R*,*R*)-Fc-tfb ligand might be very important for achieving the high level of stereo-control. For chiral recognition of the two enantiotopic aldehyde groups of 1a, the coordination of 1a with Rh/(*R*,*R*)-Fc-tfb is more favorable in the configuration IMA than in IMB, leading to the formation of planar chirality in the (*R*p)-configuration. This can originate from the favorable non-covalent interactions, *i.e.* π-stacking interaction between the ferrocenyl moiety of 1a and the ferrocenyl group on the olefin of (*R*,*R*)-Fc-tfb and hydrogen-bonding interaction between the aldehyde fragment of 1a and the Rh-Ph moiety. To avoid the unfavorable steric hindrance between the carbonyl and ferrocenyl moieties of 1a and the ferrocenyl group on the olefin of (*R*,*R*)-Fc-tfb, IMA is preferred to IMC or IMD, offering the (*R*)-configuration product. Thus, the Rh/(*R*,*R*)-Fc-tfb catalyst effectively constructs the stereochemical model of (*R*,*R*p)-configuration.

**Scheme 4:**
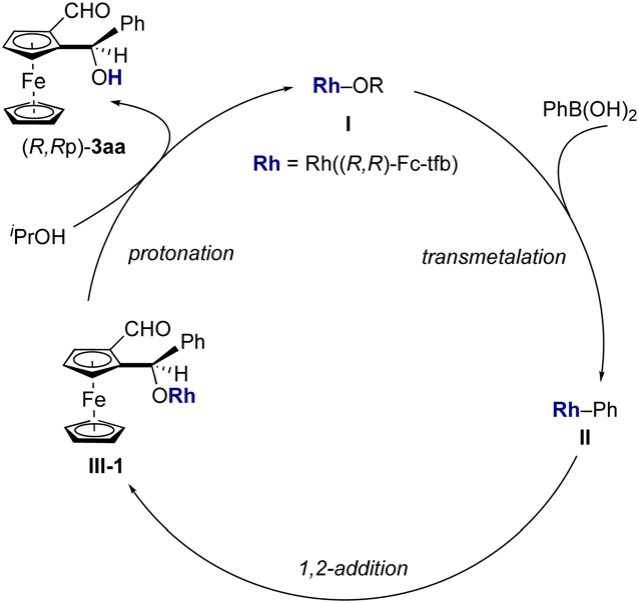
Proposed catalytic cycle.

The catalytic cycle for the rhodium/chiral diene-catalyzed asymmetric desymmetrization of 1,2-diformylmetallocenes is proposed in Scheme 4. 1,2-Addition of 1,2-diformylferrocene (1a) with phenyl-rhodium species II, which is generated by transmetalation of PhB(OH)_2_ with [Rh]–OR species I, affords a rhodium alkoxide species III. Further protonation of intermediate III gives product 3aa and regenerates [Rh]–OR species I.

**Scheme 5:**
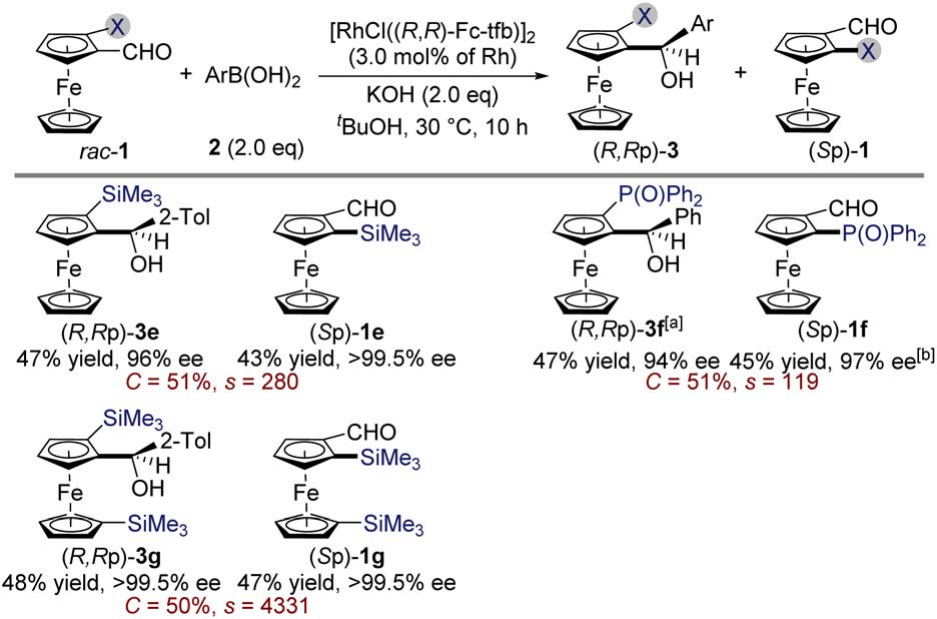
Kinetic resolution of planar chiral 2-substituted formylferrocenes. Reaction conditions: 1 (0.10 mmol), 2 (0.20 mmol), KOH (0.20 mmol), [RhCl((*R*,*R*)-Fc-tfb)]_2_ (3.0 mol% Rh), and ^*t*^BuOH (1.0 mL) at 30 °C for 10 h. Calculated conversion, *C* = ee_1_/(ee_1_ + ee_3_). The % ee was determined by HPLC on a chiral stationary phase column. Selectivity factor, *s* = ln[(1 − *C*)(1 − ee_1_)]/ln[(1 − *C*)(1 + ee_1_)]. Diastereomeric ratio (dr) = >50 : 1 (determined by ^1^H NMR) spectroscopy. ^*a*^At 80 °C. ^*b*^Compound 1f was transformed to compound 12a (Scheme 6) for HPLC analysis.

Next, the Rh/(*R*,*R*)-Fc-tfb catalytic system was successfully applied to the kinetic resolution (KR) of racemic 2-substituted formylferrocenes (Scheme 5). The KR of 2-silyl formylferrocene 1e with 2-methylphenylboronic acid in the presence of the Rh/(*R*,*R*)-Fc-tfb catalyst in *tert*-butanol proceeded smoothly with 51% conversion, giving the arylation product 3e (>50 : 1 dr) with 96% ee and recovered 1e with >99.5% ee, and the corresponding selectivity factor (*s*) for *rac*-1e was 280. This KR methodology also proved to be highly efficient towards 1f bearing a phosphine oxide group, giving a high *s*-factor value, with a high yield of the corresponding product 3f, and both 3f and recovered 1f having high ee. The KR of 1g, with a Me_3_Si- group at the 1′-position of the ferrocene moiety, proceeded well and gave the corresponding product 3g with an excellent *s*-factor (*s* = 4331).

**Scheme 6:**
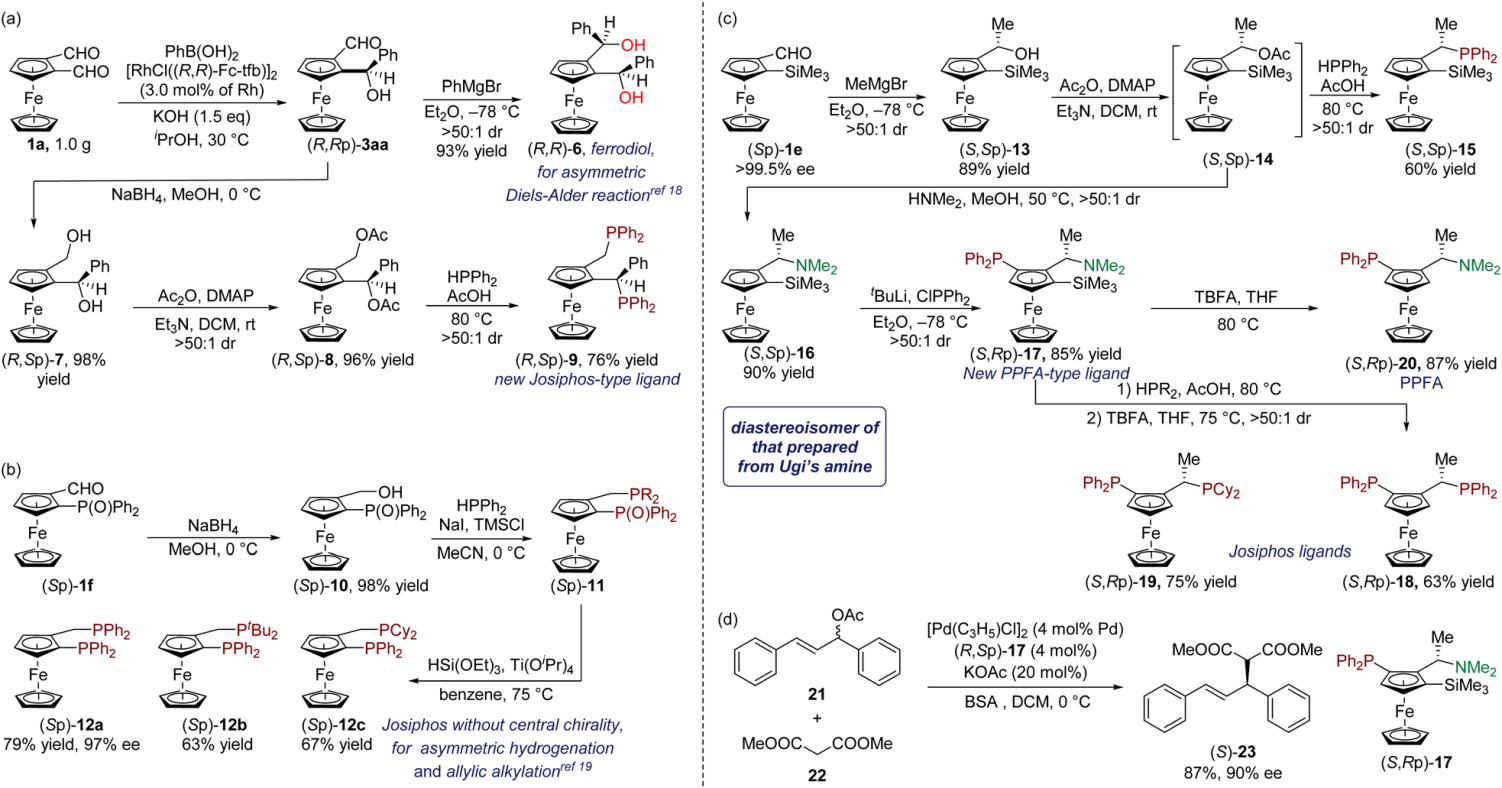
Synthetic transformations. (a) Scale experiment. (b) Josiphos-type ligands without central chirality. (c) PPFA- and Josiphos-type ligands. (d) Asymmetric allylic alkylation.

To demonstrate the synthetic potential of our method, the products were expeditiously transformed into potentially useful phosphine ligands (Scheme 6). A ferrocenyl-based chiral diol ligand (FERRODIOL), prepared from optically pure aminoformylferrocene, has been developed as a chiral ligand for scandium-catalyzed asymmetric Diels–Alder reactions.^19^ As shown in Scheme 6a, FERRODIOL 6 was easily prepared by the diastereoselective addition of a Grignard reagent to compound 3aa, which was obtained as indicated in Table 1, entry 1. Moreover, a new Josiphos-type ligand 9 was prepared through a sequence that comprised reduction, acylation, and diastereoselective S_N_1-type reaction with HPPh_2_ as a nucleophilic reagent. There are very few examples of bisphosphanes that possess only planar chirality. Here, we successfully prepared several enantiopure Josiphos-type ligands 12a–12c with planar chirality as the sole source of chirality. Their rhodium complexes proved to be efficient catalysts for the asymmetric hydrogenation of various alkenes (Scheme 6b).^20^ Thus, starting from 1f, ligands 12a–12c were efficiently synthesized through a sequence that involved reduction with NaBH_4_, S_N_1-type reaction, and reduction with HSi(OEt)_3_. Subsequently, addition of Grignard reagent MeMgBr to 1e yielded alcohol 13, which was then converted into acetate 14 by treating it with acetic anhydride (Scheme 6c). The crude compound 14 served as the starting material for the synthesis of a ferrocene-derived chiral monophosphine ligand 15 by a simple S_N_1-type reaction with HPPh_2_.^21^ Moreover, compound 14 was also transformed into 16, the diastereoselectivity of which is opposite to that of the product obtained from Ugi's amine through nucleophilic substitution with HNMe_2_. Furthermore, the chiral PPFA ligand derivative 17 was obtained in 85% isolated yield by diphenylphosphination of the lithiated ferrocene, produced through the reaction of 16 with butyllithium. The efficiency of ligand 17 was demonstrated by a Pd-catalyzed asymmetric allylic alkylation reaction (87% yield, 90% ee) (Scheme 6d).^22^ Similarly, the new PPFA ligand also underwent S_N_1 reactions with HPPh_2_ and HPCy_2_, and subsequent desilication afforded Josiphos ligands 18 and 19, respectively, in high yields. Desilication of compound 17 also gave the normal PPFA ligand 20 in high yield. In summary, various chiral ligands, including FERRODIOL 6, new Josiphos-type ligand 9, Josiphos-type ligands 12a–12c without central chirality, a ferrocene-derived chiral monophosphine ligand 15, PPFA-type ligand 17, known Josiphos ligands 18 and 19, and known PPFA ligand 20, were successfully produced.

## Conclusions

In summary, we have developed a highly efficient synthesis of enantiopure planar and central chiral metallocenes based on rhodium-catalyzed asymmetric addition under mild conditions. These chiral metallocenes, created through the simultaneous construction of planar and central chirality from achiral 1,2-diformylmetallocene, were obtained in high yields with excellent diastereo- and enantioselectivity. Furthermore, the KR reaction proceeded smoothly, achieving a selectivity factor of up to 4331. The synthetic utility of the present method has been demonstrated by the asymmetric synthesis of a series of chiral phosphine ligands, including Josiphos- and PPFA-type ligands.

## Data availability

Experimental procedures, compound characterization data and X-ray crystallographic data of compound 3ah. CCDC 2403094. For ESI and crystallographic data in CIF or other electronic format see DOI: https://doi.org/10.1039/d5sc00158g.

## Author contributions

N.-N. Hang and E.-G. Tong contributed equally. The manuscript was written through contributions of all authors.

## Conflicts of interest

There are no conflicts to declare.

## Supplementary Material

SC-016-D5SC00158G-s001

SC-016-D5SC00158G-s002
