# Supplementary material for: Asymmetric synthesis of metallocenes with planar and central chirality by rhodium-catalyzed desymmetrization reactions
Source: Chem Sci. 2025 Feb 26;16(14):5880–6. doi: 10.1039/d5sc00158g (PMC11880838; doi:10.1039/d5sc00158g)
Supplement: SC-016-D5SC00158G-s001 [file SC-016-D5SC00158G-s001.pdf]

Supplementary Information (SI) for Chemical Science.
This journal is © The Royal Society of Chemistry 2025

Supporting Information

Asymmetric synthesis of metallocenes with planar and central chirality by rhodium-catalyzed desymmetrization reactions

Nan-Nan Hang,[a,b] En-Guang Tong,[a,b] Ting Qi,[c] Chao Sun,[a] and Jialin Ming*[a]

[a]Natural Products Chem-Bio Innovation Center, College of Food and Biological Enginering, College of Chemistry and Chemical Engineering, Chengdu University, Chengdu 610016, China

[b]Inner Mongolia Key Laboratory of Fine Organic Synthesis, Inner Mongolia University, 235 West University Street, Hohhot 010021

[c]School of Pharmacy, Chengdu University, Chengdu 610106, China

*Email: mingjialin@imu.edu.cn

Contents

1. **General method** 3

2. **Materials** 4

3. **Preparation of 1,2-diformylmetallocenes** 5

4. **A typical procedure for Rh-catalyzed desymmetric addition of organoboronic acids to 1,2-diformylmetallocenes (Table 1, entry 1)** 7

5. **A typical procedure for kinetic resolution of 2-substituted 1-formylferrocenes (Scheme 4)** 8

6. **Optimization of the reaction conditions** 9

7. **Synthetic transformations** 11

   7.1 **Synthesis of Josiphos-type ligands** 11

   7.2 **Synthesis of Josiphos without central chirality** 12

   7.3 **Synthesis of Josiphos, PPFA, and other phosphorous ligands** 13

   7.4 **Palladium-catalyzed asymmetric allylic alkylation** 16

8. **Characterization of the products** 18

9. **Single crystal x-ray diffraction date for compound 3ah(CCDC 2403094)** 38

10. **References** 40

11. **Chiral HPLC charts and NMR spectra** 41

1. General method

All air-sensitive manipulations were carried out with standard Schlenk techniques under nitrogen or argon. NMR spectra were recorded on Bruker spectrometer AV 600MHz, QNP probe (600 MHz for ^{1}H, 150 MHz for ^{13}C, and 243 MHz for ^{31}P). Chemical shifts are reported in δ (ppm) referenced to the residual solvent peak of $CHCl_3$ (δ 7.26) for ^{1}H NMR spectroscopy and $CDCl_3$ (δ 77.0) for ^{13}C NMR spectroscopy. The following abbreviations are used to describe the multiplicities; s: singlet, d: doublet, t: triplet, q: quartet, quint: quintet, m: multiplet, br: broad. Coupling constants are reported in Hertz (Hz). Optical rotations were measured on an Anton Paar MCP-4100. High resolution mass spectra (HRMS) (ESI) were obtained on a Bruker microTOF II spectrometer. Matrix-Assisted Laser Desorption/Ionization Time of Flight (MALDI-TOF) mass spectra were measured on an AXIMA Performance spectrometer. For thin layer chromatography (NUO TAI precoated TLC plates (SHF254) were used, and compounds were visualized with a UV light at 254 nm TLC). Further visualization was achieved by staining with $KMnO_4$ followed by heating. Flash column chromatography was performed with Silica gel (SANPONT). Enantiomeric excesses (ee) were determined by HPLC analysis on Shimadzu (LC-40) HPLC and Shimazu (LC-16) HPLC with Daicel chiral columns.

2. Materials

N,*N*-dimethylaminomethylferrocene, *N*,*N*-dimethylaminomethylruthenocene, 1-dimethylaminomethyl-1'-trimethylsilylferrocene, tetrabutylammonium fluoride, tricarbonyl[(1,2,3,4,5-*η*)-1-[(dimethylamino)methyl]-2,4-cyclopentadien-1-yl]manganese, chlorotrimethylsilane, chlorodiphenylphosphine, hydrogen peroxide (30 wt.% in H_2O), methylmagnesium bromide, phenylmagnesium bromide, sodium borohydride, diphenylphosphine, sodium iodide, triethoxysilane, titanium tetraisopropanolate, 4-dimethylaminopyridine, dicyclohexylphosphine, dimethylamine, tetrabutylammonium fluoride, organoboronic acids **2a**–**2t** were purchased and used as received. Tetrahydrofuran, ethyl ether, methylene chloride, isopropanol, *tert*-butanol and toluene were distilled over benzophenone ketyl under Ar.

$[RhCl(coe)_2]_2$,[1] [RhCl((*R,R*)-Fc-tfb)]$_2$,[2] **1e** (CAS No. 146291-53-4),[3] **1f** (CAS No. 854497-34-0),[4] **1g** (CAS No. 934276-76-3),[5] and **21**[6] were prepared according to the reported procedures.

3. Preparation of 1,2-diformylmetallocenes

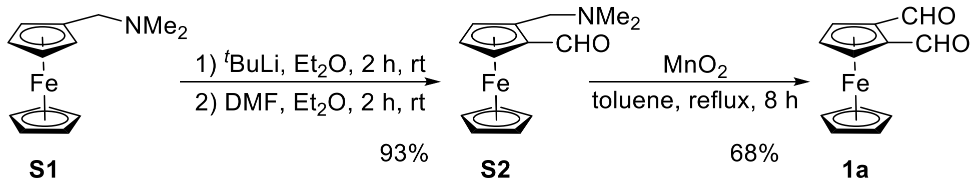

Under argon, to a solution of *N*,*N*-dimethylaminomethylferrocene (**S1**, 452.2 mg, 1.86 mmol, 1.0 equiv) in Et_2O (25 mL), ^{t}BuLi (1.3 M in heptane, 2.2 mL, 2.79 mmol, 1.5 equiv) was added dropwise at 0 °C, and the mixture was stirred at room temperature for 2 h. DMF (271.9 mg, 3.72 mmol, 2.0 equiv) was added to the mixture. The mixture was stirred at room temperature for 2 h, quenched with H_2O (5.0 mL), and extracted with ethyl acetate. The combined organic layers were dried over $MgSO_4$, filtered, and concentrated under vacuum. The residue was purified by flash column chromatography on silica gel (hexane/ethyl ether/triethylamine = 10:1:1) to give compound **S2** (93% yield, 469.1 mg, 1.73 mmol) as a red oil. **^{1}H NMR** (600 MHz, $CDCl_3$): δ 10.10 (s, 1H), 4.78 (s, 1H), 4.61 (s, 1H), 4.55 (s, 1H), 4.22 (s, 5H), 3.83 (d, *J* = 13.0 Hz, 1H), 3.34 (d, *J* = 13.0 Hz, 1H), 2.21 (s, 6H). The experimental data are in agreement with the literature report.[7]

Under argon, a solution of compound **S2** (317.2 mg, 1.17 mmol, 1.0 equiv) in toluene (30 mL) was added to activated MnO_2 (2.101 g, 23.4 mmol, 20.0 equiv). The mixture was stirred at reflux for 8 h and then filtered over Celite 545. The organic layers were concentrated under vacuum. The residue was purified by flash column chromatography on silica gel (hexane/ethyl ether = 5:1) to give compound **1a** (68% yield, 193.6 mg, 0.80 mmol) as a red solid. **^{1}H NMR** (600 MHz, $CDCl_3$): δ 10.4 (s, 2H), 5.21 (s, 2H), 4.94 (s, 1H), 4.40 (s, 5H). The experimental data are in agreement with the literature report.[7]

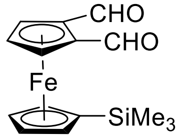

1b

Compound 1b: (66% yield, 103.7 mg, 0.33 mmol, a red solid, R_f = 0.3. (eluent: hexane/ethyl acetate = 5:1)). It was synthesized from compound 1-dimethylaminomethyl-1'-trimethylsilylferrocene according to the procedure for compound **1a** in 0.50 mmol scale. **^{1}H NMR** (600 MHz, $CDCl_3$): δ 10.4 (s, 2H), 5.16 (d, *J* = 2.8 Hz, 2H), 4.88 (s, 1H), 4.60

(s, 2H), 4.29 (s, 2H), 0.22 (s, 9H). The experimental data are in agreement with the literature report.[7]

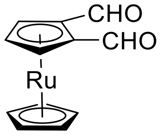

1c

Compound 1c: (58% yield, 83.3 mg, 0.29 mmol, a yellow solid, R_f = 0.3. (eluent: hexane/ethyl acetate = 5:1)). It was synthesized from compound *N*,*N*-dimethylaminomethylruthenocene according to the procedure for compound **1a** in 0.50 mmol scale. **^{1}H NMR** (600 MHz, $CDCl_3$): δ 10.1 (s, 2H), 5.39 (d, *J* = 2.7 Hz, 2H), 5.10 (t, *J* = 2.7 Hz, 1H), 4.77 (s, 5H). The experimental data are in agreement with the literature report.[7]

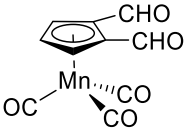

1d

Compound 1d: (48% yield, 62.4 mg, 0.24 mmol, a dark green solid, R_f = 0.4. (eluent: hexane/ethyl acetate = 5:1)). It was synthesized from compound tricarbonyl[(1,2,3,4,5-*η*)-1-[(dimethylamino)methyl]-2,4-cyclopentadien-1-yl]-manganese according to the procedure for compound **1a** in 0.50 mmol scale. **^{1}H NMR** (600 MHz, $CDCl_3$): δ 10.0 (s, 2H), 5.66 (s, 2H), 4.95 (s, 1H). The experimental data are in agreement with the literature report.[7]

4. A typical procedure for Rh-catalyzed desymmetric addition of organoboronic acids to 1,2-diformylmetallocenes (Table 1, entry 1)

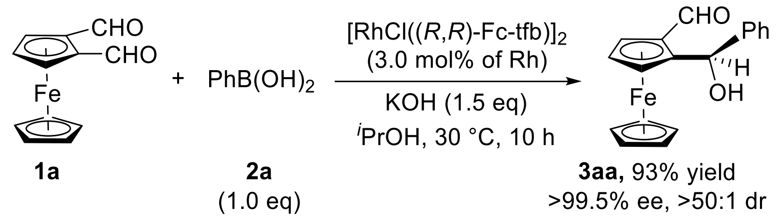

An oven-dried Schlenk tube was charged with **1a** (24.2 mg, 0.10 mmol, 1.0 equiv), [RhCl((*R,R*)-Fc-tfb)]$_2$ (2.19 mg, 1.5 μmol, 3.0 mol% of Rh), PhB(OH)$_2$ (12.2 mg, 0.10 mmol, 1.0 equiv), KOH (8.40 mg, 0.15 mmol, 1.5 equiv), and isopropanol (1.0 mL) under argon. The tube was placed in a preheated oil bath at 30 °C. The mixture was stirred at 30 °C for 10 h before extracted with ethyl acetate. The organic layers were concentrated under vacuum. The residue was subjected to column chromatography on silica gel to give compound (*R*,*R*p)-**3aa** (93% yield, 29.8 mg, 0.093 mmol) as a red solid, R_f = 0.4 (hexane/ethyl acetate (5/1)).

5. A typical procedure for kinetic resolution of 2-substituted 1-formylferrocenes (Scheme 4)

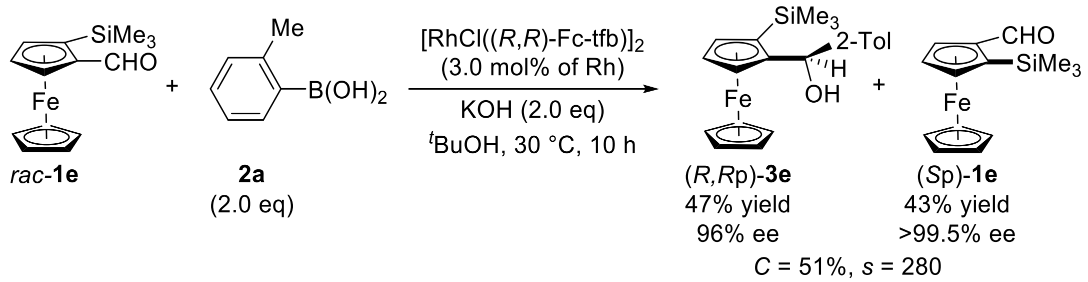

An oven-dried Schlenk tube was charged with compound *rac*-**1e** (28.6 mg, 0.10 mmol, 1.0 equiv), [RhCl((*R*,*R*)-Fc-tfb)]$_2$ (2.19 mg, 1.5 μmol, 3.0 mol% Rh), 2-methyphenylboronic acid (27.2 mg, 0.20 mmol, 2.0 equiv), KOH (11.2 mg, 0.20 mmol, 2.0 equiv), and *tert*-butanol (1.0 mL) under argon. The tube was placed in a preheated oil bath at 30 °C. The mixture was stirred at 30 °C for 10 h before extracted with ethyl acetate. The organic layers were concentrated under vacuum. The residue was subjected to column chromatography on silica gel to give compound (*R*,*R*p)-**3e** (47% yield, 17.8 mg, 0.047 mmol) as a yellow solid, R_f = 0.60 (hexane/ethyl acetate (20/1)), and the recovered compound (*S*p)-**1e** (43% yield, 12.3 mg, 0.043 mmol) as a red solid, R_f = 0.40 (hexane/ethyl acetate (20/1)). (Calculated conversion, *C*. = $ee_1/(ee_1+ee_3)$, selectivity factor, $s = \ln[(1–C)(1–ee_1)]/\ln[(1–C)(1+ee_1)]$).

6. Optimization of the reaction conditions

Table S1 Rh-catalyzed addition to metallocene dicarbaldehydes **1a**: evaluation of ligands[a].

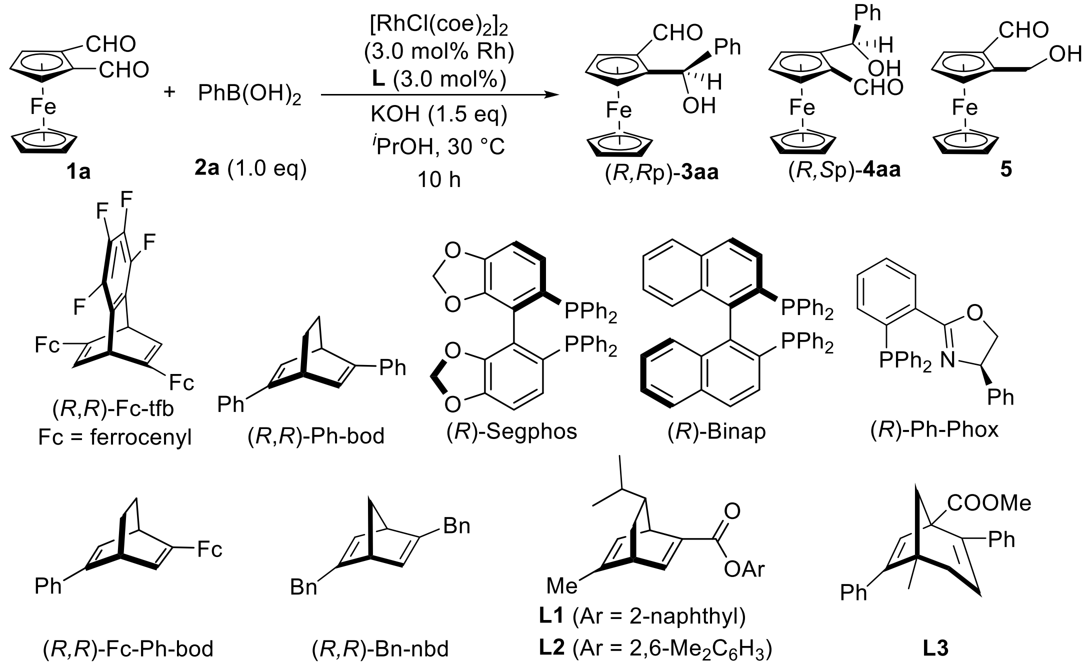

entry	Rh catalyst	yield (%)[b] **3aa** + **4aa**	ratio[c] of **3aa**/**4aa**	ee[d] of **3aa**
1	$[RhCl(coe)_2]_2$ +(*R*,*R*)-Fc-tfb	93	>50:1	>99.5
2	$[RhCl(coe)_2]_2$ + (*R*,*R*)-Ph-bod	44	67:33	51
3	$[RhCl(coe)_2]_2$ + (*R*)-Segphos	<5	--	--
4	$[RhCl(coe)_2]_2$ +(*R*)-Binap	11	63:37	24
5	$[RhCl(coe)_2]_2$ +(*R*)-Ph-Phox	10	80:20	0
6	$[RhCl(coe)_2]_2$ + (*R*,*R*)-Fc-Ph-bod	<5	--	--
7	$[RhCl(coe)_2]_2$ + Bn-nbd	19	79:21	21
8	$[RhCl(coe)_2]_2$ + **L1**	6	85:15	40
9	$[RhCl(coe)_2]_2$ + **L2**	6	88:12	9
10	$[RhCl(coe)_2]_2$ + **L3**	<5	--	--
11	PhZnCl	<5	--	--

[a]Reaction conditions: **1a** (0.10 mmol), **2a** (0.10 mmol), $[RhCl(coe)_2]_2$ (1.5 mol% dimer, 3 mol% Rh), ligand (3.0 mol%), KOH (0.15 mmol), and 2-Propanol (1.0 mL) at 30 °C (oil bath) for 10 h. [b]Isolated yield. [c]Determined by ^1H NMR spectroscopy. [d] % ee was determined by HPLC on a chiral stationary phase column.

Table S2 Rh-catalyzed addition to metallocene dicarbaldehydes **1a**: screening of temperatures[a].

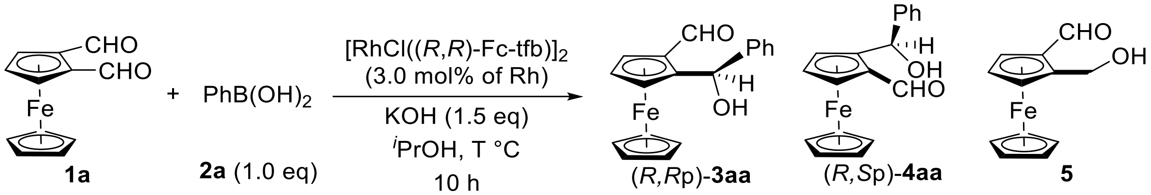

entry	T (°C)	conv[b] of **1a** (%)	yield (%)[c] **3aa** + **4aa**	ratio[b] of **3aa**/**4aa**
1	0	85	80	>50:1
2	30	100	93	>50:1
3	60	100	<5	--

[a]Reaction conditions: **1a** (0.10 mmol), **2a** (0.10 mmol), [RhCl((*R*,*R*)-Fc-tfb)]$_2$ (3.0 mol% Rh), KOH (0.15 mmol), and 2-propanol (1.0 mL) at T (°C) (oil bath) for 10 h. [b]Determined by ^{1}H NMR spectroscopy. [c]Isolated yield.

Table S3 Rh-catalyzed addition to metallocene dicarbaldehydes **1a**: screening of bases[a].

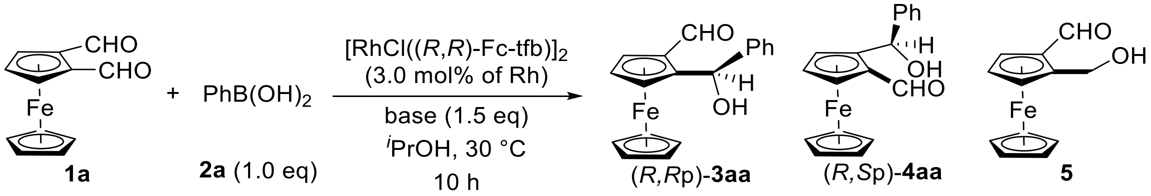

entry	base	conv[b] of **1a** (%)	yield (%)[c] **3aa** + **4aa**	ratio[b] of **3aa**/**4aa**
1	KOH	100	93	>50:1
2	K_2CO_3	37	33	>50:1
3	Et_3N	<5	--	--

[a]Reaction conditions: **1a** (0.10 mmol), **2a** (0.10 mmol), [RhCl((*R*,*R*)-Fc-tfb)]$_2$ (3.0 mol% Rh), base (0.15 mmol), and 2-Propanol (1.0 mL) at 30 °C (oil bath) for 10 h. [b]Determined by ^{1}H NMR spectroscopy. [c]Isolated yield.

Table S4 Rh-catalyzed addition to metallocene dicarbaldehydes **1a**: screening of solvents[a].

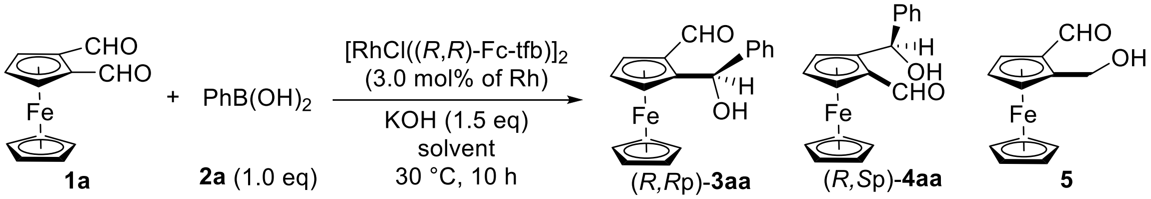

entry	solvent	conv[b] of **1a** (%)	yield (%)[c] **3aa** +	ratio[b] of **3aa**/**4aa**	ee[d] of **3aa**
1	*i*PrOH	100	93	>50:1	>99.5
2	*t*BuOH	100	94	>50:1	96
3	MeOH	22	20	>50:1	79
4	dioxane/H_2O (10/1)	92	90	>50:1	79

[a]Reaction conditions: **1a** (0.10 mmol), **2a** (0.10 mmol), [RhCl((*R*,*R*)-Fc-tfb)]$_2$ (3.0 mol% Rh), KOH (0.15 mmol), and solvent (1.0 mL) at 30 °C (oil bath) for 10 h. [b]Determined by ^{1}H NMR spectroscopy. [c]Isolated yield. [d]% ee was determined by HPLC on a chiral stationary phase column.

7. Synthetic transformations

7.1 Synthesis of Josiphos-type ligands

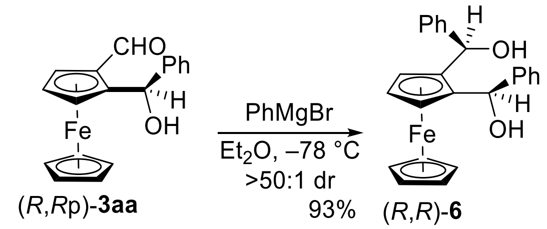

Under argon, to a solution of compound (*R*,*R*p)-**3aa** (160.1 mg, 0.50 mmol, 1.0 equiv, >99.5% ee) in Et_2O (5.0 mL), phenymagnesium bromide (0.77 M in THF, 1.3 mL, 2.0 equiv) was added dropwise at –78 °C, and the mixture was stirred at –78 °C for 2 h. The mixture was allowed to warm to room temperature slowly and stirred at room temperature for 8 h, quenched with H_2O (5.0 mL), and extracted with dichloromethane. The combined organic layers were dried over $MgSO_4$, filtered, and concentrated under vacuum. The residue was purified by flash column chromatography on silica gel (hexane/dichloromethane = 20:1) to give compound (*R*,*R*)-**6** (93% yield, 185.2 mg, 0.46 mmol) as a brown solid.

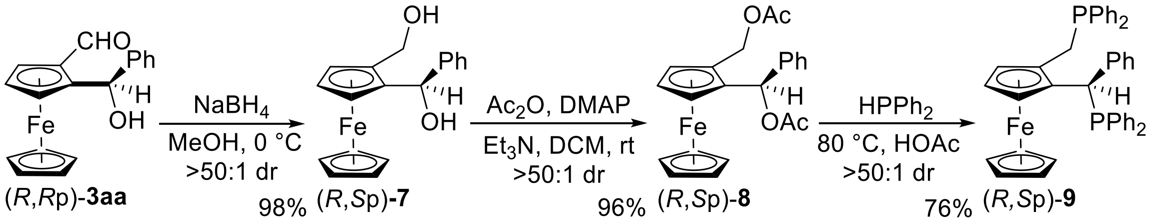

Under argon, to a solution of compound (*R*,*R*p)-**3aa** (121.7 mg, 0.38 mmol, 1.0 equiv) in MeOH (10 mL), $NaBH_4$ (21.6 mg, 0.57 mmol, 1.5 equiv) was added in several portions. The mixture was stirred at 0 °C for 1 h, quenched with H_2O (2.0 mL), and extracted with dichloromethane. The combined organic layers were dried over $MgSO_4$, filtered, and concentrated under vacuum. The residue was purified by flash column chromatography on silica gel (hexane/dichloromethane = 5:1) to give compound (*R*,*S*p)-**7** (98% yield, 120.1 mg, 0.37 mmol) as an orange solid.

An oven-dried Schlenk tube was charged with compound (*R*,*S*p)-**7** (96.6 mg, 0.30 mmol, 1.0 equiv), DMAP (29.8 mg, 0.24 mmol, 0.8 equiv), Et_3N (242.9 mg, 2.4 mmol, 8.0 equiv), and DCM (5.0 mL) under argon. Ac_2O (245.2 mg, 2.4 mmol, 8.0 equiv) was slowly

added to the Schlenk tube at room temperature. The mixture was stirred for 8 h, quenched with H_2O (3.0 mL), and extracted with ethyl acetate. The combined organic layers were dried over $MgSO_4$, filtered, and concentrated under vacuum. The residue was purified by flash column chromatography on silica gel (hexane/ethyl acetate = 5:1) to give compound (*R*,*S*p)-**8** (96% yield, 117.2 mg, 0.29 mmol) as a yellow oil.

Under argon, to a solution of compound (*R*,*S*p)-**8** (101.6 mg, 0.25 mmol, 1.0 equiv) in HOAc (2.0 mL), $HPPh_2$ (465.5 mg, 2.5 mmol, 10 equiv) was added dropwise. The mixture was stirred at 80 °C for 12 h, quenched with saturated $NaHCO_3$ solution (3.0 mL), and extracted with ethyl acetate. The combined organic layers were dried over $MgSO_4$, filtered, and concentrated under vacuum. The residue was purified by flash column chromatography on silica gel (hexane/ethyl acetate = 5:1) to give compound (*R*,*S*p)-**9** (76% yield, 125.1 mg, 0.19 mmol) as a yellow solid.

7.2 Synthesis of Josiphos without central chirality

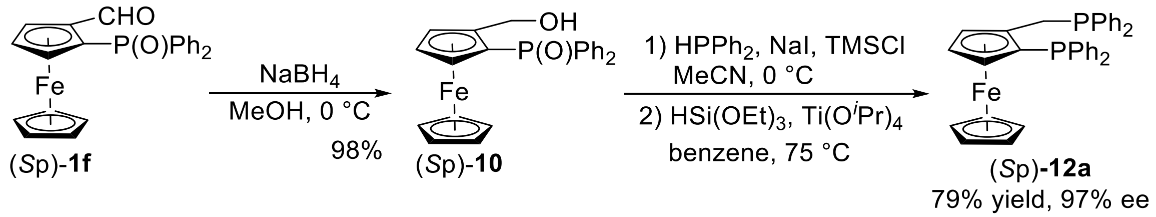

Under argon, to a solution of compound (*S*p)-**1f** (83.0 mg, 0.20 mmol, 1.0 equiv) in MeOH (5.0 mL), $NaBH_4$ (15.2 mg, 0.40 mmol, 2.0 equiv) was added in several portions. The mixture was stirred at 0 °C for 2 h, quenched with H_2O (1.0 mL), and extracted with dichloromethane. The combined organic layers were dried over $MgSO_4$, filtered, and concentrated under vacuum. The residue was purified by flash column chromatography on silica gel (hexane/dichloromethane = 2:1) to give compound (*S*p)-**10** (98% yield, 79.1 mg, 0.19 mmol) as a yellow solid.

An oven-dried Schlenk tube was charged with compound (*S*p)-**10** (62.4 mg, 0.15 mmol, 1.0 equiv), NaI (45.0 mg, 0.30 mmol, 2.0 equiv), and MeCN (5.0 mL) under argon. Chlorotrimethylsilane (40.8 mg, 0.38 mmol, 2.5 equiv) was added. After stirring for 10 min, $HPPh_2$ (56.0 mg, 0.30 mmol, 2.0 equiv) was added dropwise at 0 °C. The mixture was stirred at room temperature for 8 h, quenched with saturated $NaHCO_3$ solution (2.0 mL), and extracted with ethyl acetate. The combined organic layers were dried over $MgSO_4$,

filtered, and concentrated under vacuum without further purification. Under argon, to a solution of the residue in benzene (5.0 mL), titanium tetraisopropanolate (42.6 mg, 0.15 mmol 1.0 equiv) and triethoxysilane (266.1 mg, 1.62 mmol, 10.8 equiv) was added dropwise. The mixture was stirred at 75 °C for 12 h, filtered, and concentrated under vacuum. The residue was purified by flash column chromatography on silica gel (hexane/ethyl acetate = 20:1) to give compound (*S*p)-**12a** (79% yield, 67.4 mg, 0.12 mmol) as a red solid. The compounds (*S*p)-**12b** and compound (*S*p)-**12c** were synthesized using the same procedure.

7.3 Synthesis of Josiphos, PPFA, and other phosphorous ligands

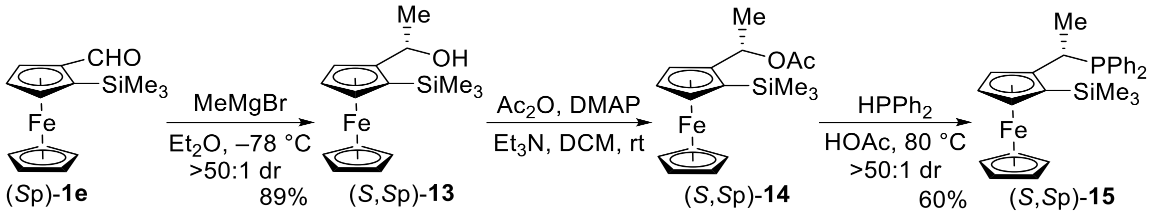

Under argon, to a solution of compound (*S*p)-**1e** (57.2 mg, 0.20 mmol, 1.0 equiv, >99.5% ee) in Et_2O (5.0 mL), MeMgBr (3.0 M in THF, 0.20 mL, 3.0 equiv) was added dropwise at –78 °C. The mixture was slowly warmed to room temperature and stirred at room temperature for 8 h. The mixture was quenched with H_2O (5.0 mL) and extracted with ethyl acetate. The combined organic layers were dried over $MgSO_4$, filtered, and concentrated under vacuum. The residue was purified by flash column chromatography on silica gel (hexane/ethyl acetate = 20:1) to give compound (*S*,*S*p)-**13** (89% yield, 53.8 mg, 0.18 mmol) as a yellow solid.

An oven-dried Schlenk tube was charged with compound (*S*,*S*p)-**13** (45.3 mg, 0.15 mmol, 1.0 equiv), DMAP (14.7 mg, 0.12 mmol, 0.8 equiv), Et_3N (121 mg, 1.2 mmol, 8.0 equiv), and DCM (5.0 mL) under argon. Ac_2O (30.6 mg, 0.30 mmol, 2.0 equiv) was slowly added to the mixture at room temperature. The mixture was stirred at room temperature for 8 h, quenched with H_2O (2.0 mL), and extracted with ethyl acetate. The combined organic layers were dried over $MgSO_4$, filtered, and concentrated under vacuum. Compound (*S*,*S*p)-**14** was obtained as a red oil, which was used for the next step without further purification.

Under argon, to a solution of compound (*S*,*S*p)-**14** (34.4 mg, 0.10 mmol, 1.0 equiv) in HOAc (2.0 mL), $HPPh_2$ (37.2 mg, 0.20 mmol, 2.0 equiv) was added dropwise. The mixture was stirred at 80 °C for 12 h, quenched with saturated $NaHCO_3$ solution (1.0 mL), and extracted with ethyl acetate. The combined organic layers were dried over $MgSO_4$, filtered, and concentrated under vacuum. The residue was purified by flash column chromatography on silica gel (hexane/ethyl acetate = 20:1) to give compound (*S*,*S*p)-**15** (60% yield, 28.2 mg, 0.060 mmol) as an orange solid.

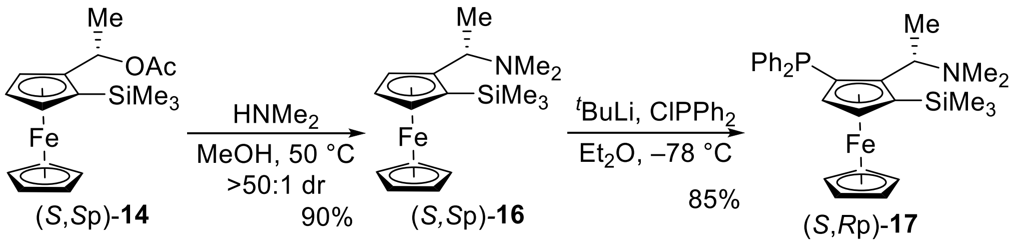

Under argon, to a solution of compound (*S*,*S*p)-**14** (68.9 mg, 0.20 mmol, 1.0 equiv) in MeOH (5.0 mL), $HNMe_2$ (40 wt.% in H_2O, 0.20 mL, 10 equiv) was added. The mixture was stirred at 50 °C for 6 h, quenched with phosphoric acid (1.0 M, 0.10 mL), and extracted with ethyl acetate. The combined organic layers were dried over $MgSO_4$, filtered, and concentrated under vacuum. The residue was purified by flash column chromatography on silica gel (hexane/ ethyl acetate/ triethylamine = 20:1:1%) to give compound (*S*,*S*p)-**16** (90% yield, 59.2 mg, 0.18 mmol) as an orange oil.

Under argon, to a solution of compound (*S*,*S*p)-**16** (49.5 mg, 0.15 mmol, 1.0 equiv) in Et_2O (5.0 mL), ^{t}BuLi (3.0 M in hexane, 76.7 µL, 0.23 mmol, 1.5 equiv) was added dropwise at −78 °C, and the mixture was stirred at room temperature for 2 h. $ClPPh_2$ (66.2 mg, 0.30 mmol, 2.0 equiv) was added to the mixture. The mixture was stirred at room temperature for 4 h, quenched with H_2O (1.0 mL), and extracted with ethyl acetate. The combined organic layers were dried over $MgSO_4$, filtered, and concentrated under vacuum. The residue was purified by flash column chromatography on silica gel (hexane/ethyl acetate/triethylamine = 10:1:1%) to give compound (*S*,*R*p)-**17** (85% yield, 65.4 mg, 0.13 mmol) as a red solid.

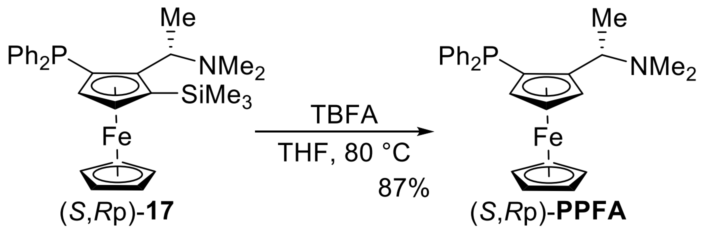

To a solution of (*S*,*R*p)-**17** (51.3 mg, 0.10 mmol, 1.0 equiv) in THF (2.0 mL), TBFA (1.0 M in THF, 0.50 mL, 5.0 equiv) was added. The mixture was stirred at 80 °C for 8 h, quenched with H_2O (2.0 mL), and extracted with ethyl acetate. The combined organic layers were dried over $MgSO_4$, filtered, and concentrated under vacuum. The residue was purified by flash column chromatography on silica gel (hexane/ethyl acetate = 20:1) to give compound (*S*,*R*p)-**PPFA** (87% yield, 38.4 mg, 0.087 mmol) as a red solid.

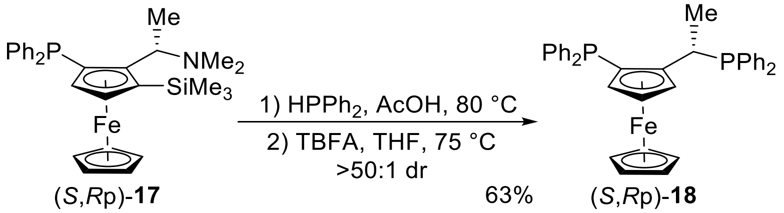

Under argon, to a solution of compound (*S*,*S*p)-**17** (25.7 mg, 0.05 mmol, 1.0 equiv) in HOAc (2.0 mL), $HPPh_2$ (18.6 mg, 0.10 mmol, 2.0 equiv) was added dropwise. The mixture was stirred at 80 ℃ for 12 h, quenched with saturated $NaHCO_3$ solution (2.0 mL), and extracted with ethyl acetate. The combined organic layers were dried over $MgSO_4$, filtered, and concentrated under vacuum. To a solution of the residue in THF (4.0 mL), TBFA (1.0 M in THF, 0.25 mL, 5.0 equiv) was added. The mixture was stirred at 75 °C for 8 h, quenched with H_2O (1.0 mL), and extracted with ethyl acetate. The combined organic layers were dried over $MgSO_4$, filtered, and concentrated under vacuum. The residue was purified by flash column chromatography on silica gel (hexane/ethyl acetate = 20:1) to give compound (*S*,*R*p)-**18** (63% yield, 18.3 mg, 0.031 mmol) as a yellow solid.

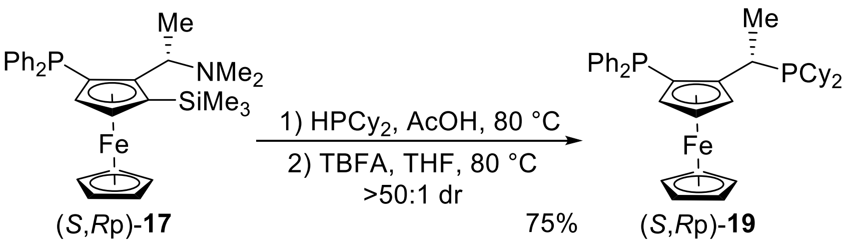

Under argon, to a solution of compound (*S*,*S*p)-**17** (25.9 mg, 0.05 mmol, 1.0 equiv) in HOAc (2.0 mL), $HPCy_2$ (19.9 mg, 0.10 mmol, 2.0 equiv) was added dropwise. The mixture was stirred at 80 ℃ for 12 h, quenched with saturated $NaHCO_3$ solution (2.0 mL), and extracted with ethyl acetate. The combined organic layers were dried over $MgSO_4$, filtered, and concentrated under vacuum. To a solution of the residue in THF (4.0 mL), TBFA (1.0 M in THF, 0.25 mL, 5.0 equiv) was added. The mixture was stirred at 80 °C for 8 h, quenched with H_2O (1.0 mL), and extracted with ethyl acetate. The combined organic layers were dried over $MgSO_4$, filtered, and concentrated under vacuum. The residue was purified by flash column chromatography on silica gel (hexane/ethyl acetate = 20:1) to give compound (*S*,*R*p)-**19** (75% yield, 21.9 mg, 0.038 mmol) as a red solid.

7.4 Palladium-catalyzed asymmetric allylic alkylation

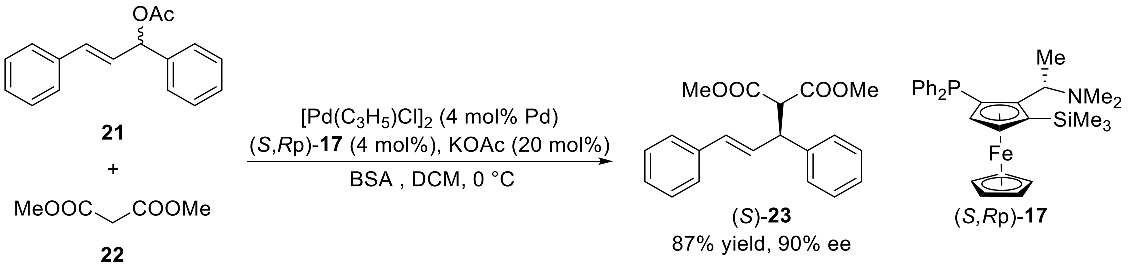

An oven-dried Schlenk tube was charged with compound ligand (*S*,*R*p)-**17** (2.1 mg, 0.004 mmol, 4 mol%), $[Pd(C_3H_5)Cl]_2$ (1.4 mg, 0.004 mmol, 4 mol%), and DCM (1.0 mL) under argon. The mixture was stirred at room temperature for 20 min. Compound **21** (25.2 mg, 0.10 mmol, 1.0 equiv) was added. After an additional 20 min, dimethyl malonate **22** (39.6 mg, 0.30 mmol, 3.0 equiv), BSA (40.7 mg, 0.20 mmol, 2.0 equiv), and KOAc (1.90 mg, 0.02 mmol, 20 mol%) were added. The mixture was stirred at 0 ℃ for 6 h, quenched with saturated NH_4Cl solution, and extracted with ethyl acetate. The combined organic layers were dried over $MgSO_4$, filtered, and concentrated under vacuum. The residue was

purified by flash column chromatography on silica gel (hexane/ethyl acetate = 10:1) to give compound (*S*)-**23** (87% yield, 28.2 mg, 0.087 mmol) as a colorless solid.

8. Characterization of the products

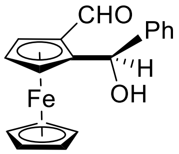

(R,Rp)-**3aa**

Compound (R,Rp)-3aa. (Table 2, entry 1, 29.8 mg, 93% yield, 0.093 mmol, a red solid, eluent: hexane/ethyl acetate (5/1), R_f = 0.4). The ee was measured by HPLC (Chiralpak IB N-5 column, hexane/2-propanol = 90/10, 1.0 mL/min, 230 nm, t_{major} = 16.7 min, t_{minor} = 14.9 min); $[\alpha]^{25}_{D}$ −7.4×10^2 (c 0.38, $CHCl_3$) for >99.5% ee. **^{1}H NMR** (600 MHz, $CDCl_3$) δ 9.96 (s, 1H), 7.50 (d, J = 7.6 Hz, 2H), 7.38 (t, J = 7.5 Hz, 2H), 7.30 (t, J = 7.4 Hz, 1H), 5.80 (s, 1H), 4.71–4.70 (m, 1H), 4.50 (d, J = 2.3 Hz, 1H), 4.48 (t, J = 2.7 Hz, 1H), 4.39 (s, 5H), 4.25 (s, 1H); **^{13}C NMR** (150 MHz, $CDCl_3$) δ 195.8, 142.3, 128.1, 127.6, 126.7, 97.4, 74.9, 73.1, 71.5, 70.4, 69.5, 69.1; **HRMS (ESI)** calcd for $C_{18}H_{16}FeO_2Na$ $[M+Na]^+$ 343.0392, found 343.0396.

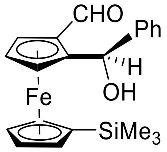

(R,Rp)-**3ba**

Compound (R,Rp)-3ba. (Table 2, entry 2, 34.5 mg, 88% yield, 0.088 mmol, a red solid, eluent: hexane /ethyl acetate (5/1), R_f = 0.4). The ee was measured by HPLC (Chiralpak IA column, hexane/2-propanol = 90/10, 1.0 mL/min, 230 nm, t_{major} = 9.2 min, t_{minor} = 8.1 min); $[\alpha]^{25}_{D}$ −6.6×10^2 (c 0.12, $CHCl_3$) for 94% ee. **^{1}H NMR** (600 MHz, $CDCl_3$): δ 9.96 (s, 1H), 7.49 (d, J = 7.0 Hz, 2H), 7.37 (t, J = 7.6 Hz, 2H), 7.30 (t, J = 7.4 Hz, 1H), 5.81 (s, 1H), 4.77 (s, 1H), 4.65 (s, 1H), 4.60 (s, 1H), 4.43 (t, J = 2.6 Hz, 1H), 4.35−4.34 (m, 2H), 4.29 (s, 1H), 4.12 (s, 1H), 0.19 (s, 9H). **^{13}C NMR** (150 MHz, $CDCl_3$): δ 195.6, 142.4, 128.2, 127.6, 126.7, 97.0, 75.1, 74.8, 74.5, 74.1, 73.5, 73.4, 71.8, 69.6, −0.4; **HRMS (ESI)** calcd for $C_{21}H_{24}FeO_2SiNa$ $[M+Na]^+$ 415.0787, found 415.0780.

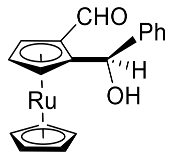

(*R*,*R*p)-**3ca**

Compound (*R,R*p)-3ca. (Table 2, entry 3, 28.1 mg, 77% yield, 0.077 mmol, a yellow solid, eluent: hexane/ethyl acetate (5/1), R_f = 0.4). The ee was measured by HPLC (Chiralpak IJ column, hexane/2-propanol = 90/10, 1.0 mL/min, 230 nm, t_{major} = 31.1 min, t_{minor} = 38.5 min); $[\alpha]^{25}_{D}$ −3.4×10^2 (*c* 0.20, $CHCl_3$) for 86% ee. **^{1}H NMR** (600 MHz, $CDCl_3$): δ 9.82 (s, 1H), 7.45 (d, *J* = 7.2 Hz, 2H), 7.34 (t, *J* = 7.5 Hz, 2H), 7.28 (t, *J* = 7.3 Hz, 1H), 5.74 (s, 1H), 4.98 (s, 1H), 4.75 (s, 5H), 4.72 (t, *J* = 2.6 Hz, 1H), 4.50 (s, 1H), 3.83 (d, *J* = 2.3 Hz, 1H); **^{13}C NMR** (150 MHz, $CDCl_3$): δ 192.0, 142.0, 128.1, 127.6, 126.6, 100.7, 81.4, 76.0, 74.1, 73.1, 72.9, 68.4; **HRMS (ESI)** calcd for $C_{18}H_{16}O_2RuNa$ $[M+Na]^+$ 389.0086, found 389.0095.

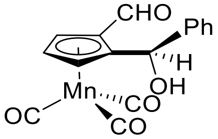

(*R*,*R*p)-**3da**

Compound (*R,R*p)-3da. (Table 2, entry 4, 24.7 mg, 73% yield, 0.073 mmol, a deep green solid, eluent: hexane/ethyl acetate (5/1), R_f= 0.4). The ee was measured by HPLC (Chiralpak IJ column, hexane/2-propanol = 90/10, 1.0 mL/min, 230 nm, t_{major} = 32.2 min, t_{minor} = 38.0 min); $[\alpha]^{25}_{D}$ −1.9×10^2 (*c* 0.17, $CHCl_3$) for 89% ee. **^{1}H NMR** (600 MHz, $CDCl_3$) δ 9.61 (s, 1H), 7.48 (d, *J* =7.5 Hz, 2H), 7.38 (t, *J* =7.5 Hz, 2H), 7.33 (t, *J* =7.5 Hz, 1H), 5.93 (s, 1H), 5.38 (s, 1H), 4.89 (s, 1H), 4.70 (s, 1H), 3.51 (s, 1H); **^{13}C NMR** (150 MHz, $CDCl_3$): δ 222.2, 188.4, 141.4, 128.7, 128.5, 126.7, 114.8, 90.0, 87.9, 86.0, 80.0, 68.8; **HRMS (ESI)** calcd for $C_{16}H_{11}MnO_5Na$ $[M+Na]^+$ 360.9879, found 360.9882.

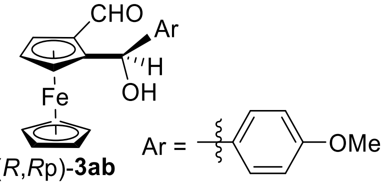

(*R*,*R*p)-**3ab**

Compound (*R,R*p)-3ab. (Table 2, entry 5, 30.1 mg, 86% yield, 0.086 mmol, a deep green solid, eluent: hexane/ethyl acetate (5/1), R_f= 0.4). The ee was measured by HPLC

(Chiralpak IB N-5 column, hexane/2-propanol = 90/10, 0.5 mL/min, 230 nm, t_{major} = 39.8 min, t_{minor} = 37.2 min); $[\alpha]^{25}_{D}$ −7.9×10^2 (*c* 0.12, $CHCl_3$) for >99.5% ee. **^{1}H NMR** (600 MHz, $CDCl_3$): δ 9.95 (s, 1H), 7.41 (d, *J* = 8.2 Hz, 2H), 6.90 (d, *J* = 8.2 Hz, 2H), 5.77 (s, 1H), 4.70 (s, 1H), 4.48 (t, *J* = 2.9 Hz, 1H), 4.38 (s, 5H), 4.32 (s, 1H), 4.30 (s, 1H), 3.82 (s, 3H); **^{13}C NMR** (150 MHz, $CDCl_3$): δ 195.7, 159.1, 134.7, 127.9, 113.6, 97.6, 75.0, 74.7, 73.1, 71.4, 70.4, 69.2, 55.3; HRMS (ESI) calcd for $C_{19}H_{18}FeO_3Na$ [M+Na]+ 373.0497, found 373.0503.

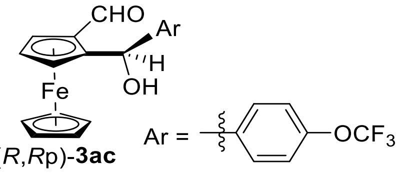

(*R*,*R*p)-**3ac**

Compound (*R*,*R*p)-3ac. (Table 2, entry 6, 33.1 mg, 82% yield, 0.082 mmol, a red solid, eluent: hexane/ethyl acetate (5/1), R_f = 0.4). The ee was measured by HPLC (Chiralpak IC column, hexane/2-propanol = 80/20, 1.0 mL/min, 230 nm, t_{major} = 6.8 min, t_{minor} = 5.7 min); $[\alpha]^{25}_{D}$ −5.8×10^2 (*c* 0.070, $CHCl_3$) for 92% ee. **^{1}H NMR** (600 MHz, $CDCl_3$): δ 9.95 (s, 1H), 7.54 (d, *J* = 8.6 Hz, 2H), 7.22 (d, *J* = 8.2 Hz, 2H), 5.81 (s, 1H), 4.73–4.70 (m, 1H), 4.58 (d, *J* = 2.2 Hz, 1H), 4.50 (t, *J* = 2.7 Hz, 1H), 4.39 (s, 5H), 4.23 (s, 1H); **^{13}C NMR** (150 MHz, $CDCl_3$): δ 195.8, 148.6, 141.0, 128.2, 120.6, 120.5 (q, $J_{C,^{19}F}$ = 255.2 Hz), 96.9, 75.0, 74.6, 73.5, 71.6, 70.4, 68.9; **HRMS (ESI)** calcd for $C_{19}H_{15}F_3FeO_3Na$ $[M+Na]^+$ 427.0215, found 427.0218.

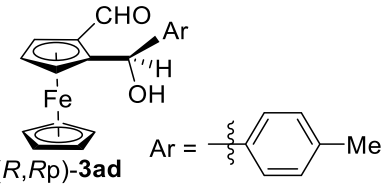

(*R*,*R*p)-**3ad**

Compound (*R*,*R*p)-3ad. (Table 2, entry 7, 28.4 mg, 85% yield, 0.085 mmol, a red solid, eluent: hexane/ethyl acetate (5/1), R_f = 0.4). The ee was measured by HPLC (Chiralpak IA column, hexane/2-propanol = 90/10, 1.0 mL/min, 230 nm, t_{major} = 16.2 min, t_{minor} = 14.6 min); $[\alpha]^{25}_{D}$ −6.0×10^2 (*c* 0.13, $CHCl_3$) for 99% ee. **^{1}H NMR** (600 MHz, $CDCl_3$): δ 9.96 (s, 1H), 7.38 (d, *J* = 7.6 Hz, 2H), 7.18 (d, *J* = 7.6 Hz, 2H), 5.77 (s, 1H), 4.70 (s, 1H), 4.48 (s, 1H), 4.38 (s, 5+1H), 4.28 (s, 1H), 2.36 (s, 3H); **^{13}C NMR** (150 MHz, $CDCl_3$): δ

195.8, 139.4, 137.2, 128.9, 126.7, 97.5, 74.90, 74.88, 73.1, 71.5, 70.4, 69.4, 21.2. **HRMS (ESI)** calcd for $C_{19}H_{18}FeO_2Na$ [M+Na]$^+$ 357.0548, found 357.0551.

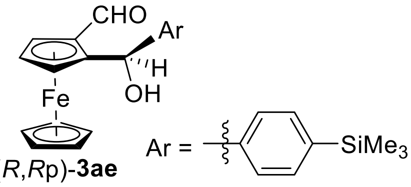

(*R*,*R*p)-**3ae**

Compound (*R*,*R*p)-3ae. (Table 2, entry 8, 34.1 mg, 87% yield, 0.087 mmol, a red solid, eluent: hexane/ethyl acetate (5/1), R_f = 0.4). The ee was measured by HPLC (Chiralpak IJ column, hexane/2-propanol = 95/5, 0.8 mL/min, 230 nm, t_{major} = 21.7 min, t_{minor} = 25.2 min); $[\alpha]^{25}_{D}$ −6.3×10^2 (*c* 0.29, $CHCl_3$) for >99.5% ee. **^{1}H NMR** (600 MHz, $CDCl_3$) δ 9.97 (s, 1H), 7.52 (d, *J* = 7.4 Hz, 2H), 7.47 (d, *J* = 7.6 Hz, 2H), 5.80 (s, 1H), 4.70 (s, 1H), 4.49 (s, 1H), 4.39 (s, 5H), 4.33 (s, 1+1H), −0.27 (s, 9H); **^{13}C NMR** (150 MHz, $CDCl_3$): δ 195.6, 142.9, 139.7, 133.2, 126.1, 97.2, 75.1, 74.7, 73.0, 71.5, 70.4, 69.6, −1.1. **HRMS (ESI)** calcd for $C_{21}H_{24}FeO_2SiNa$ [M+Na]$^+$ 415.0787, found 415.0791.

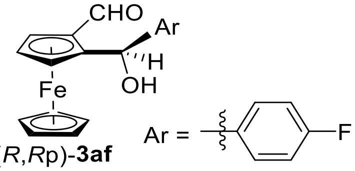

(*R*,*R*p)-**3af**

Compound (*R*,*R*p)-3af. (Table 2, entry 9, 27.7 mg, 82% yield, 0.082 mmol, a red solid, eluent: hexane/ethyl acetate (5/1), R_f = 0.4). The ee was measured by HPLC (Chiralpak IA column, hexane/2-propanol = 90/10, 1.0 mL/min, 230 nm, t_{major} = 15.9 min, t_{minor} = 15.0 min); $[\alpha]^{25}_{D}$ −7.4×10^2 (*c* 0.18, $CHCl_3$) for 95% ee. **^{1}H NMR** (600 MHz, $CDCl_3$): δ 9.94 (s, 1H), 7.48 (dd, *J* = 8.5 Hz, 5.5 Hz, 2H), 7.06 (dd, *J* = 8.6 Hz, 8.5 Hz, 2H), 5.78 (s, 1H), 4.71 (s, 1H), 4.57 (s, 1H), 4.49 (t, *J* = 2.8 Hz, 1H), 4.38 (s, 5H), 4.22 (s, 1H); **^{13}C** NMR (150 MHz, $CDCl_3$): δ 195.8, 162.2 (d, $J_{C,^{19}F}$ = 244.0 Hz), 138.2 (d, $J_{C,^{19}F}$ = 3.2 Hz), 128.4 (d, $J_{C,^{19}F}$ = 8.0 Hz), 115.0 (d, $J_{C,^{19}F}$ = 21.2 Hz), 97.2, 74.9, 74.7, 73.4, 71.5, 70.4, 68.9; **HRMS (ESI)** calcd for $C_{18}H_{15}FFeO_2Na$ [M+Na]$^+$ 361.0298, found 361.0301.

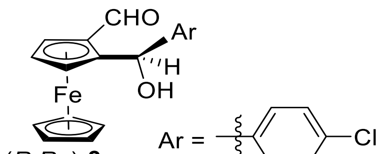

(*R*,*R*p)-**3ag**

Compound (*R*,*R*p)-3ag. (Table 2, entry 10, 28.4 mg, 80% yield, 0.080 mmol, a red solid, eluent: hexane/ethyl acetate (5/1), R_f = 0.4). The ee was measured by HPLC (Chiralpak IA column, hexane/2-propanol = 90/10, 0.6 mL/min, 230 nm, t_{major} = 36.5 min, t_{minor} = 30.8 min); $[\alpha]^{25}_{D}$ −6.9×10^2 (*c* 0.19, $CHCl_3$) for 96% ee. **^{1}H NMR** (600 MHz, $CDCl_3$): δ 9.94 (s, 1H), 7.45 (d, *J* = 8.6 Hz, 2H), 7.35 (d, *J* = 8.3 Hz, 2H), 5.78 (s, 1H), 4.71 (s, 1H), 4.63 (s, 1H), 4.49 (t, *J* = 2.6 Hz, 1H), 4.38 (s, 5H), 4.21 (s, 1H); **^{13}C NMR** (150 MHz, $CDCl_3$): δ 196.0, 140.8, 133.2, 128.3, 128.2, 97.0, 74.9, 73.5, 71.6, 71.2, 70.4, 68.9; **HRMS (ESI)** calcd for $C_{18}H_{15}ClFeO_2Na$ $[M+Na]^+$ 377.0002, found 377.0005.

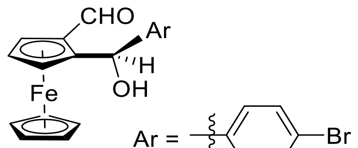

3ah

Compound (*R*,*R*p)-3ah. (Table 2, entry 11, 31.1 mg, 78% yield, 0.078 mmol, a red solid, eluent: hexane/ethyl acetate (5/1), R_f = 0.4). The ee was measured by HPLC (Chiralpak IA column, hexane/2-propanol = 90/10, 1.0 mL/min, 230 nm, t_{major} = 19.7 min, t_{minor} = 16.8 min); $[\alpha]^{25}_{D}$ −8.9×10^2 (*c* 0.15, $CHCl_3$) for 98% ee. **^{1}H NMR** (600 MHz, $CDCl_3$): δ 9.94 (s, 1H), 7.51 (d, *J* = 8.1 Hz, 2H), 7.39 (d, *J* = 8.1 Hz, 2H), 5.75 (s, 1H), 4.71 (s, 1H), 4.65 (s, 1H), 4.49 (t, *J* = 2.7 Hz, 1H), 4.38 (s, 5H), 4.20 (s, 1H); **^{13}C NMR** (150 MHz, $CDCl_3$): δ 196.0, 141.3, 131.3, 128.5, 121.4, 96.9, 74.9, 74.8, 73.6, 71.6, 70.4, 68.9; **HRMS (ESI)** calcd for $C_{18}H_{15}BrFeO_2Na$ $[M+Na]^+$ 420.9497, found 420.9508. The absolute configuration was determined by X–ray analysis (CCDC 2403094).

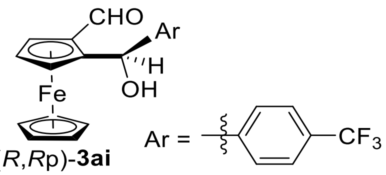

(*R*,*R*p)-**3ai**

Compound (*R*,*R*p)-3ai. (Table 2, entry 12, 29.5 mg, 76% yield, 0.076 mmol, a red solid, eluent: hexane/ethyl acetate (5/1), R_f = 0.4). The ee was measured by HPLC (Chiralpak IA column, hexane/2-propanol = 90/10, 1.0 mL/min, 230 nm, t_{major} = 13.5 min,

t_{minor} = 12.5 min); $[\alpha]^{25}_{D}$ −7.4×10^2 (*c* 0.14, $CHCl_3$) for 84% ee. **^{1}H NMR** (600 MHz, $CDCl_3$): δ 9.96 (s, 1H), 7.64 (s, 2+2H), 5.86 (s, 1H), 4.73–4.71 (m, 1H), 4.69 (d, *J* = 2.2 Hz, 1H), 4.50 (t, *J* = 2.6 Hz, 1H), 4.39 (s, 5H), 4.19 (s, 1H); **^{13}C NMR** (150 MHz, $CDCl_3$) δ 195.9, 146.3, 129.8 (q, $J_{C,}{}^{19}{}_{F}$ = 32.1 Hz), 127.1, 125.1 (q, $J_{C,}{}^{19}{}_{F}$ = 3.8 Hz), 124.2 (q, $J_{C,}{}^{19}{}_{F}$ = 270.3 Hz), 96.6, 75.0, 74.6, 73.6, 71.7, 70.5, 69.0; **HRMS (ESI)** calcd for $C_{19}H_{15}F_3FeO_2Na$ $[M+Na]^+$ 411.0266, found 411.0270.

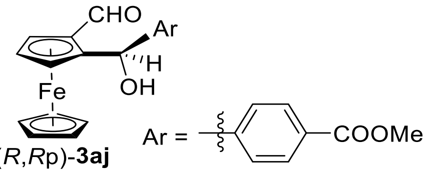

(*R*,*R*p)-**3aj**

Compound (*R*,*R*p)-3aj. (Table 2, entry 13, 30.3 mg, 80% yield, 0.080 mmol, a red solid, eluent: hexane/ethyl acetate (5/1), R_f = 0.4). The ee was measured by HPLC (Chiralpak IC column, hexane/2-propanol = 80/20, 1.0 mL/min, 230 nm, t_{major} = 22.6 min, t_{minor} = 18.9 min); $[\alpha]^{25}_{D}$ −7.6×10^2 (*c* 0.11, $CHCl_3$) for 89% ee. **^{1}H NMR** (600 MHz, $CDCl_3$): δ 9.95 (s, 1H), 8.06 (d, *J* = 8.1 Hz, 2H), 7.60 (d, *J* = 8.0 Hz, 2H), 5.83 (s, 1H), 4.82 (d, *J* = 2.2 Hz, 1H), 4.71 (dd, *J* = 2.6 Hz, 1.6 Hz, 1H), 4.48 (t, *J* = 2.7 Hz, 1H), 4.38 (s, 5H), 4.15 (s, 1H), 3.93 (s, 3H); **^{13}C NMR** (150 MHz, $CDCl_3$): δ 196.1, 167.0, 147.3, 129.5, 129.4, 126.8, 96.8, 75.0, 73.6, 71.6, 70.9, 70.5, 69.1, 52.1; **HRMS (ESI)** calcd for $C_{20}H_{18}FeO_4Na$ $[M+Na]^+$ 401.0447, found 401.0454.

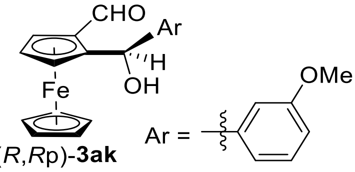

(*R*,*R*p)-**3ak**

Compound (*R*,*R*p)-3ak. (Table 2, entry 14, 27.3 mg, 78% yield, 0.078 mmol, a red solid, eluent: hexane/ethyl acetate (5/1), R_f = 0.4). The ee was measured by HPLC (Chiralpak IB N-5 column, hexane/2-propanol = 90/10, 0.8 mL/min, 230 nm, t_{major} = 34.9 min, t_{minor} = 41.7 min); $[\alpha]^{25}_{D}$ −6.2×10^2 (*c* 0.19, $CHCl_3$) for 89% ee. **^{1}H NMR** (600 MHz, $CDCl_3$): δ 9.96 (s, 1H), 7.28 (t, *J* = 7.9 Hz, 1H), 7.09 (s, 1H), 7.06 (d, *J* = 7.6 Hz, 1H), 6.85 (dd, *J* = 8.2 Hz, 2.6 Hz, 1H), 5.77 (s, 1H), 4.71−4.69 (m, 1H), 4.52 (s, 1H), 4.49 (t, *J* = 2.6 Hz, 1H), 4.39 (s, 5H), 4.29 (s, 1H), 3.83 (s, 3H); **^{13}C NMR** (150 MHz, $CDCl_3$): δ 195.8,

159.6, 143.9, 129.1, 119.2, 113.2, 112.2, 97.3, 74.9, 73.1, 71.5, 71.2, 70.4, 69.4, 55.2. **HRMS (ESI)** calcd for $C_{19}H_{18}FeO_3Na$ $[M+Na]^+$ 373.0497, found 373.0502.

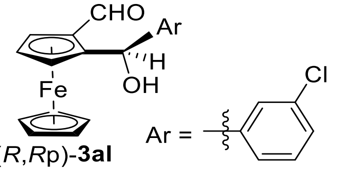

(*R*,*R*p)-**3al**

Compound (*R*,*R*p)-3al. (Table 2, entry 15, 28.7 mg, 81% yield, 0.081 mmol, a red solid, eluent: hexane/ethyl acetate (5/1), R_f = 0.4). The ee was measured by HPLC (Chiralpak IA column, hexane/2-propanol = 90/10, 1.0 mL/min, 230 nm, t_{major} = 19.2 min, t_{minor} = 14.0 min); $[\alpha]^{25}_D$ −8.3×10^2 (*c* 0.25, $CHCl_3$) for 89% ee. **^{1}H NMR** (600 MHz, $CDCl_3$): δ 9.94 (s, 1H), 7.54 (s, 1H), 7.36 (d, *J* = 6.9 Hz, 1H), 7.31−7.27 (m, 2H), 5.75 (s, 1H), 4.71 (s, 1H), 4.68 (s, 1H), 4.50 (t, *J* = 2.8 Hz, 1H), 4.39 (s, 5H), 4.22 (s, 1H); **^{13}C NMR** (150 MHz, $CDCl_3$): δ 195.9, 144.3, 134.1, 129.4, 127.7, 127.0, 125.1, 96.8, 74.9, 73.5, 71.6, 71.2, 70.4, 69.0. **HRMS (ESI)** calcd for $C_{18}H_{15}ClFeO_2Na$ $[M+Na]^+$ 377.0002, found 377.0007.

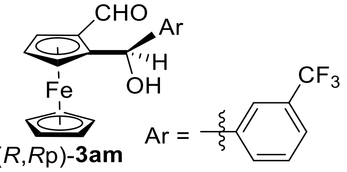

(*R*,*R*p)-**3am**

Compound (*R*,*R*p)-3am. (Table 2, entry 16, 30.7 mg, 79% yield, 0.079 mmol, a red solid, eluent: hexane/ethyl acetate (5/1), R_f = 0.4). The ee was measured by HPLC (Chiralpak IA column, hexane/2-propanol = 90/10, 1.0 mL/min, 230 nm, t_{major} = 12.4 min, t_{minor} = 10.0 min); $[\alpha]^{25}_D$ −7.4×10^2 (*c* 0.14, $CHCl_3$) for 90% ee. **^{1}H NMR** (600 MHz, $CDCl_3$): δ 9.95 (s, 1H), 7.86 (s, 1H), 7.67 (d, *J* = 7.7 Hz, 1H), 7.59 (d, *J* = 7.8 Hz, 1H), 7.50 (t, *J* = 7.7 Hz, 1H), 5.82 (s, 1H), 4.91 (s, 1H), 4.72 (s, 1H), 4.50 (t, *J* = 2.8 Hz, 1H), 4.39 (s, 5H), 4.10 (s, 1H); **^{13}C NMR** (150 MHz, $CDCl_3$) δ 196.1, 143.3, 130.6 (q, $J_{C,^{19}F}$ = 32.0 Hz), 130.3, 128.5, 124.4 (q, $J_{C,^{19}F}$ = 3.8 Hz), 124.2 (q, $J_{C,^{19}F}$ = 270.7 Hz), 123.7 (q, $J_{C,^{19}F}$ = 3.8 Hz), 96.9, 75.0, 74.8, 73.7, 71.7, 70.4, 69.0; **HRMS (ESI)** calcd for $C_{19}H_{15}F_3FeO_2Na$ $[M+Na]^+$ 411.0266, found 411.0271.

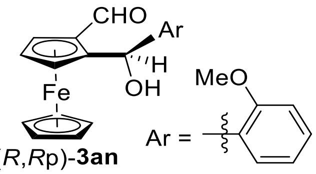

(*R*,*R*p)-**3an**

Compound (*R*,*R*p)-3an. (Table 2, entry 17, 29.1 mg, 83% yield, 0.083 mmol, a red solid, eluent: hexane/ethyl acetate (5/1), R_f = 0.4). The ee was measured by HPLC (Chiralpak IB N-5 column, hexane/2-propanol = 90/10, 0.5 mL/min, 230 nm, t_{major} =26.8 min, t_{minor} = 31.6 min); $[\alpha]^{25}_{D}$ −6.5×10^2 (*c* 0.18, $CHCl_3$) for >99.5% ee. **^{1}H NMR** (600 MHz, $CDCl_3$): δ 9.99 (s, 1H), 7.58 (dd, *J* = 7.4 Hz, 1.7 Hz, 1H), 7.29−7.26 (m, 1H), 7.01 (t, *J* = 7.5 Hz, 1H), 6.88 (d, *J* = 8.2 Hz, 1H), 6.07 (d, *J* = 3.1 Hz, 1H), 4.73−4.70 (m, 1H), 4.58 (d, *J* = 3.1 Hz, 1H), 4.50 (t, *J* = 2.7 Hz, 1H), 4.36 (s, 5H), 4.32 (s, 1H), 3.80 (s, 3H); **^{13}C NMR** (150 MHz, $CDCl_3$): δ195.5, 156.1, 131.1, 128.5, 127.2, 120.7, 110.2, 96.4, 75.2, 74.8, 71.9, 71.4, 70.3, 64.1, 55.3; **HRMS (ESI)** calcd for $C_{19}H_{18}FeO_3Na$ $[M+Na]^+$ 373.0497, found 373.0503.

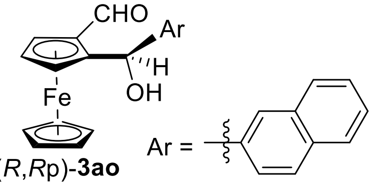

(*R*,*R*p)-**3ao**

Compound (*R*,*R*p)-3ao. (Table 2, entry 18, 29.6 mg, 80% yield, 0.080 mmol, a red solid, eluent: hexane/ethyl acetate (5/1), R_f = 0.4). The ee was measured by HPLC (Chiralpak IB N-5 column, hexane/2-propanol = 80/20, 0.8 mL/min, 230 nm, t_{major} = 17.0 min, t_{minor} = 16.0 min); $[\alpha]^{25}_{D}$ −7.9×10^2 (*c* 0.14, $CHCl_3$) for 91% ee. **^{1}H NMR** (600 MHz, $CDCl_3$): δ 9.99 (s, 1H), 7.98 (s, 1H), 7.89−7.83 (m, 3H), 7.62 (dd, *J* = 8.4 Hz, 1.7 Hz, 1H), 7.51−7.45 (m, 2H), 5.97 (s, 1H), 4.72−4.70 (m, 1H), 4.66 (d, *J* = 2.2 Hz, 1H), 4.47 (t, *J* = 2.7 Hz, 1H), 4.40 (s, 5H), 4.25 (s, 1H); **^{13}C NMR** (150 MHz, $CDCl_3$): δ 195.8, 139.8, 133.3, 133.1, 128.1, 127.8, 127.7, 126.0, 125.8, 125.5, 125.1, 97.4, 75.1, 73.2, 71.5, 70.5, 69.6; **HRMS (ESI)** calcd for $C_{22}H_{18}FeO_2Na$ $[M+Na]^+$ 393.0548, found 393.0553.

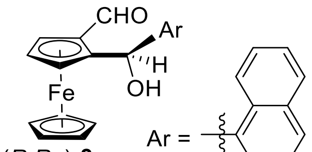

(*R*,*R*p)-**3ap**

Compound (*R,R*p)-3ap. (Table 2, entry 19, 28.9 mg, 78% yield, 0.078 mmol, a red solid, eluent: hexane/ethyl acetate (5/1), R_f = 0.4). The ee was measured by HPLC (Chiralpak IF column, hexane/2-propanol = 85/15, 1.0 mL/min, 230 nm, t_{major} = 44.8 min, t_{minor} = 13.9 min); $[\alpha]^{25}_D$ −1.0×10^3 (*c* 0.24, $CHCl_3$) for >99.5% ee. **^{1}H NMR** (600 MHz, $CDCl_3$): δ 10.0 (s, 1H), 7.98 (d, *J* = 7.2 Hz, 1H), 7.89 (d, *J* = 8.1 Hz, 1H), 7.85 (d, *J* = 8.2 Hz, 1H), 7.82 (d, *J* = 8.4 Hz, 1H), 7.59 (t, *J* = 7.7 Hz, 1H), 7.46 (t, *J* = 7.4 Hz, 1H), 7.42 (t, *J* = 7.6 Hz, 1H), 6.46 (s, 1H), 5.25 (s, 1H), 4.71 (s, 1H), 4.40–4.39 (m, 1+5H), 3.91 (s, 1H); **^{13}C NMR** (150 MHz, $CDCl_3$): δ 196.6, 137.9, 133.4, 130.9, 128.7, 128.0, 126.0, 125.6, 125.4, 124.1, 123.6, 97.0, 74.7, 73.2, 71.6, 71.5, 70.5, 65.4; **HRMS (ESI)** calcd for $C_{22}H_{18}FeO_2Na$ $[M+Na]^+$ 393.0548, found 393.0552.

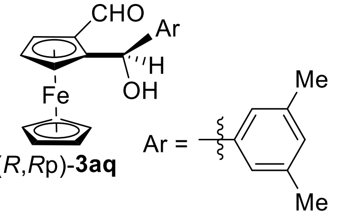

(*R*,*R*p)-**3aq**

Compound (*R,R*p)-3aq. (Table 2, entry 20, 29.6 mg, 85% yield, 0.085 mmol, a red solid, eluent: hexane/ethyl acetate (5/1), R_f = 0.4). The ee was measured by HPLC (Chiralpak IA column, hexane/2-propanol = 90/10, 0.5 mL/min, 230 nm, t_{major} = 30.6 min, t_{minor} = 25.8 min); $[\alpha]^{25}_D$ −6.2×10^2 (*c* 0.19, $CHCl_3$) for 92% ee. **^{1}H NMR** (600 MHz, $CDCl_3$): δ 9.97 (s, 1H), 7.10 (s, 2H), 6.93 (s, 1H), 5.73 (d, *J* = 2.2 Hz, 1H), 4.72–4.68 (m, 1H), 4.49 (t, *J* = 2.7 Hz, 1H), 4.38 (s, 5H), 4.33 (s, 1H), 4.28 (d, *J* = 2.3 Hz, 1H), 2.33 (s, 6H); **^{13}C NMR** (150 MHz, $CDCl_3$): δ 195.6, 142.3, 137.6, 129.2, 124.6, 97.5, 75.1, 74.8, 72.9, 71.5, 70.4, 69.6, 21.4; **HRMS (ESI)** calcd for $C_{20}H_{20}FeO_2Na$ $[M+Na]^+$ 371.0705, found 371.0708.

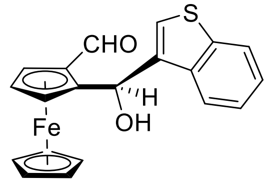

(*R*,*R*p)-**3ar**

Compound (*R,R*p)-3ar. (Table 2, entry 21, 21.8 mg, 58% yield, 0.058 mmol, a red solid, eluent: hexane/ethyl acetate (5/1), R_f = 0.4). The ee was measured by HPLC (Chiralpak IJ column, hexane/2-propanol = 90/10, 1.0 mL/min, 230 nm, t_{major} = 75.0 min, t_{minor} = 53.1 min); $[\alpha]^{25}_{D}$ −5.0×10^2 (*c* 0.11, $CHCl_3$) for 85% ee. **^{1}H NMR** (600 MHz, $CDCl_3$): δ 9.97 (s, 1H), 7.88 (d, *J* = 7.4 Hz, 1H), 7.68 (d, *J* = 7.5 Hz, 1H), 7.63 (s, 1H), 7.36−7.31 (m, 2H), 6.16 (s, 1H), 4.96 (s, 1H), 4.71 (s, 1H), 4.46 (s, 1H), 4.38 (s, 5H), 4.24 (s, 1H); **^{13}C NMR** (150 MHz, $CDCl_3$): δ 196.1, 140.5, 137.7, 137.6, 124.3, 124.0, 123.5, 122.8, 122.3, 96.0, 75.8, 74.9, 73.2, 71.6, 70.5, 65.0; **HRMS (ESI)** calcd for $C_{20}H_{16}FeO_2SNa$ $[M+Na]^+$ 399.0112, found 399.0116.

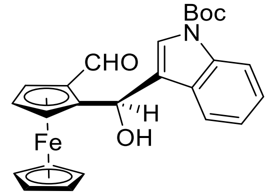

(*R*,*R*p)-**3as**

Compound (*R,R*p)-3as. (Table 2, entry 22, 26.2 mg, 57% yield, 0.057 mmol, a red solid, eluent: hexane/ethyl acetate (5/1), R_f = 0.4). The ee was measured by HPLC (Chiralpak IB N-5 column, hexane/2-propanol = 90/10, 0.8 mL/min, 230 nm, t_{major} = 16.4 min, t_{minor} = 18.4 min); $[\alpha]^{25}_{D}$ −5.9×10^2 (*c* 0.31, $CHCl_3$) for 97% ee. **^{1}H NMR** (600 MHz, $CDCl_3$): δ 9.98 (s, 1H), 8.17 (d, *J* = 7.9 Hz, 1H), 7.70 (s, 1H), 7.52 (d, *J* = 7.9 Hz, 1H), 7.31 (t, *J* = 7.8 Hz, 1H), 7.21 (t, *J* = 7.5 Hz, 1H), 6.10 (s, 1H), 4.72 (s, 1H), 4.55−4.45 (m, 3H), 4.40 (s, 5H), 1.67 (s, 9H); **^{13}C** NMR (150 MHz, $CDCl_3$): δ 195.8, 149.7, 135.5, 129.0, 124.4, 123.7, 122.8, 122.6, 119.7, 115.3, 96.0, 83.7, 75.2, 75.0, 73.1, 71.6, 70.4, 63.5, 28.2; **HRMS (ESI)** calcd for $C_{25}H_{25}FeNO_4Na$ $[M+Na]^+$ 482.1025, found 482.1026.

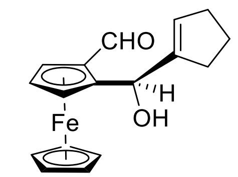

(R,Rp)-**3at**

Compound (R,Rp)-3at. (Table 2, entry 23, 24.2 mg, 78% yield, 0.078 mmol, a red solid, eluent: hexane/ethyl acetate (5/1), R_f = 0.4). The ee was measured by HPLC (Chiralpak IC column, hexane/2-propanol = 80/20, 1.0 mL/min, 230 nm, t_{major} = 10.9 min, t_{minor} = 9.6 min); $[\alpha]^{25}_{D}$ −4.6×10^{2} (c 0.15, $CHCl_3$) for 94% ee. **^{1}H NMR** (600 MHz, $CDCl_3$): δ 9.96 (s, 1H), 5.67 (s, 1H), 5.45 (s, 1H), 4.72 (s, 1H), 4.65 (s, 1H), 4.55 (s, 1H), 4.35 (s, 5H), 3.39 (s, 1H), 2.44−2.32 (m, 4H), 1.92−1.87 (m, 2H); **^{13}C NMR** (150 MHz, $CDCl_3$): δ 194.7, 145.3, 127.1, 95.4, 73.0, 71.8, 71.4, 71.0, 70.3, 66.8, 32.2, 31.9, 23.2; **HRMS (ESI)** calcd for $C_{17}H_{18}FeO_2Na$ $[M+Na]^+$ 333.0548, found 333.0552.

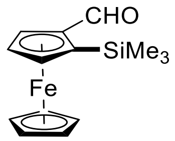

(Sp)-**1e**

Compound **(Sp)-1e [32648-55-8]**. (Scheme 2, 12.3 mg, 43% yield, 0.043 mmol, a red solid, eluent: hexane/ethyl acetate (20/1), R_f = 0.6). The ee was measured by HPLC (Chiralpak IC column, hexane/2-propanol = 95/5, 1.0 mL/min, 230 nm, t_{major} = 8.7 min, t_{minor} = 8.0 min); $[\alpha]^{25}_{D}$ −1.5×10^{2} (c 0.25, EtOH) for >99.5% ee. Reported value[8] for 99.8% ee (Sp)-**1e** is $[\alpha]^{20}_{D}$ −2.0×10^{2} (c 0.24, EtOH). **^{1}H NMR** (600 MHz, $CDCl_3$): δ 10.0 (s, 1H), 4.96 (s, 1H), 4.70 (s, 1H), 4.51 (s, 1H), 4.24 (s, 5H), 0.31 (s, 9H). The spectral data are in agreement with reported literature values.[8]

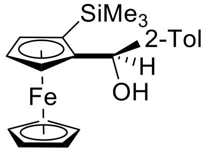

3e

Compound (R,Rp)-3e. (Scheme 2, 17.8 mg, 47% yield, 0.047 mmol, a yellow solid, eluent: hexane/ethyl acetate (20/1), R_f = 0.4). The ee was measured by HPLC (Chiralpak IC column, hexane/2-propanol = 95/5, 1.0 mL/min, 230 nm, t_{major} = 6.7 min, t_{minor} = 5.2 min); $[\alpha]^{25}_{D}$ −17 (c 0.43, $CHCl_3$) for 96% ee. **^{1}H NMR** (600 MHz, $CDCl_3$): δ 7.16−7.13

(m, 3H), 7.09 (t, J = 7.3 Hz, 1H), 5.64 (s, 1H), 4.47 (s, 1H), 4.43 (s, 1H), 4.32 (s, 5H), 4.26 (s, 1H), 2.39 (s, 3H), 2.26 (s, 1H), 0.08 (s, 9H); **^{13}C NMR** (150 MHz, $CDCl_3$): δ 141.1, 136.1, 130.2, 127.6, 127.0, 125.7, 99.0, 75.2, 71.2, 70.7, 69.9, 69.0, 68.7, 19.1, 0.1. HRMS (MALDl-TOF) m/z: $[M]^{\bullet+}$ Calcd for $C_{21}H_{26}FeSiO^{\bullet+}$ 378.1097; Found 378.1106.

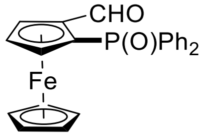

(*S*p)-**1f**

Compound (*S*p)-1f. (Scheme 2, 18.6 mg, 45% yield, 0.045 mmol, an orange solid, eluent: dichloromethane/ethyl acetate (4/1), R_f= 0.2). The ee of (*S*p)-**1f** was determined by its derivative (*S*p)-**12**; $[\alpha]^{25}_{D}$ +3.5×10^2 (c 0.21, $CHCl_3$) for 97% ee. Reported value[4] for (*R*p)-**1f** is $[\alpha]^{25}_{D}$ –5.1×10^2 (c 0.13, $CHCl_3$). **1H NMR** (600 MHz, $CDCl_3$): δ 10.4 (s, 1H), 7.80 (dd, J = 11.9 Hz, 7.4 Hz, 2H), 7.60−7.48 (m, 6H), 7.40 (td, J = 7.7 Hz, 2.8 Hz, 2H), 5.23 (s, 1H), 4.76 (s, 1H), 4.40 (s, 5H), 4.24 (s, 1H); **^{31}P NMR** (243 MHz, $CDCl_3$): δ 27.7. The spectral data are in agreement with reported literature values.[4]

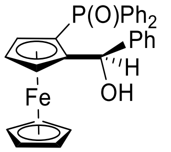

(*R*,*R*p)-**3f**

Compound (*R*,*R*p)-3f. (Scheme 2, 23.1 mg, 47% yield, 0.047 mmol, a yellow solid, eluent: dichloromethane/ethyl acetate (5/1), R_f = 0.4). The ee was measured by HPLC (Chiralpak IA column, hexane/2-propanol = 80/20, 1.0 mL/min, 230 nm, t_{major} = 31.8 min, t_{minor} = 14.7 min); $[\alpha]^{25}_{D}$ −17 (c 0.43, $CHCl_3$) for 94% ee. **^{1}H NMR** (600 MHz, $CDCl_3$): δ 7.82 (dd, J = 12.2 Hz, 7.6 Hz, 2H), 7.60−7.52 (m, 5H), 7.50 (t, J = 7.5 Hz, 1H), 7.42−7.41 (m, 4H), 7.31 (t, J = 7.4 Hz, 2H), 7.25 (t, J = 7.3 Hz, 1H), 6.26 (s, 1H), 5.43 (s, 1H), 4.37 (s, 5H), 4.20 (s, 1H), 3.91 (s, 1H), 3.84 (s, 1H); **^{13}C NMR** (150 MHz, $CDCl_3$): δ 142.1, 134.2, 133.5, 132.7, 132.0 (d, $J_{C\text{-}P}$ = 3.3 Hz), 131.8 (d, $J_{C\text{-}P}$ = 2.7 Hz), 131.7 (d, $J_{C\text{-}P}$ = 9.9 Hz), 131.5 (d, $J_{C\text{-}P}$ = 10.1 Hz), 128.5 (d, $J_{C\text{-}P}$ = 12.1 Hz), 128.3 (d, $J_{C\text{-}P}$ = 12.1 Hz), 127.9, 127.2, 127.0, 101.1 (d, $J_{C\text{-}P}$ = 10.8 Hz), 74.7 (d, $J_{C\text{-}P}$ = 9.4 Hz), 73.3 (d, $J_{C\text{-}P}$ = 15.0 Hz), 70.2, 70.0 (d, $J_{C\text{-}P}$ = 20.5 Hz), 69.1 (d, $J_{C\text{-}P}$ = 11.1 Hz), 68.8; **^{31}P NMR** (243 MHz, $CDCl_3$):

δ 33.2. HRMS (MALDl-TOF) m/z: $[M]^{•+}$ Calcd for $C_{29}H_{25}FeO_2P^{•+}$ 492.0936; Found 492.0932.

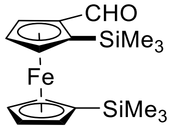

(*S*p)-**1g**

Compound (*S*p)-1g [934276-76-3]. (Scheme 2, 16.8 mg, 47% yield, 0.047 mmol, a red solid, eluent: hexane/ethyl acetate (20/1), R_f = 0.6). The ee was measured by HPLC (Chiralpak ID column, hexane/2-propanol = 98/2, 0.5 mL/min, 230 nm, t_{major} = 11.0 min, t_{minor} = 10.5 min); $[\alpha]^{25}_{D}$ +1.6×10^2 (*c* 0.15, $CHCl_3$) for >99.5% ee. **^{1}H NMR** (600 MHz, $CDCl_3$): δ 10.0 (s, 1H), 4.93 (s, 1H), 4.67 (s, 1H), 4.49 (s, 2H), 4.41 (s, 1H), 4.24 (s, 1H), 4.10 (s, 1H), 0.32 (s, 9H), 0.22 (s, 9H). The spectral data are in agreement with reported literature values.[5]

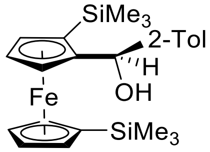

(*R*,*R*p)-**3g**

Compound (*R,R*p)-3g. (Scheme 2, 21.6 mg, 48% yield, 0.048 mmol, a yellow solid, eluent: hexane/ethyl acetate (20/1), R_f = 0.4). The ee was measured by HPLC (Chiralpak ID column, hexane/2-propanol = 95/5, 1.0 mL/min, 230 nm, t_{major} = 5.5 min, t_{minor} = 4.4 min); $[\alpha]^{25}_{D}$ −5.1 (*c* 0.079, $CHCl_3$) for >99.5% ee. **^{1}H NMR** (600 MHz, $CDCl_3$): δ 7.17−7.13 (m, 3H), 7.10 (t, J = 7.3 Hz, 1H), 5.59 (s, 1H), 4.53−4.26 (m, 7H), 2.38 (s, 3H), 2.22 (s, 1H), 0.19 (s, 9H), 0.06 (s, 9H); **^{13}C NMR** (150 MHz, $CDCl_3$): δ 141.1, 136.1, 130.2, 127.6, 127.0, 125.7, 98.6, 75.1, 73.7, 73.2, 71.8, 71.0, 70.7, 70.3, 68.7, 19.1, 0.1, –0.2. HRMS (MALDl-TOF) m/z: $[M]^{•+}$ Calcd for $C_{24}H_{34}FeOSi_2^{•+}$ 450.1492; Found 450.1496.

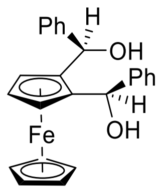

(*R*,*R*)-**6**

Compound (*R*,*R*)-6 [417710-62-4]. (Scheme 3, 185.2 mg, 93% yield, 0.46 mmol, a brown solid, eluent: hexane/ethyl acetate (20/1), $R_f = 0.5$); **^{1}H NMR** (600 MHz, $CDCl_3$): δ 7.39 (d, J = 7.6 Hz, 2H), 7.35−7.28 (m, 6H), 7.27−7.25 (m, 2H), 5.64–5.63 (m, 1H), 5.43 (s, 1H), 4.29 (s, 5H), 4.17 (t, J = 2.0 Hz, 1H), 4.07 (t, J = 2.6 Hz, 1H), 3.80 (t, J = 2.0 Hz, 1H), 3.26 (s, 1H), 3.21 (s, 1H); **HRMS (ESI)** calcd for $C_{24}H_{22}FeO_2Na$ $[M+Na]^+$ 421.0861, found 421.0866. The experimental data are in agreement with the literature report.[9]

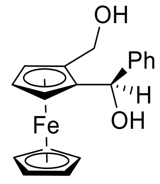

(*R*,*S*p)-**7**

Compound (*R*,*S*p)-7. (Scheme 3, 120.1 mg, 98% yield, 0.37 mmol, an orange solid, eluent: hexane/ethyl acetate (5/1), $R_f = 0.5$); **^{1}H NMR** (600 MHz, $CDCl_3$): δ 7.41 (d, J = 7.1 Hz, 2H), 7.34 (t, J = 7.5 Hz, 2H), 7.28−7.27 (m, 1H), 5.61 (s, 1H), 4.41 (d, J = 12.2 Hz, 1H), 4.35 (d, J = 12.3 Hz, 1H), 4.30 (s, 5+1H), 4.22 (s, 1H), 4.16 (s, 1H), 2.66 (brs., 1H), 1.63 (brs., 1H); **^{13}C NMR** (150 MHz, $CDCl_3$): δ 143.3, 128.3, 127.6, 126.3, 93.0, 85.9, 70.4, 69.5, 68.9, 67.2, 66.5, 59.1; **HRMS (ESI)** calcd for $C_{18}H_{18}FeO_2$ $[M+Na]^+$ 345.0548, found 345.0554.

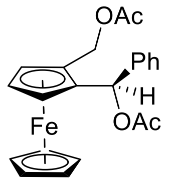

(*R*,*S*p)-**8**

Compound (*R*,*S*p)-8. (Scheme 3, 117.2 mg, 96% yield, 0.29 mmol, an orange solid, eluent: hexane/ethyl acetate (5/1), $R_f = 0.5$); **^{1}H NMR** (600 MHz, $CDCl_3$): δ 7.33−7.27 (m, 5H), 6.88 (s, 1H), 4.99 (d, J = 12.4 Hz, 1H), 4.82 (d, J = 12.1 Hz, 1H), 4.41 (s, 1H), 4.30 (s, 1H), 4.24 (s, 1H), 4.21 (s, 5H), 2.22 (s, 3H), 1.76 (s, 3H); **^{13}C NMR** (150 MHz, $CDCl_3$):

δ 170.5, 169.7, 140.5, 128.2, 128.0, 127.4, 88.7, 79.1, 72.2, 70.5, 69.4, 67.5, 67.4, 61.2, 21.2, 20.5; **HRMS (ESI)** calcd for $C_{22}H_{22}FeO_4$ $[M+Na]^+$ 429.0760, found 429.0762.

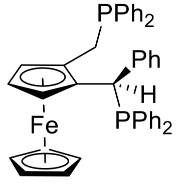

(*R*,*S*p)-**9**

Compound (*R*,*S*p)-9. (Scheme 3, 125.1 mg, 76% yield, 0.19 mmol, a yellow solid, eluent: hexane/ethyl acetate (5/1), R_f = 0.6); **^{1}H NMR** (600 MHz, $CDCl_3$): δ 7.70 (s, 2H), 7.95–6.46 (m, 21H), 6.71 (s, 2H), 4.88 (s, 1H), 4.42 (s, 1H), 4.03 (s, 1H), 3.98 (s, 5H), 3.88 (s, 1H), 3.03 (d, *J* = 15.2 Hz, 1H), 2.79 (d, *J* = 15.4 Hz, 1H); **^{13}C NMR** (150 MHz, $CDCl_3$): δ 141.6, 139.5 (d, $J_{C\text{-}P}$ = 15.1 Hz), 139.2 (d, $J_{C\text{-}P}$ = 15.7 Hz,), 135.5 (d, $J_{C\text{-}P}$ = 22.2 Hz,), 133.3 (d, $J_{C\text{-}P}$ = 20.0 Hz,), 133.2 (d, $J_{C\text{-}P}$ = 19.0 Hz,), 132.1 (d, $J_{C\text{-}P}$ = 18.0 Hz,), 131.2 , 130.3, 128.9 (d, $J_{C\text{-}P}$ = 3.7 Hz), 128.8 (d, $J_{C\text{-}P}$ = 21.9 Hz), 128.5 (d, $J_{C\text{-}P}$ = 5.1 Hz), 128.4 (d, $J_{C\text{-}P}$ = 8.9 Hz), 128.3 (d, $J_{C\text{-}P}$ = 11.7 Hz), 128.1 (d, $J_{C\text{-}P}$ = 24.0 Hz), 127.6 (d, $J_{C\text{-}P}$ = 8.0 Hz), 127.5, 125.7, 124.4, 113.0, 89.9 (dd, $J_{C\text{-}P}$ = 20.6, 3.8 Hz), 83.4 (dd, $J_{C\text{-}P}$ = 14.8, 6.9 Hz), 69.9, 69.2 (d, $J_{C\text{-}P}$ = 9.8 Hz), 65.5, 57.3, 45.6 (d, $J_{C\text{-}P}$ = 21.9 Hz), 28.0 (d, $J_{C\text{-}P}$ = 14.8 Hz); **^{31}P NMR** (243 MHz, $CDCl_3$): δ 1.9, –19.1; **HRMS (ESI)** calcd for $C_{42}H_{37}FeP_2$ $[M+H]^+$ 659.1715, found 659.1711.

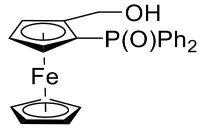

(*S*p)-**10**

Compound (*S*p)-10 [494205-49-1]. (Scheme 3, 79.1 mg, 98% yield, 0.19 mmol, a yellow solid, eluent: dichloromethane/ethyl acetate (4/1), R_f = 0.6). **^{1}H NMR** (600 MHz, $CDCl_3$): δ 7.83−7.80 (m, 2H), 7.60 (td, *J* = 7.4 Hz, 1.5 Hz, 1H), 7.56−7.52 (m, 4H), 7.49−7.46 (m, 1H), 7.41−7.37 (m, 2H), 5.47 (dd, *J* = 10.5 Hz, 2.8 Hz, 1H), 4.54 (s, 1H), 4.34−4.32 (m, 2H), 4.26 (s, 5H), 4.17 (dd, *J* = 13.1 Hz, 10.5 Hz, 1H), 3.93 (s, 1H). **^{31}P NMR** (243 MHz, $CDCl_3$): δ 33.4. The spectral data are in agreement with reported literature values.[10]

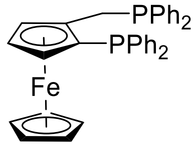

(Sp)-**12a**

Compound (Sp)-12a [395080-13-4]. (Scheme 3, 67.4 mg, 79% yield, 0.12 mmol, a red solid, eluent: hexane/ethyl acetate (20/1), R_f = 0.6). The ee was measured by HPLC (Chiralpak IA column, hexane/2-propanol = 95/5, 1.0 mL/min, 230 nm, t_{major} = 4.8 min (major), t_{minor} = 4.4 min); $[\alpha]^{25}_{D}$ −1.5×10^2 (c 0.12, $CHCl_3$) for 97% ee. Reported value[11] for (Rp)-**12a** is $[\alpha]^{25}_{D}$ +1.8×10^2 (c 0.44, $CHCl_3$). **^{1}H NMR** (600 MHz, $CDCl_3$): δ 7.61−7.58 (m, 2H), 7.45−7.42 (m, 2H), 7.40−7.39 (m, 3H), 7.35−7.34 (m, 3H), 7.32 (td, J = 6.2 Hz, 2.2 Hz, 2H), 7.27−7.25 (m, 6H), 7.20 (td, J = 7.0 Hz, 2.8 Hz, 2H), 4.15 (s, 1H), 4.08 (s, 1H), 3.97 (s, 5H), 3.76 (s, 1H), 3.36 (s, 2H); **^{31}P NMR** (243 MHz, $CDCl_3$): δ −15.0, −23.5. The spectral data are in agreement with reported literature values.[11]

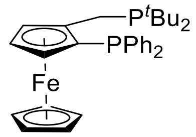

(Sp)-**12b**

Compound (Sp)-12b [950982-86-2]. (Scheme 3, 50.2 mg, 63% yield, 0.095 mmol, a yellow solid, eluent: hexane/ethyl acetate (20/1), R_f= 0.4); $[\alpha]^{25}_{D}$ −1.6×10^2 (c 0.12, $CHCl_3$). Reported value[11] for (Sp)-**12b** is $[\alpha]^{20}_{D}$ −1.6×10^2 (c 1.0, $CHCl_3$). **^{1}H NMR** (600 MHz, $CDCl_3$): δ 7.61−7.58 (m, 2H), 7.38−7.37 (m, 3H), 7.25−7.23 (m, 5H), 4.76 (s, 1H), 4.21(s, 1H), 3.99 (s, 5H), 3.77 (s, 1H), 2.81 (d, J = 16.4 Hz, 1H), 2.62 (dd, J = 16.4 Hz, 5.0 Hz, 1H), 1.19 (d, J = 11.0 Hz, 9H), 0.89 (d, J = 10.9 Hz, 9H); **^{31}P NMR** (243 MHz, $CDCl_3$): δ 26.9, −23.6. The spectral data are in agreement with reported literature values.[11]

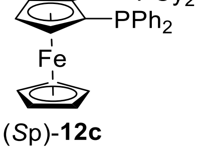

(Sp)-**12c**

Compound (Sp)-12c [395080-11-2]. (Scheme 3, 58.3 mg, 67% yield, 0.10 mmol, a yellow solid, eluent: hexane/ethyl acetate (20/1), R_f= 0.4); $[\alpha]^{25}_{D}$ −1.5×10^2 (c 0.12, $CHCl_3$). Reported value[11] for (Sp)-**12c** is $[\alpha]^{20}_{D}$ −1.6×10^2 (c 1.0, $CHCl_3$). **^{1}H NMR** (600 MHz,

CDCl3): δ 7.59−7.56 (m, 2H), 7.38−7.37 (m, 3H), 7.23−7.18 (m, 5H), 4.55 (s, 1H), 4.22 (s, 1H), 3.97 (s, 5H), 3.74 (s, 1H), 2.71 (d, J = 15.4 Hz, 1H), 2.63 (d, J = 15.6 Hz, 1H), 1.81−1.79 (m, 4H), 1.71 (s, 1H), 1.63−1.56 (m, 3H), 1.51−1.47 (m, 2H), 1.35−1.22 (m, 7H), 1.07−1.02 (m, 5H); **^{31}P NMR** (243 MHz, CDCl3): δ –2.1, –23.6. The spectral data are in agreement with reported literature values.[11]

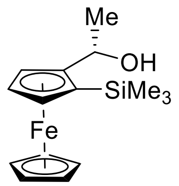

(*S*,*S*p)-**13**

Compound (*S*,*S*p)-13. (Scheme 3, 53.8 mg, 89% yield, 0.18 mmol, a yellow solid, eluent: hexane/ethyl acetate (20/1), R_f = 0.4); $[\alpha]^{25}_{D}$ +38 (c 0.080, CHCl3). Reported value[12] for (*R*,*R*p)-**13** is $[\alpha]^{20}_{D}$ –45 (c 0.83, CHCl3). **^{1}H NMR** (600 MHz, CDCl3): δ 4.54−4.51 (m, 2H), 4.38 (s, 1H), 4.27 (s, 5H), 4.21 (s, 1H), 1.96 (s, 1H), 1.34 (d, J = 6.1 Hz, 3H), 0.23 (s, 9H). The spectral data are in agreement with reported literature values.[12]

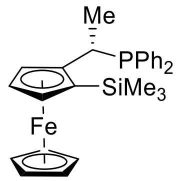

(*S*,*S*p)-**15**

Compound (*S*,*S*p)-15. (Scheme 3, 28.2 mg, 60% yield, 0.060 mmol, an orange solid, eluent: hexane/ethyl acetate (20/1), R_f = 0.4); $[\alpha]^{25}_{D}$ +1.3× 10^2 (c 0.093 CHCl3). **^{1}H NMR** (600 MHz, CDCl3): δ 7.65−7.62 (m, 2H), 7.51 (t, J = 7.2 Hz, 2H), 7.40−7.38 (m, 5H), 7.33 (dd, J = 7.5 Hz, 7.2 Hz, 1H), 4.24 (s, 1H), 4.21 (s, 1H), 4.12 (s, 5H), 4.07 (s, 1H), 3.42 (qd, J = 7.8 Hz, 3.1 Hz, 1H), 1.19 (dd, J = 7.7 Hz, 7.6 Hz, 3H), 0.38 (s, 9H); **^{31}P NMR** (243 MHz, CDCl3): δ 0.2. The spectral data are in agreement with reported literature values.[13]

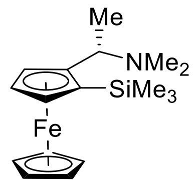

(*S*,*S*p)-**16**

Compound (*S*,*S*p)-16 [62960-90-1]. (Scheme 3, 59.2 mg, 90% yield, 0.18 mmol, an orange oil, eluent: hexane/ethyl acetate/triethylamine (20/1/1%), R_f = 0.4); $[\alpha]^{25}_{D}$ −67 (*c* 0.042, $CHCl_3$). Reported value[12] for (*R*,*R*p)-**16** is $[\alpha]^{20}_{D}$ +46 (*c* 1.32, $CHCl_3$). **^{1}H NMR** (600 MHz, $CDCl_3$): δ 4.33 (s, 1H), 4.26 (t, *J* = 2.5 Hz, 1H), 4.12 (s, 5H), 4.02 (s, 1H), 3.33 (q, *J* = 6.9 Hz, 1H), 2.36 (s, 6H), 1.18 (d, *J* = 6.9 Hz, 3H), 0.30 (s, 9H). The spectral data are in agreement with reported literature values.[12]

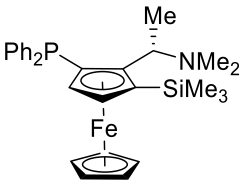

(*S*,*R*p)-**17**

Compound (*S*,*R*p)-17. (Scheme 3, 65.4 mg, 85% yield, 0.13 mmol, a red solid, eluent: hexane/ethyl acetate/triethylamine (10/1/1%), R_f = 0.4); $[\alpha]^{25}_{D}$ +1.6×10^2 (*c* 0.12, $CHCl_3$). **^{1}H NMR** (600 MHz, $CDCl_3$): δ 7.59−7.56 (m, 2H), 7.39−7.37 (m, 3H), 7.24−7.22 (m, 5H), 4.20 (d, *J* = 2.4 Hz, 1H), 3.96 (s, 5H), 3.93 (d, *J* = 2.4 Hz, 1H), 3.17 (qd, *J* = 7.1 Hz, 3.1 Hz, 1H), 1.82 (s, 6H), 1.63 (d, *J* = 7.0 Hz, 3H), 0.29 (s, 9H); **^{13}C NMR** (150 MHz, $CDCl_3$): δ 139.8 (d, $J_{C\text{-}P}$ = 9.1 Hz), 138.5 (d, $J_{C\text{-}P}$ = 10.2 Hz), 135.3 (d, $J_{C\text{-}P}$ = 21.7 Hz), 132.7 (d, $J_{C\text{-}P}$ = 18.6 Hz), 128.9, 128.0 (d, $J_{C\text{-}P}$ = 7.7 Hz), 127.7 (d, $J_{C\text{-}P}$ = 6.4 Hz), 127.7, 107.4 (d, $J_{C\text{-}P}$ = 20.2 Hz), 78.1, 75.4, 73.5 (d, $J_{C\text{-}P}$ =2.7 Hz), 72.4 (d, $J_{C\text{-}P}$ = 4.4 Hz), 69.8, 59.5 (d, $J_{C\text{-}P}$ = 10.1 Hz), 43.6, 22.6, 1.6; **^{31}P NMR** (243 MHz, $CDCl_3$): δ −23.8; HRMS (MALDl-TOF) m/z: $[M]^{\bullet+}$ Calcd for $C_{29}H_{36}FeNPSi^{\bullet+}$ 513.1699; Found 513.1704.

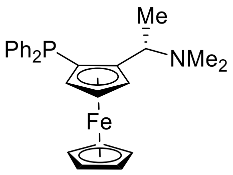

(*S*,*R*p)-**PPFA**

Compound (*S*,*R*p)-PPFA [55650-58-3]. (Scheme 3, 38.4 mg, 87% yield, 0.087 mmol, a red solid, eluent: hexane/ethyl acetate (20/1), R_f = 0.4); $[\alpha]^{25}_{D}$ +3.5×10^2 (*c* 0.12, $CHCl_3$). Reported value[14] for (*R*,*S*p)-**PPFA** is $[\alpha]^{25}_{D}$ −3.5×10^2 (*c* 0.25, $CHCl_3$). **^{1}H NMR** (600 MHz, $CDCl_3$): δ 7.61−7.58 (m, 2H), 7.36−7.35 (m, 3H), 7.22−7.15 (m, 5H), 4.37 (s, 1H), 4.25 (s, 1H), 4.16 (qd, *J* = 6.6 Hz, 2.7 Hz, 1H), 3.94 (s, 5H), 3.86 (s, 1H), 1.78 (s, 6H), 1.26 (d, *J* = 6.7 Hz, 3H); **^{31}P NMR** (243 MHz, $CDCl_3$): δ –22.8. The spectral data are in agreement with reported literature values.[14]

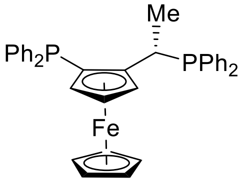

(*S*,*R*p)-**18**

Compound (*S*,*R*p)-18 [155941-31-4]. (Scheme 3, 18.3 mg, 0.031 mmol, 63% yield, a yellow solid, eluent: hexane/ethyl acetate (20/1), R_f = 0.4; $[\alpha]^{25}_{D}$ +3.2×10^2 (*c* 0.12, $CHCl_3$). Reported value[15] for (*S*,*R*p)-**18** is $[\alpha]^{22}_{D}$ +3.6×10^2 (*c* 1.0, $CHCl_3$). **^{1}H NMR** (600 MHz, $CDCl_3$): δ 7.68−7.65 (m, 2H), 7.40−7.38 (m, 3H), 7.34−7.30 (m, 3H), 7.29−7.26 (m, 4H), 7.25−7.22 (m, 2H), 7.22−7.17 (m, 6H), 4.24 (t, *J* = 2.6 Hz, 1H), 4.05 (s, 1H), 4.01 (s, 1H), 3.86 (s, 5H), 3.77 (qd, *J* =7.0 Hz, 3.7 Hz, 1H), 1.46 (dd, *J* = 7.3 Hz, 7.3 Hz, 3H); **^{31}P NMR** (243 MHz, $CDCl_3$): δ 6.34 (d, *J* = 20.5 Hz), –25.5 (d, *J* = 20.5 Hz). The spectral data are in agreement with reported literature values.[15]

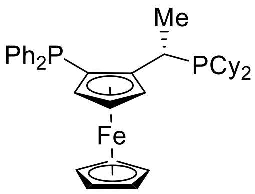

(*S*,*R*p)-**19**

Compound (*S*,*R*p)-19 [162291-02-3]. (Scheme 3, 22.6 mg, 75% yield, 0.038 mmol, a red solid, eluent: hexane/ethyl acetate (20/1), R_f = 0.4); $[\alpha]^{25}_{D}$ +2.9×10^2 (*c* 0.12, $CHCl_3$).

Reported value[15] for (R,Sp)-**19** is $[\alpha]^{25}_{D}$ −3.5×10^2 (c 0.60, $CHCl_3$). **^{1}H NMR** (600 MHz, $CDCl_3$): δ 7.70−7.67 (m, 2H), 7.38 (t, J = 7.0 Hz, 2H), 7.08−7.05 (m, 5H), 6.99−6.98 (m, 1H), 4.21 (s, 1H), 4.12 (s, 1H), 4.08 (s, 1H), 3.78 (s, 5H), 3.51 (qd, J = 7.0 Hz, 3.1 Hz, 1H), 1.83 (d, J = 12.9 Hz, 1H), 1.76 (d, J = 10.9 Hz, 2H), 1.70−1.67 (m, 3H), 1.65−1.63 (m, 3H), 1.61−1.58 (m, 5H), 1.41−1.35 (m, 1H), 1.26−1.11 (m, 10H); **^{31}P NMR** (243 MHz, $CDCl_3$): δ 14.8 (d, J = 36.7 Hz), –25.8 (d, J = 36.5 Hz). The spectral data are in agreement with reported literature values.[15]

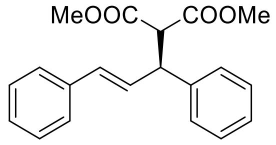

(S)-**23**

Compound (*S*)-23[96482-64-3]. (Scheme 3, 28.2 mg, 87% yield, 0.087 mmol, a colorless solid, eluent: hexane/ethyl acetate (10/1), R_f = 0.4). The ee was measured by HPLC (Chiralpak IA column, hexane/2-propanol = 90/10, 1.0 mL/min, 230 nm, t_{major} = 10.3 min (major), t_{minor} = 8.4 min); $[\alpha]^{25}_{D}$ −14 (c 0.27, $CHCl_3$) for 90% ee. Reported value[6] for 93% ee (R)-**23** is $[\alpha]^{22}_{D}$ +4.7 (c 1.8, CH_2Cl_2). **^{1}H NMR** (600 MHz, $CDCl_3$): δ 7.35−7.32 (m, 6H), 7.29 (t, J = 7.3 Hz, 2H), 7.26−7.25 (m, 1H), 7.22 (t, J = 7.4 Hz, 1H), 6.52 (d, J = 15.7 Hz, 1H), 6.38 (dd, J = 15.8 Hz, 8.6 Hz, 1H), 4.31 (dd, J = 9.9 Hz, 9.7 Hz, 1H), 4.00 (d, J = 10.9 Hz, 1H), 3.72 (s, 3H), 3.54 (s, 3H); **^{13}C NMR** (150 MHz, $CDCl_3$): δ 168.2, 167.8, 140.3, 136.9, 131.9, 129.2, 128.8, 128.5, 127.9, 127.6, 127.2, 126.4, 57.7, 52.6, 52.4, 49.2. The spectral data are in agreement with reported literature values.[6]

9. Single crystal x-ray diffraction date for compound 3ah(CCDC 2403094)

Suitable crystals of compound **3ah** were obtained by slowly evaporating a mixture of dichloromethane and hexane solution at ambient temperature. red crystal of **3ah** was mounted on a glass fiber at a random orientation. The data were collected by Rigaku Oxford Diffraction Supernova Dual Source, Cu at Zero equipped with an AtlasS2 CCD using CuK_{α} radiation (1.54178 Å) by using a w scan mode. The structures were solved by direct methods using Olex2 software, and the nonhydrogen atoms were located from the trial structure and then refined anisotropically with XL using a full-matrix least squares procedure based on F^2. The weighted R factor, wR and goodness-of-fit S values were obtained based on F^2. The hydrogen atom positions were fixed geometrically at the calculated distances and allowed to ride on the parent atoms.

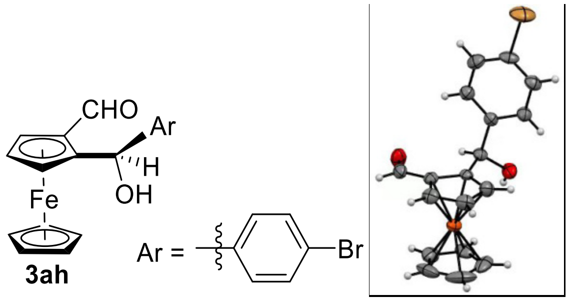

Figure S1. ORTEP illustration of compound **3ha** with thermal ellipsoids drawn at 50% probability level.

Table S5 Crystal data and structure refinement for 3ah.

Identification code	Febr
Empirical formula	$C_{18}H_{15}BrFeO_2$
Formula weight	399.06
Temperature/K	219.99(10)
Crystal system	triclinic
Space group	P1
a/Å	7.6130(5)
b/Å	8.5829(4)
c/Å	12.8193(7)
α/°	80.360(4)
β/°	73.628(5)
γ/°	82.082(5)

Volume/Å^3	788.68(8)
Z	2
ρ_{calc}g/cm^3	1.680
μ/mm^{-1}	10.660
F(000)	400.0
Crystal size/mm^3	0.16 × 0.12 × 0.11
Radiation	Cu Kα (λ = 1.54184)
2Θ range for data collection/°	7.254 to 148.02
Index ranges	$-9 \le h \le 9$, $-10 \le k \le 10$, $-15 \le l \le 15$
Reflections collected	12506
Independent reflections	5856 [R_{int} = 0.0551, R_{sigma} = 0.0644]
Data/restraints/parameters	5856/3/399
Goodness-of-fit on F^2	1.080
Final R indexes [I>=2σ (I)]	R_1 = 0.0445, wR_2 = 0.1133
Final R indexes [all data]	R_1 = 0.0507, wR_2 = 0.1195
Largest diff. peak/hole / e Å^{-3}	0.40/-0.52
Flack/Hooft parameter	-0.006(5)/-0.004(4)

10. References

1. A. van der Ent and A. L. Onderdelinden, *Inorg. Synth.*, 1990, **28**, 90–92.
2. T. Nishimura, H. Kumamoto, M. Nagaosa and T. Hayashi, *Chem. Commun.*, 2009, 5713–5715.
3. A. Sadeer, T. Kojima, J. S. Ng, K. Gan, R. J. Chew, Y. Li and S. A. Pullarkat, *Tetrahedron*, 2020, **76**, 131259.
4. S.-i. Fukuzawa, I. Oura, K. Shimizu, M. Kato and K.-i. Ogata, *Eur. J. Org. Chem.*, 2009, **2009**, 716–720.
5. H. Li, H.-S. Cheng, A.-H. Seow and T.-P. Loh, *Tetrahedron. Lett.*, 2007, **48**, 2209–2211.
6. C.-X. Liu, F. Zhao, Q. Gu and S.-L. You, *ACS Cent. Sci.*, 2023, **9**, 2036–2043.
7. Y. Yang, N.-N. Hang, H.-Y. Liu, S. Chen and J. Ming, *ACS Catal.*, 2023, **13**, 12464–12471.
8. O. Riant, O. Samuel, T. Flessner, S. Taudien and H. B. Kagan, *J. Org. Chem.*, 1997, **62**, 6733−6745.
9. S.-i. Fukuzawa, K. Fujimoto, Y. Komuro and H. Matsuzawa, *Org. Lett.*, 2002, **4**, 707–709.
10. P. Štěpnička and I. Císařová, *New J. Chem.*, 2002, **26**, 1389–1396.
11. G. Argouarch, O. Samuel and H. B. Kagan, *Eur. J. Org. Chem.*, 2000, **2000**, 2885–2891.
12. V. Bariak, A. Malastová, A. Almássy and R. Šebesta, *Chem. Eur. J.*, 2015, **21**,13445–13453.
13. M. Murakami, R. Minamida, K. Itami, M. Sawamura and Y. Ito, *Chem. Commun.*, 2000, 2293–2294.
14. H. Nie, L. Yao, B. Li, S. Zhang and W. Chen, *Organometallics*, 2014, **33**, 2109−2114.
15. A. Togni, C. Breutel, A. Schnyder, F. Spindler, H. Landert and A. Tijani, *J. Am. Chem. Soc.*, 1994, **116**, 4062−4066.

11. Chiral HPLC charts and NMR spectra

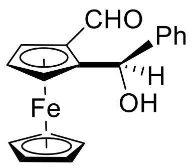

(R,Rp)-**3aa**

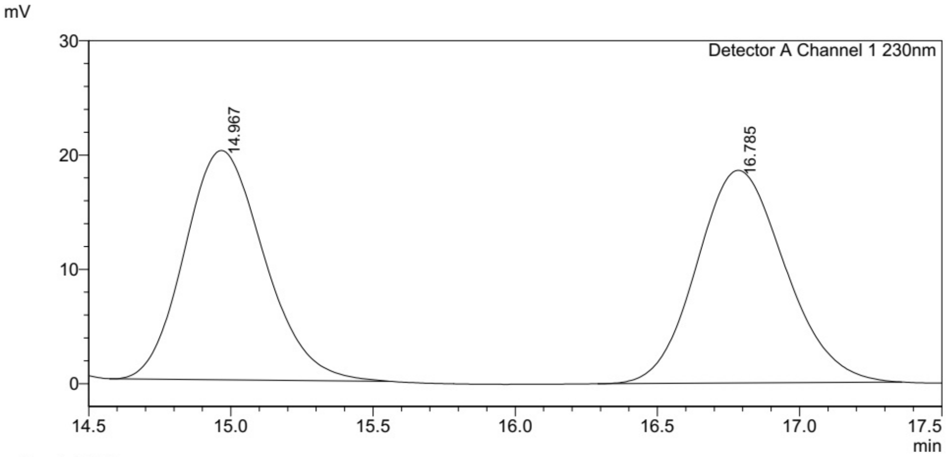

<Peak Table>

Detector A Channel 1 230nm

Peak#	Ret. Time	Area	Height	Area%
1	14.967	391856	20070	49.277
2	16.785	403357	18611	50.723
Total		795213	38682	100.000

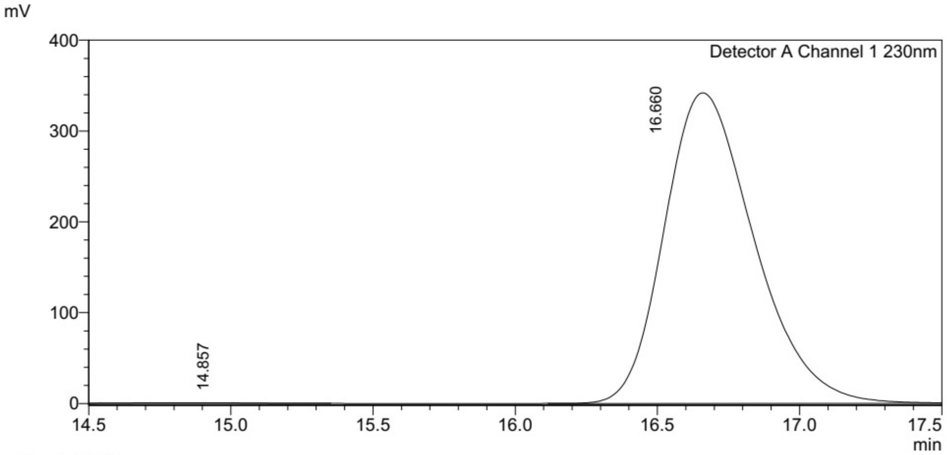

<Peak Table>

Detector A Channel 1 230nm

Peak#	Ret. Time	Area	Height	Area%
1	14.857	20801	733	0.276
2	16.660	7525188	341934	99.724
Total		7545990	342666	100.000

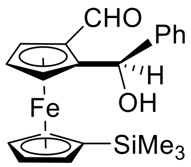

(R,Rp)-**3ba**

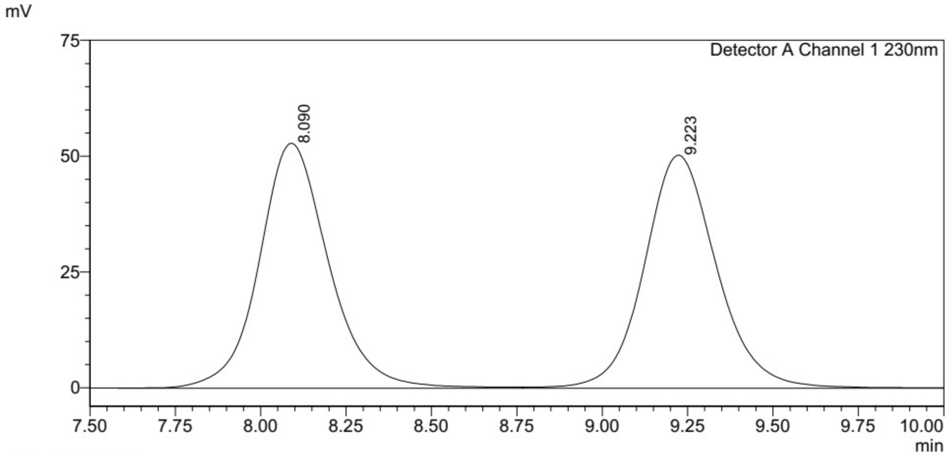

<Peak Table>

Detector A Channel 1 230nm

Peak#	Ret. Time	Area	Height	Area%
1	8.090	750972	52881	50.200
2	9.223	744990	50296	49.800
Total		1495962	103177	100.000

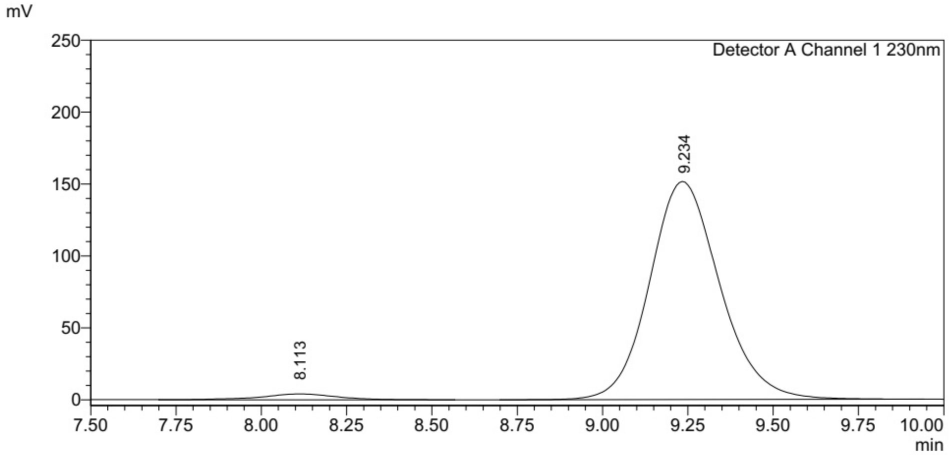

<Peak Table>

Detector A Channel 1 230nm

Peak#	Ret. Time	Area	Height	Area%
1	8.113	67035	4148	2.989
2	9.234	2176047	151591	97.011
Total		2243082	155739	100.000

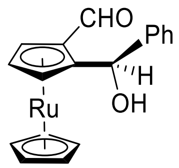

(R,Rp)-**3ca**

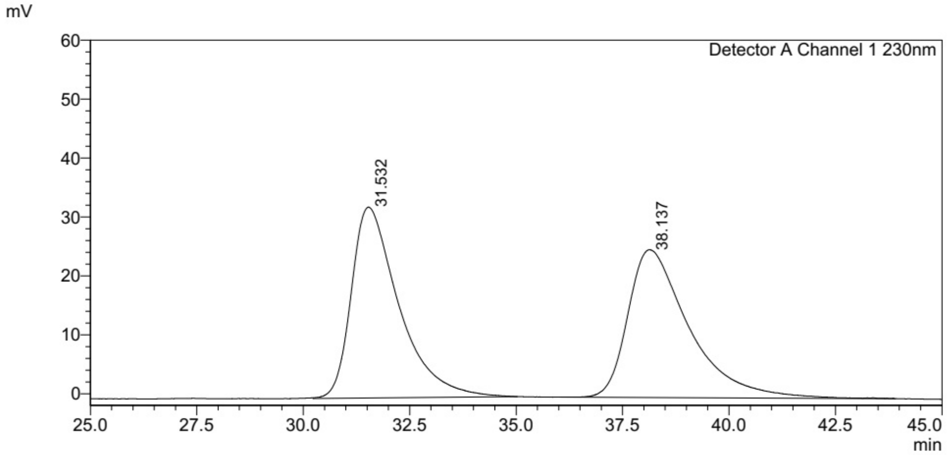

<Peak Table>

Detector A Channel 1 230nm

Peak#	Ret. Time	Area	Height	Area%
1	31.532	2518845	32405	50.122
2	38.137	2506605	25087	49.878
Total		5025450	57491	100.000

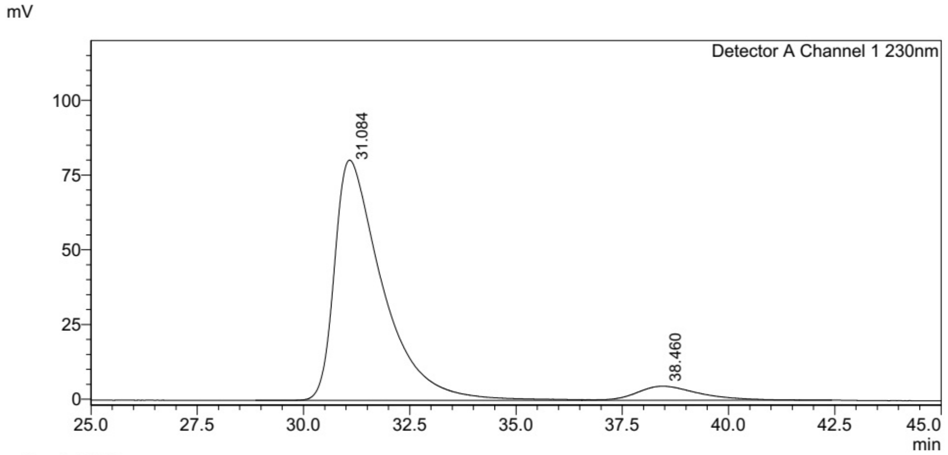

<Peak Table>

Detector A Channel 1 230nm

Peak#	Ret. Time	Area	Height	Area%
1	31.084	6324957	80398	92.930
2	38.460	481174	4707	7.070
Total		6806131	85104	100.000

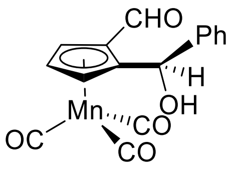

(R,Rp)-**3da**

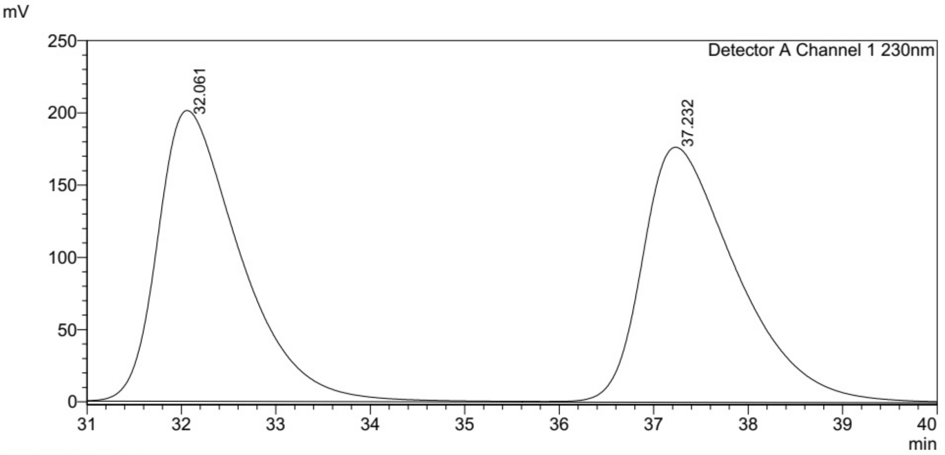

<Peak Table>

Detector A Channel 1 230nm

Peak#	Ret. Time	Area	Height	Area%
1	32.061	12259335	201335	49.919
2	37.232	12298933	176577	50.081
Total		24558267	377911	100.000

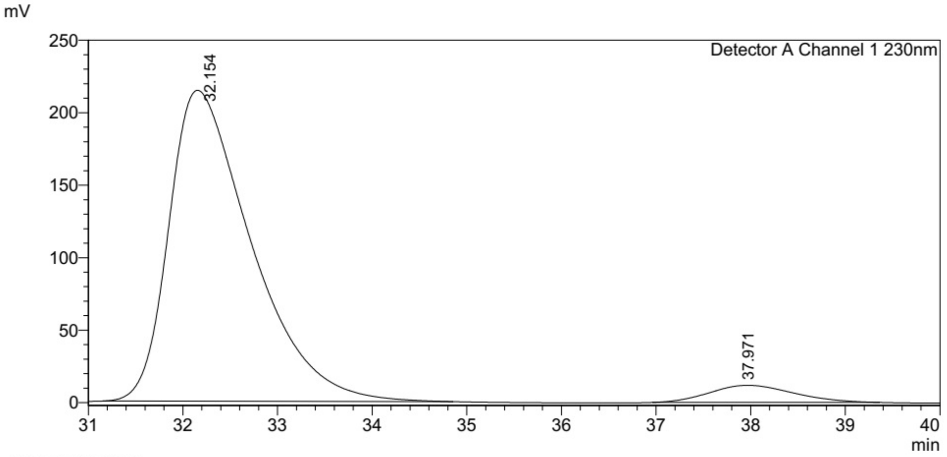

<Peak Table>

Detector A Channel 1 230nm

Peak#	Ret. Time	Area	Height	Area%
1	32.154	12977772	214474	94.675
2	37.971	729925	11804	5.325
Total		13707697	226278	100.000

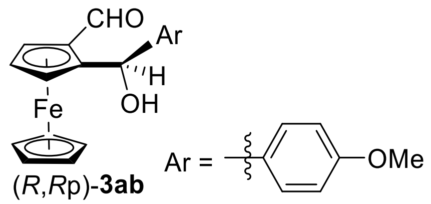

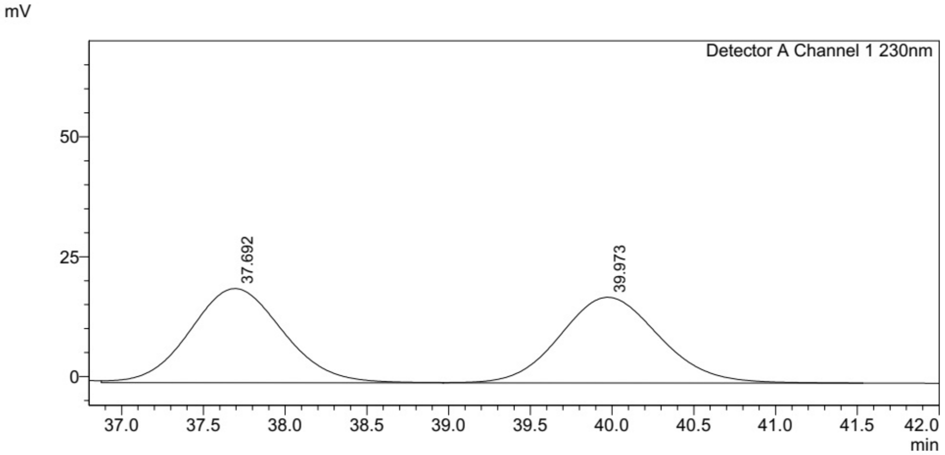

<Peak Table>

Detector A Channel 1 230nm

Peak#	Ret. Time	Area	Height	Area%
1	37.692	782422	19629	50.798
2	39.973	757852	17849	49.202
Total		1540274	37478	100.000

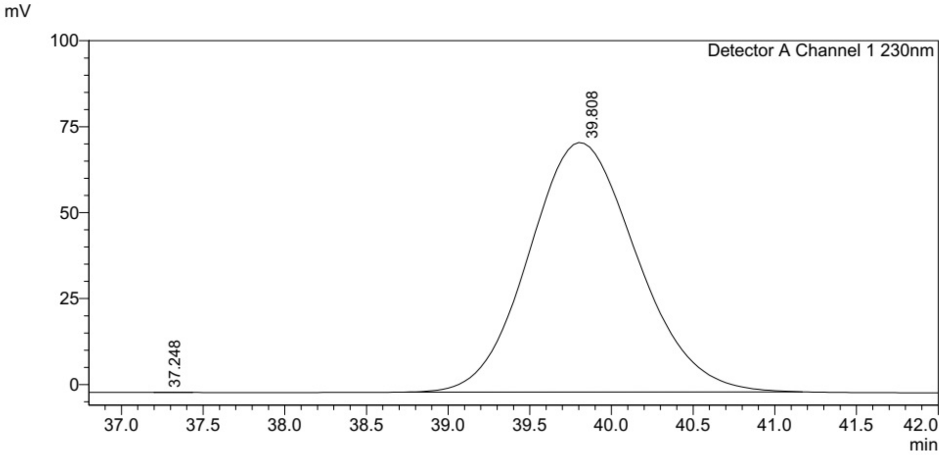

<Peak Table>

Detector A Channel 1 230nm

Peak#	Ret. Time	Area	Height	Area%
1	37.248	39	4	0.001
2	39.808	3341730	72569	99.999
Total		3341769	72573	100.000

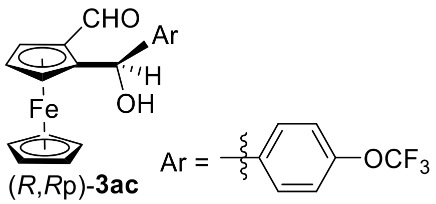

(*R*,*R*p)-**3ac**

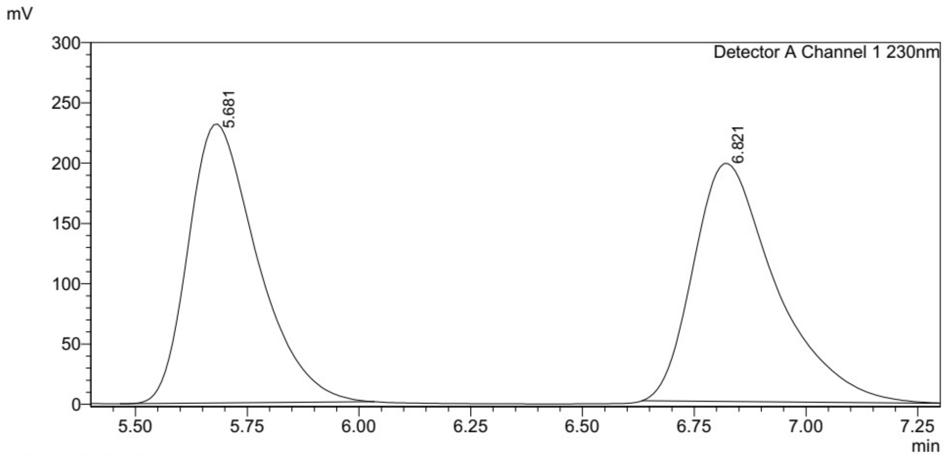

<Peak Table>

Detector A Channel 1 230nm

Peak#	Ret. Time	Area	Height	Area%
1	5.681	2489199	231354	49.471
2	6.821	2542481	197356	50.529
Total		5031680	428710	100.000

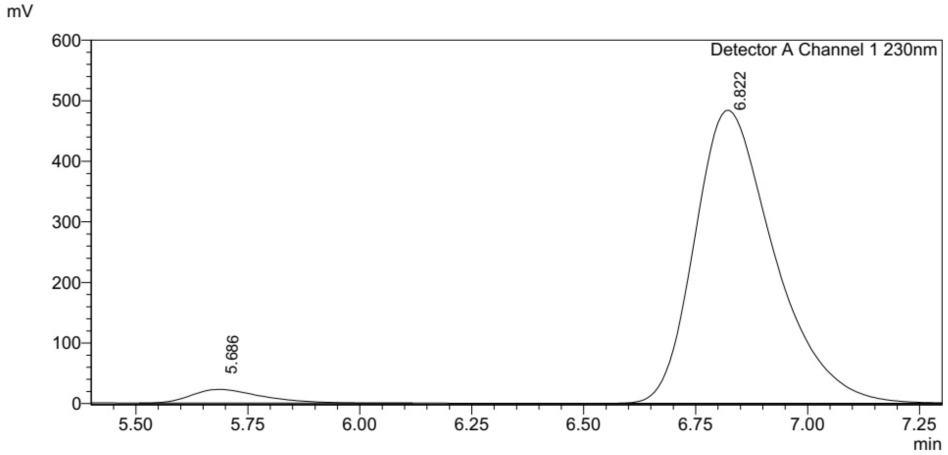

<Peak Table>

Detector A Channel 1 230nm

Peak#	Ret. Time	Area	Height	Area%
1	5.686	243481	22662	3.906
2	6.822	5989673	483793	96.094
Total		6233154	506455	100.000

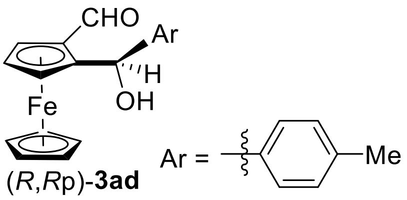

(*R*,*R*p)-**3ad**

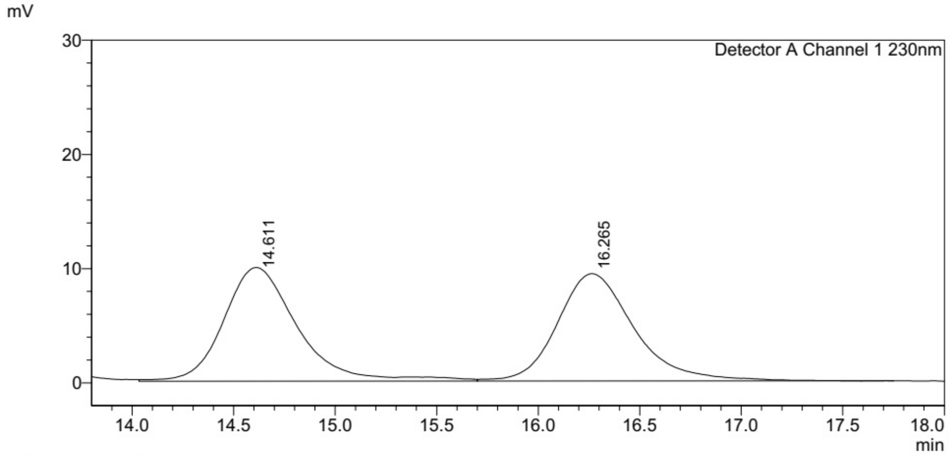

<Peak Table>

Detector A Channel 1 230nm

Peak#	Ret. Time	Area	Height	Area%
1	14.611	253109	9959	50.583
2	16.265	247279	9396	49.417
Total		500389	19355	100.000

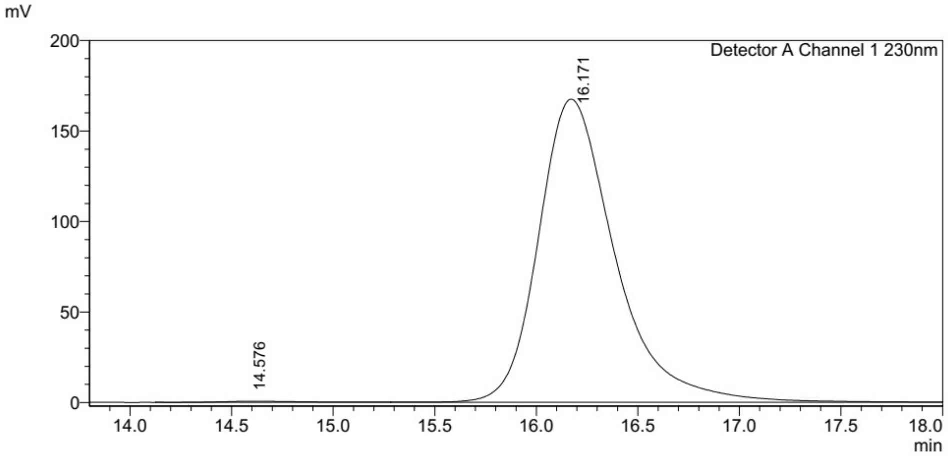

<Peak Table>

Detector A Channel 1 230nm

Peak#	Ret. Time	Area	Height	Area%
1	14.576	18565	600	0.418
2	16.171	4418180	167531	99.582
Total		4436745	168130	100.000

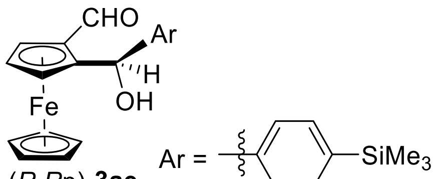

(*R*,*R*p)-**3ae**

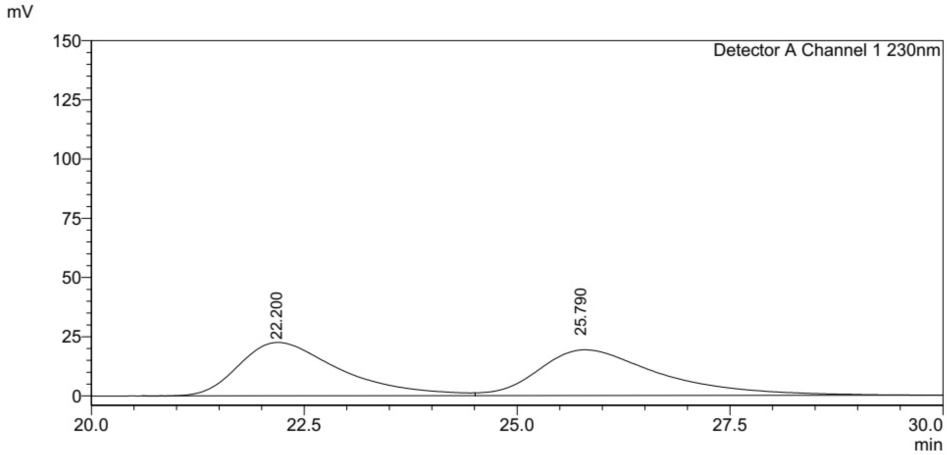

<Peak Table>

Detector A Channel 1 230nm

Peak#	Ret. Time	Area	Height	Area%
1	22.200	1870339	22549	49.384
2	25.790	1917026	19312	50.616
Total		3787365	41860	100.000

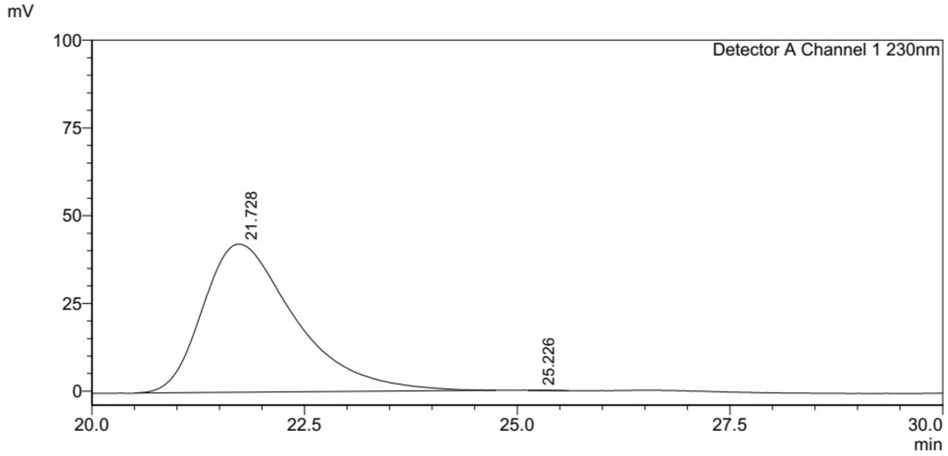

<Peak Table>

Detector A Channel 1 230nm

Peak#	Ret. Time	Area	Height	Area%
1	21.728	3190991	42209	99.969
2	25.226	986	50	0.031
Total		3191977	42259	100.000

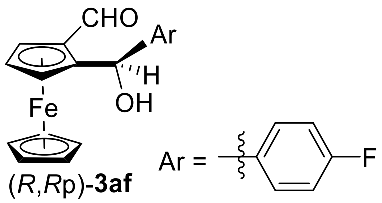

(*R*,*R*p)-**3af**

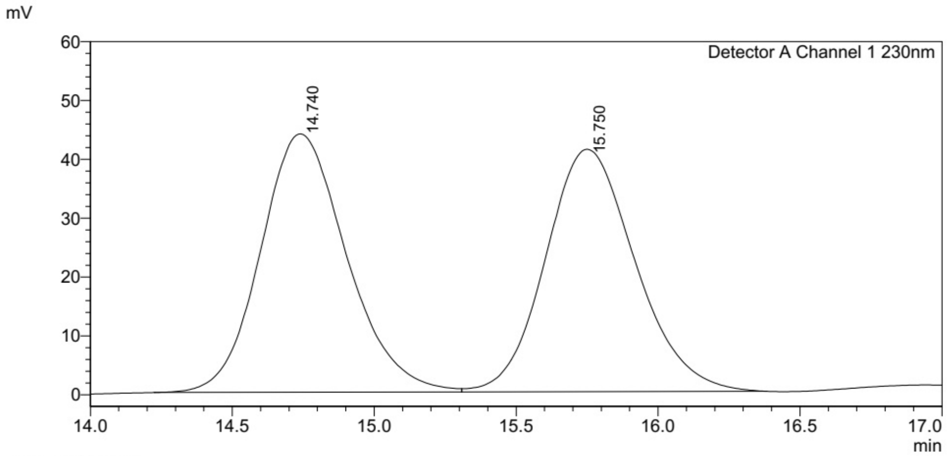

<Peak Table>

Detector A Channel 1 230nm

Peak#	Ret. Time	Area	Height	Area%
1	14.740	926682	43913	50.549
2	15.750	906560	41212	49.451
Total		1833242	85125	100.000

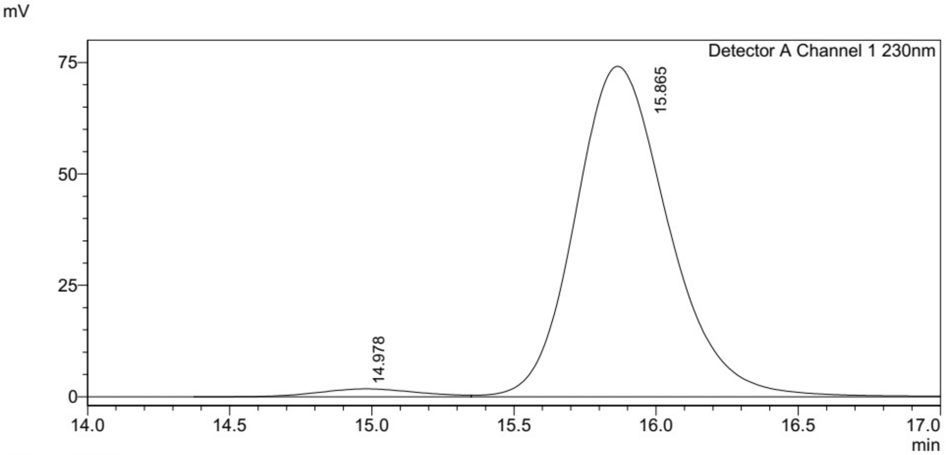

<Peak Table>

Detector A Channel 1 230nm

Peak#	Ret. Time	Area	Height	Area%
1	14.978	43369	1782	2.493
2	15.865	1696011	74190	97.507
Total		1739380	75973	100.000

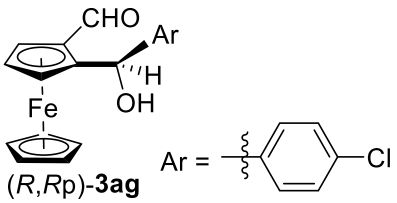

(*R*,*R*p)-**3ag**

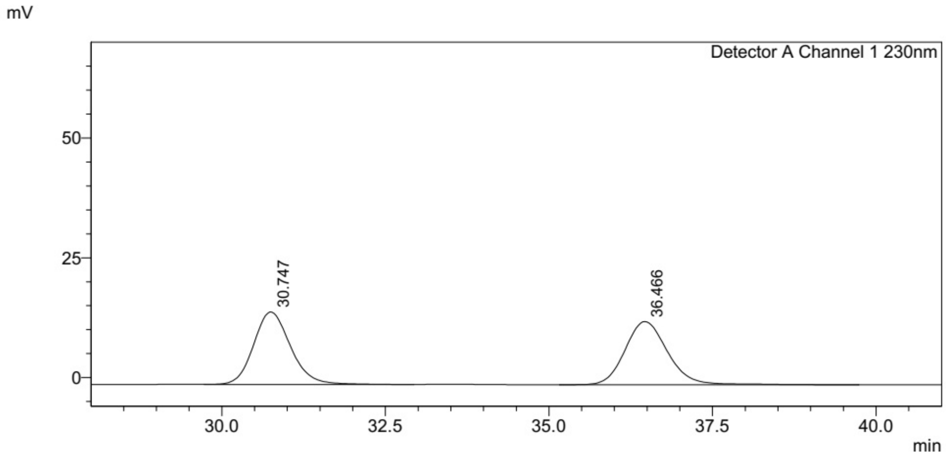

<Peak Table>

Detector A Channel 1 230nm

Peak#	Ret. Time	Area	Height	Area%
1	30.747	602108	15149	50.137
2	36.466	598829	13201	49.863
Total		1200937	28350	100.000

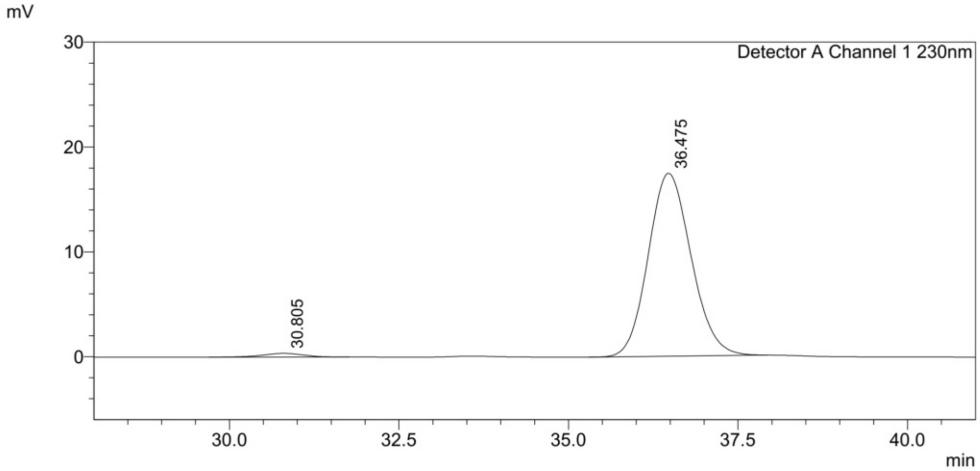

<Peak Table>

Detector A Channel 1 230nm

Peak#	Ret. Time	Area	Height	Area%
1	30.805	15156	358	1.933
2	36.475	768728	17446	98.067
Total		783884	17804	100.000

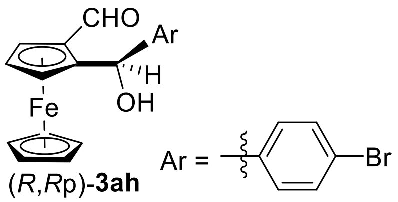

(*R*,*R*p)-**3ah**

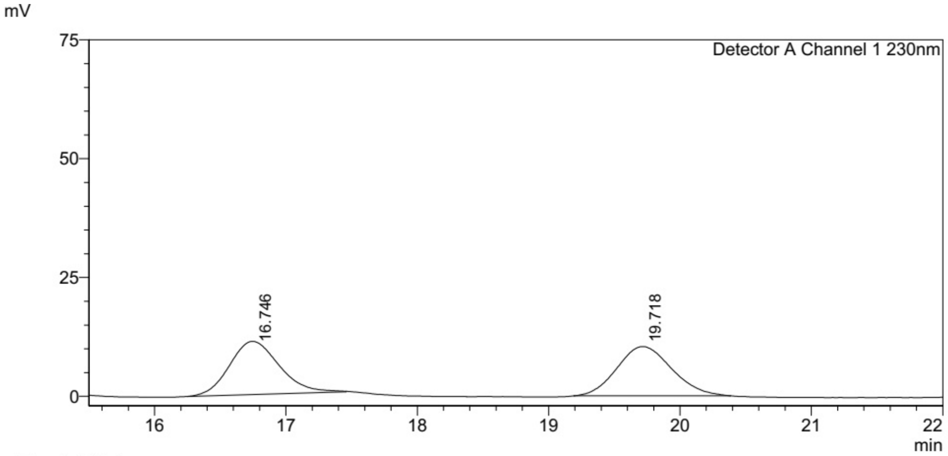

<Peak Table>

Detector A Channel 1 230nm

Peak#	Ret. Time	Area	Height	Area%
1	16.746	294873	11175	49.383
2	19.718	302239	10377	50.617
Total		597112	21552	100.000

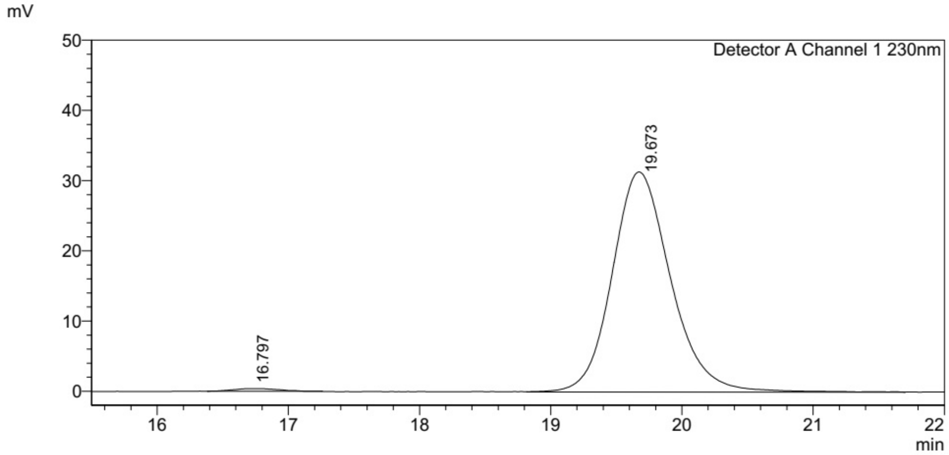

<Peak Table>

Detector A Channel 1 230nm

Peak#	Ret. Time	Area	Height	Area%
1	16.797	9313	385	0.948
2	19.673	972982	31363	99.052
Total		982296	31748	100.000

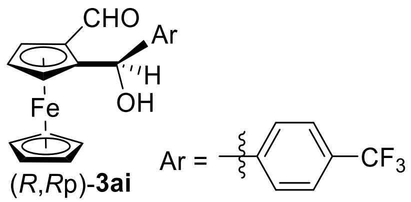

(R,Rp)-**3ai**

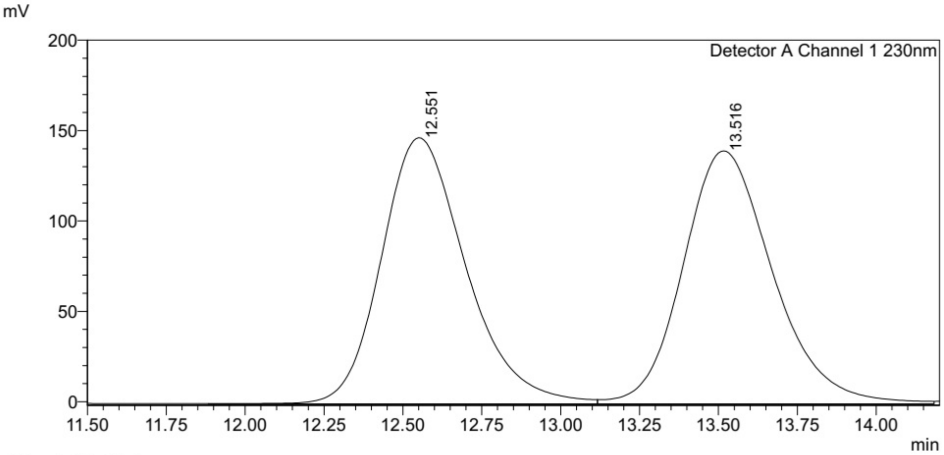

<Peak Table>

Detector A Channel 1 230nm

Peak#	Ret. Time	Area	Height	Area%
1	12.551	2749607	147253	49.823
2	13.516	2769191	140027	50.177
Total		5518798	287281	100.000

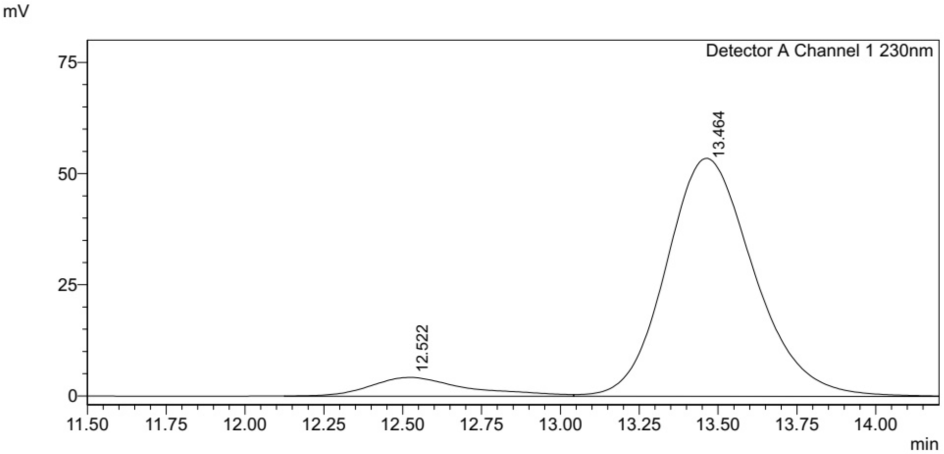

<Peak Table>

DDetector A Channel 1 230nm

Peak#	Ret. Time	Area	Height	Area%
1	12.522	91727	4247	8.112
2	13.464	1038969	53530	91.888
Total		1130696	57777	100.000

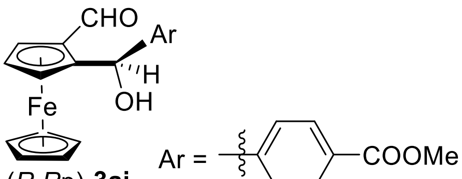

(R,Rp)-**3aj**

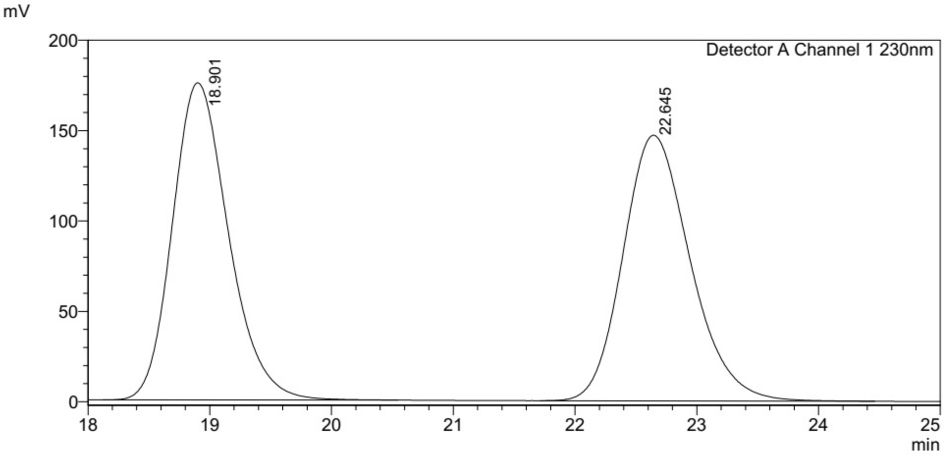

<Peak Table>

Detector A Channel 1 230nm

Peak#	Ret. Time	Area	Height	Area%
1	18.901	5635354	175491	49.943
2	22.645	5648279	146967	50.057
Total		11283633	322458	100.000

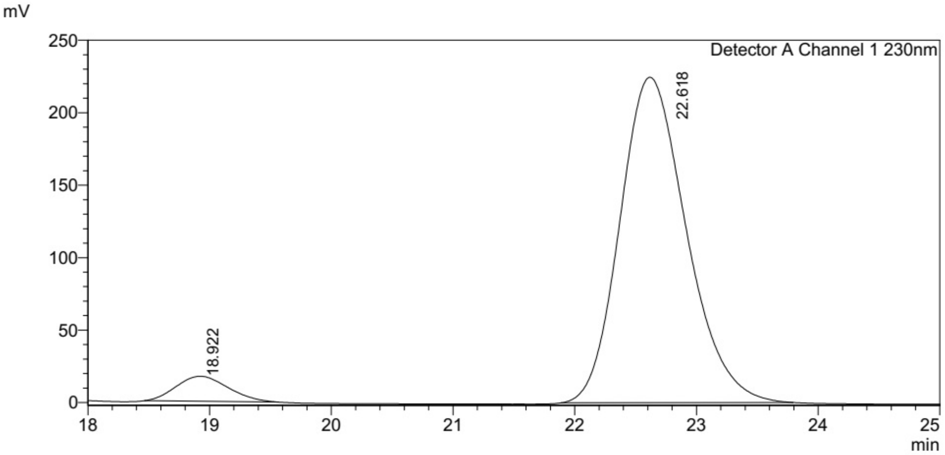

<Peak Table>

Detector A Channel 1 230nm

Peak#	Ret. Time	Area	Height	Area%
1	18.922	513080	17264	5.686
2	22.618	8510440	224560	94.314
Total		9023520	241824	100.000

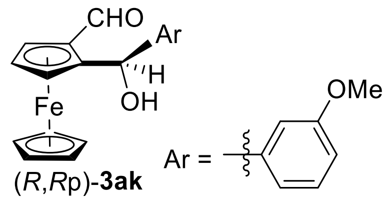

(*R*,*R*p)-**3ak**

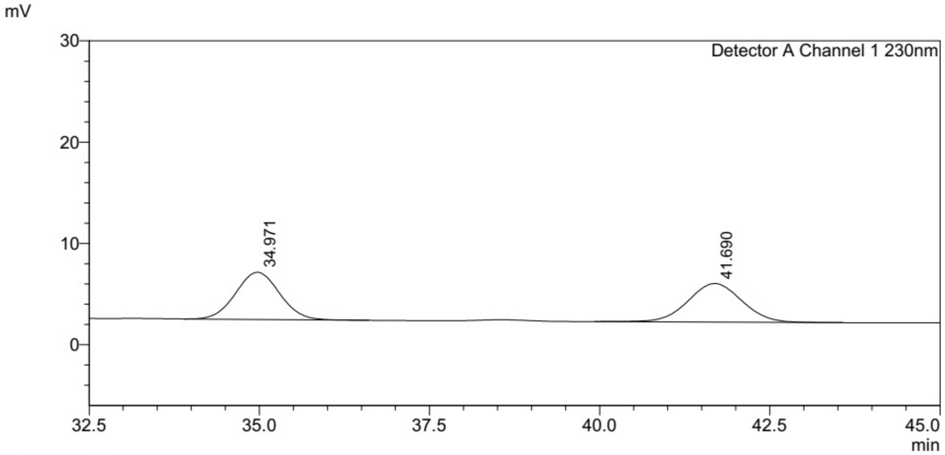

<Peak Table>

Detector A Channel 1 230nm

Peak#	Ret. Time	Area	Height	Area%
1	34.971	210457	4660	49.981
2	41.690	210620	3802	50.019
Total		421077	8462	100.000

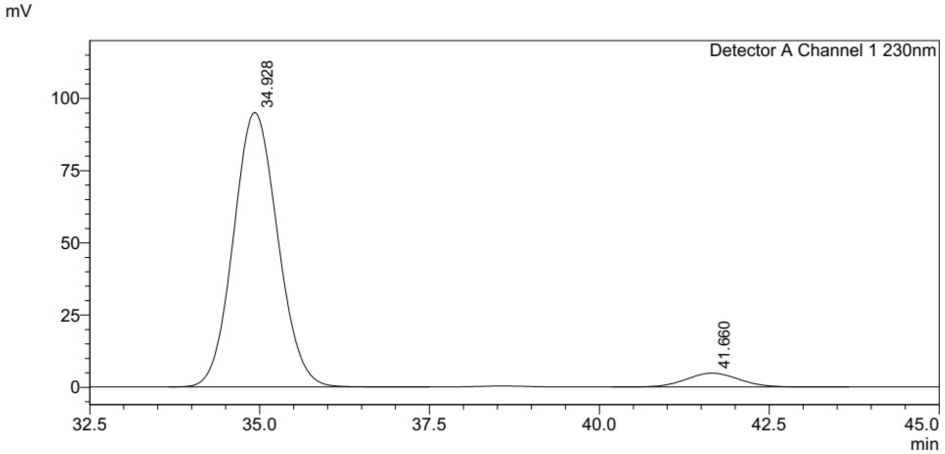

<Peak Table>

Detector A Channel 1 230nm

Peak#	Ret. Time	Area	Height	Area%
1	34.928	4309910	95124	94.266
2	41.660	262153	4807	5.734
Total		4572063	99931	100.000

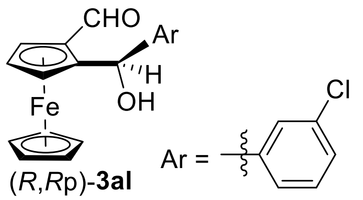

(*R*,*R*p)-**3al**

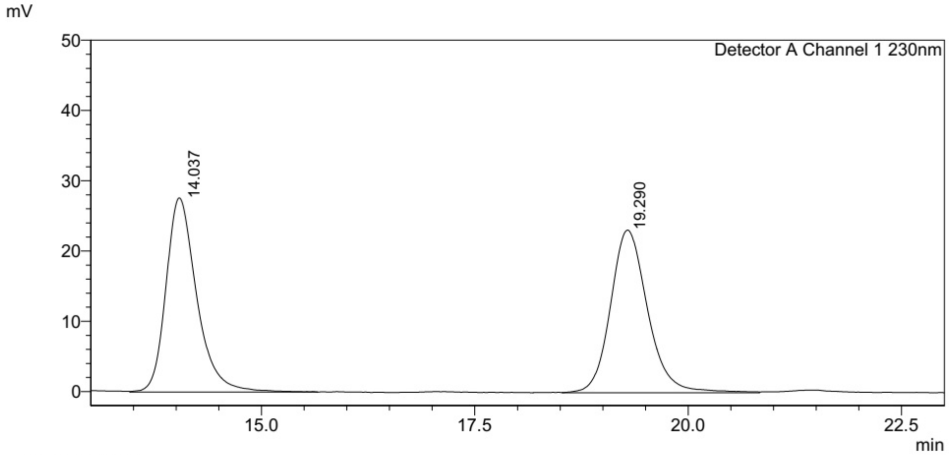

<Peak Table>

Detector A Channel 1 230nm

Peak#	Ret. Time	Area	Height	Area%
1	14.037	692357	27618	49.642
2	19.290	702342	23145	50.358
Total		1394699	50763	100.000

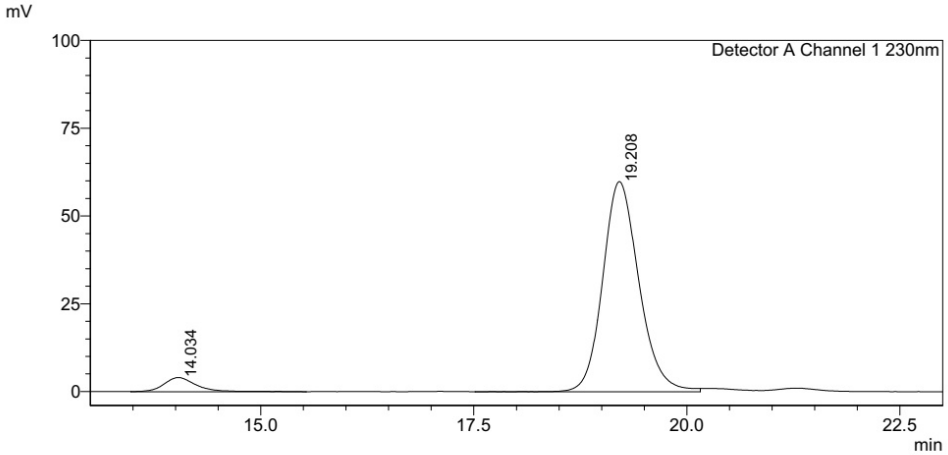

<Peak Table>

Detector A Channel 1 230nm

Peak#	Ret. Time	Area	Height	Area%
1	14.034	106446	4024	5.669
2	19.208	1771125	59845	94.331
Total		1877571	63869	100.000

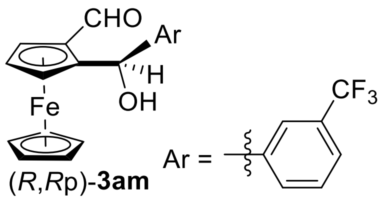

(*R*,*R*p)-**3am**

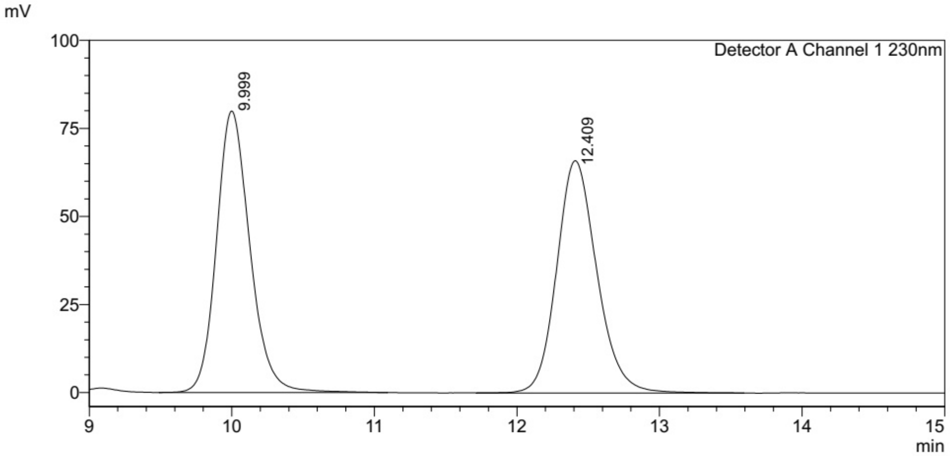

<Peak Table>

Detector A Channel 1 230nm

Peak#	Ret. Time	Area	Height	Area%
1	9.999	1308166	80019	50.225
2	12.409	1296460	66006	49.775
Total		2604626	146026	100.000

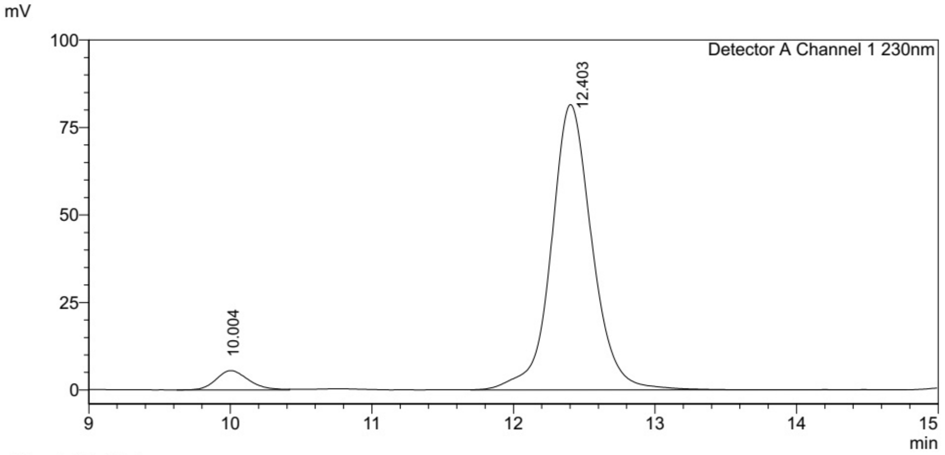

<Peak Table>

Detector A Channel 1 230nm

Peak#	Ret. Time	Area	Height	Area%
1	10.004	88493	5514	5.119
2	12.403	1640282	81520	94.881
Total		1728775	87035	100.000

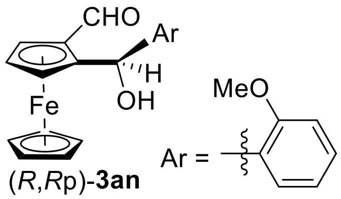

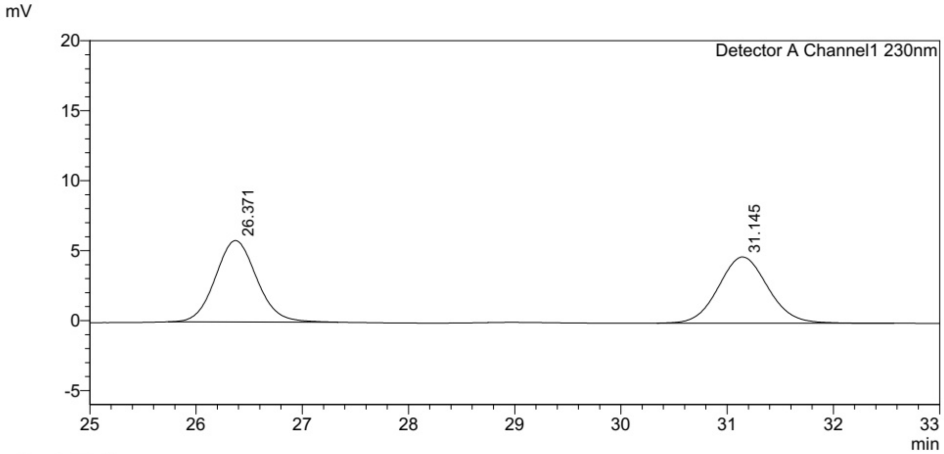

<Peak Table>

Detector A Channel 1 230nm

Peak#	Ret. Time	Area	Height	Area%
1	26.371	157838	5815	50.716
2	31.145	153382	4729	49.284
Total		311220	10544	100.000

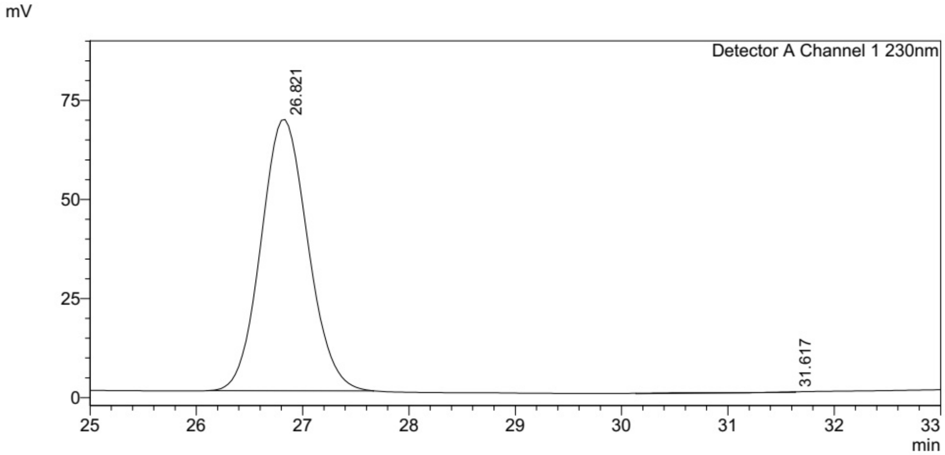

<Peak Table>

Detector A Channel 1 230nm

Peak#	Ret. Time	Area	Height	Area%
1	26.821	2098191	68438	99.741
2	31.617	5448	94	0.259
Total		2103639	68532	100.000

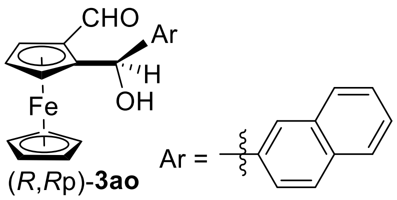

(*R*,*R*p)-**3ao**

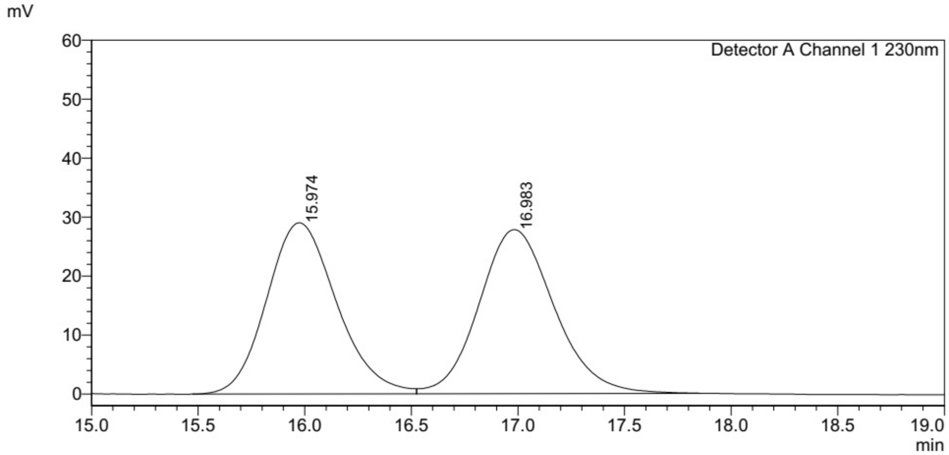

<Peak Table>

Detector A Channel 1 230nm

Peak#	Ret. Time	Area	Height	Area%
1	15.974	664119	29035	49.348
2	16.983	681657	27819	50.652
Total		1345776	56854	100.000

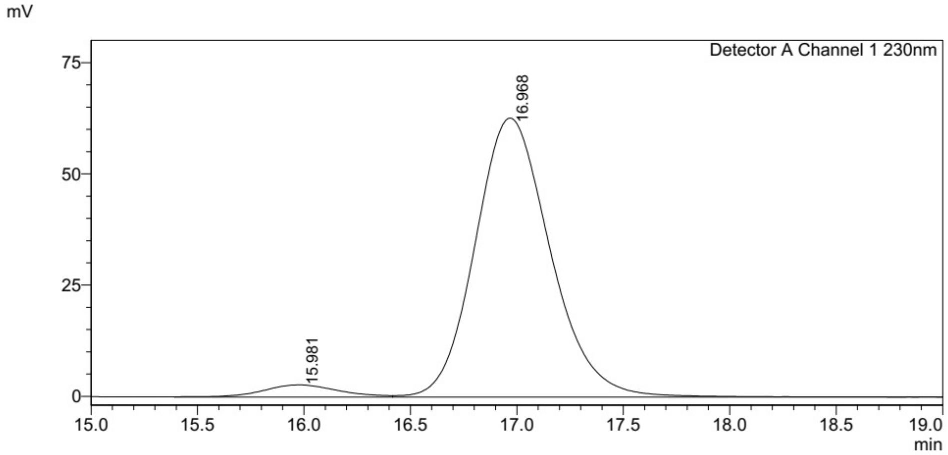

<Peak Table>

Detector A Channel 1 230nm

Peak#	Ret. Time	Area	Height	Area%
1	15.981	69490	2774	4.304
2	16.968	1544987	62761	95.696
Total		1614477	65535	100.000

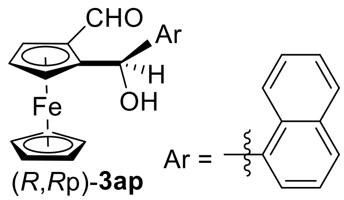

(*R*,*R*p)-**3ap**

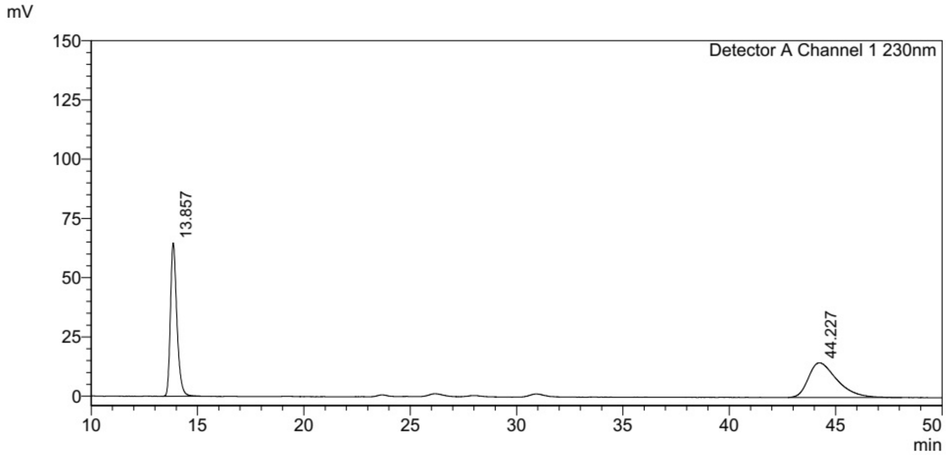

<Peak Table>

Detector A Channel 1 230nm

Peak#	Ret. Time	Area	Height	Area%
1	13.857	1373689	64724	49.950
2	44.227	1376463	14675	50.050
Total		2750153	79399	100.000

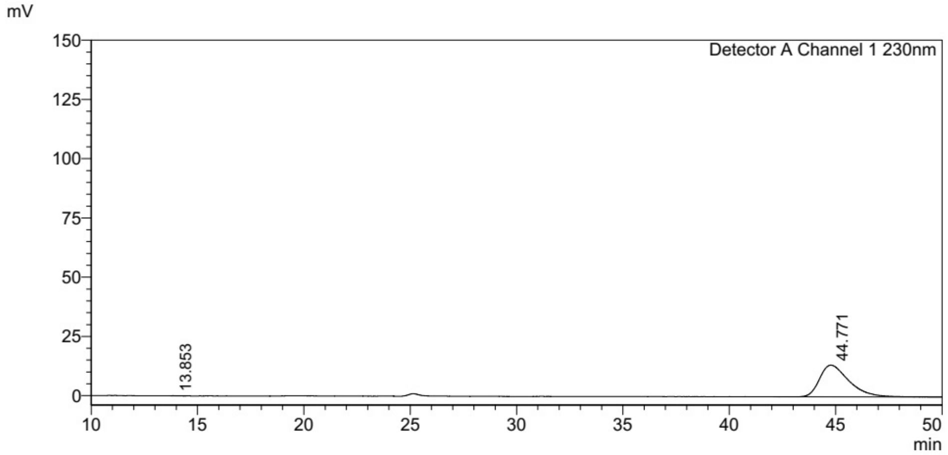

<Peak Table>

Detector A Channel 1 230nm

Peak#	Ret. Time	Area	Height	Area%
1	13.853	1211	79	0.094
2	44.771	1287766	13418	99.906
Total		1288977	13496	100.000

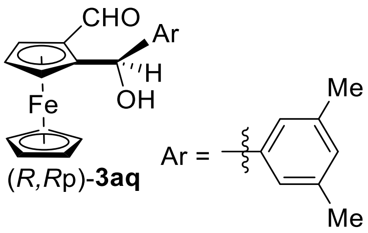

(*R*,*R*p)-**3aq**

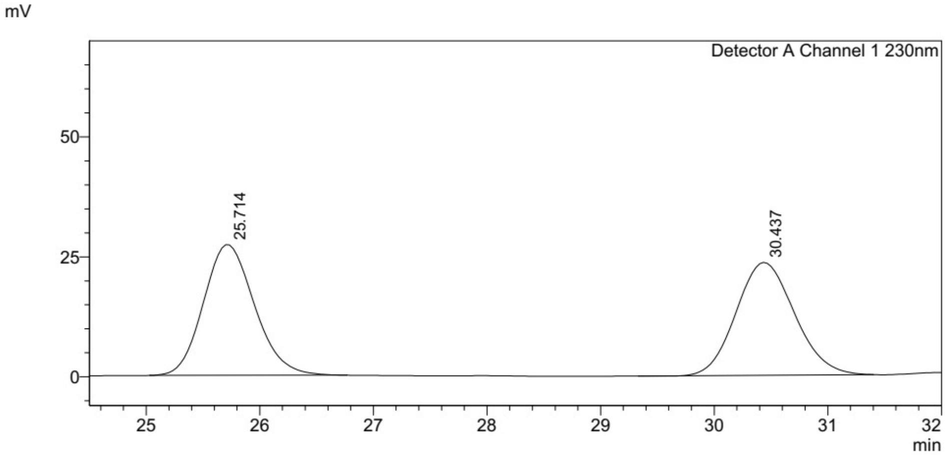

<Peak Table>

Detector A Channel 1 230nm

Peak#	Ret. Time	Area	Height	Area%
1	25.714	852668	27214	50.300
2	30.437	842484	23535	49.700
Total		1695153	50748	100.000

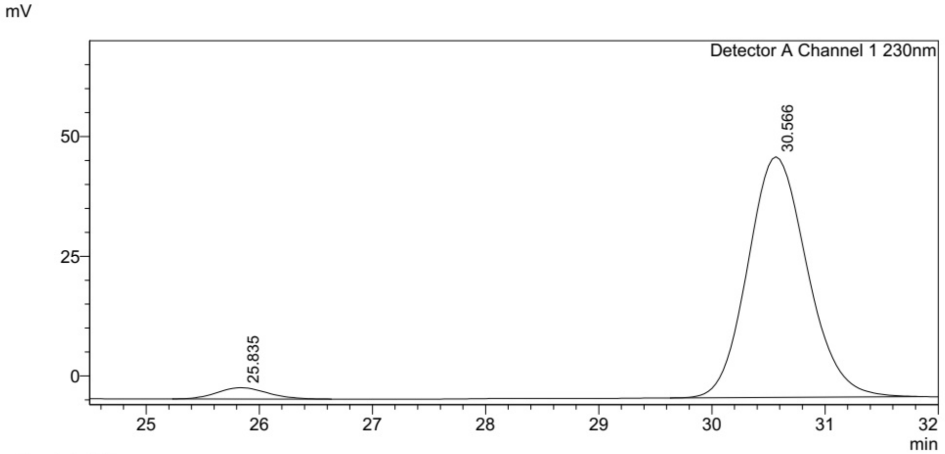

<Peak Table>

Detector A Channel 1 230nm

Peak#	Ret. Time	Area	Height	Area%
1	25.835	73185	2361	3.832
2	30.566	1836794	50237	96.168
Total		1909979	52598	100.000

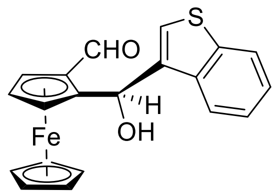

(*R*,*R*p)-**3ar**

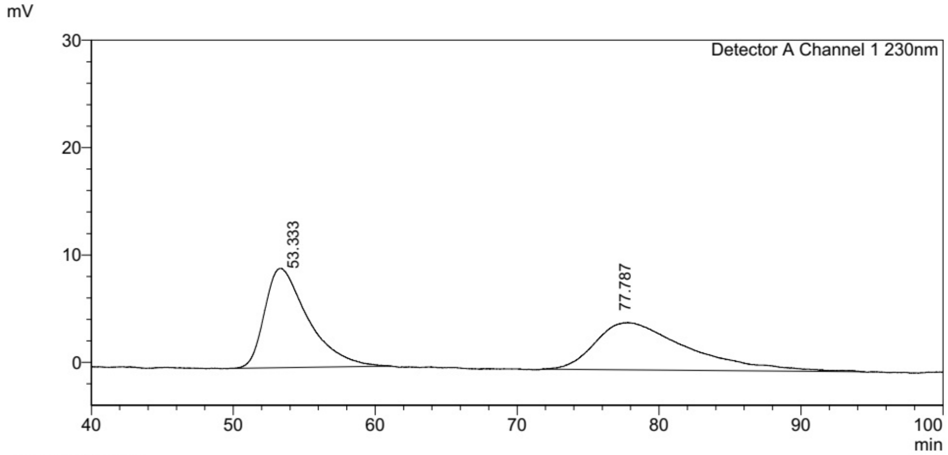

<Peak Table>

Detector A Channel 1 230nm

Peak#	Ret. Time	Area	Height	Area%
1	53.333	1963900	9241	49.546
2	77.787	1999926	4401	50.454
Total		3963826	13642	100.000

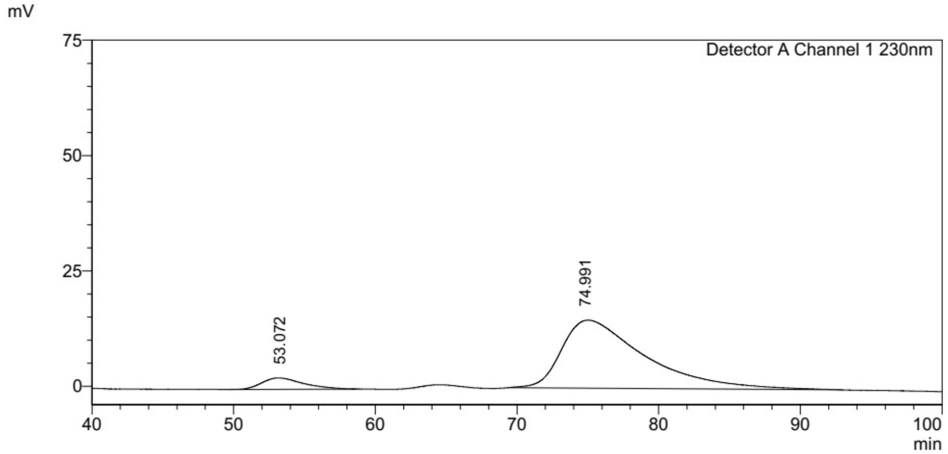

<Peak Table>

Detector A Channel 1 230nm

Peak#	Ret. Time	Area	Height	Area%
1	53.072	497142	2458	7.614
2	74.991	6032035	14760	92.386
Total		6529176	17218	100.000

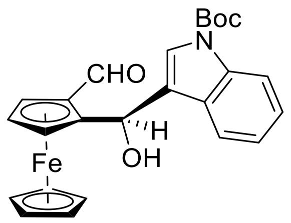

(*R*,*R*p)-**3as**

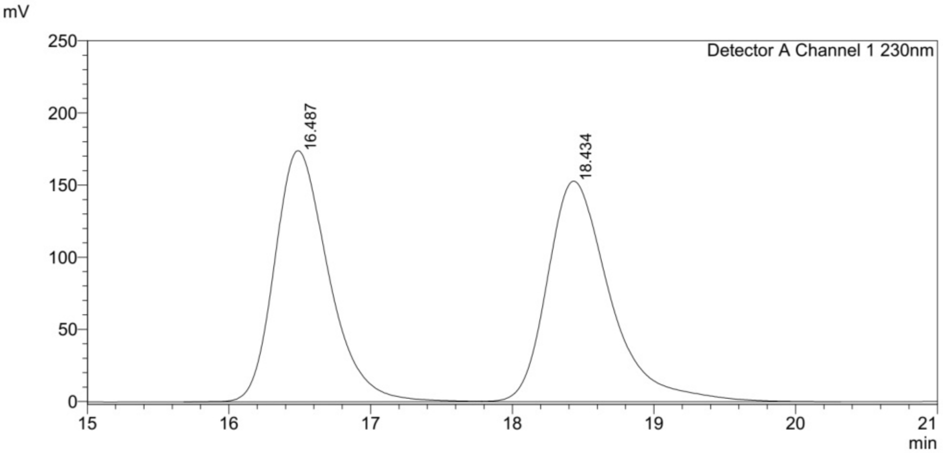

<Peak Table>

Detector A Channel 1 230nm

Peak#	Ret. Time	Area	Height	Area%
1	16.487	4610998	174068	49.485
2	18.434	4706897	152971	50.515
Total		9317895	327039	100.000

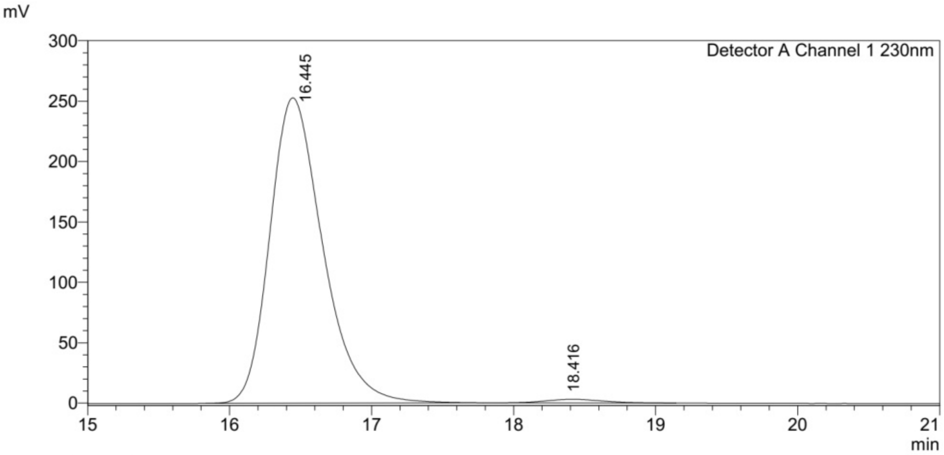

<Peak Table>

Detector A Channel 1 230nm

Peak#	Ret. Time	Area	Height	Area%
1	16.445	6559970	252788	98.719
2	18.416	85126	3061	1.281
Total		6645097	255849	100.000

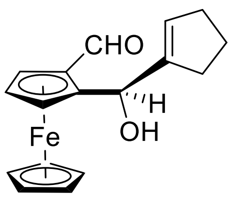

(*R*,*R*p)-**3at**

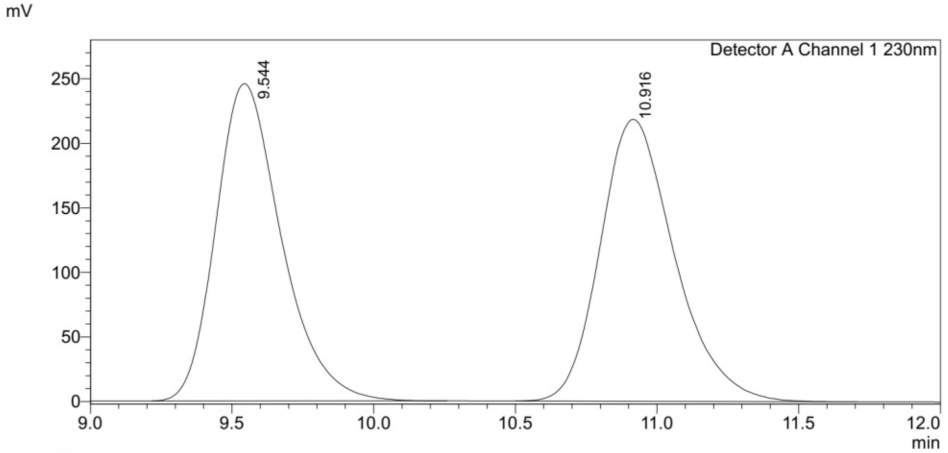

<Peak Table>

Detector A Channel 1 230nm

Peak#	Ret. Time	Area	Height	Area%
1	9.544	3962715	245934	49.865
2	10.916	3984135	218544	50.135
Total		7946850	464478	100.000

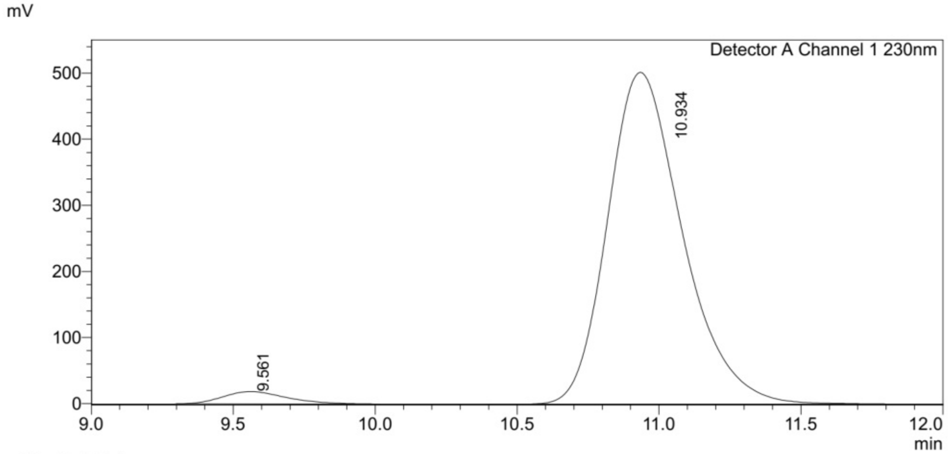

<Peak Table>

Detector A Channel 1 230nm

Peak#	Ret. Time	Area	Height	Area%
1	9.561	296302	18763	3.113
2	10.934	9222073	502163	96.887
Total		9518375	520926	100.000

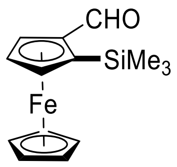

(*Sp*)-**1e**

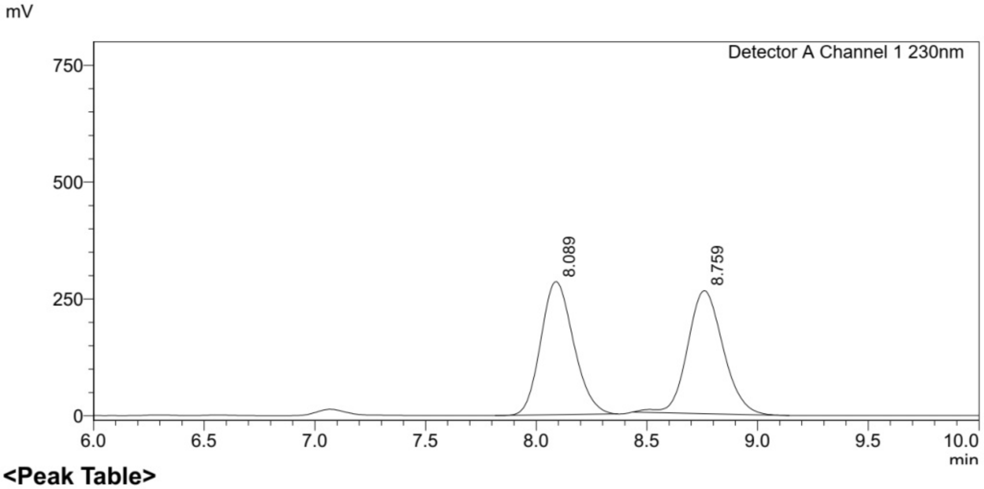

<Peak Table>

Detector A Channel 1 230nm

Peak#	Ret. Time	Area	Height	Area%
1	8.089	2945520	284840	49.892
2	8.759	2958291	263536	50.108
Total		5903810	548376	100.000

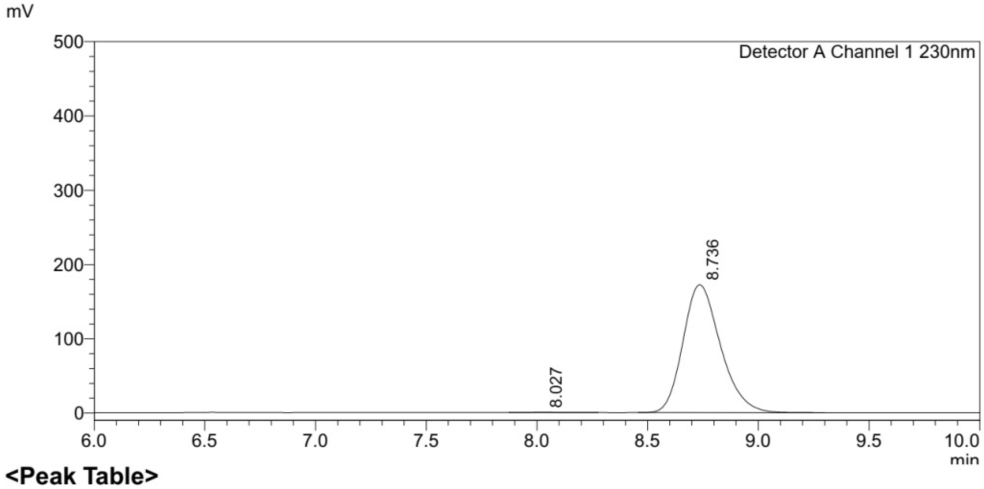

<Peak Table>

Detector A Channel 1 230nm

Peak#	Ret. Time	Area	Height	Area%
1	8.027	3285	325	0.161
2	8.736	2038092	172322	99.839
Total		2041377	172647	100.000

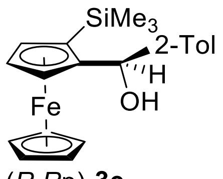

(*R*,*R*p)-**3e**

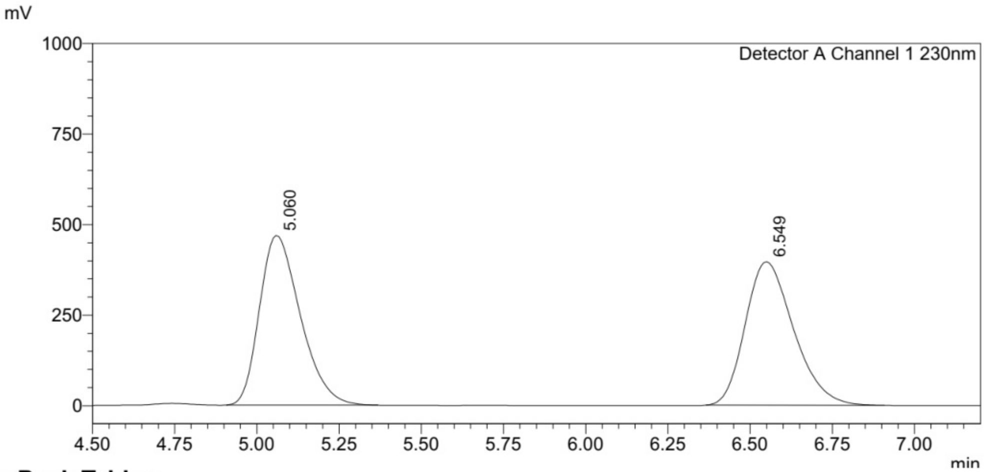

<Peak Table>

Detector A Channel 1 230nm

Peak#	Ret. Time	Area	Height	Area%
1	5.060	4049393	468303	49.980
2	6.549	4052645	396332	50.020
Total		8102038	864635	100.000

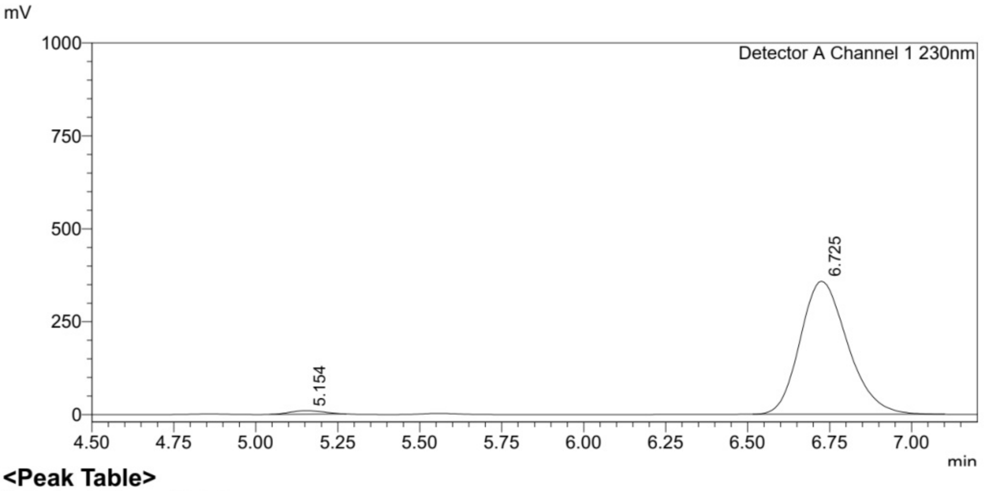

<Peak Table>

Detector A Channel 1 230nm

Peak#	Ret. Time	Area	Height	Area%
1	5.154	66471	9243	1.808
2	6.725	3609524	357783	98.192
Total		3675995	367026	100.000

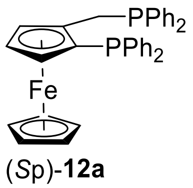

(*S*p)-**12a**

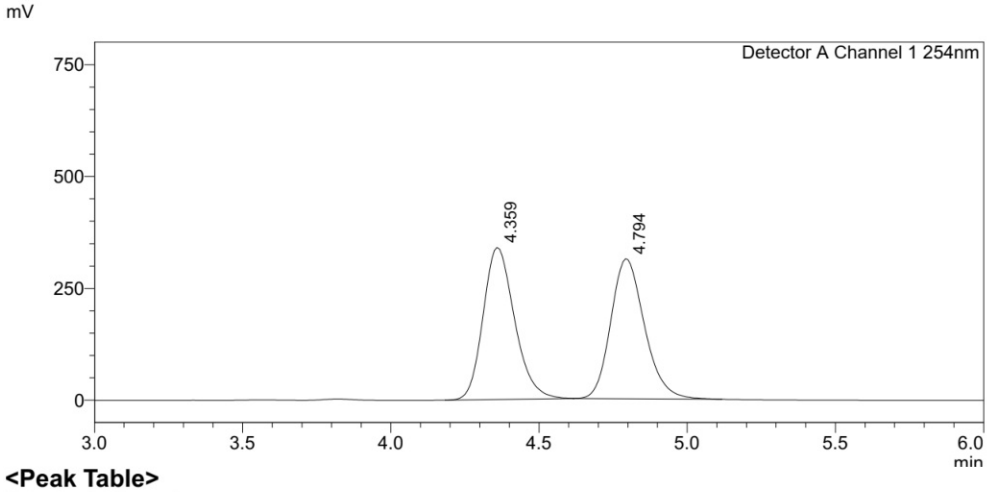

<Peak Table>

Detector A Channel 1 254nm

Peak#	Ret. Time	Area	Height	Area%
1	4.359	2505315	339449	50.358
2	4.794	2469718	312817	49.642
Total		4975033	652266	100.000

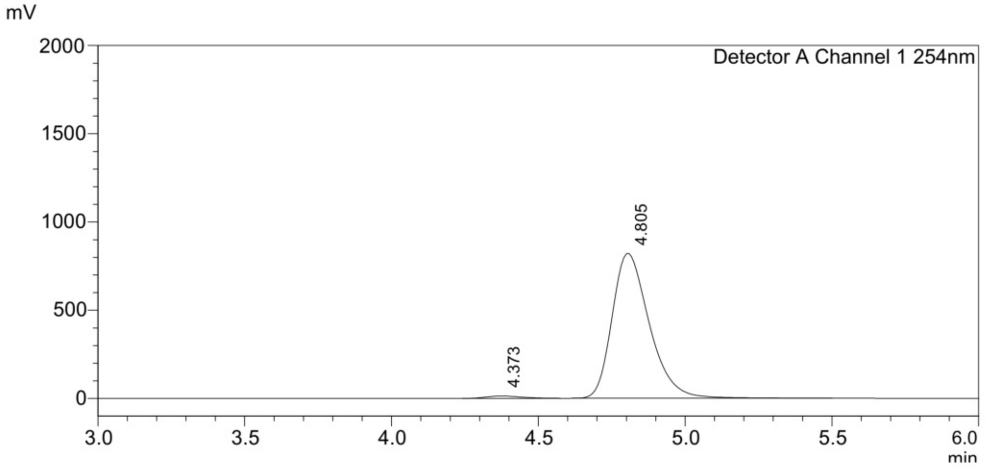

<Peak Table>

Detector A Channel 1 254nm

Peak#	Ret. Time	Area	Height	Area%
1	4.373	113944	13966	1.549
2	4.805	7243010	820187	98.451
Total		7356954	834153	100.000

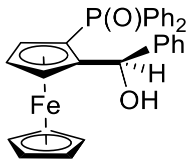

(*R*,*R*p)-**3f**

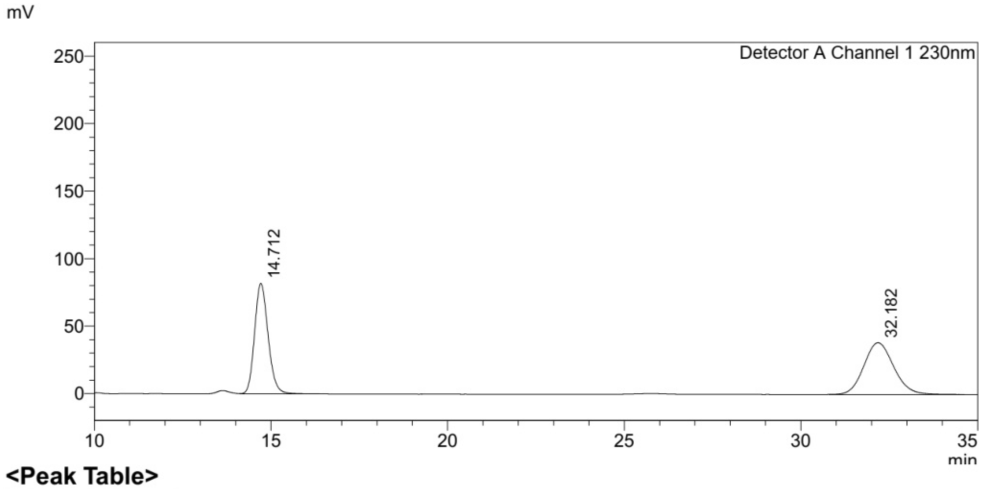

<Peak Table>

Detector A Channel 1 230nm

Peak#	Ret. Time	Area	Height	Area%
1	14.712	2182821	81865	49.088
2	32.182	2263968	38389	50.912
Total		4446789	120254	100.000

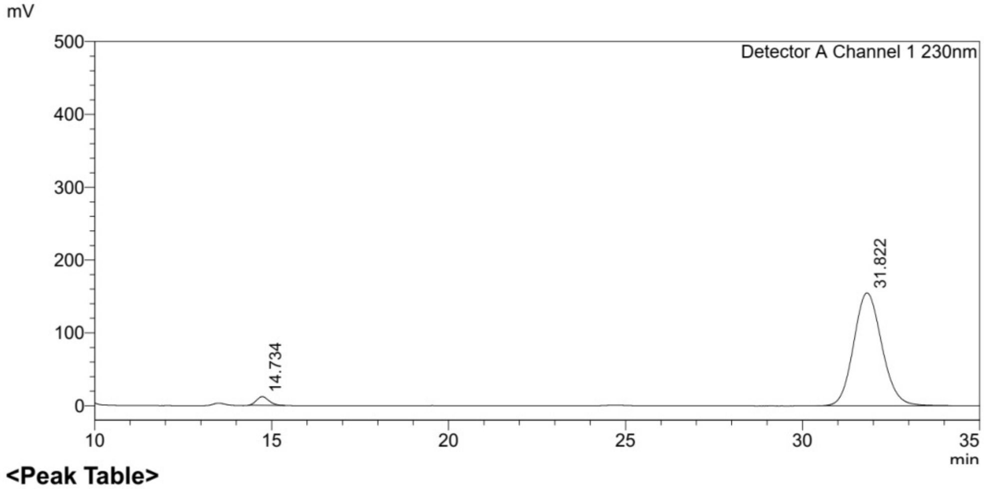

<Peak Table>

Detector A Channel 1 230nm

Peak#	Ret. Time	Area	Height	Area%
1	14.734	289484	11812	3.265
2	31.822	8575968	154853	96.735
Total		8865451	166665	100.000

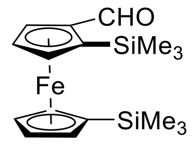

(*S*p)-**1g**

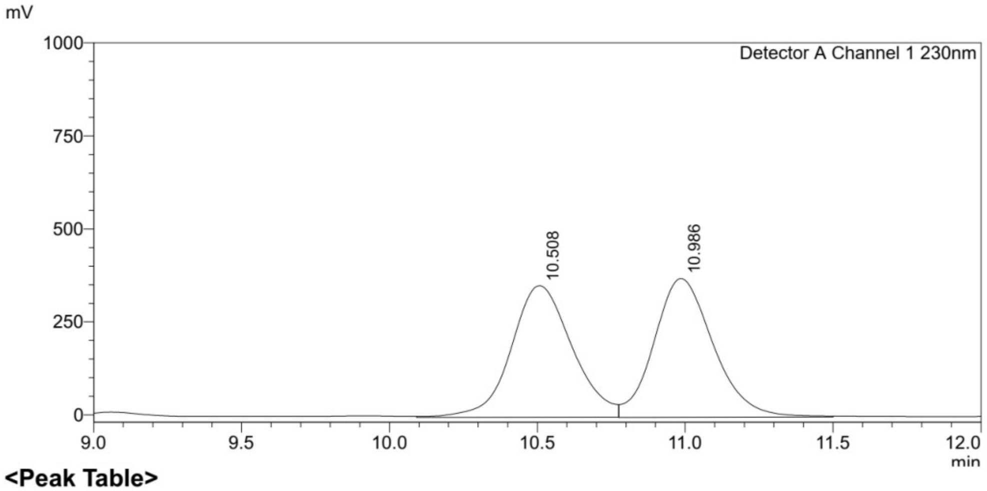

<Peak Table>

Detector A Channel 1 230nm

Peak#	Ret. Time	Area	Height	Area%
1	10.508	5089602	354047	49.475
2	10.986	5197704	373157	50.525
Total		10287306	727205	100.000

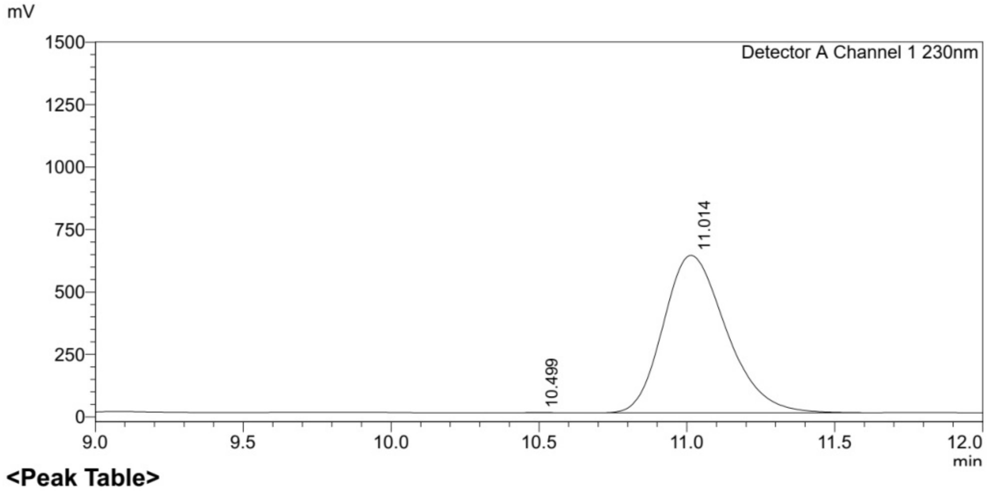

<Peak Table>

Detector A Channel 1 230nm

Peak#	Ret. Time	Area	Height	Area%
1	10.499	12240	970	0.131
2	11.014	9346431	630163	99.869
Total		9358671	631133	100.000

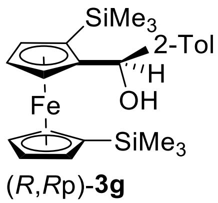

(*R*,*R*p)-**3g**

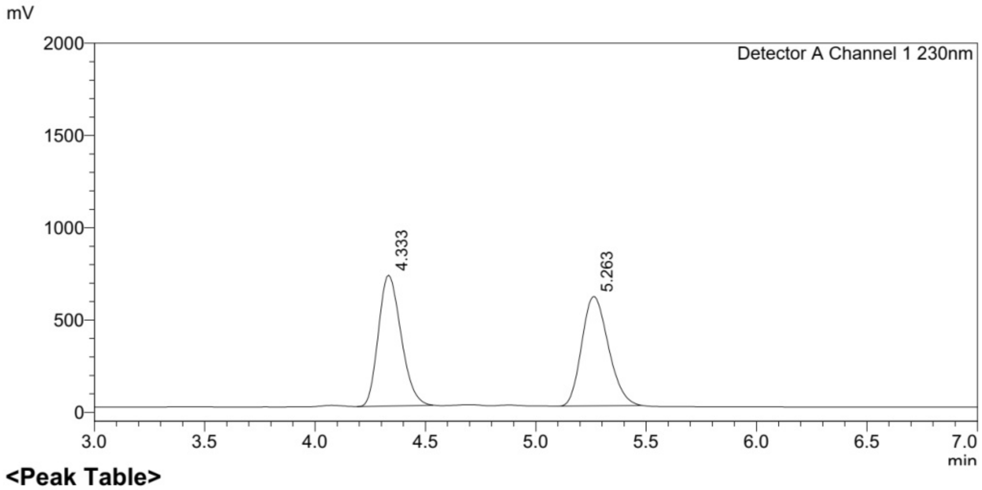

<Peak Table>

Detector A Channel 1 230nm

Peak#	Ret. Time	Area	Height	Area%
1	4.333	5067708	709305	50.341
2	5.263	4999062	591979	49.659
Total		10066770	1301284	100.000

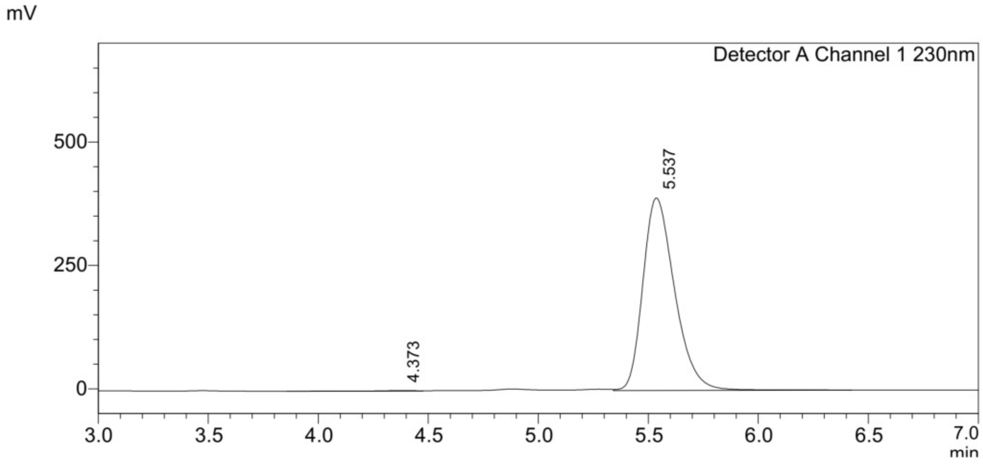

<Peak Table>

Detector A Channel 1 230nm

Peak#	Ret. Time	Area	Height	Area%
1	4.373	10415	792	0.265
2	5.537	3922625	389336	99.735
Total		3933040	390128	100.000

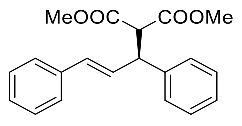

23

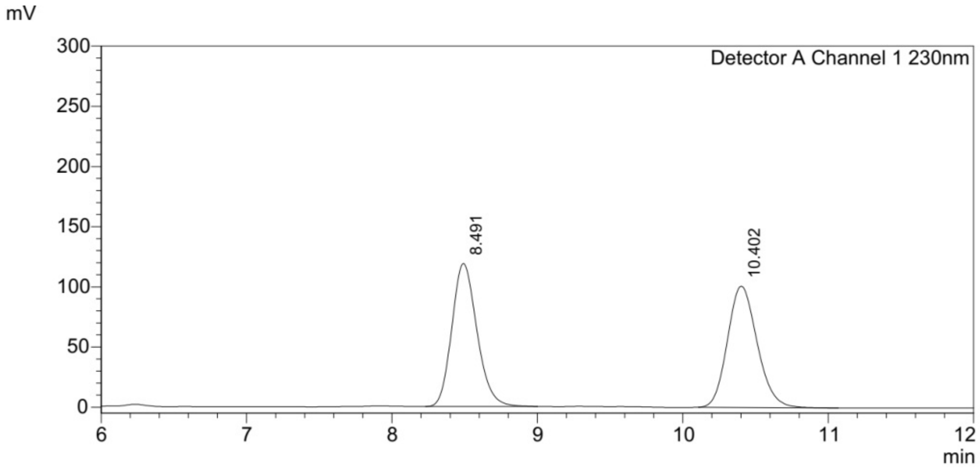

<Peak Table>

Detector A Channel 1 230nm

Peak#	Ret. Time	Area	Height	Area%
1	8.491	1428714	118962	49.948
2	10.402	1431688	100792	50.052
Total		2860402	219754	100.000

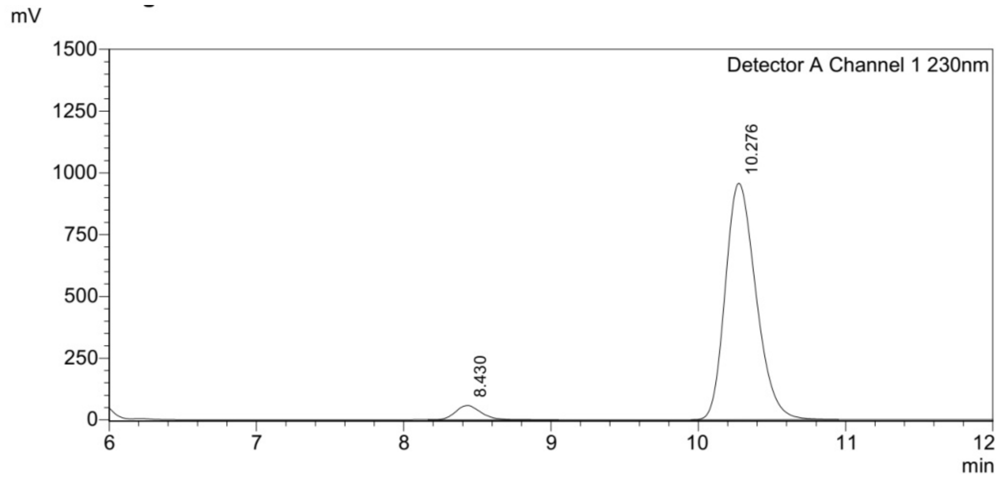

<Peak Table>

Detector A Channel 1 230nm

Peak#	Ret. Time	Area	Height	Area%
1	8.430	696633	57782	4.781
2	10.276	13874595	957482	95.219
Total		14571229	1015263	100.000

^{1}H NMR (600 MHz, $CDCl_3$)

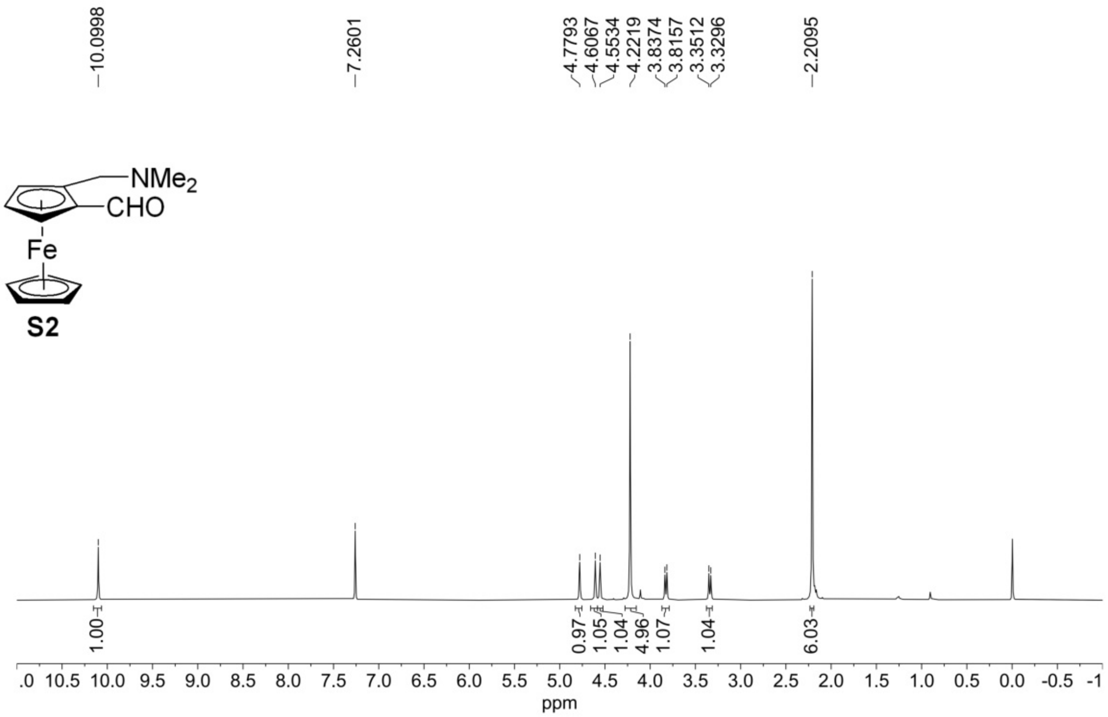

^{1}H NMR (500 MHz, $CDCl_3$)

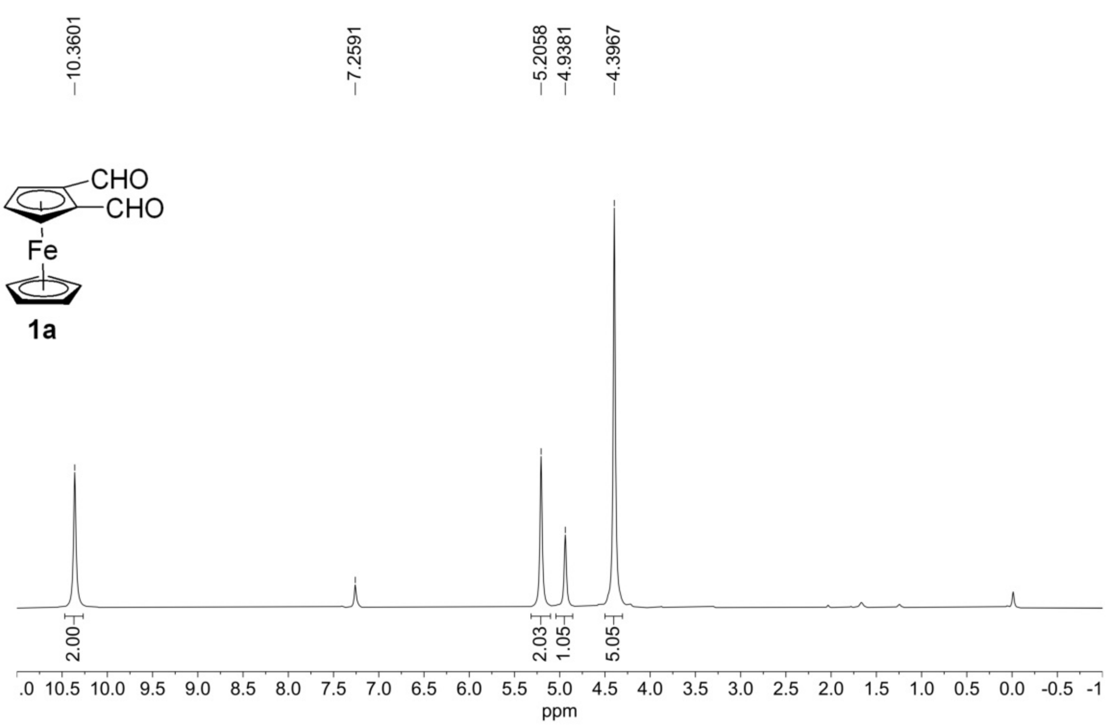

^{1}H NMR (600 MHz, CDCl3)

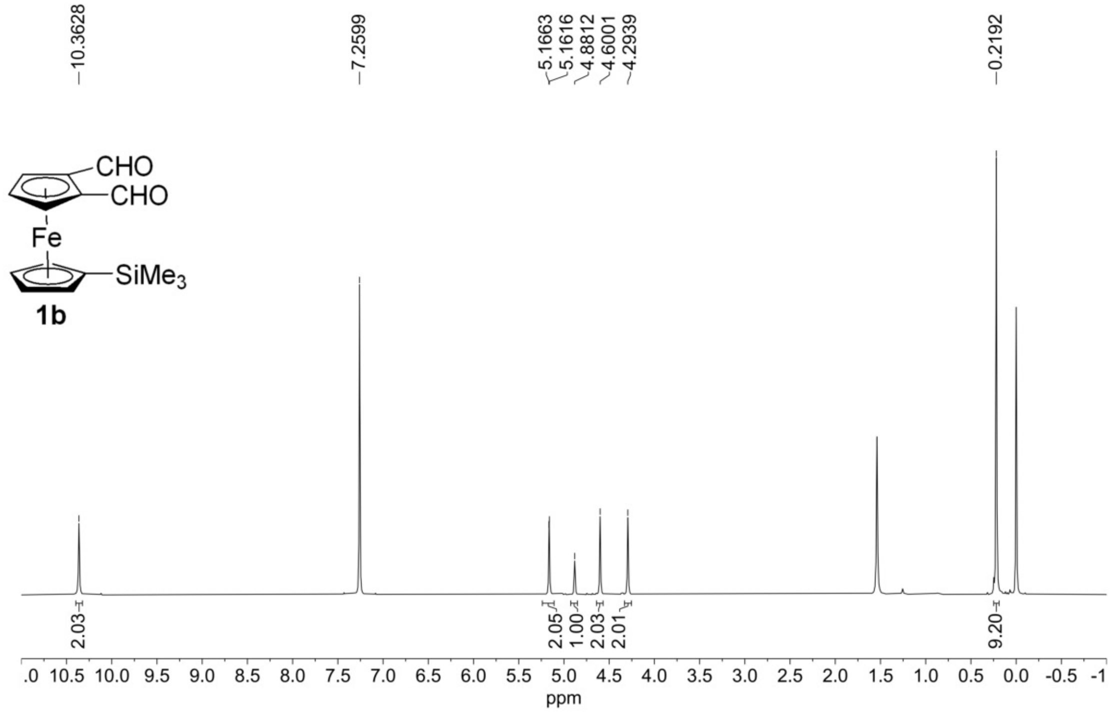

^{1}H NMR (600 MHz, CDCl3)

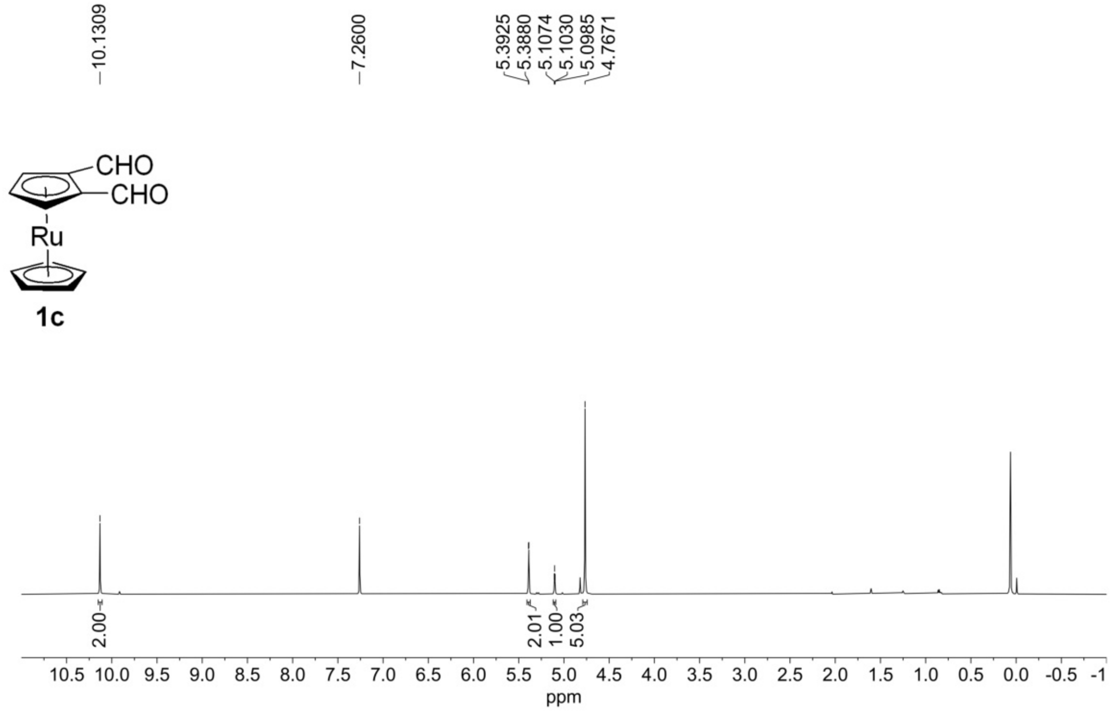

^{1}H NMR (600 MHz, CDCl$_3$)

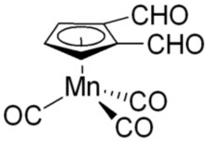

1d

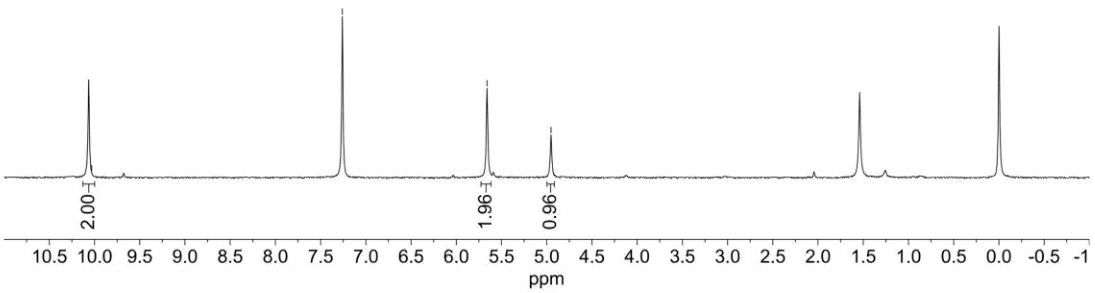

^{1}H NMR (600 MHz, $CDCl_3$)

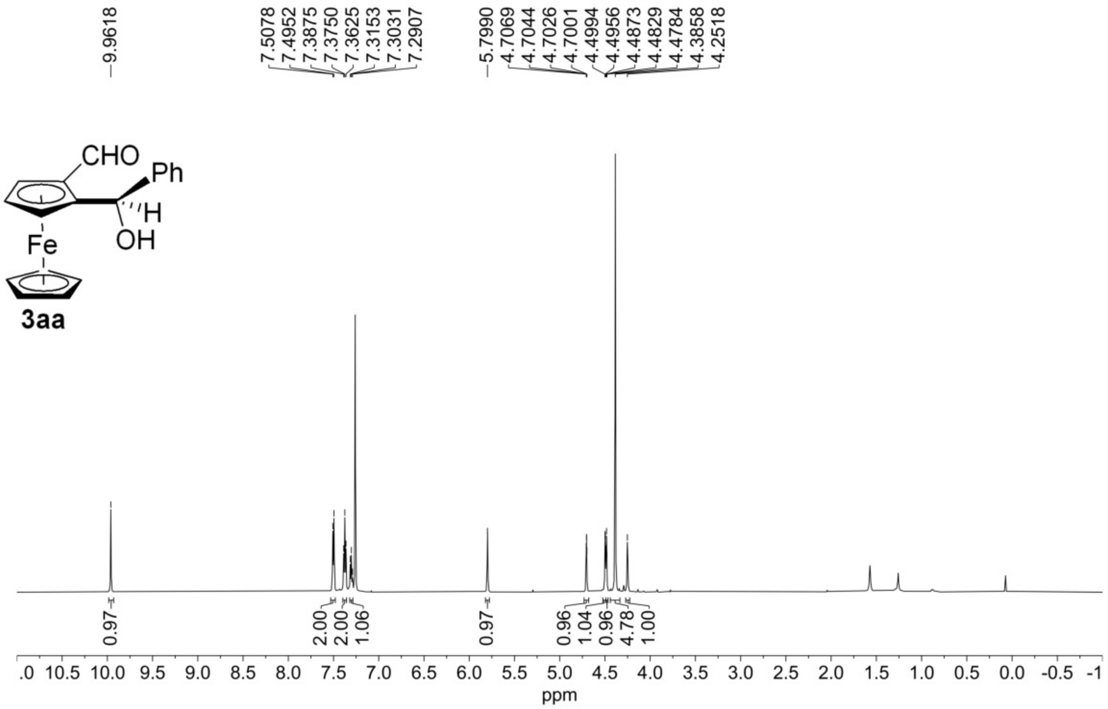

^{13}C NMR (150 MHz, $CDCl_3$)

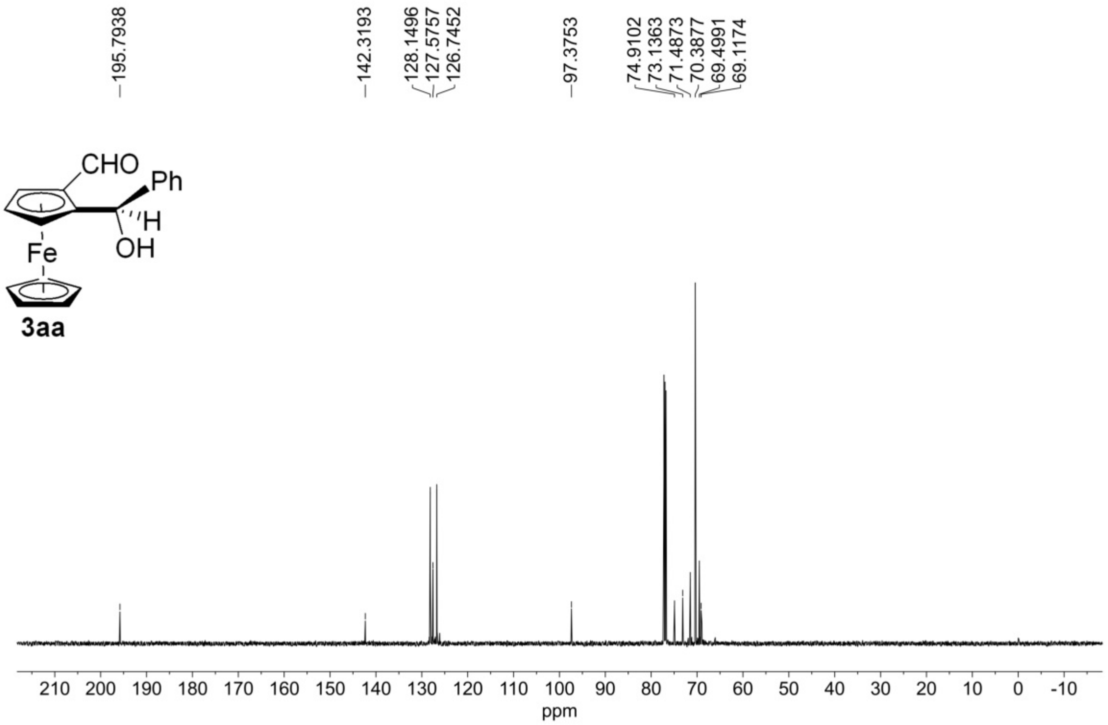

^{1}H NMR (600 MHz, $CDCl_3$)

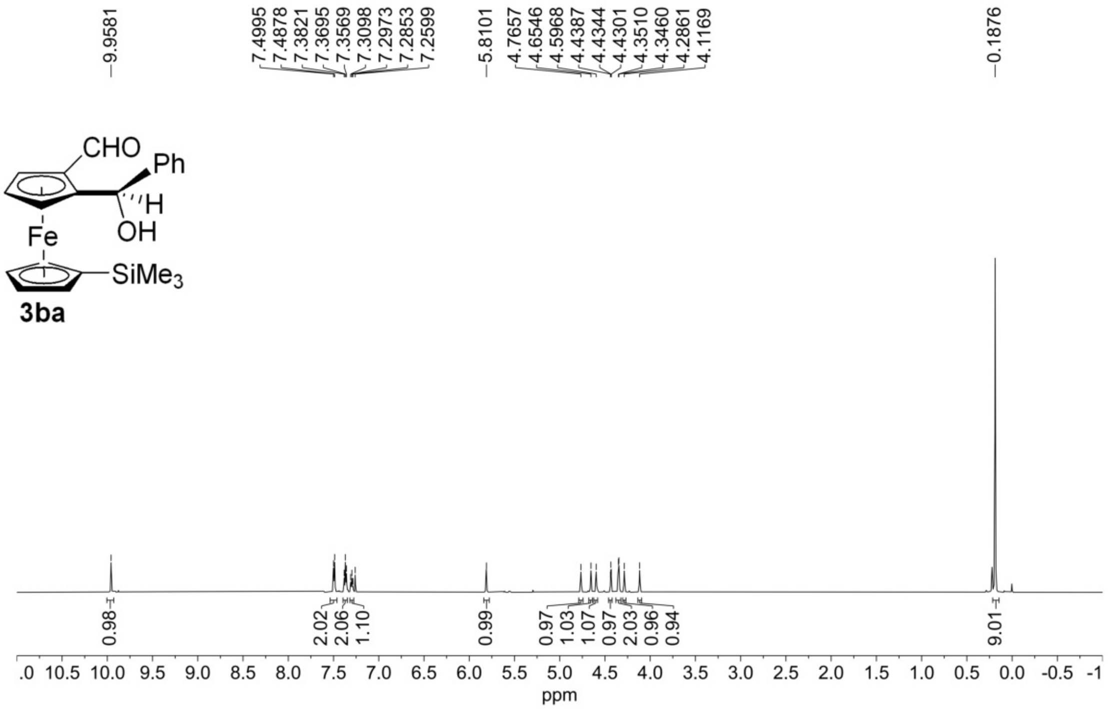

^{13}C NMR (150 MHz, $CDCl_3$)

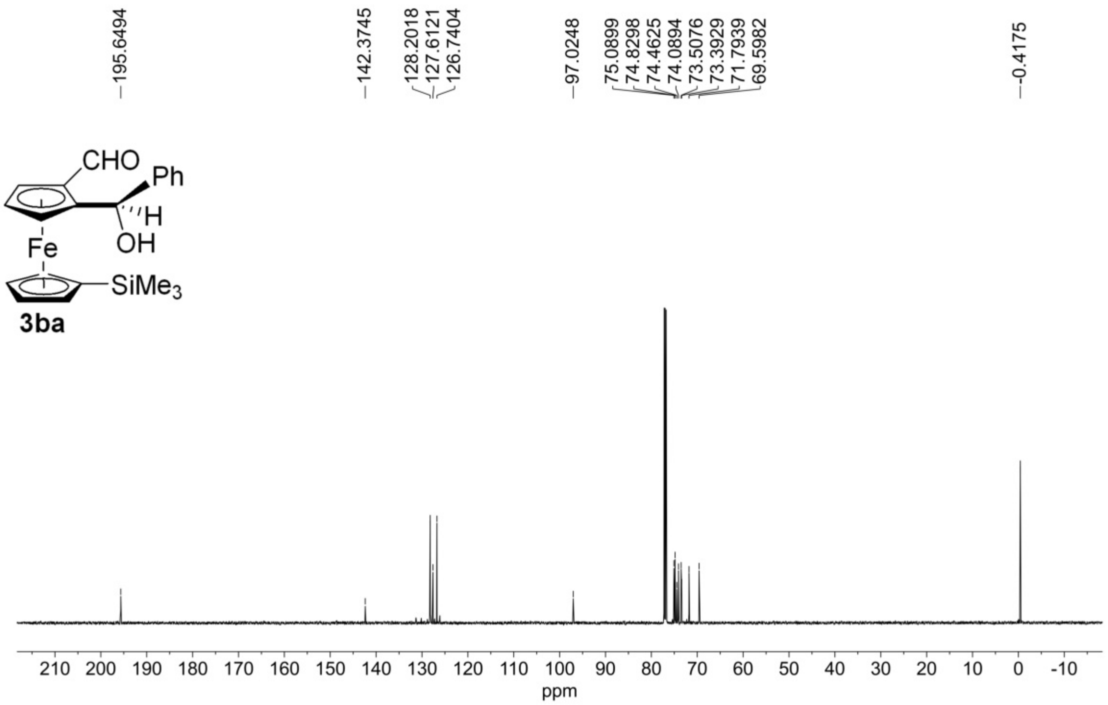

^{1}H NMR (600 MHz, $CDCl_3$)

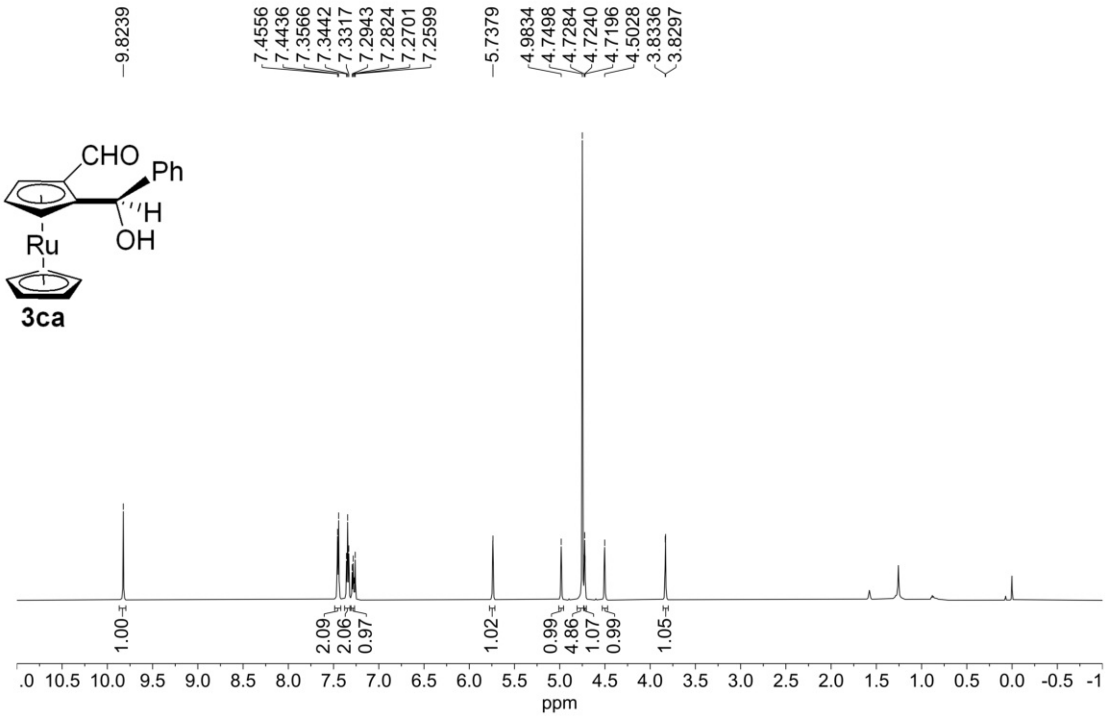

^{13}C NMR (150 MHz, $CDCl_3$)

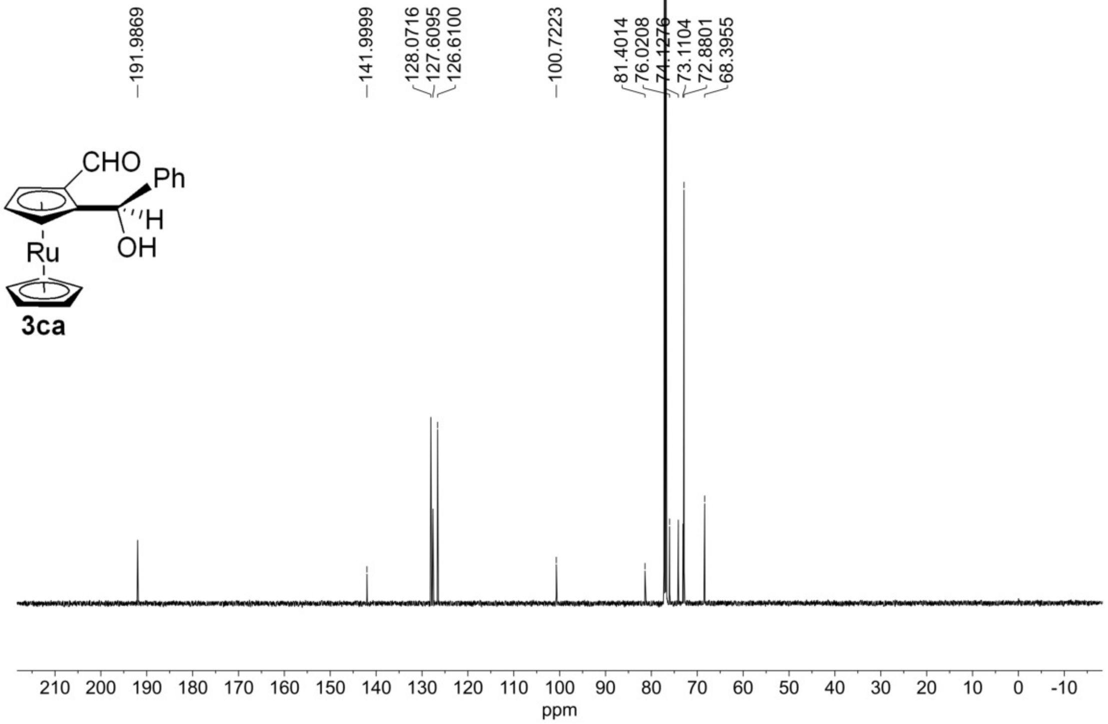

^{1}H NMR (600 MHz, $CDCl_3$)

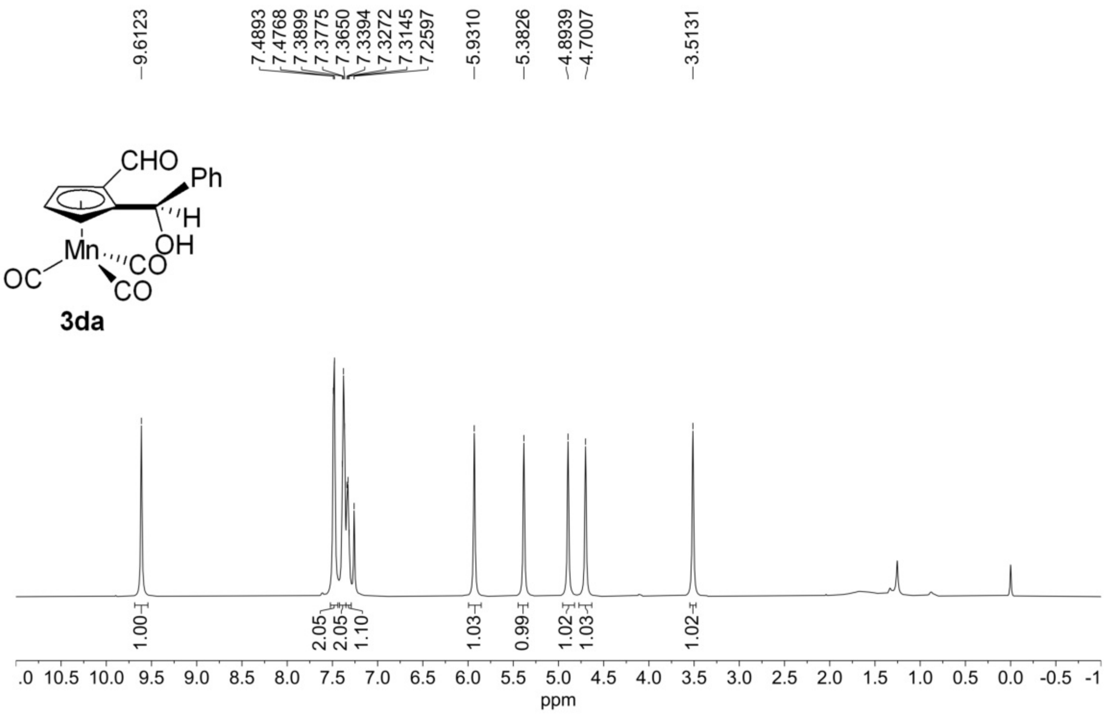

^{13}C NMR (150 MHz, $CDCl_3$)

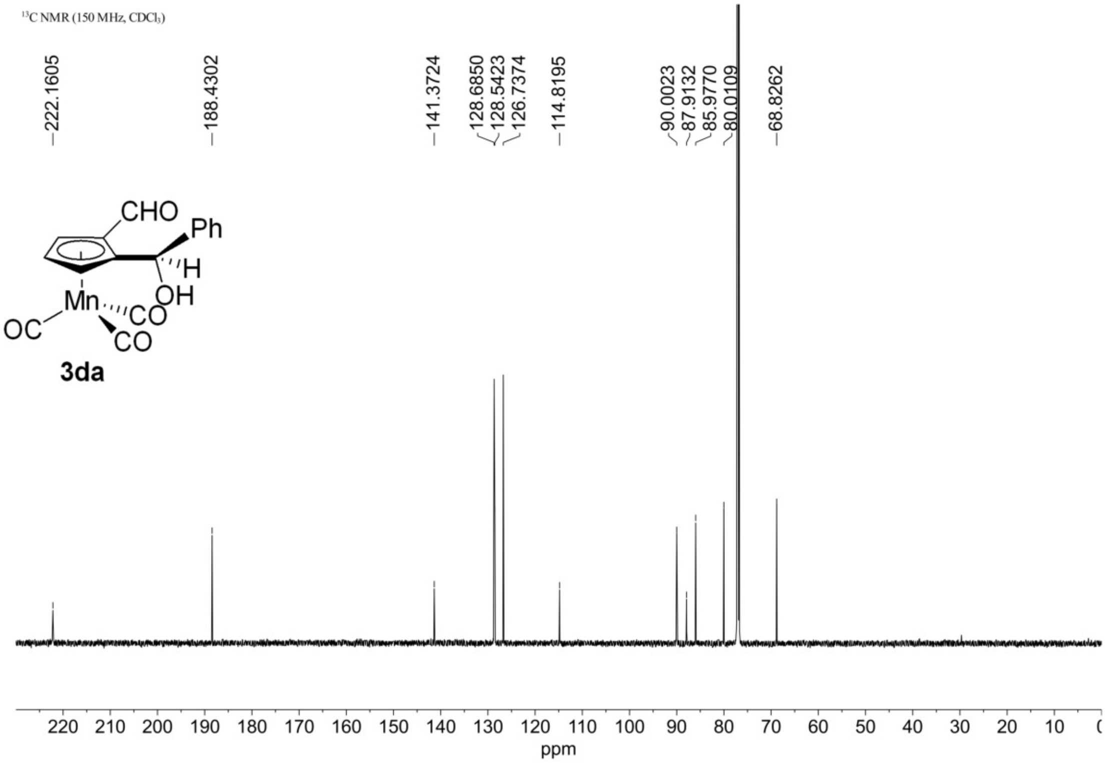

^{1}H NMR (600 MHz, $CDCl_3$)

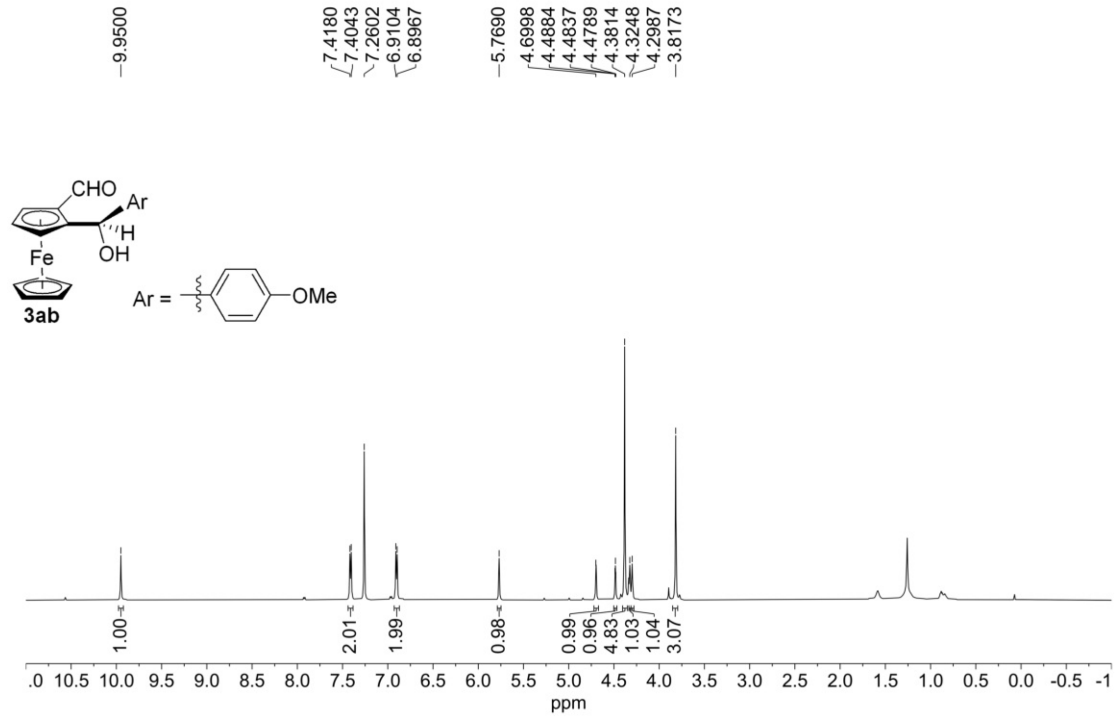

^{13}C NMR (150 MHz, $CDCl_3$)

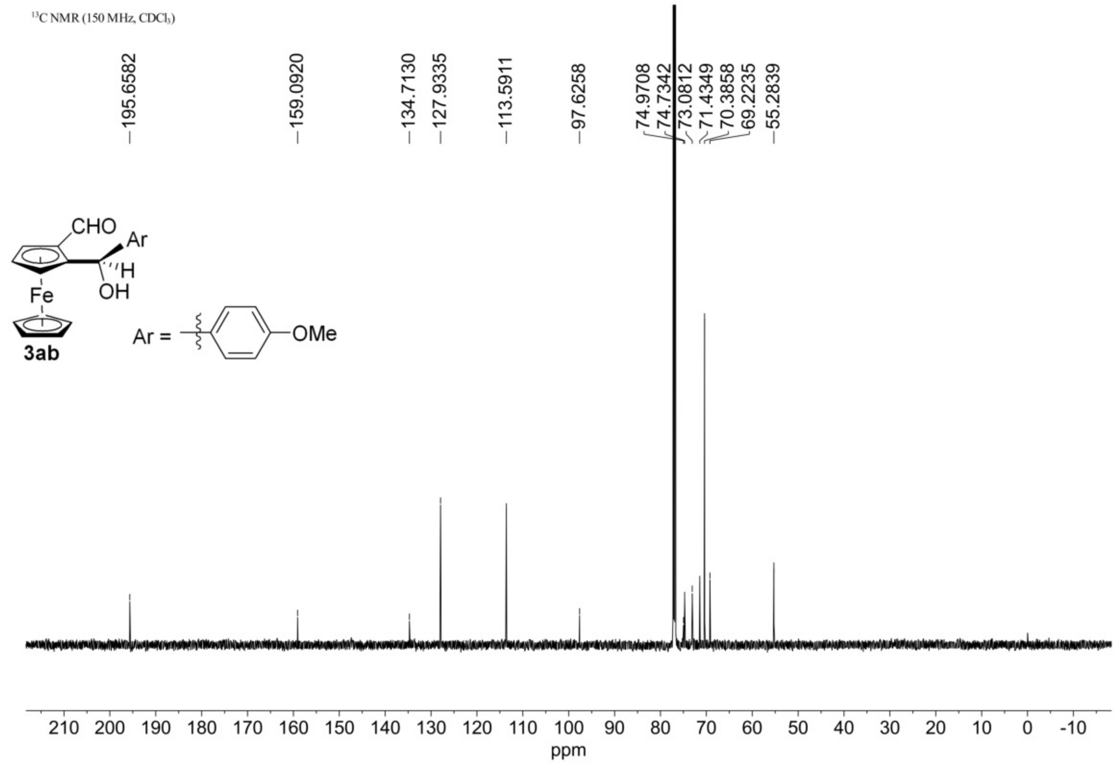

^{1}H NMR (600 MHz, $CDCl_3$)

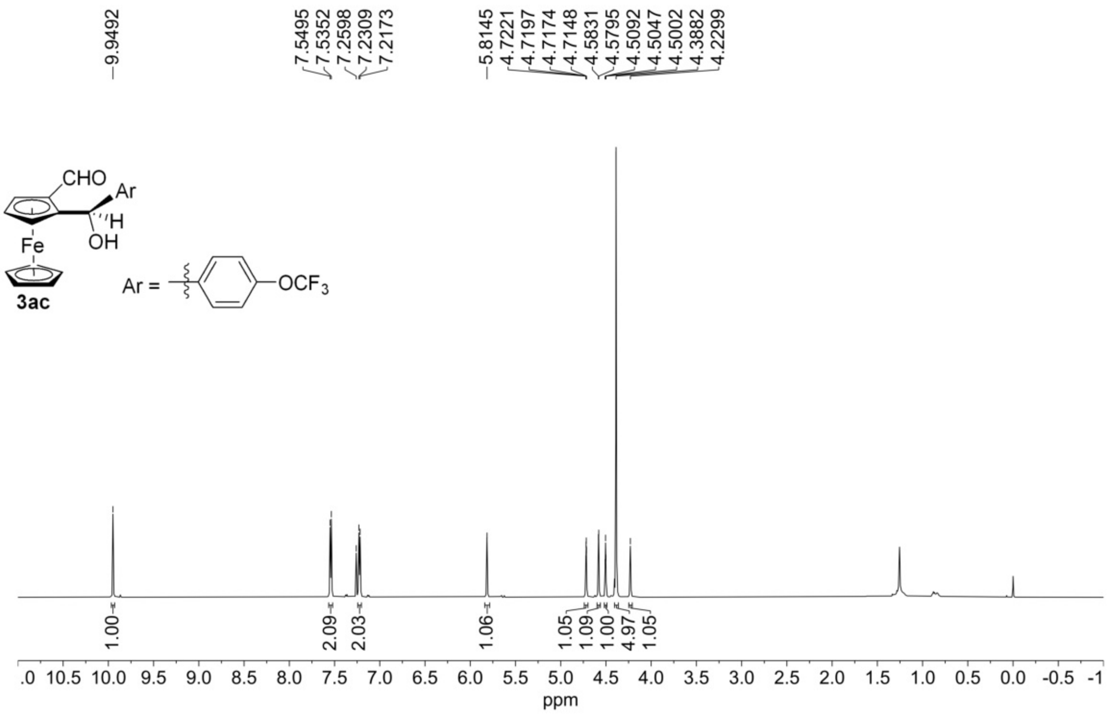

^{13}C NMR (150 MHz, $CDCl_3$)

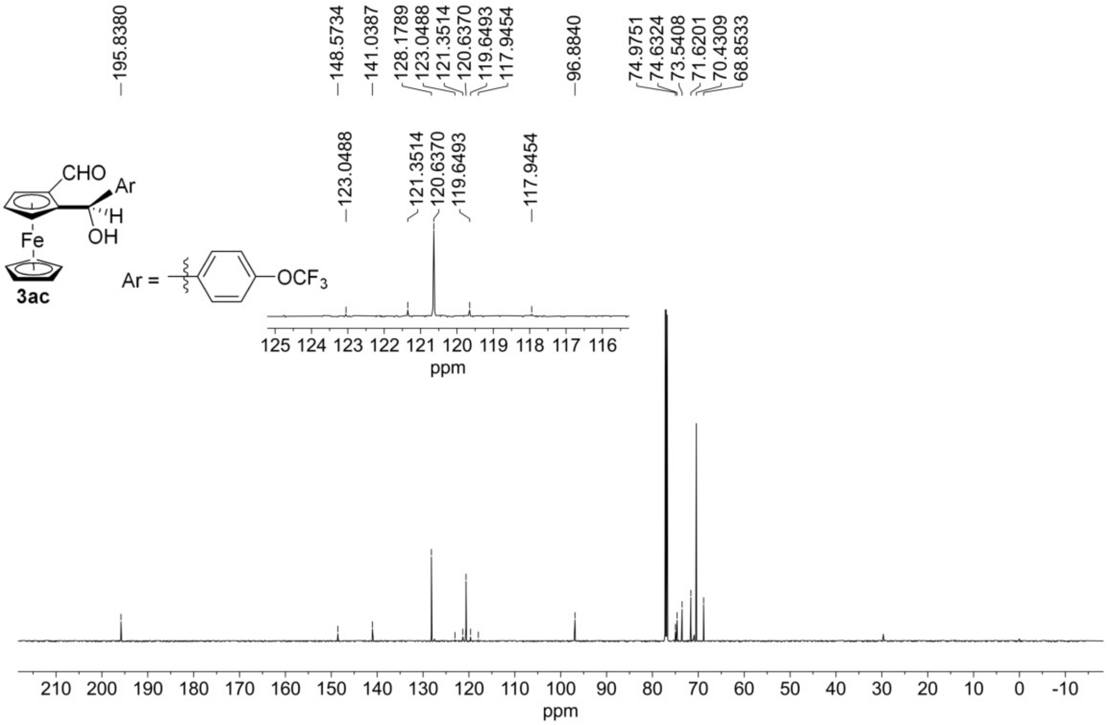

^{1}H NMR (600 MHz, CDCl$_3$)

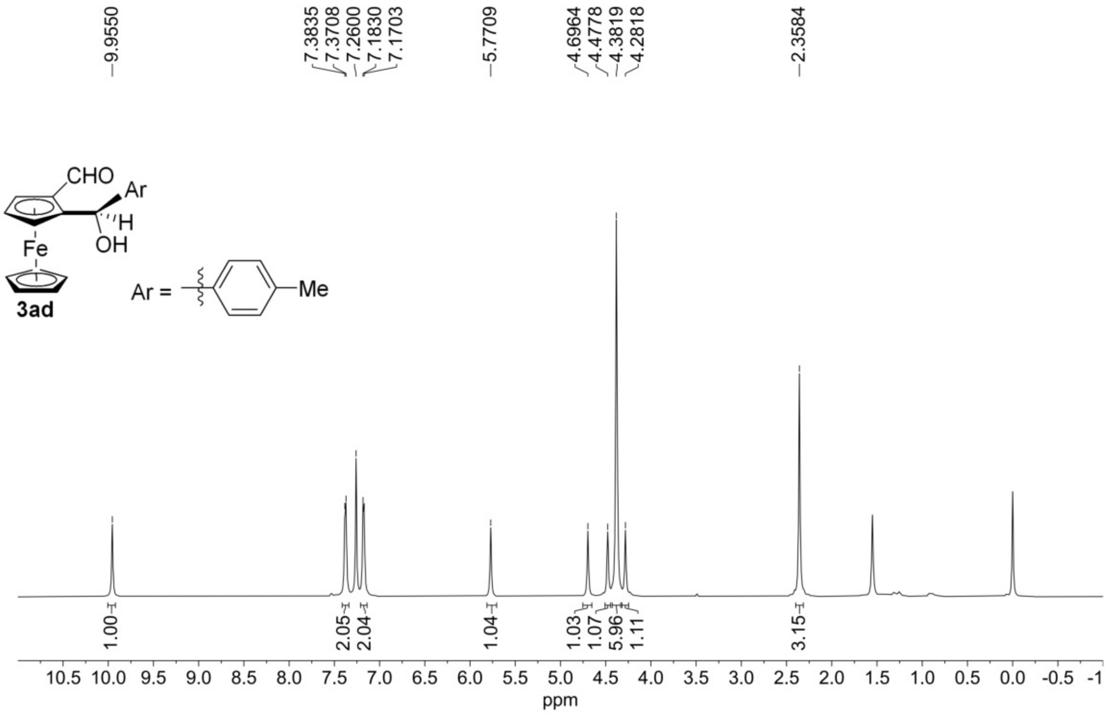

^{13}C NMR (150 MHz, CDCl$_3$)

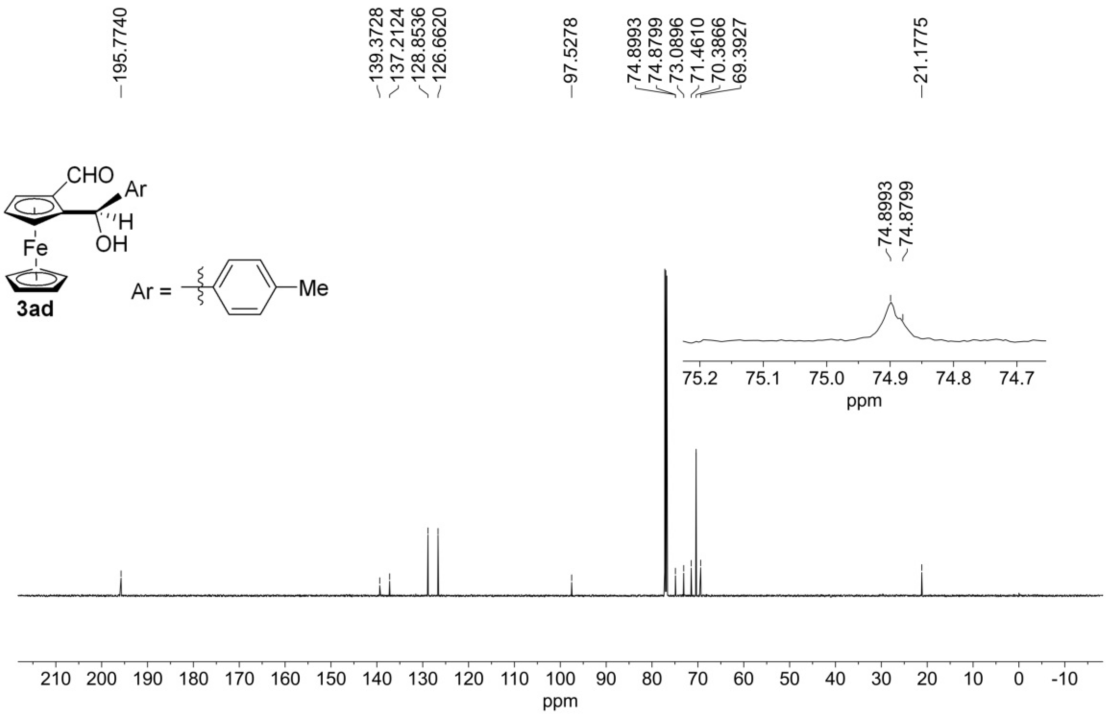

^1H NMR (600 MHz, $CDCl_3$)

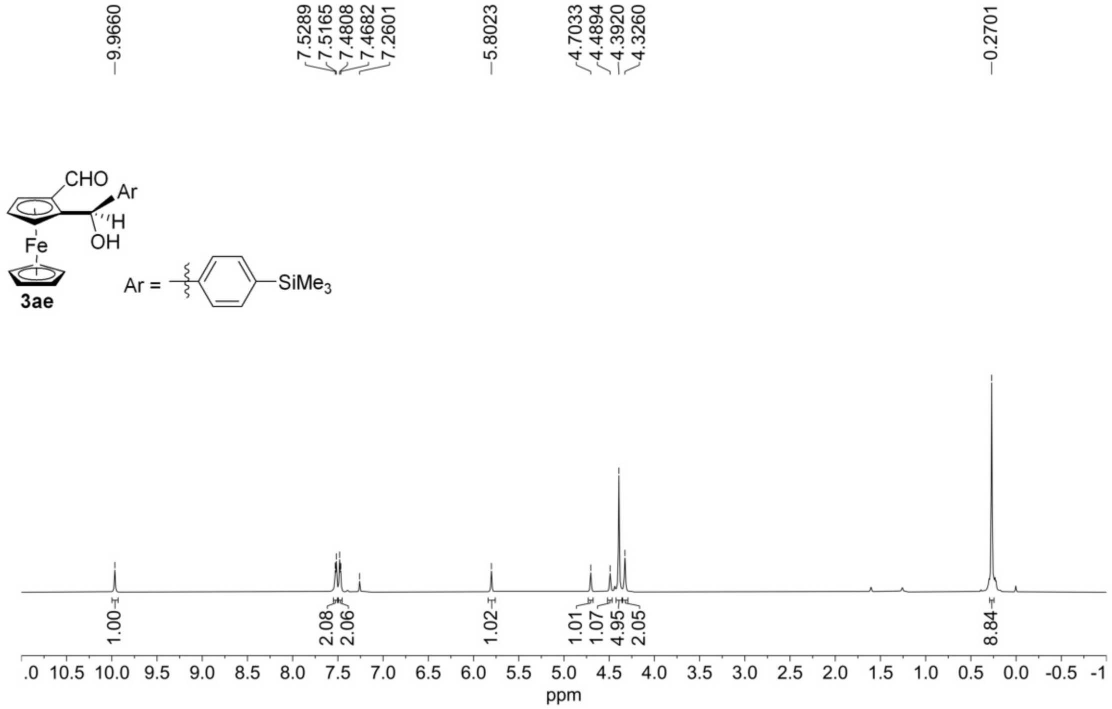

^{13}C NMR (150 MHz, $CDCl_3$)

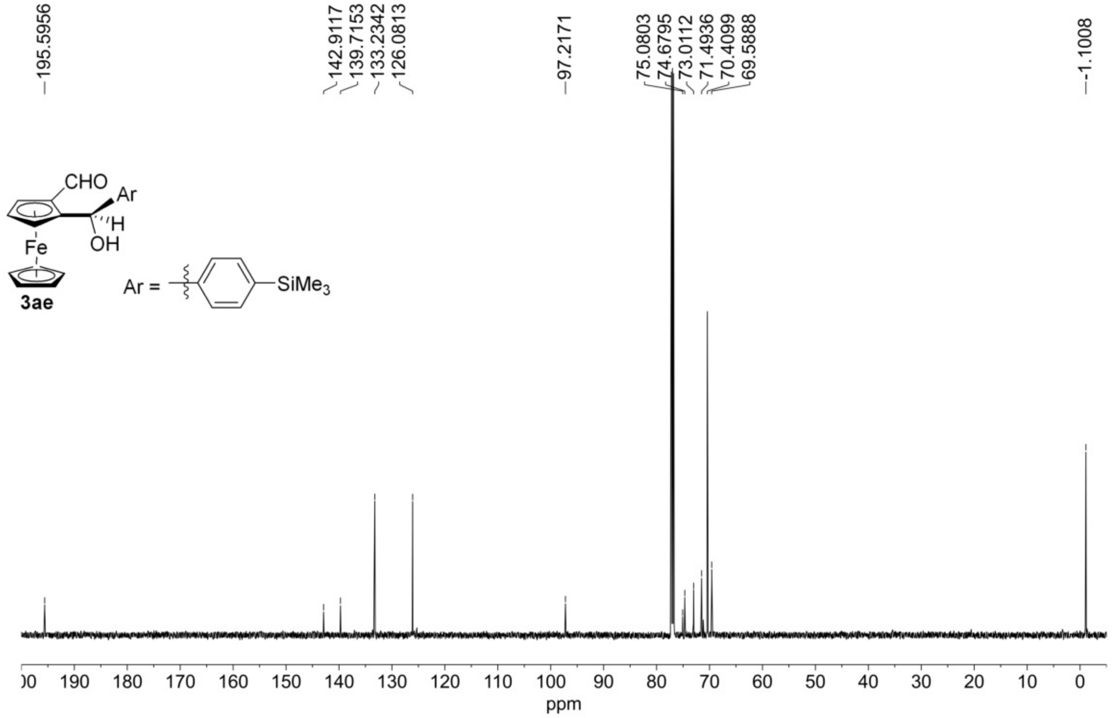

^{1}H NMR (600 MHz, $CDCl_3$)

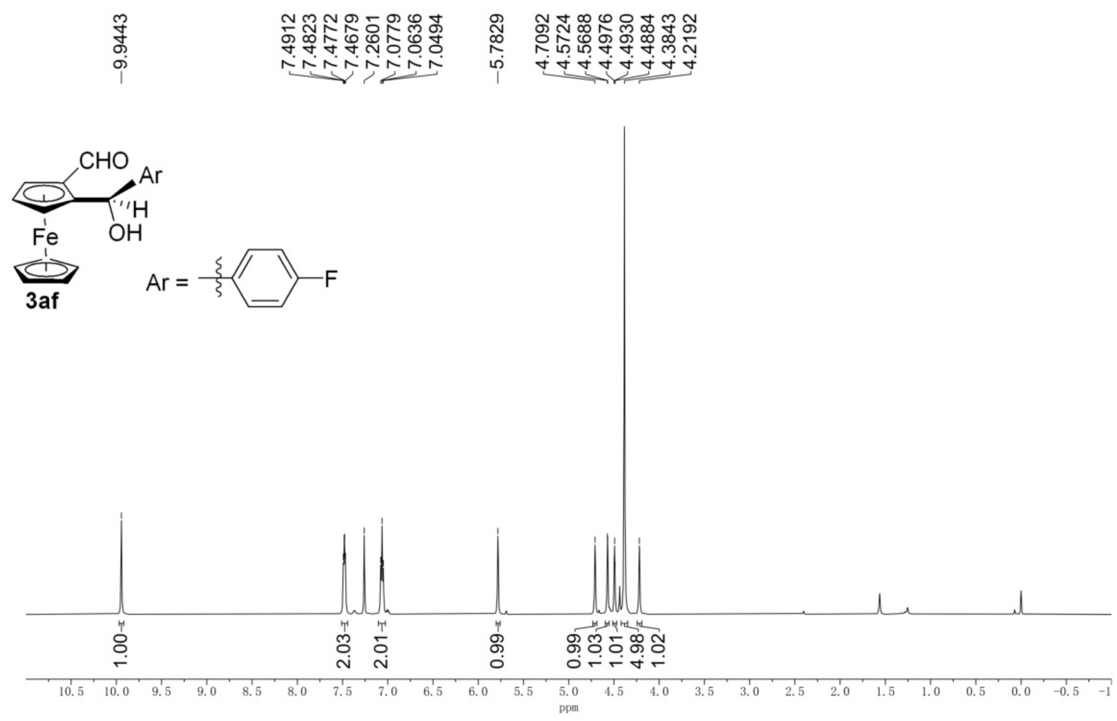

^{13}C NMR (150 MHz, $CDCl_3$)

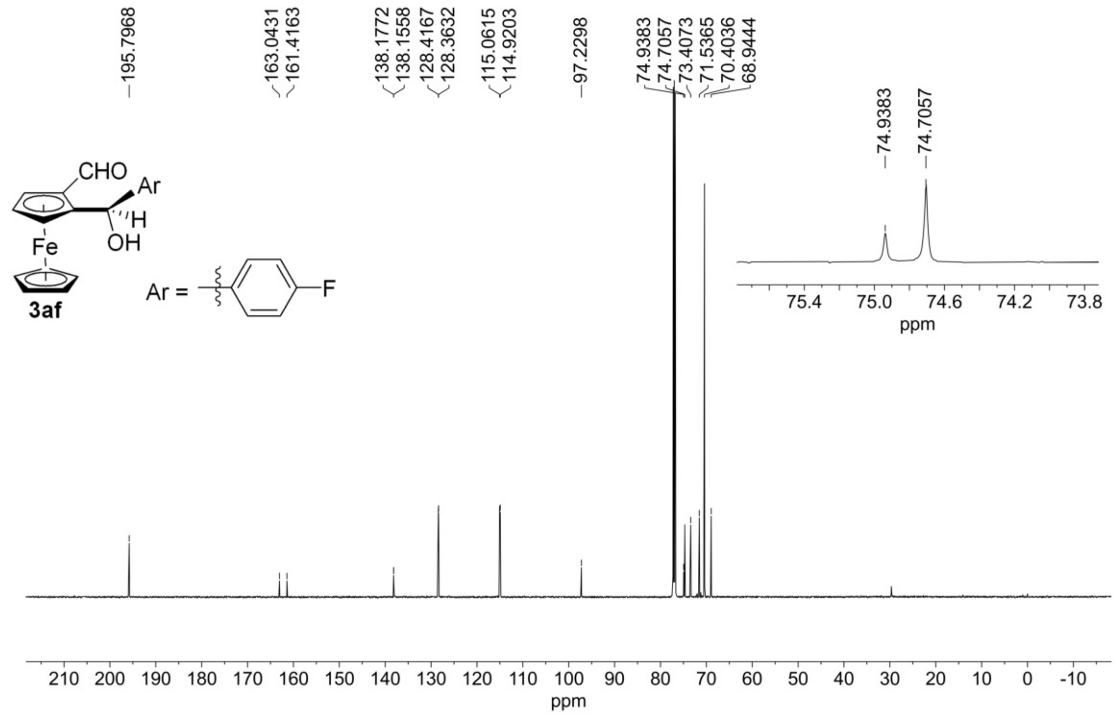

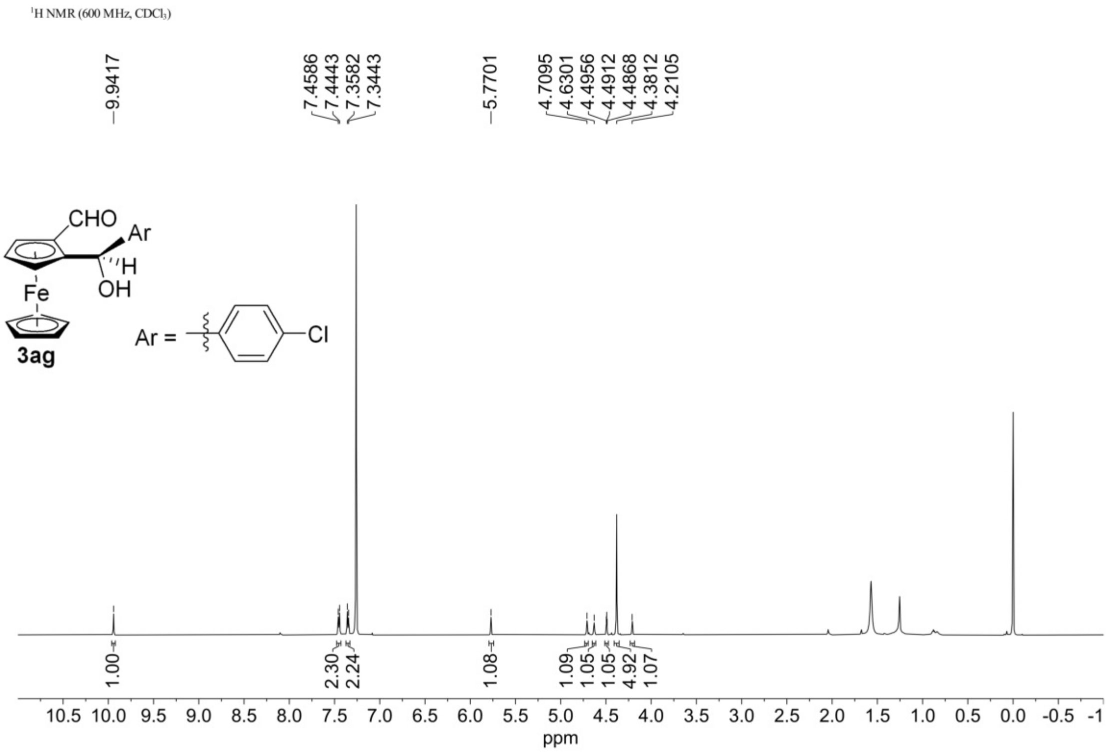

^{1}H NMR (600 MHz, CDCl3)
9.9417
7.4586
7.4443
7.3582
7.3443
5.7701
4.7095
4.6301
4.4956
4.4912
4.4868
4.3812
4.2105
CHO
Ar
H
OH
Fe
Ar =
Cl
3ag
1.00
2.30
2.24
1.08
1.09
1.05
1.05
4.92
1.07
10.5 10.0 9.5 9.0 8.5 8.0 7.5 7.0 6.5 6.0 5.5 5.0 4.5 4.0 3.5 3.0 2.5 2.0 1.5 1.0 0.5 0.0 -0.5 -1
ppm

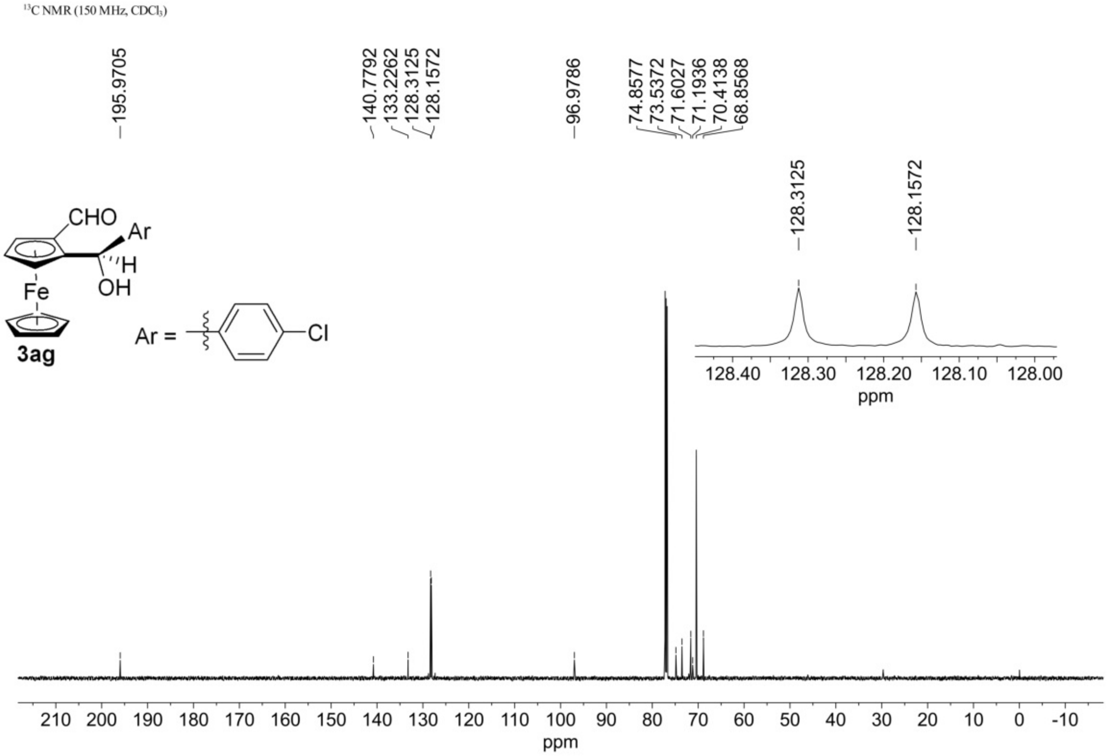

^{13}C NMR (150 MHz, CDCl3)
195.9705
140.7792
133.2262
128.3125
128.1572
96.9786
74.8577
73.5372
71.6027
71.1936
70.4138
68.8568
CHO
Ar
H
OH
Fe
Ar =
Cl
3ag
128.3125
128.1572
128.40 128.30 128.20 128.10 128.00
ppm
210 200 190 180 170 160 150 140 130 120 110 100 90 80 70 60 50 40 30 20 10 0 -10
ppm

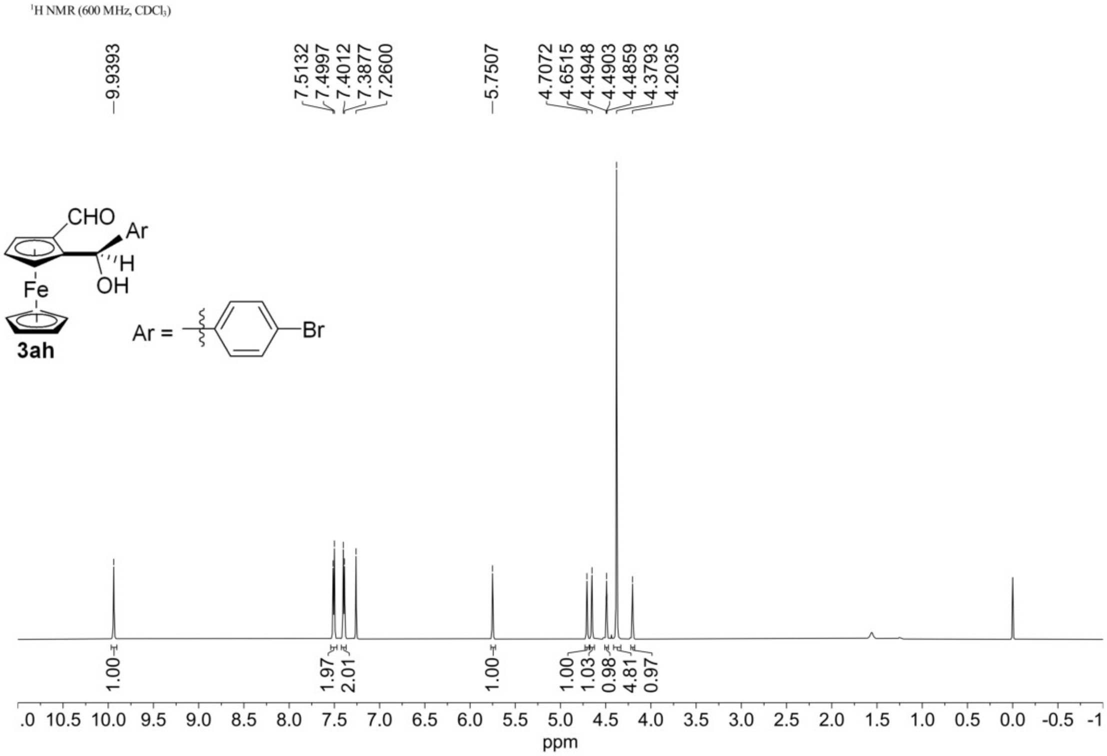
1H NMR (600 MHz, CDCl3)
9.9393
7.5132
7.4997
7.4012
7.3877
7.2600
5.7507
4.7072
4.6515
4.4948
4.4903
4.4859
4.3793
4.2035
CHO
Ar
H
OH
Fe
3ah
Ar =
Br
1.00
1.97
2.01
1.00
1.00
1.03
0.98
4.81
0.97
ppm

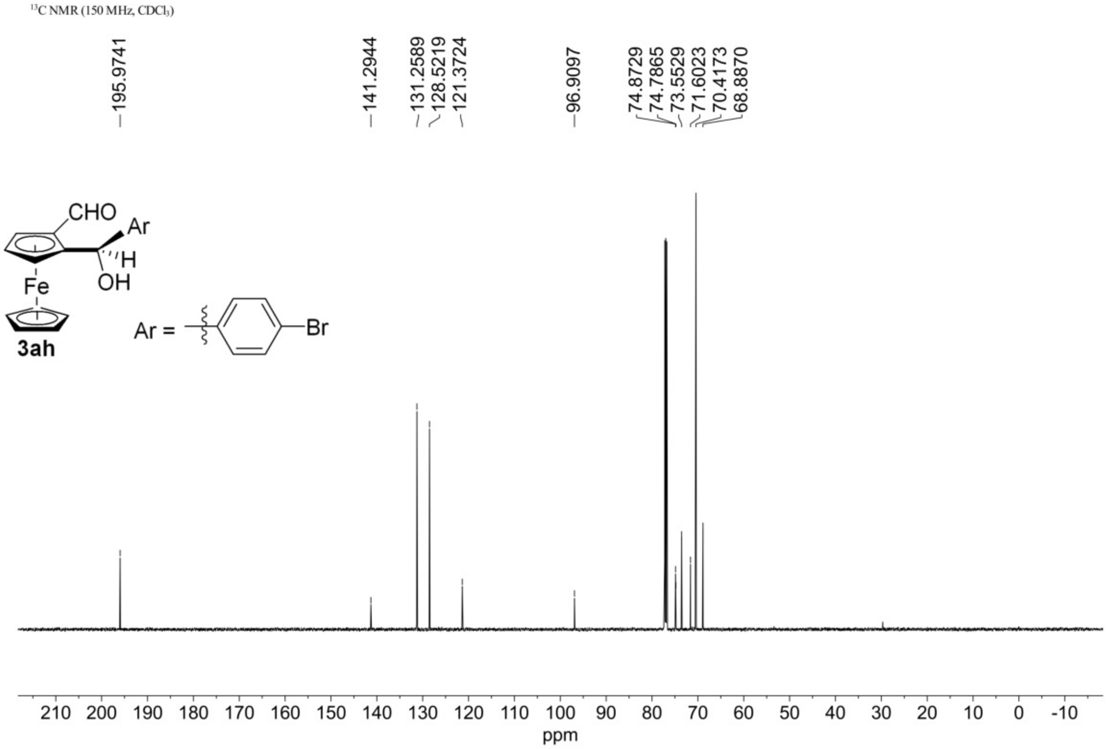
13C NMR (150 MHz, CDCl3)
195.9741
141.2944
131.2589
128.5219
121.3724
96.9097
74.8729
74.7865
73.5529
71.6023
70.4173
68.8870
CHO
Ar
H
OH
Fe
3ah
Ar =
Br
ppm

^{1}H NMR (600 MHz, $CDCl_3$)

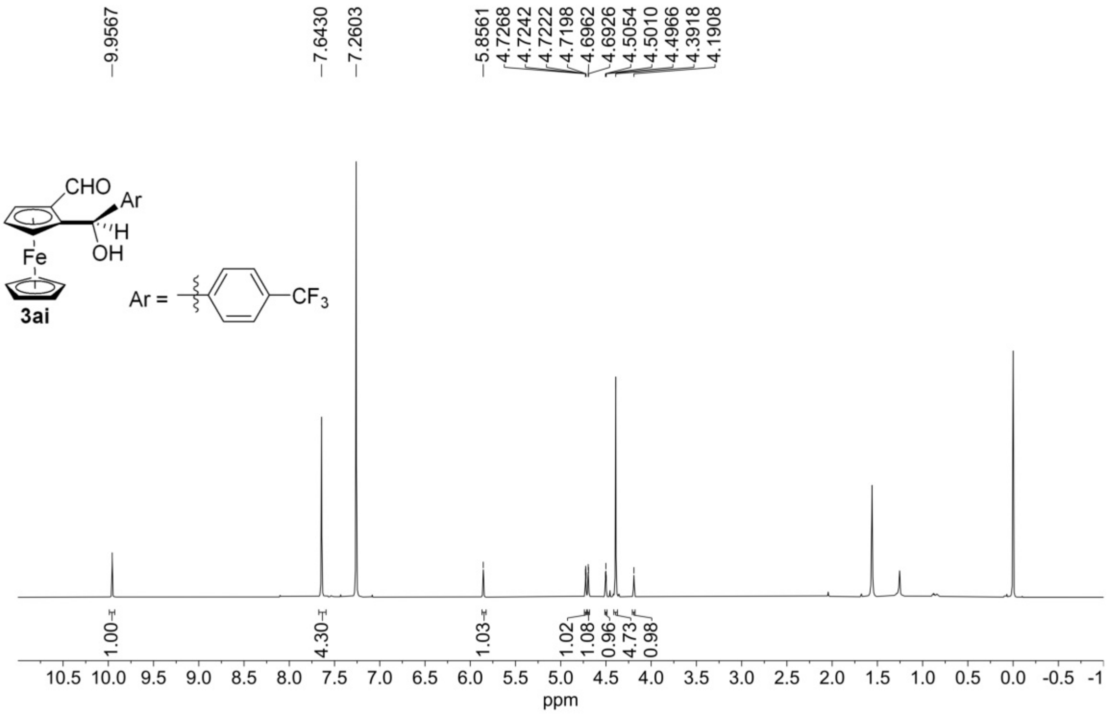

^{13}C NMR (150 MHz, $CDCl_3$)

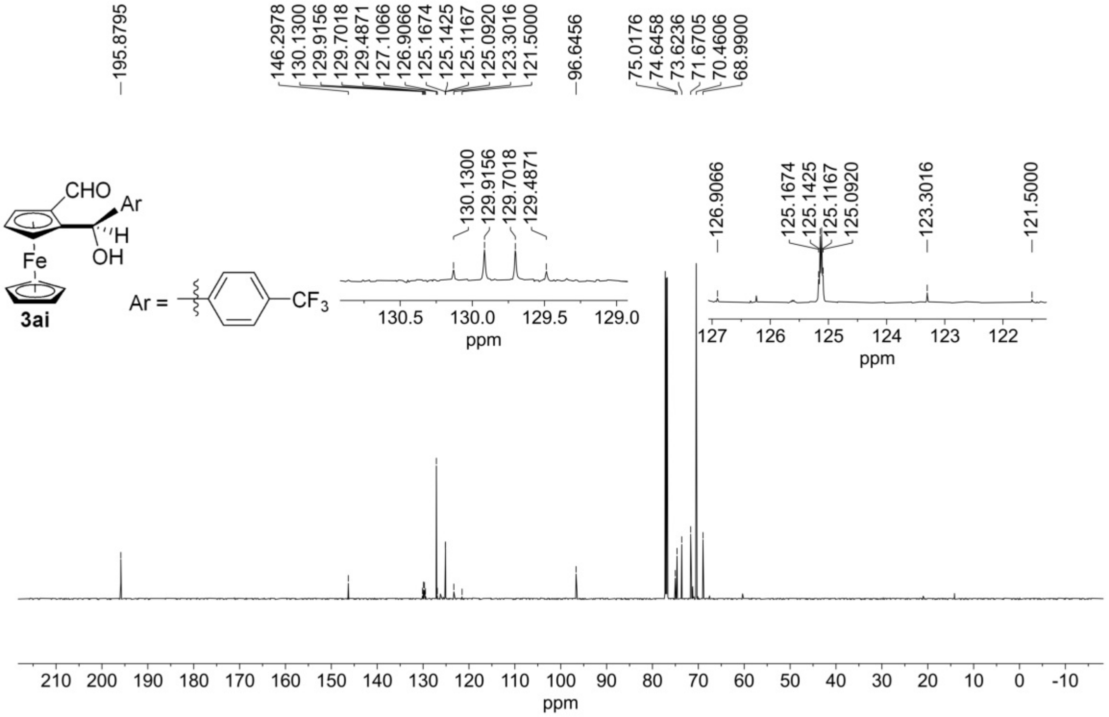

^1H NMR (600 MHz, $CDCl_3$)

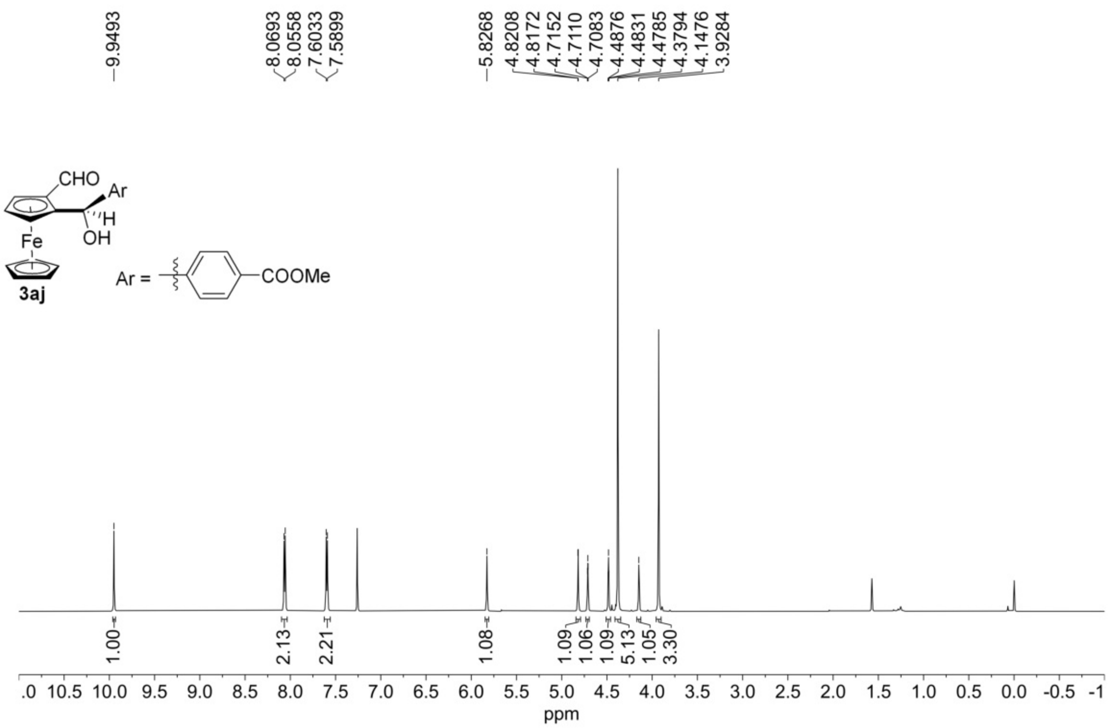

^{13}C NMR (150 MHz, $CDCl_3$)

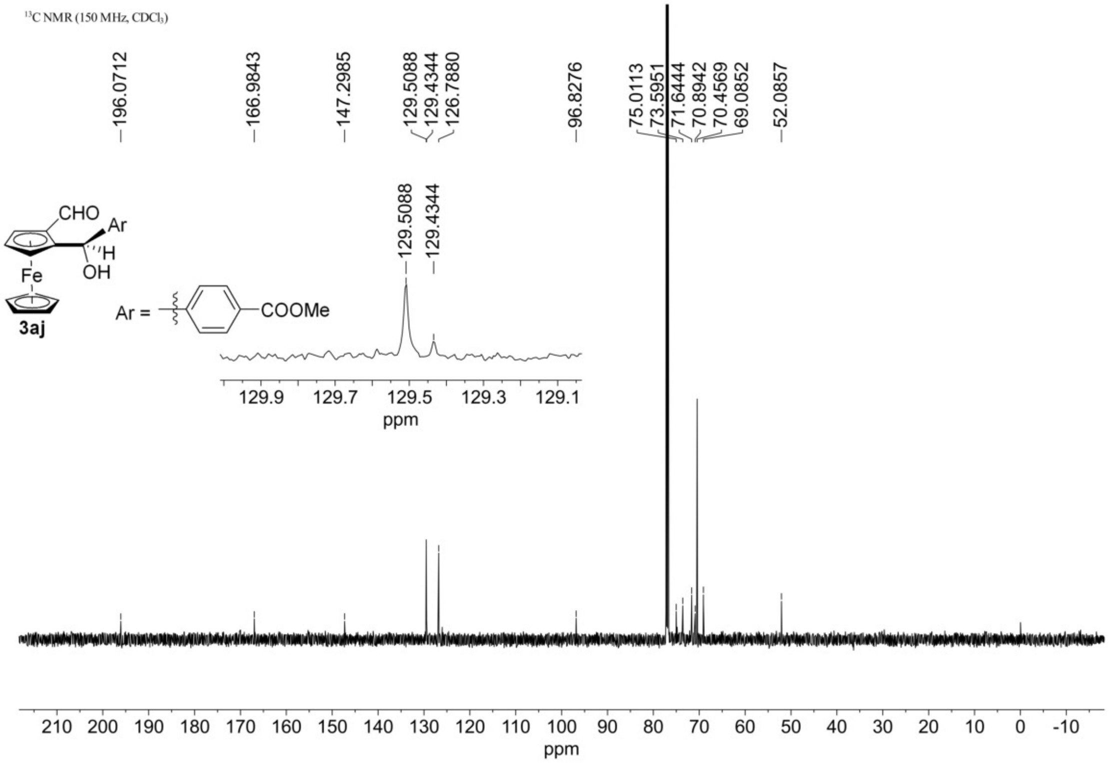

^{1}H NMR (600 MHz, $CDCl_3$)

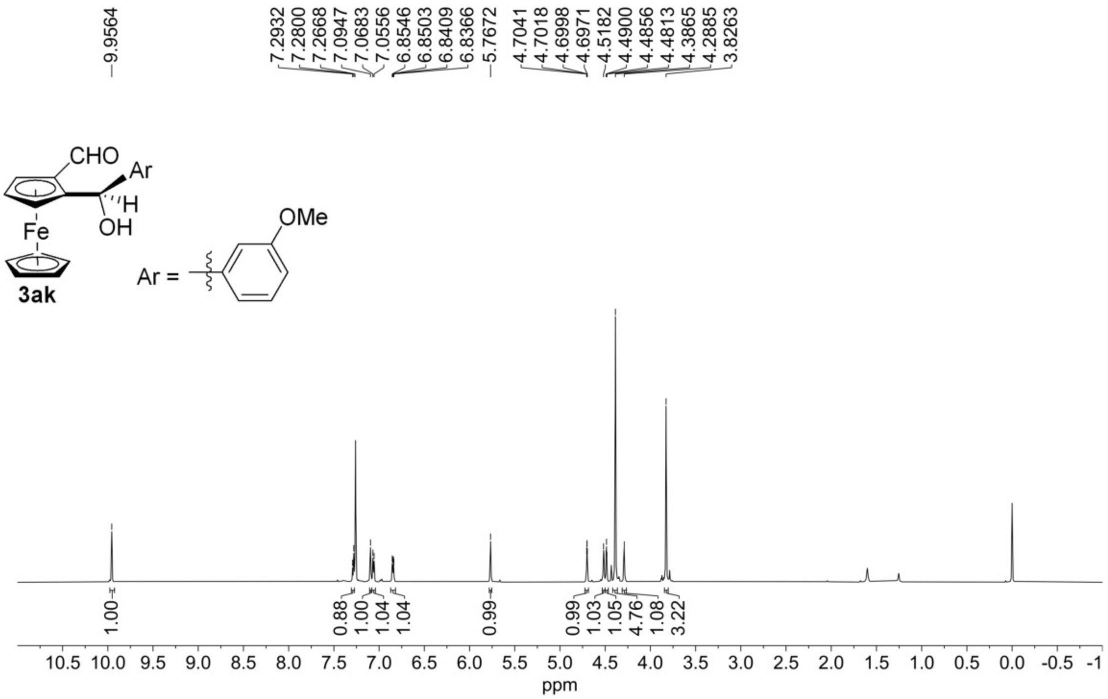

^{13}C NMR (150 MHz, $CDCl_3$)

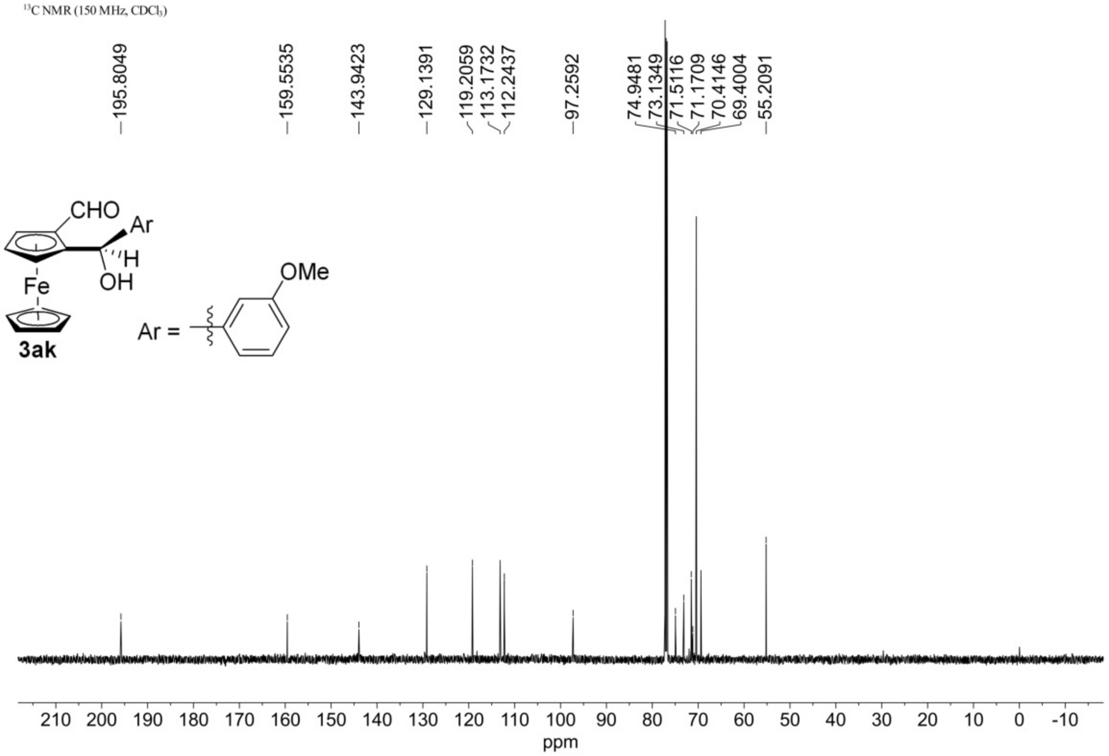

^{1}H NMR (600 MHz, CDCl$_3$)

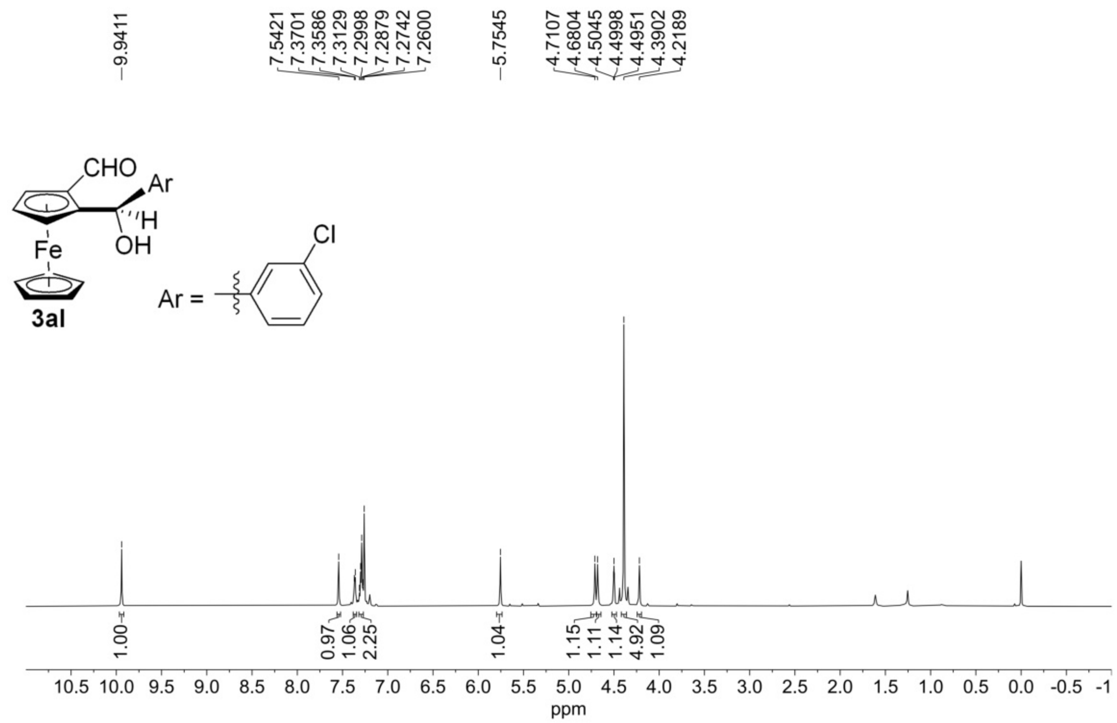

^{13}C NMR (150 MHz, CDCl$_3$)

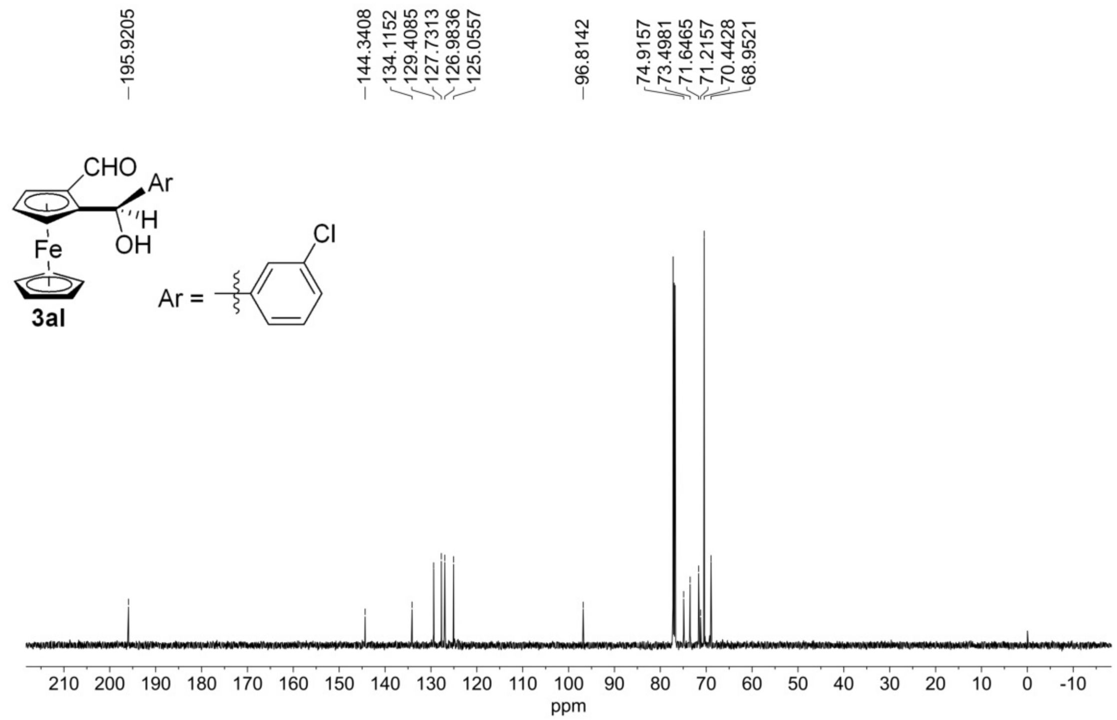

^{1}H NMR (600 MHz, $CDCl_3$)

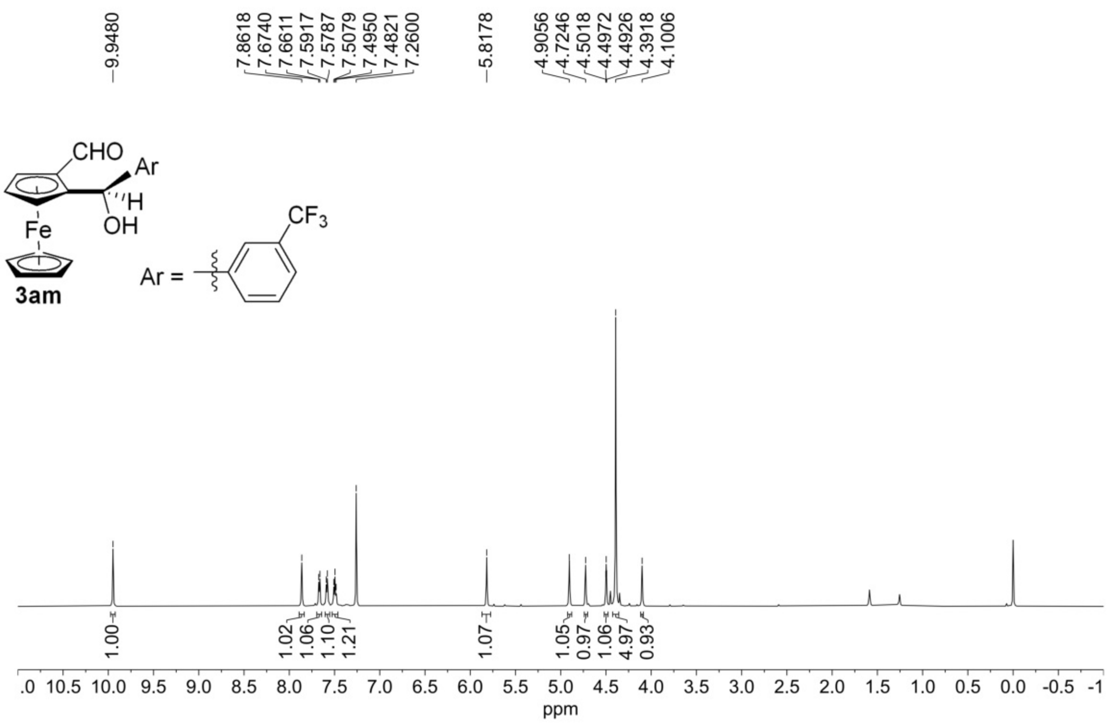

^{13}C NMR (150 MHz, $CDCl_3$)

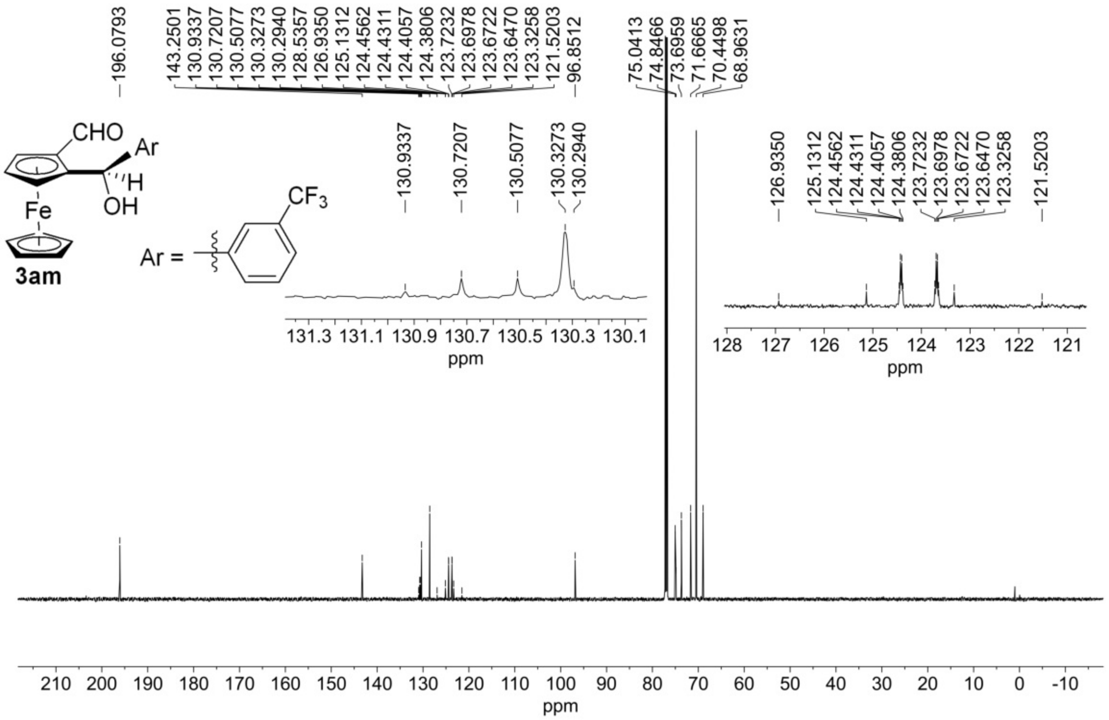

^{1}H NMR (600 MHz, $CDCl_3$)

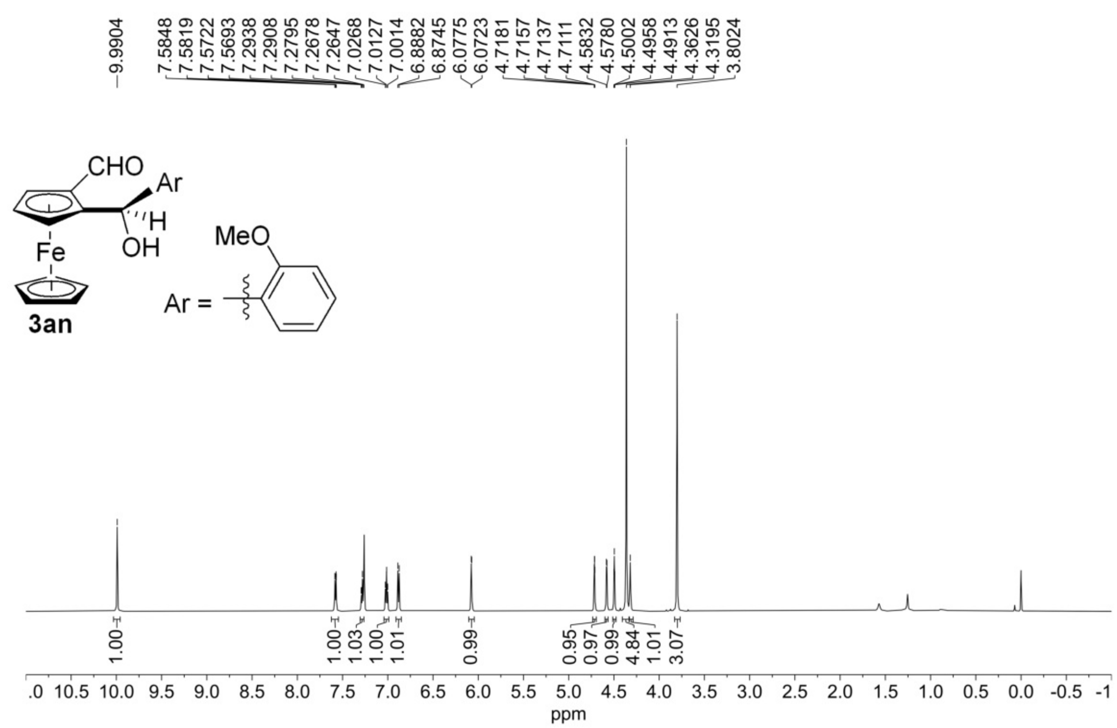

^{13}C NMR (150 MHz, $CDCl_3$)

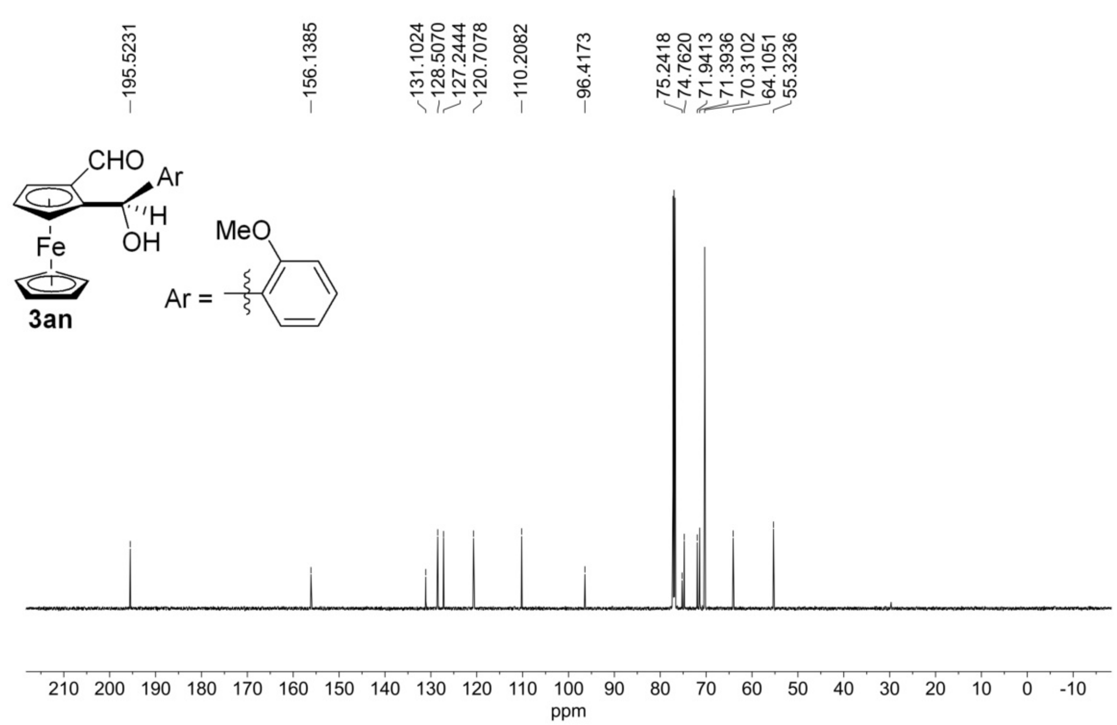

^{1}H NMR (600 MHz, CDCl$_3$)

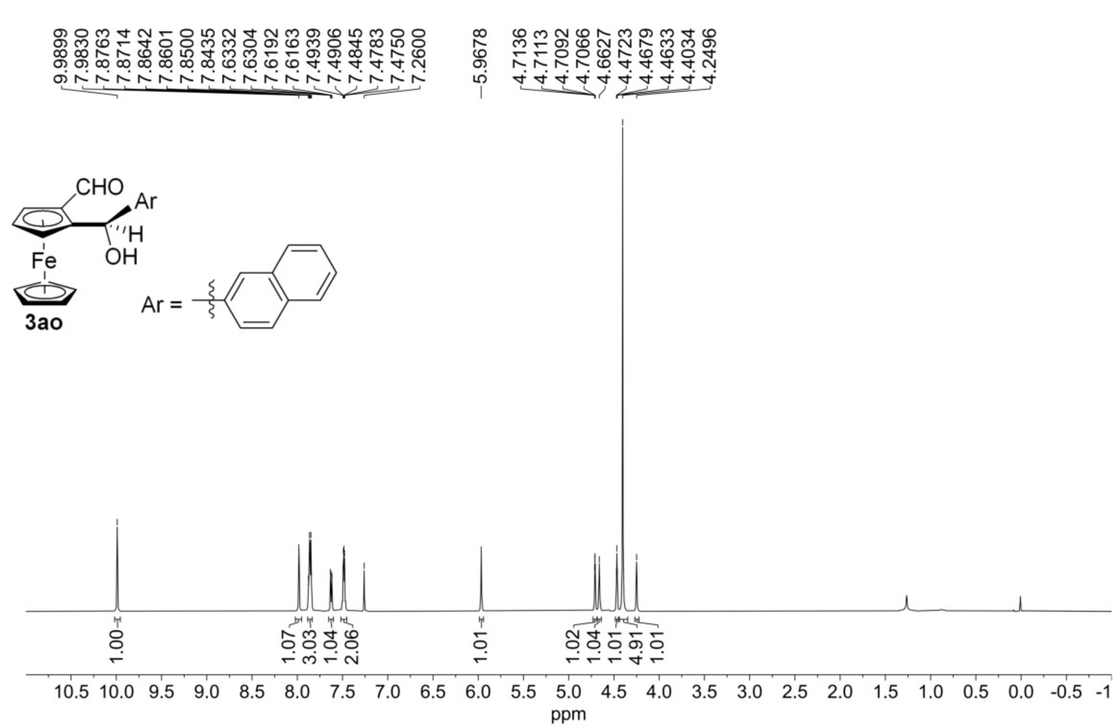

^{13}C NMR (150 MHz, CDCl$_3$)

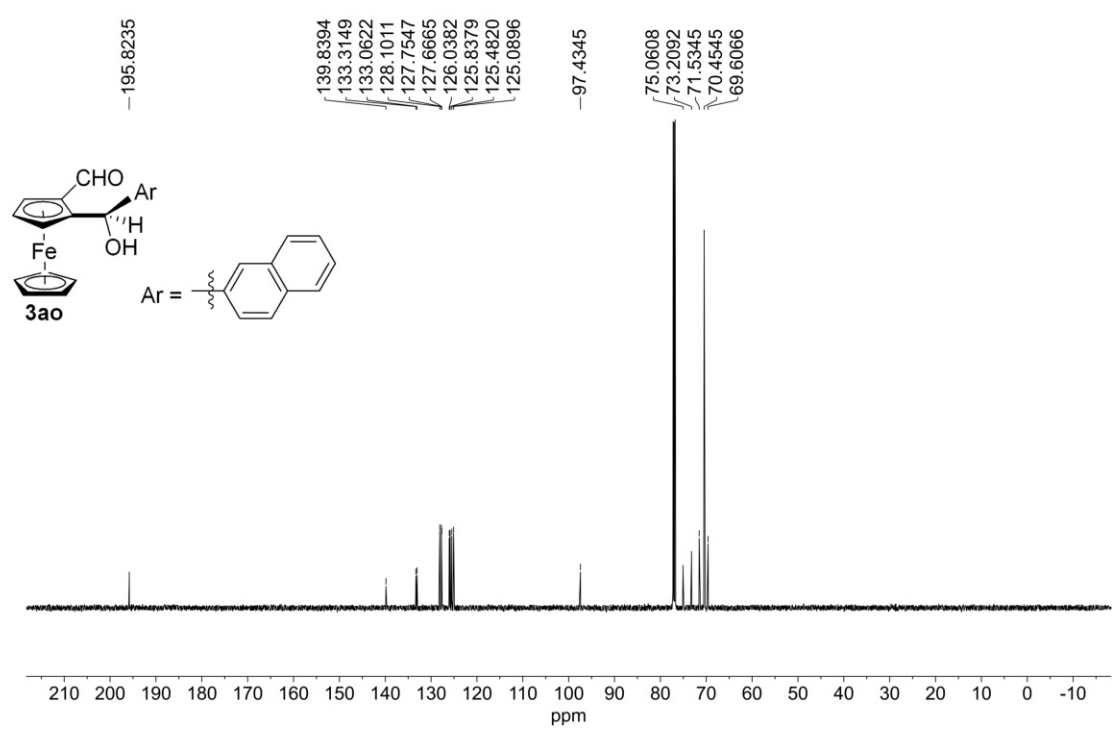

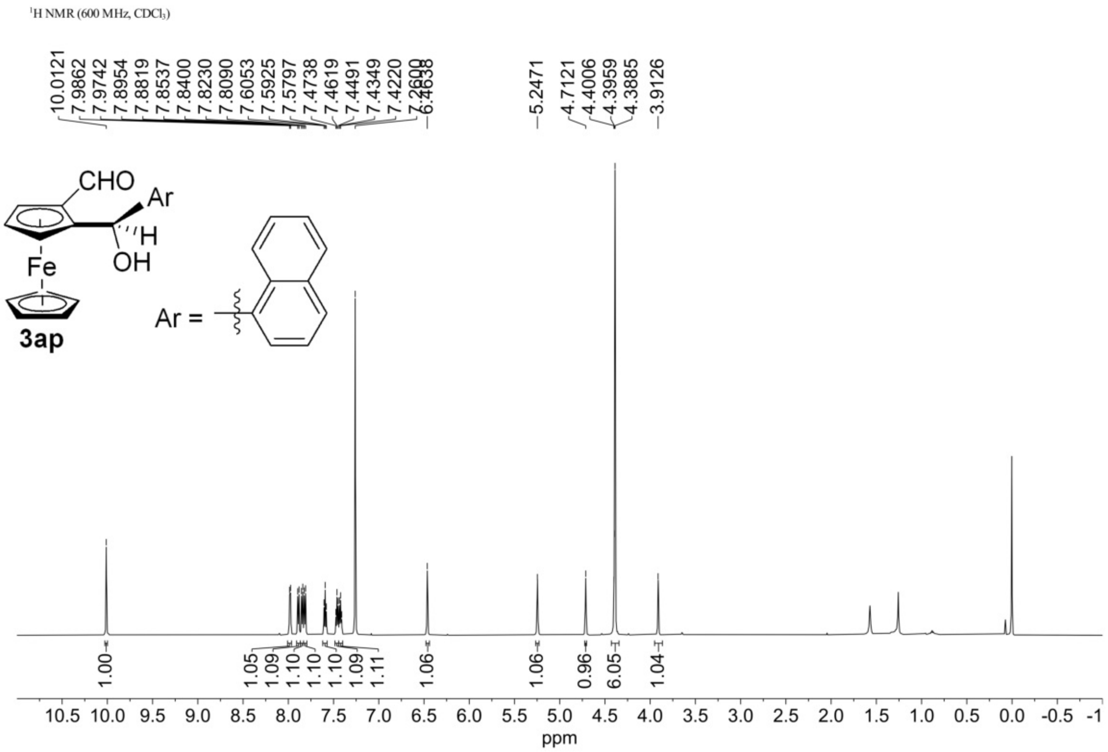

1H NMR (600 MHz, CDCl3)
CHO
Ar
H
OH
Fe
3ap
Ar =
ppm

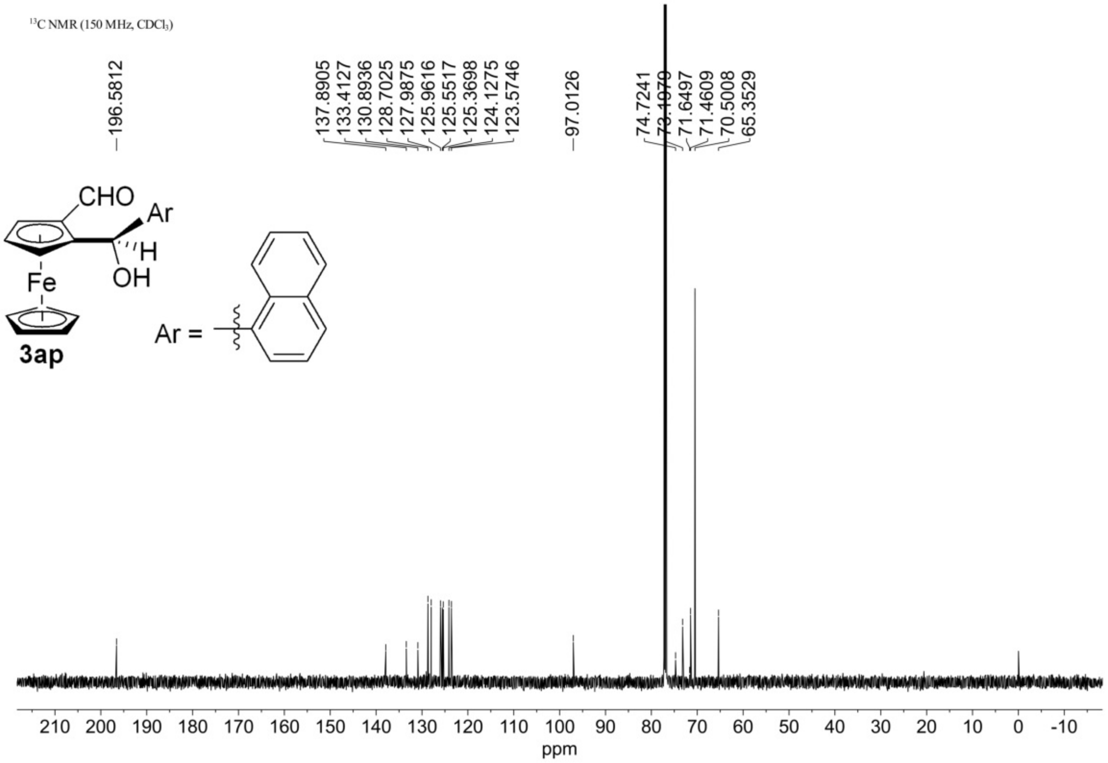

13C NMR (150 MHz, CDCl3)
CHO
Ar
H
OH
Fe
3ap
Ar =
ppm

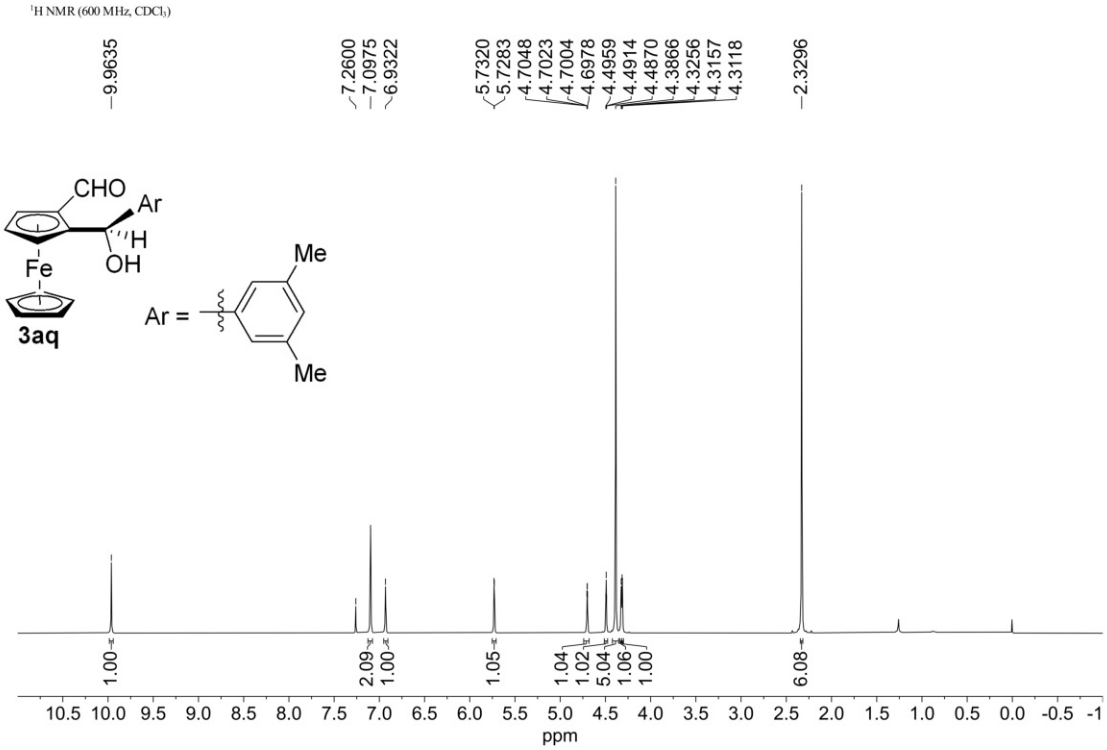

1H NMR (600 MHz, CDCl3)
9.9635
7.2600
7.0975
6.9322
5.7320
5.7283
4.7048
4.7023
4.7004
4.6978
4.4959
4.4914
4.4870
4.3866
4.3256
4.3157
4.3118
2.3296
CHO
Ar
H
OH
Fe
3aq
Ar =
Me
Me
1.00
2.09
1.00
1.05
1.04
1.02
5.04
1.06
1.00
6.08
10.5 10.0 9.5 9.0 8.5 8.0 7.5 7.0 6.5 6.0 5.5 5.0 4.5 4.0 3.5 3.0 2.5 2.0 1.5 1.0 0.5 0.0 -0.5 -1
ppm

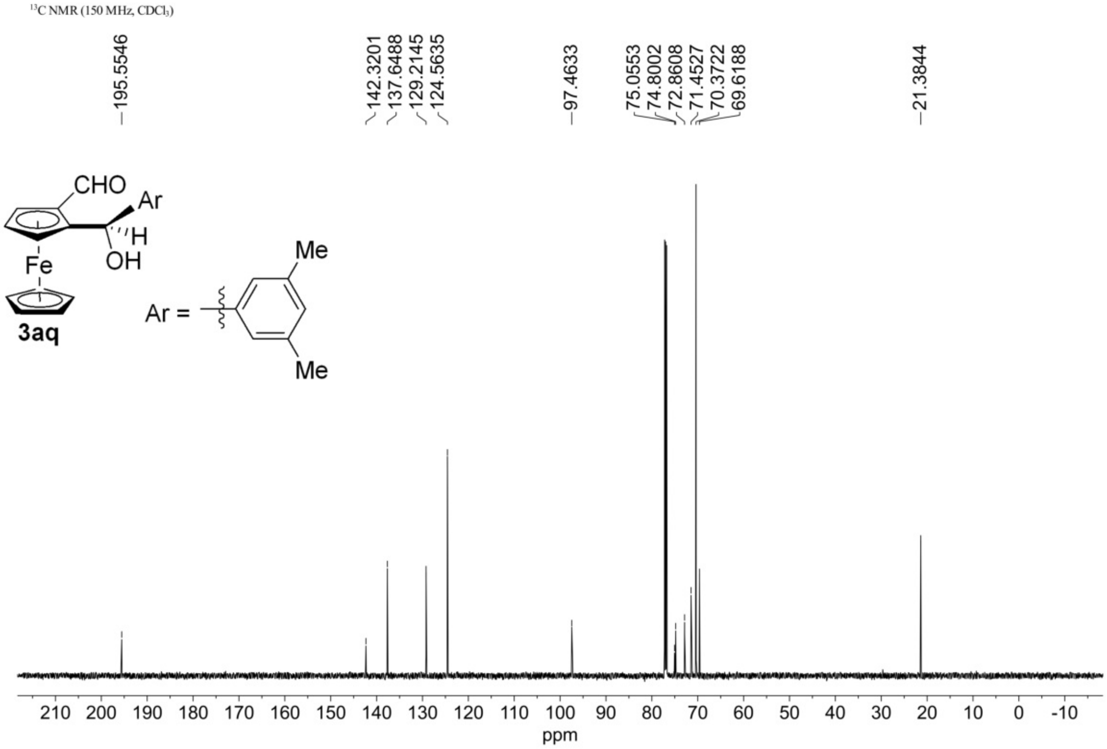

13C NMR (150 MHz, CDCl3)
195.5546
142.3201
137.6488
129.2145
124.5635
97.4633
75.0553
74.8002
72.8608
71.4527
70.3722
69.6188
21.3844
CHO
Ar
H
OH
Fe
3aq
Ar =
Me
Me
210 200 190 180 170 160 150 140 130 120 110 100 90 80 70 60 50 40 30 20 10 0 -10
ppm

^{1}H NMR (600 MHz, $CDCl_3$)

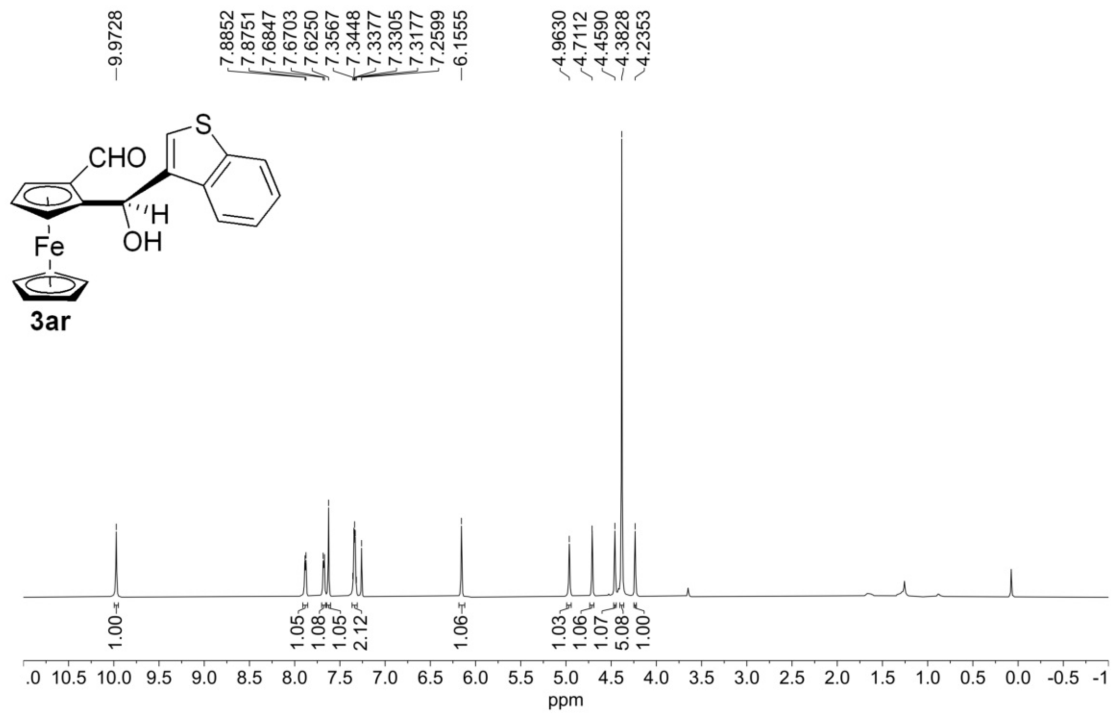

^{13}C NMR (150 MHz, $CDCl_3$)

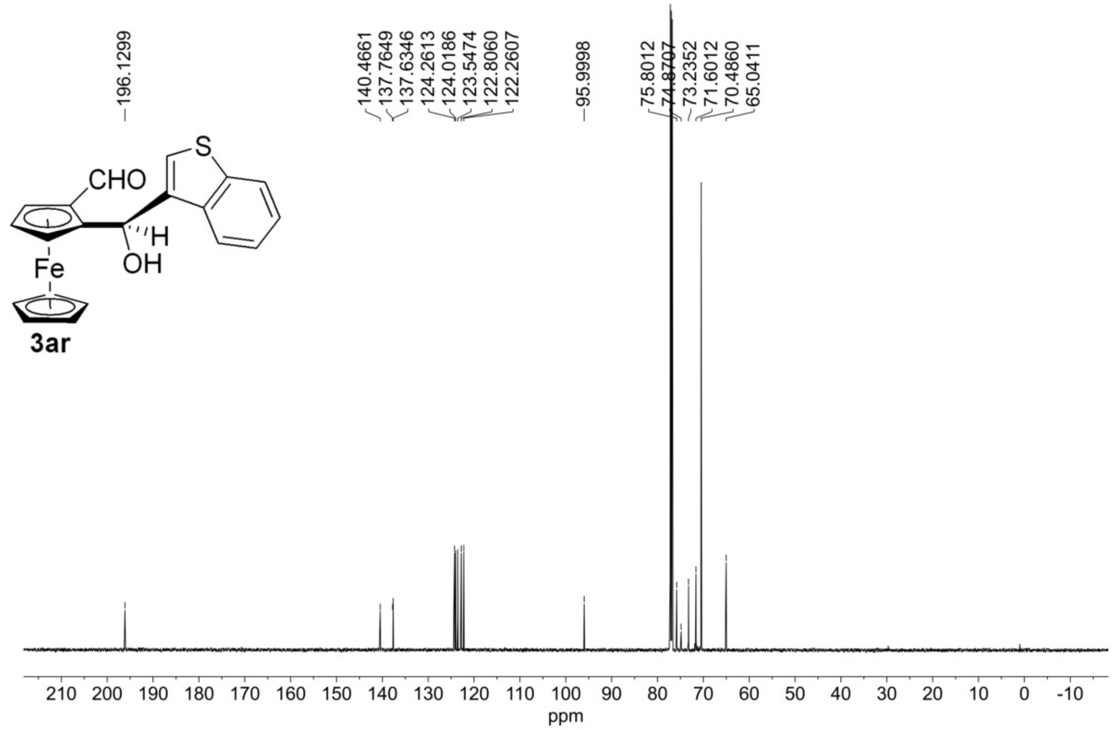

^{1}H NMR (600 MHz, $CDCl_3$)

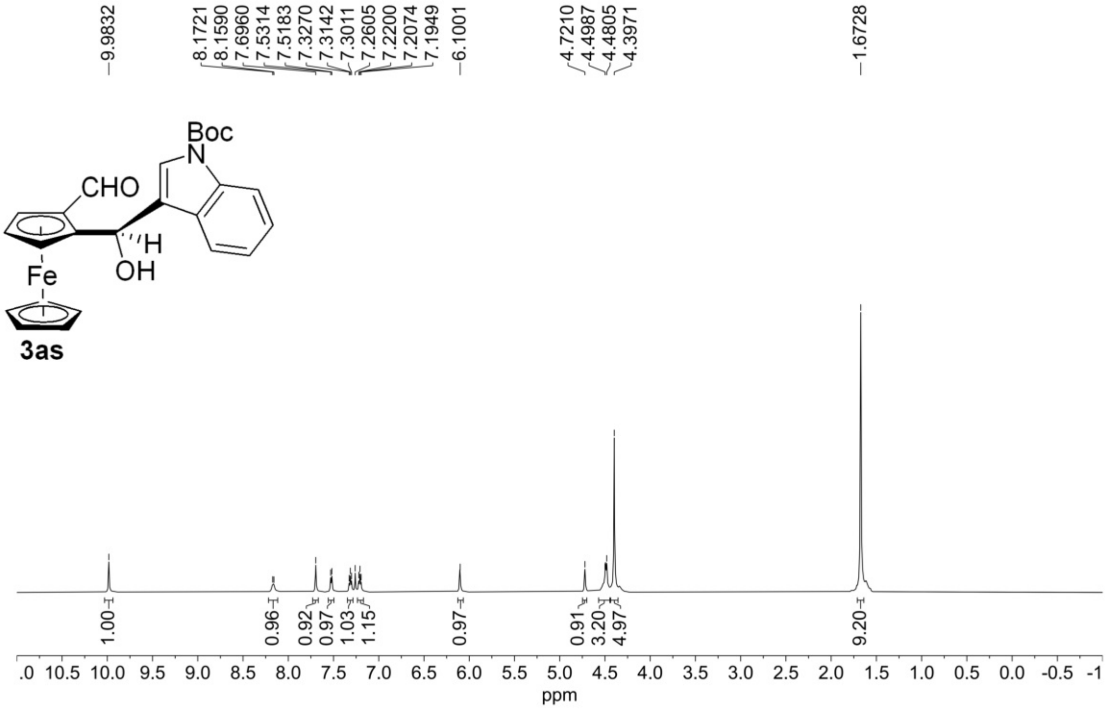

^{13}C NMR (150 MHz, $CDCl_3$)

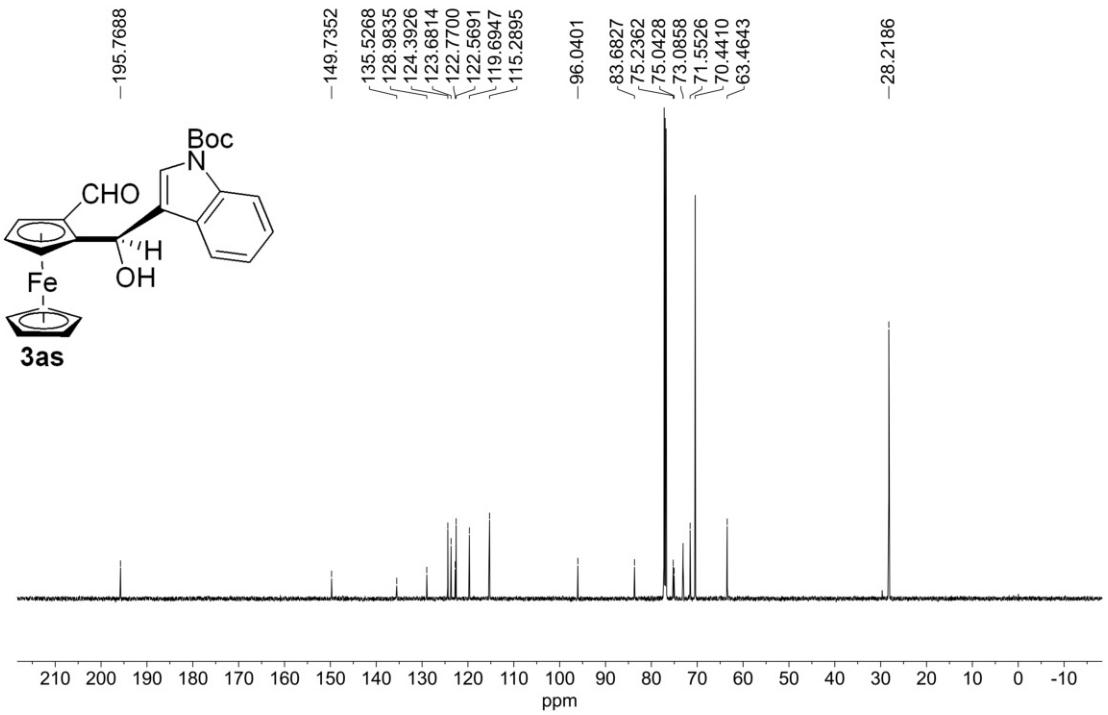

^{1}H NMR (600 MHz, $CDCl_3$)

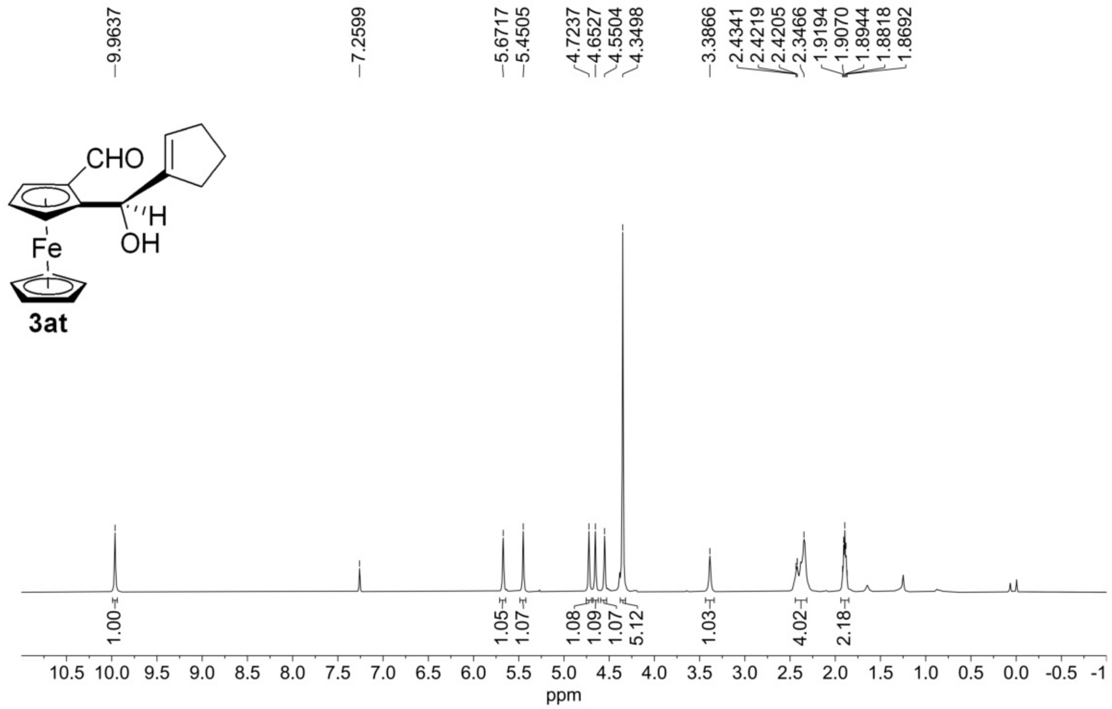

^{13}C NMR (150 MHz, $CDCl_3$)

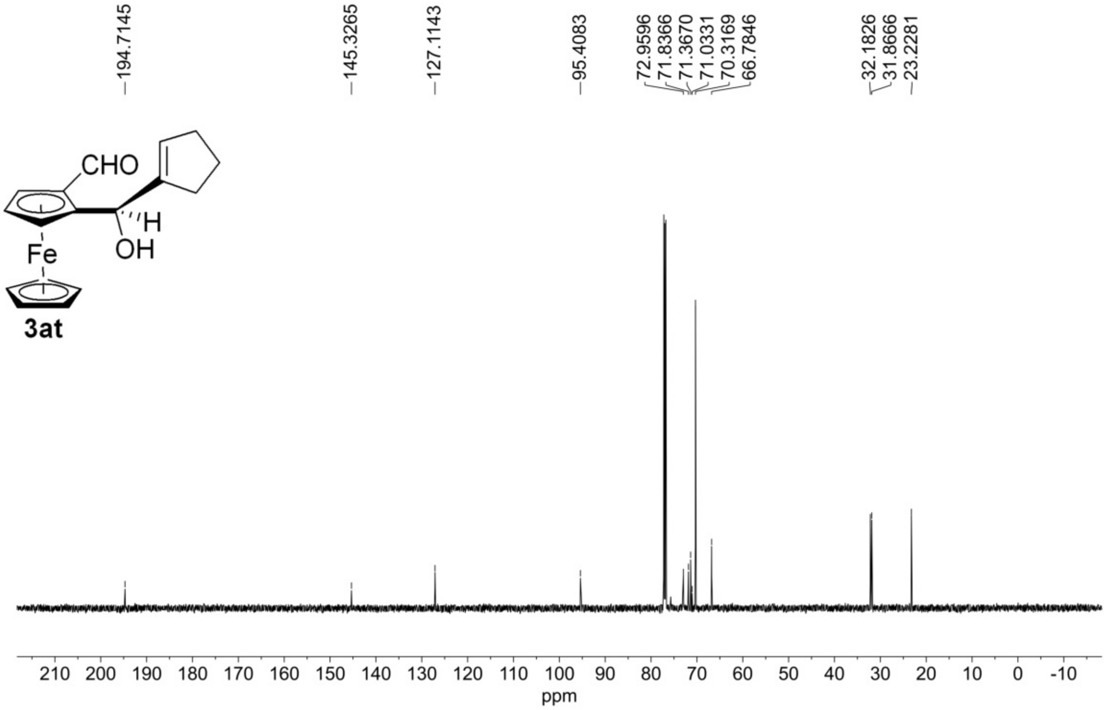

^{1}H NMR (600 MHz, $CDCl_3$)

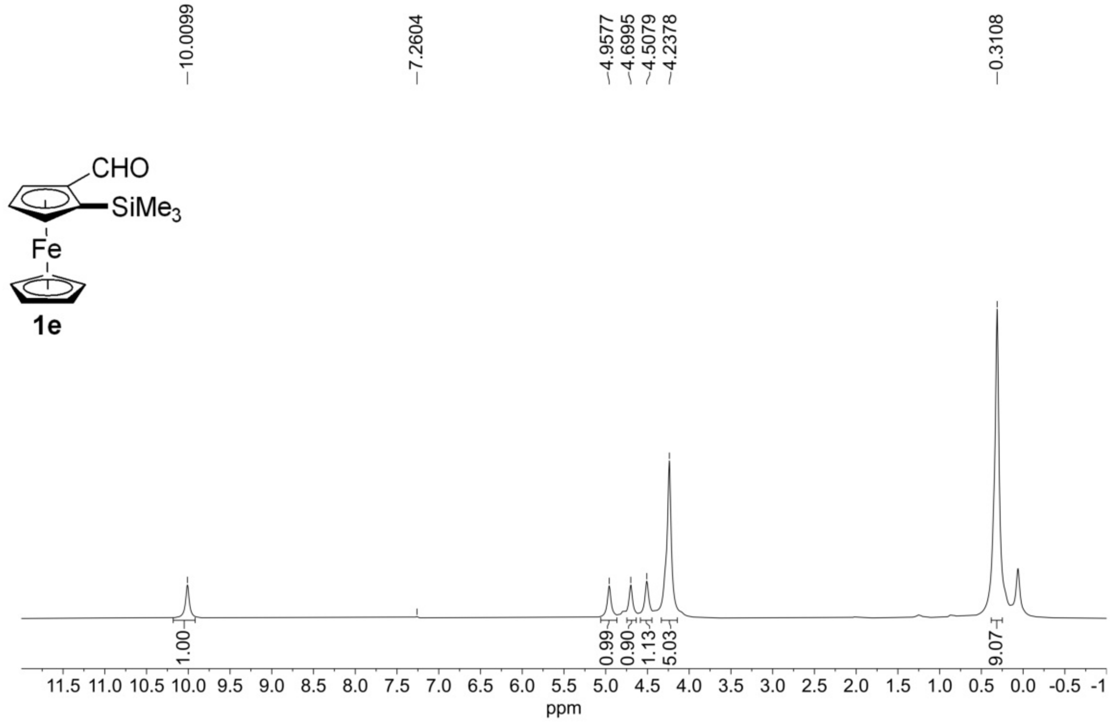

^{1}H NMR (600 MHz, $CDCl_3$)

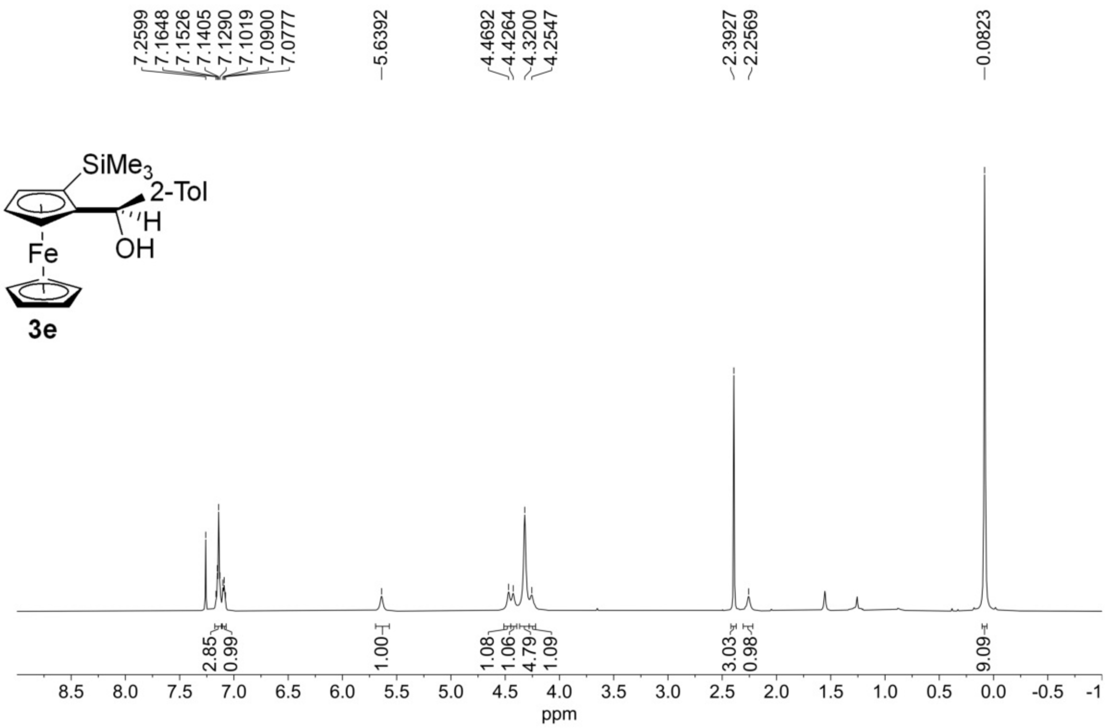

^{13}C NMR (150 MHz, $CDCl_3$)

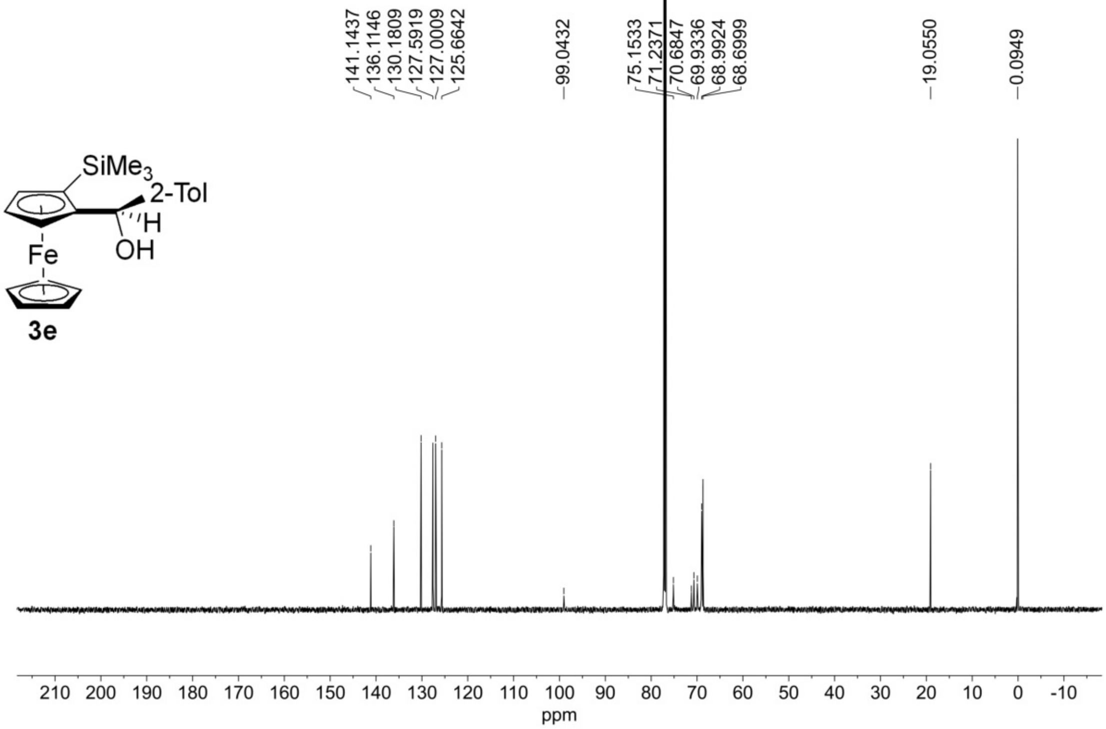

^{1}H NMR (600 MHz, $CDCl_3$)

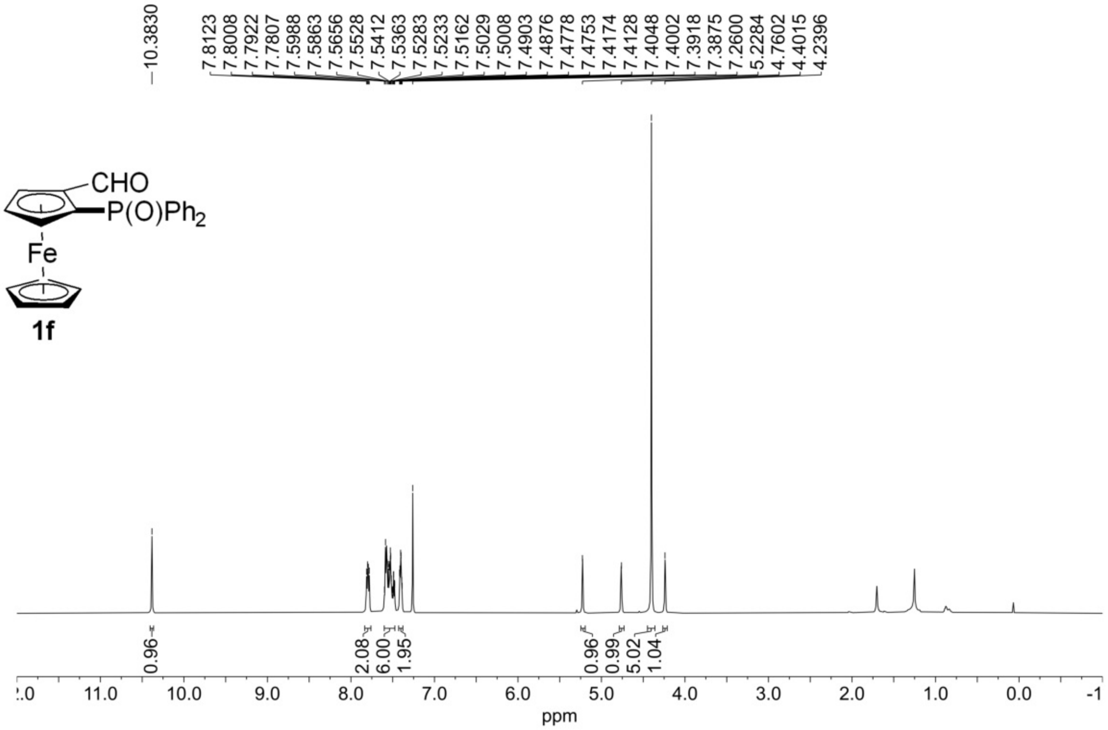

^{31}P NMR (243 MHz, $CDCl_3$)

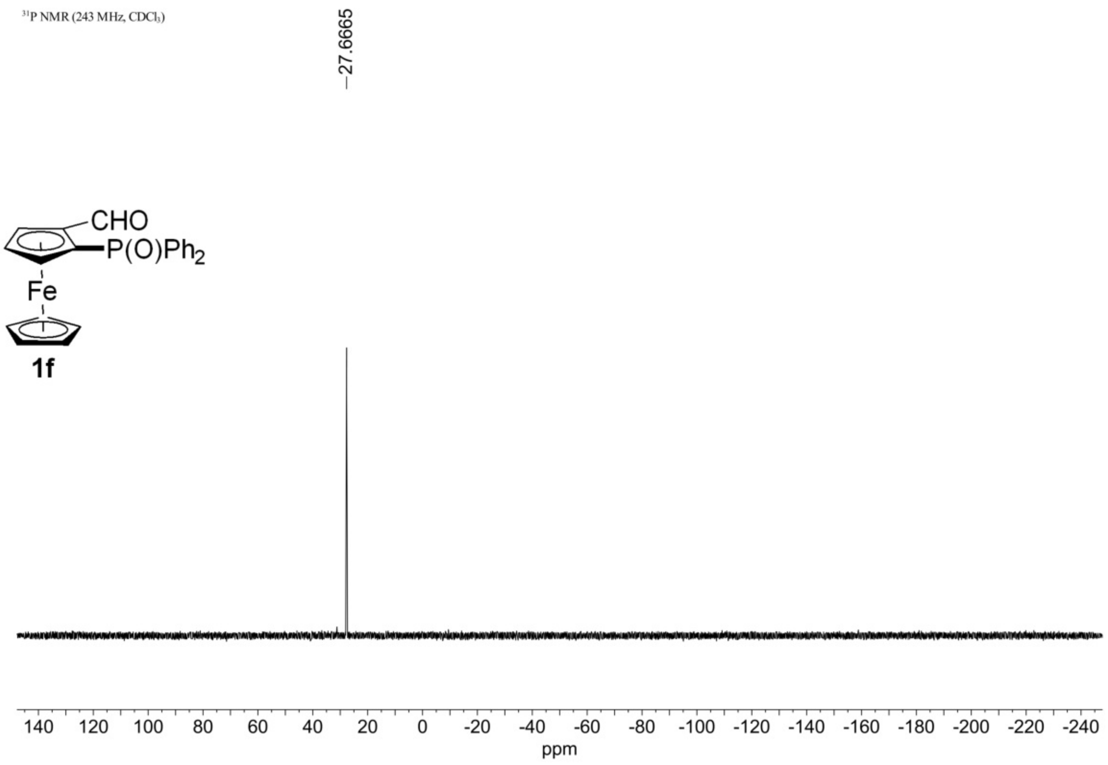

^{1}H NMR (600 MHz, $CDCl_3$)

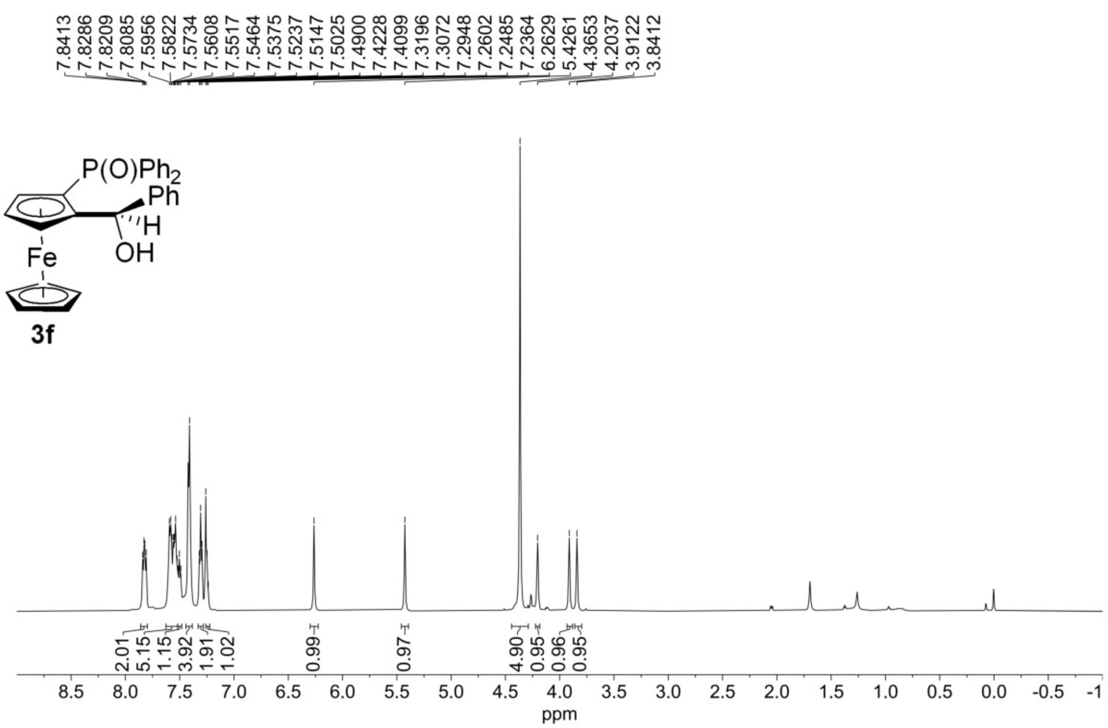

^{13}C NMR (150 MHz, $CDCl_3$)

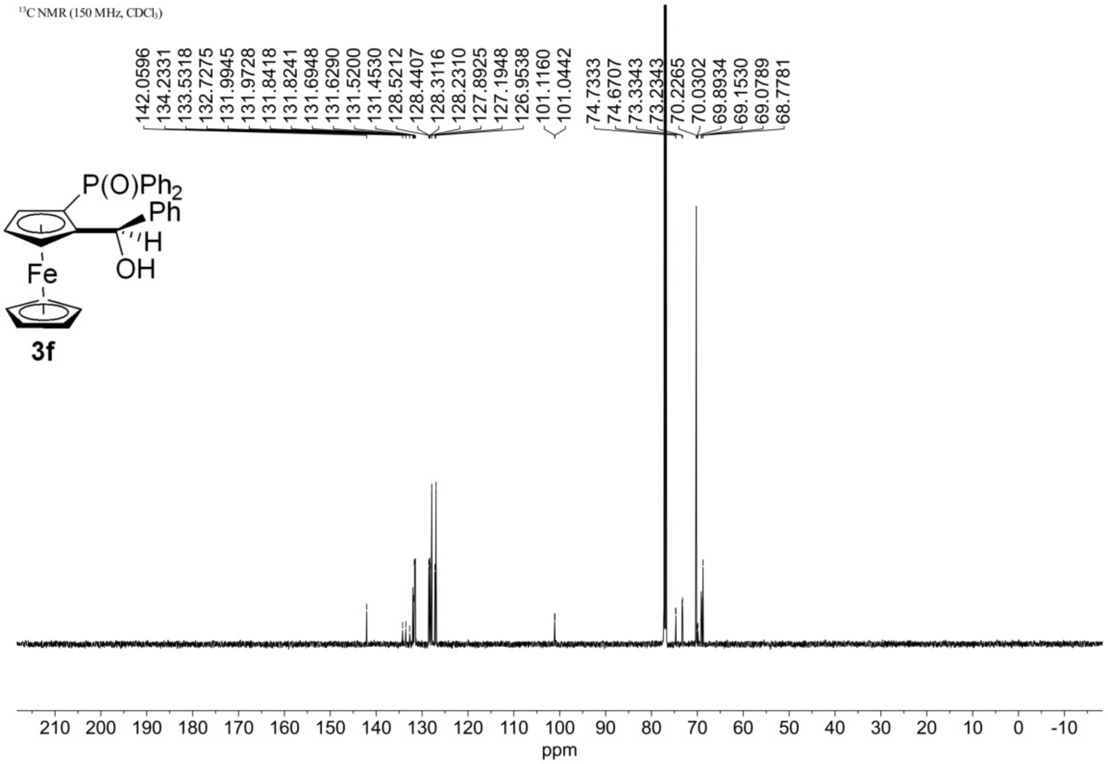

^{31}P NMR (243 MHz, CDCl$_3$)

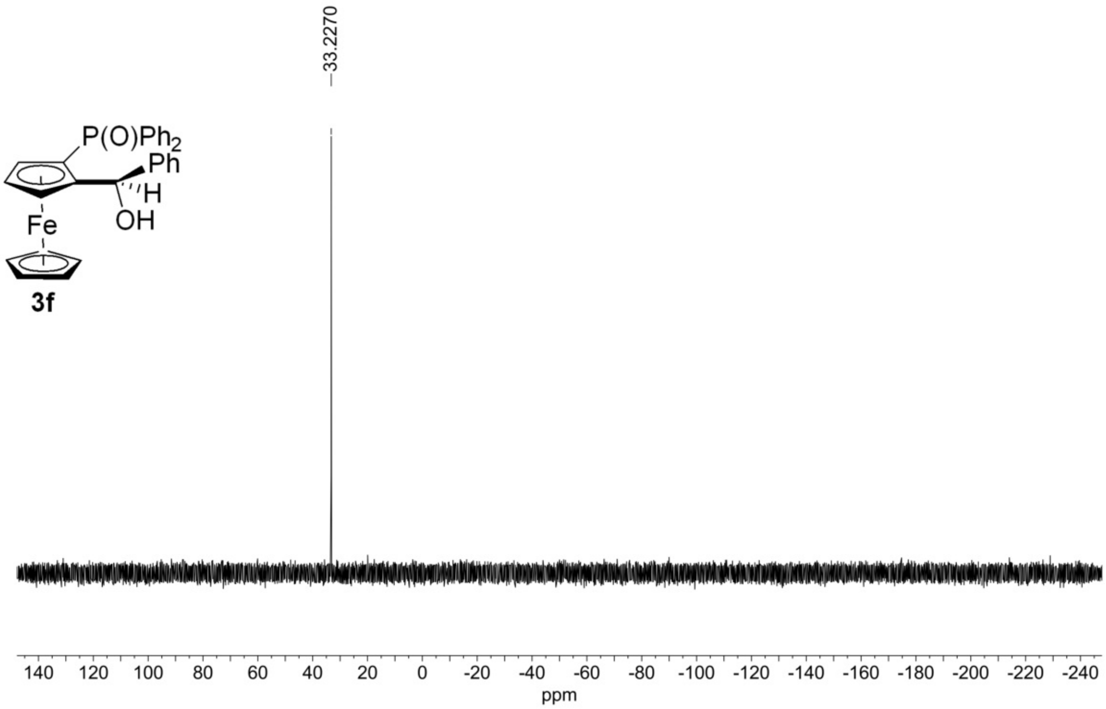

3f

^{1}H NMR (600 MHz, CDCl$_3$)

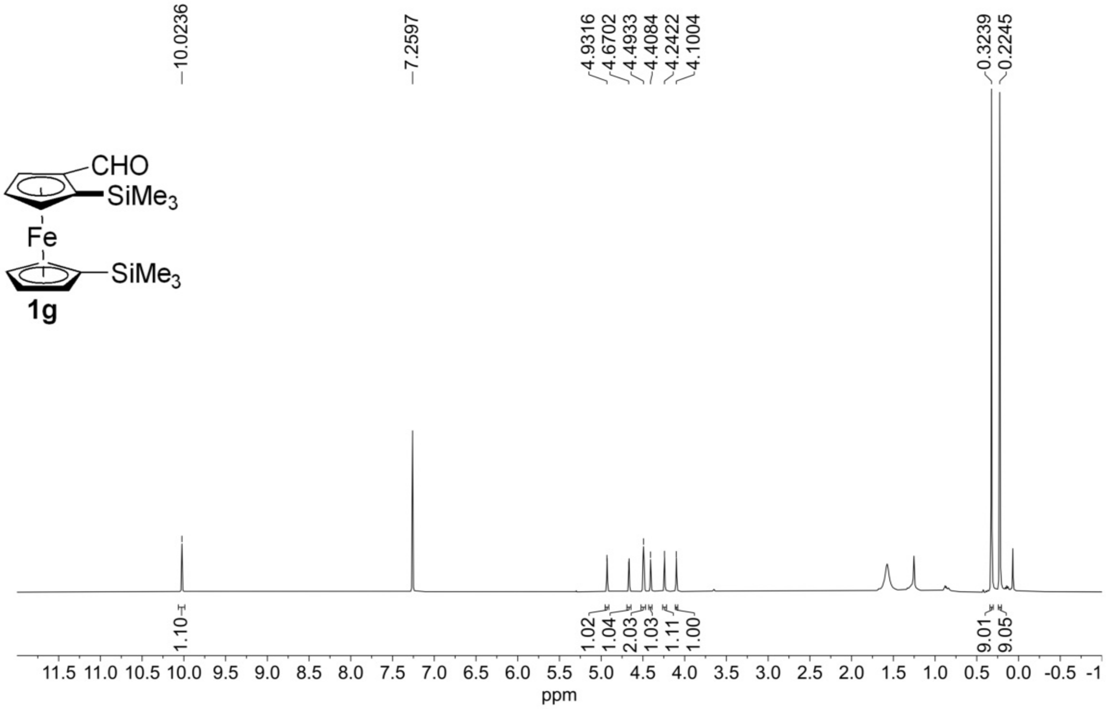

1g

^{1}H NMR (600 MHz, $CDCl_3$)

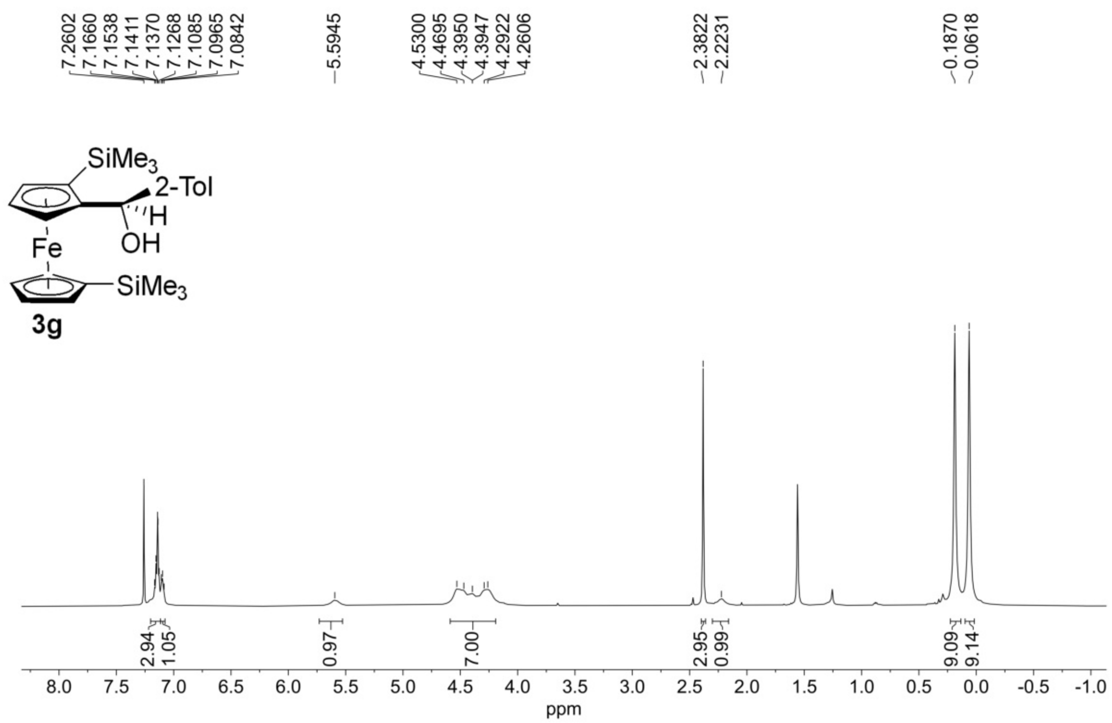

^{13}C NMR (150 MHz, $CDCl_3$)

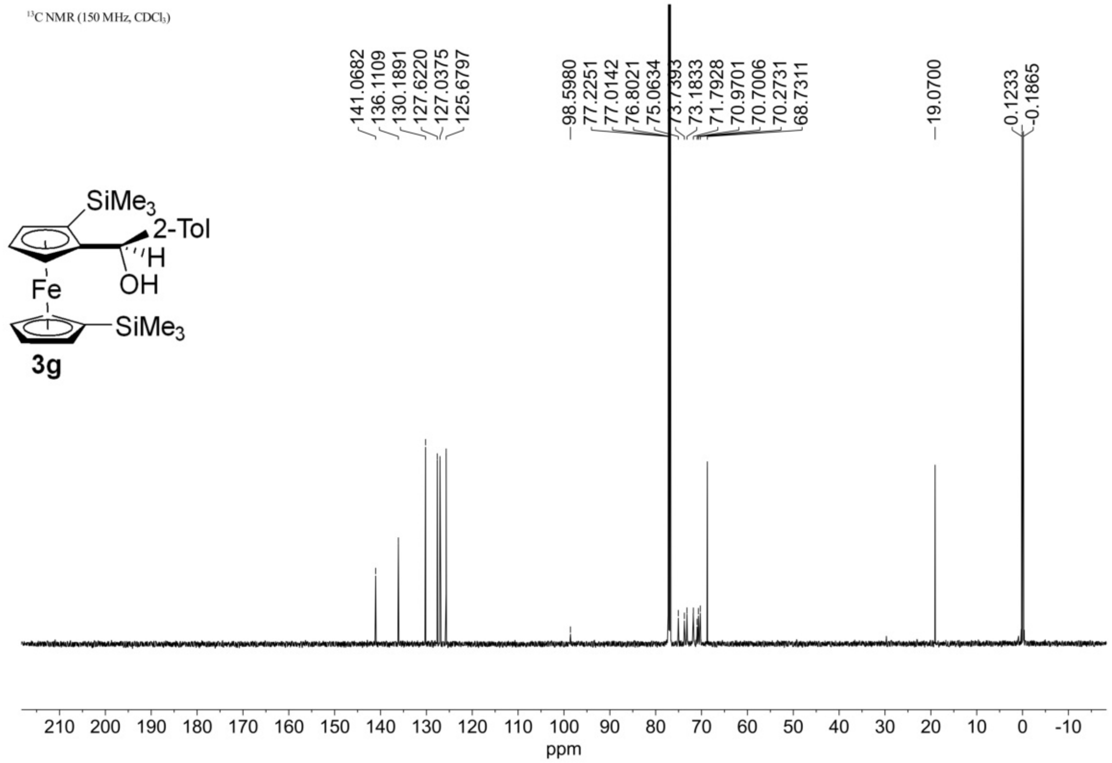

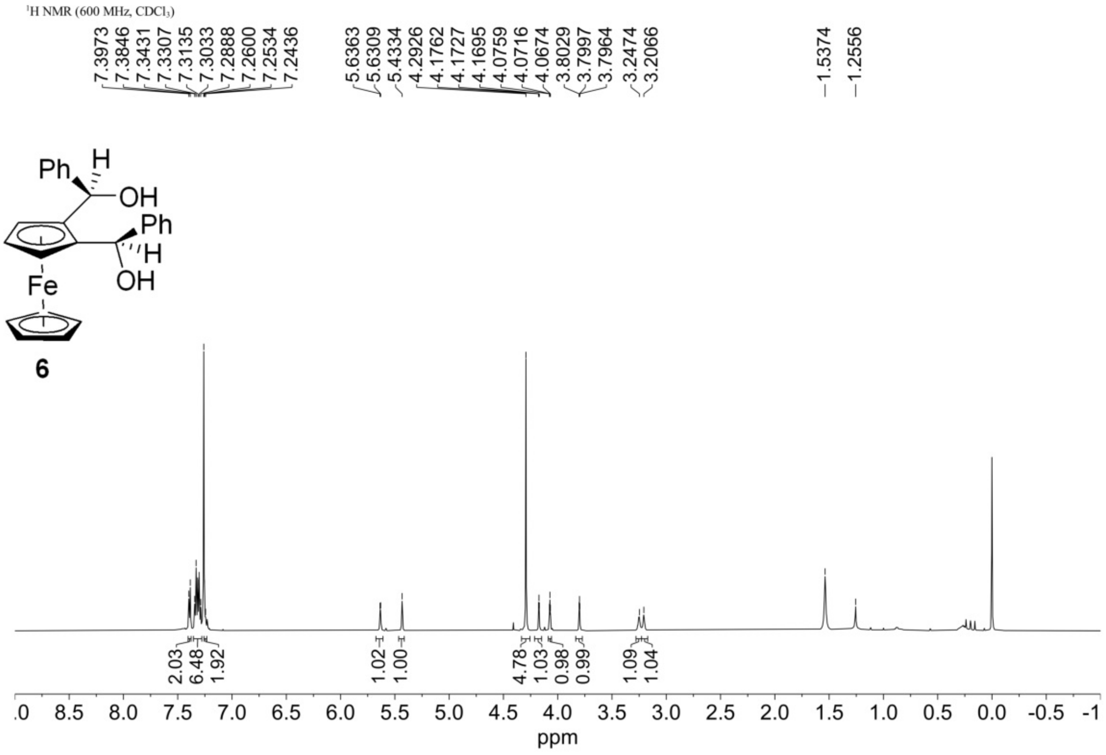

^{1}H NMR (600 MHz, CDCl$_3$)
7.3973
7.3846
7.3431
7.3307
7.3135
7.3033
7.2888
7.2600
7.2534
7.2436
5.6363
5.6309
5.4334
4.2926
4.1762
4.1727
4.1695
4.0759
4.0716
4.0674
3.8029
3.7997
3.7964
3.2474
3.2066
1.5374
1.2556
Ph
H
OH
Ph
H
OH
Fe
6
2.03
6.48
1.92
1.02
1.00
4.78
1.03
0.98
0.99
1.09
1.04
ppm

^{1}H NMR (600 MHz, $CDCl_3$)

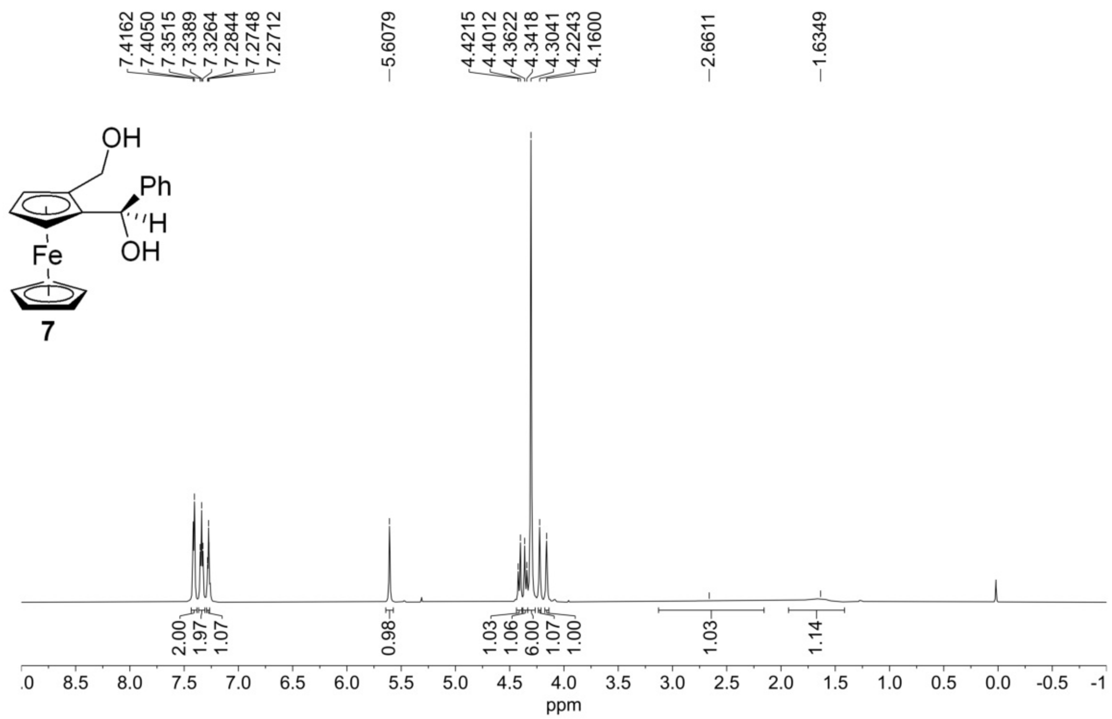

^{13}C NMR (150 MHz, $CDCl_3$)

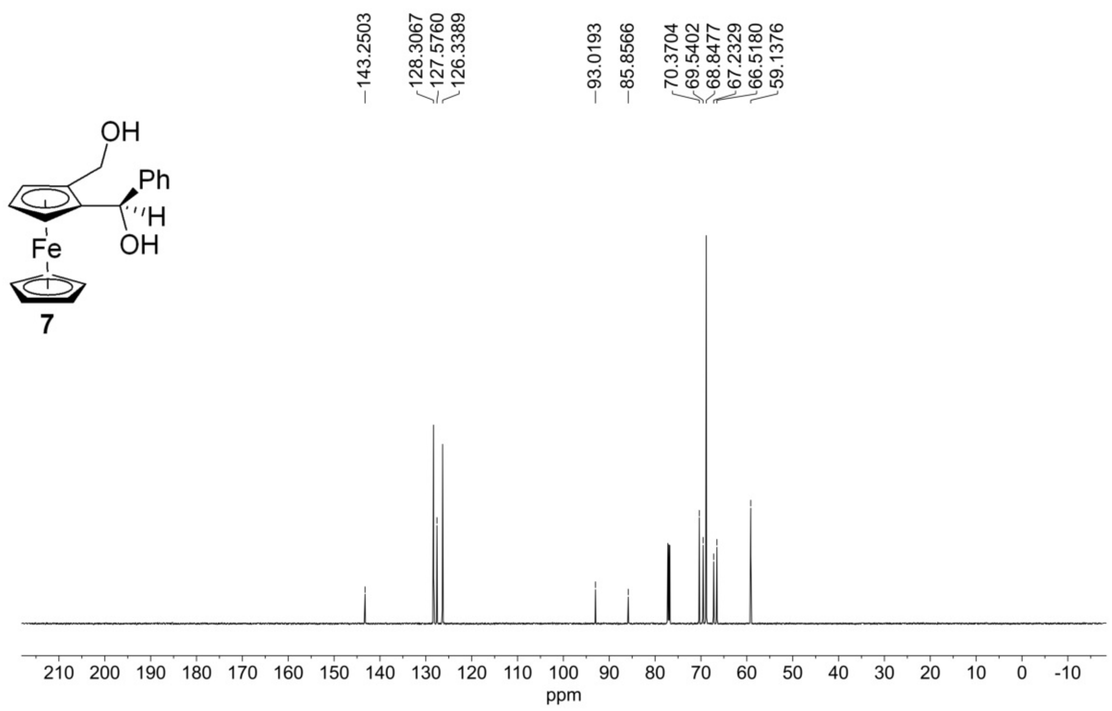

^{1}H NMR (600 MHz, $CDCl_3$)

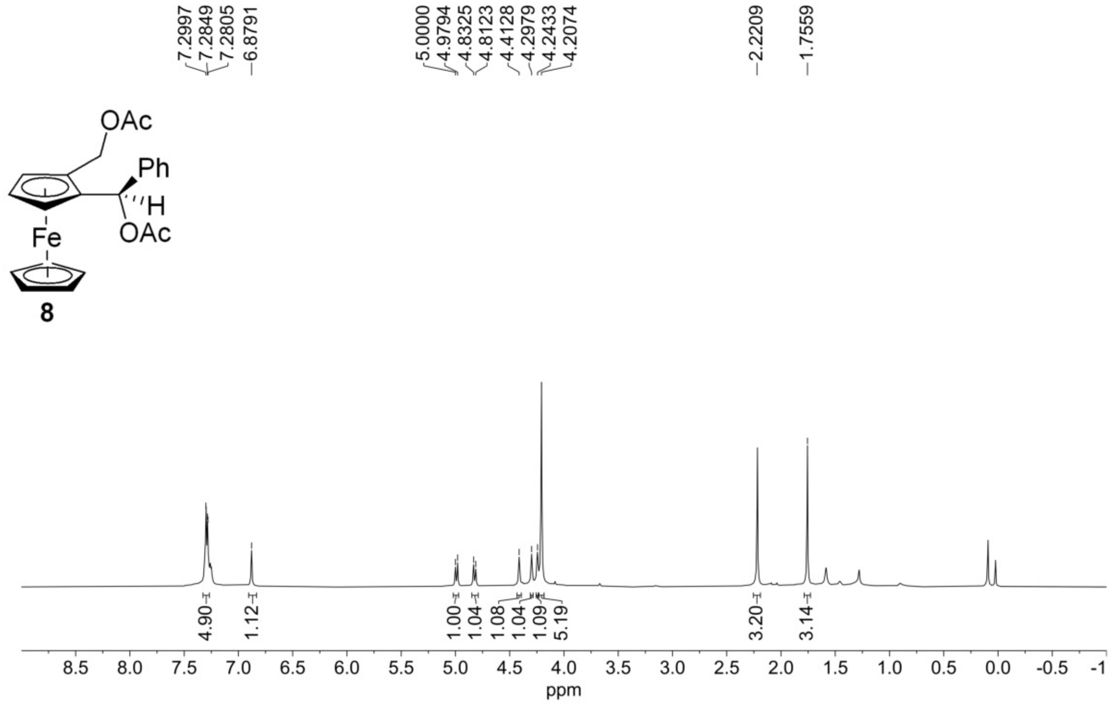

^{13}C NMR (150 MHz, $CDCl_3$)

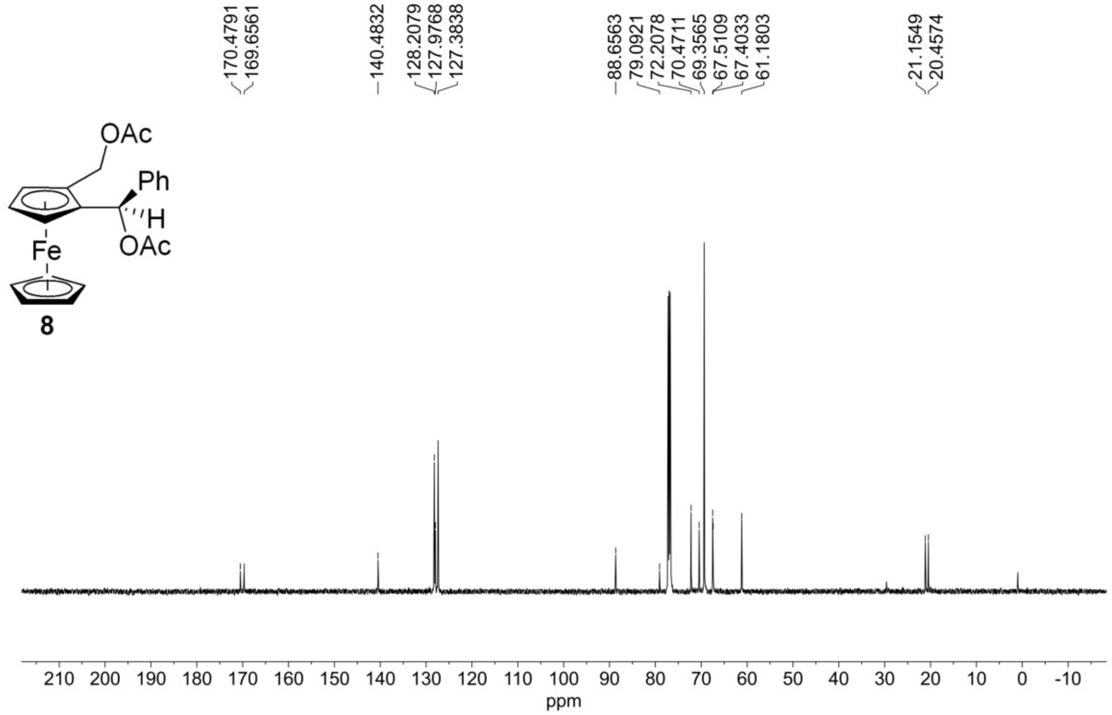

^{1}H NMR (600 MHz, $CDCl_3$)

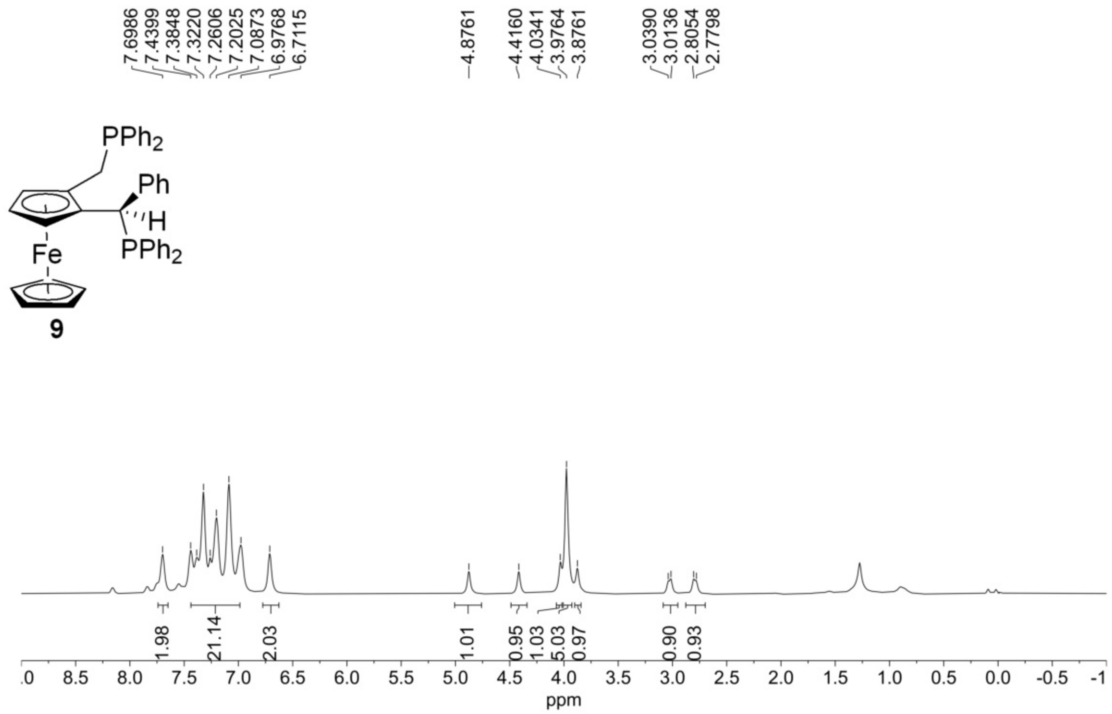

^{13}C NMR (150 MHz, $CDCl_3$)

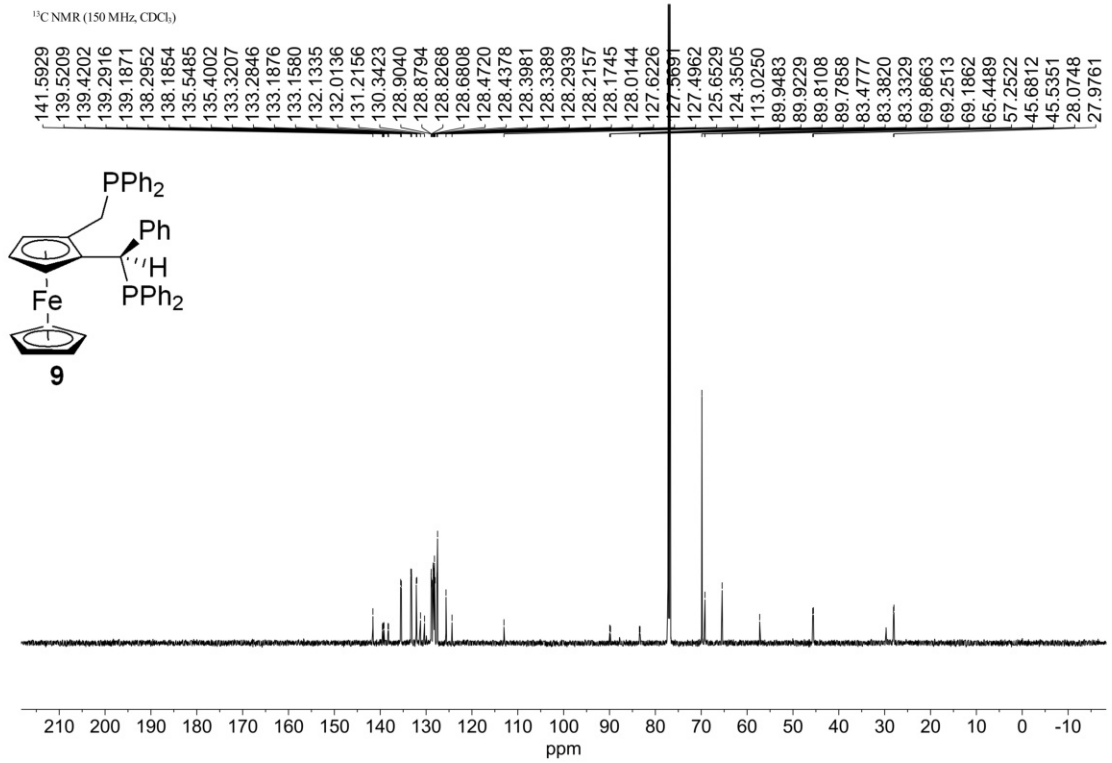

^{31}P NMR (243 MHz, $CDCl_3$)

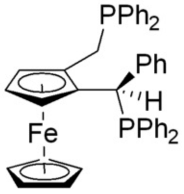

9

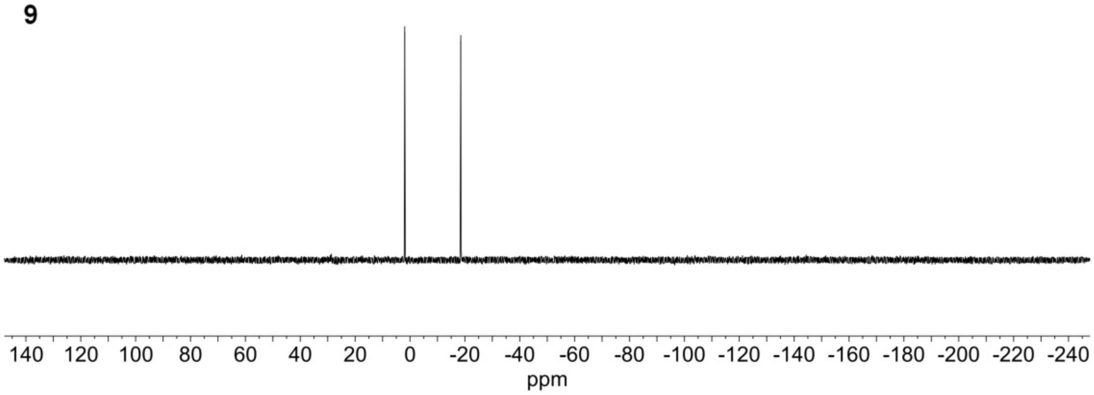

^{1}H NMR (600 MHz, $CDCl_3$)

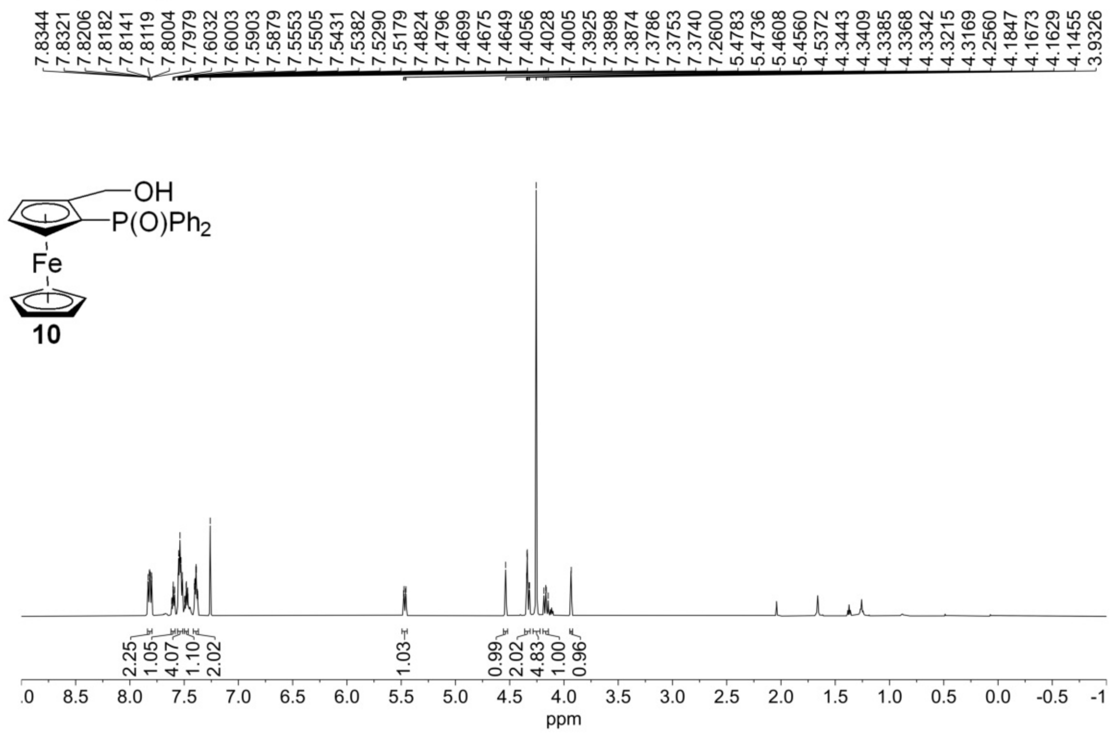

^{31}P NMR (243 MHz, $CDCl_3$)

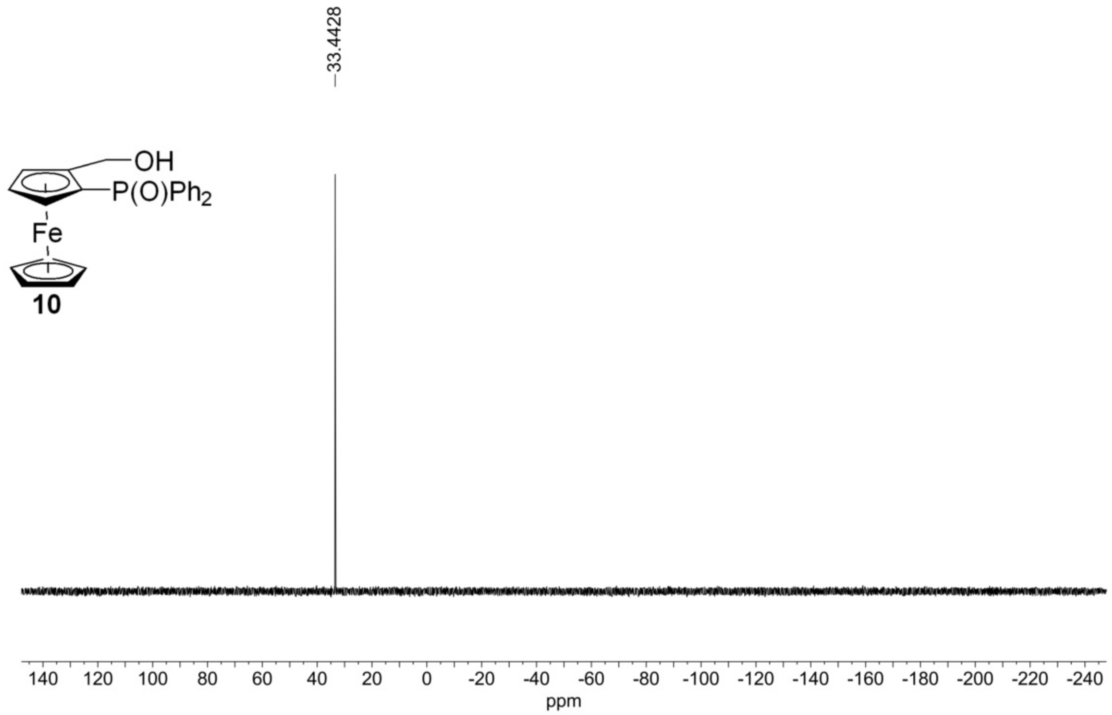

^{1}H NMR (600 MHz, $CDCl_3$)

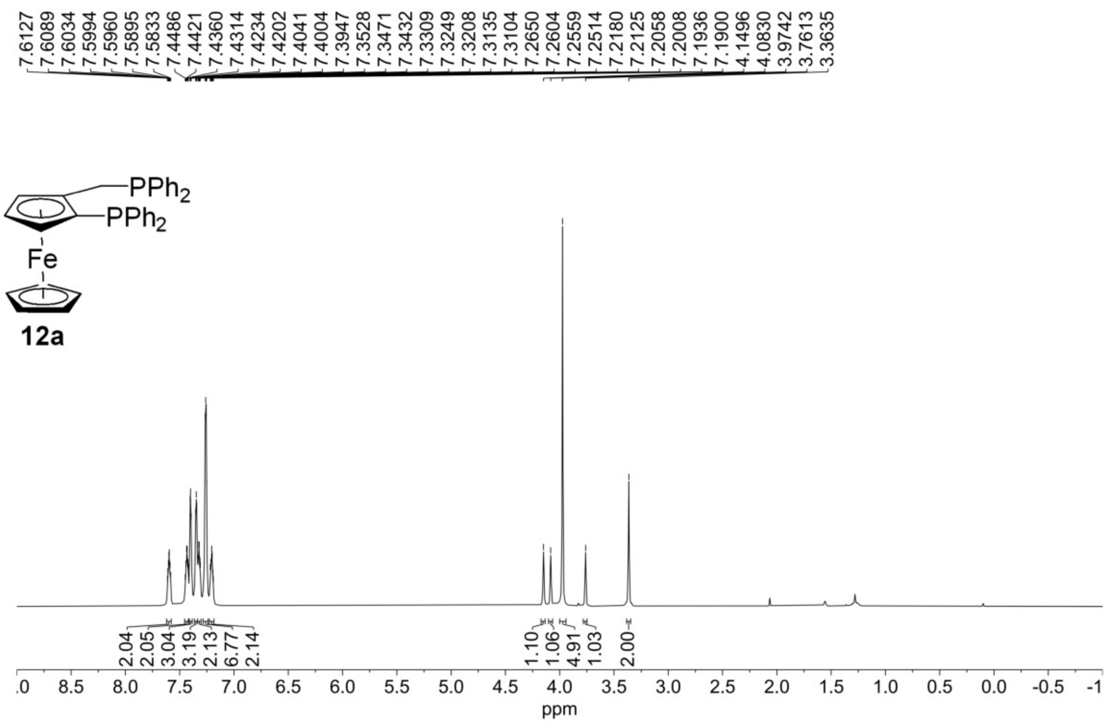

^{31}P NMR (243 MHz, $CDCl_3$)

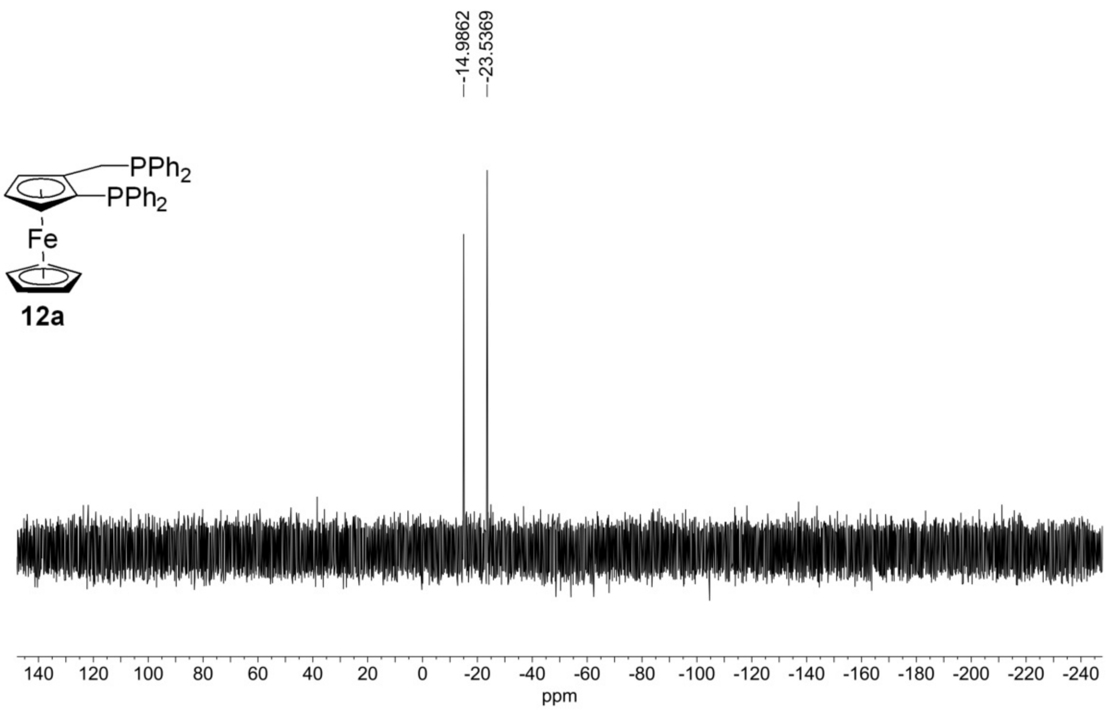

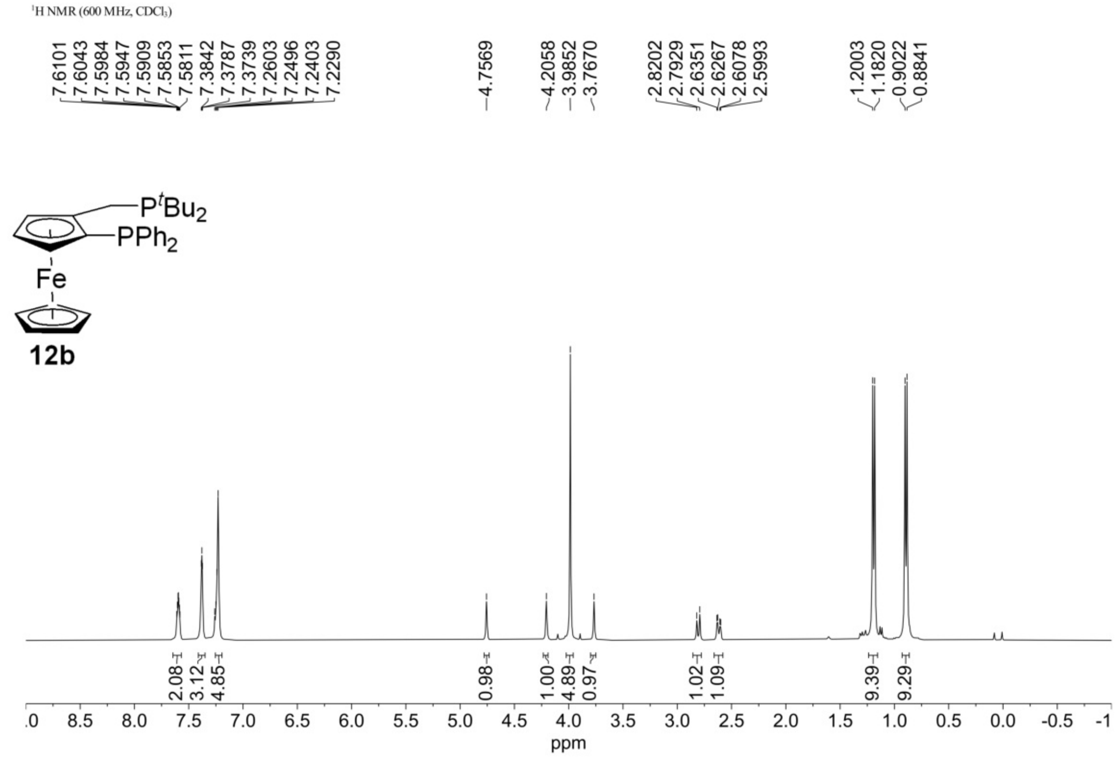
1H NMR (600 MHz, CDCl3)
7.6101
7.6043
7.5984
7.5947
7.5909
7.5853
7.5811
7.3842
7.3787
7.3739
7.2603
7.2496
7.2403
7.2290
4.7569
4.2058
3.9852
3.7670
2.8202
2.7929
2.6351
2.6267
2.6078
2.5993
1.2003
1.1820
0.9022
0.8841
PtBu2
PPh2
Fe
12b
2.08
3.12
4.85
0.98
1.00
4.89
0.97
1.02
1.09
9.39
9.29
ppm

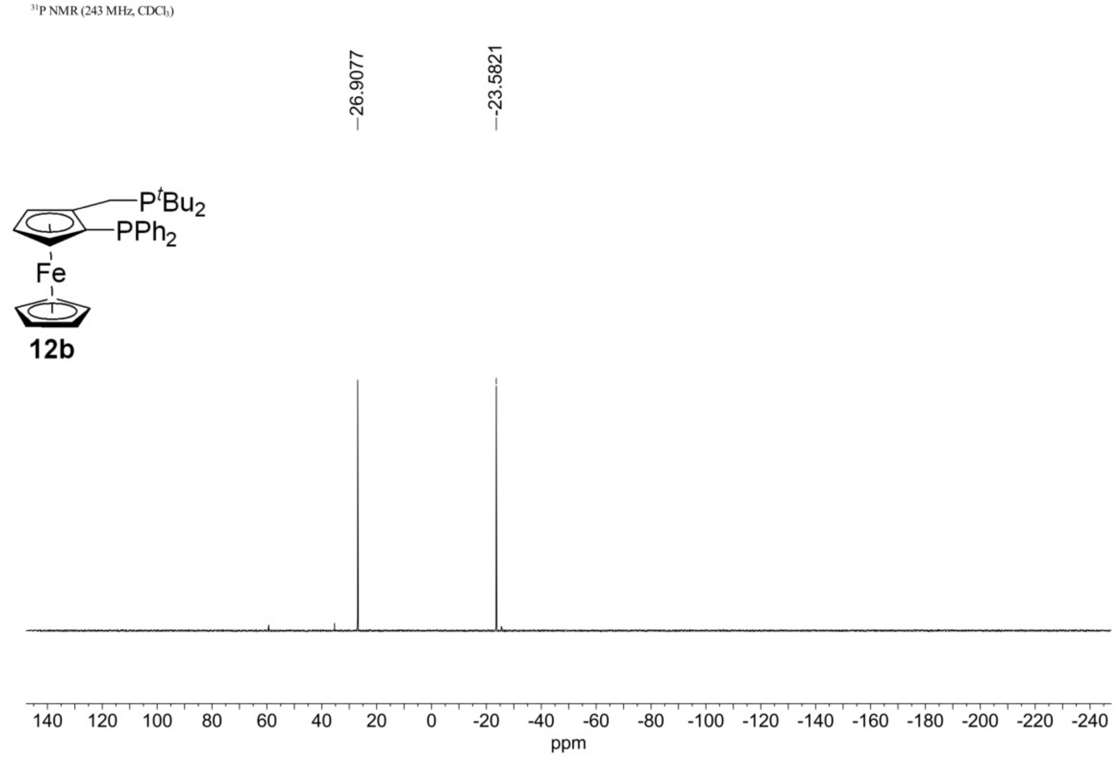
31P NMR (243 MHz, CDCl3)
26.9077
-23.5821
PtBu2
PPh2
Fe
12b
ppm

^{1}H NMR (600 MHz, $CDCl_3$)

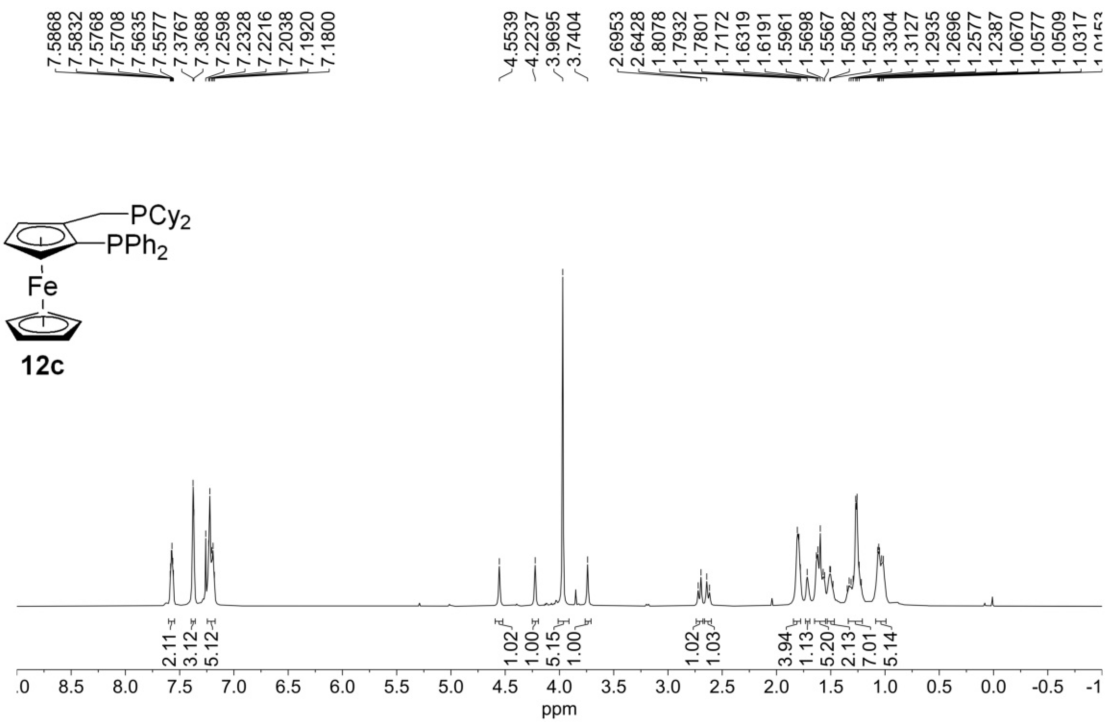

^{31}P NMR (243 MHz, $CDCl_3$)

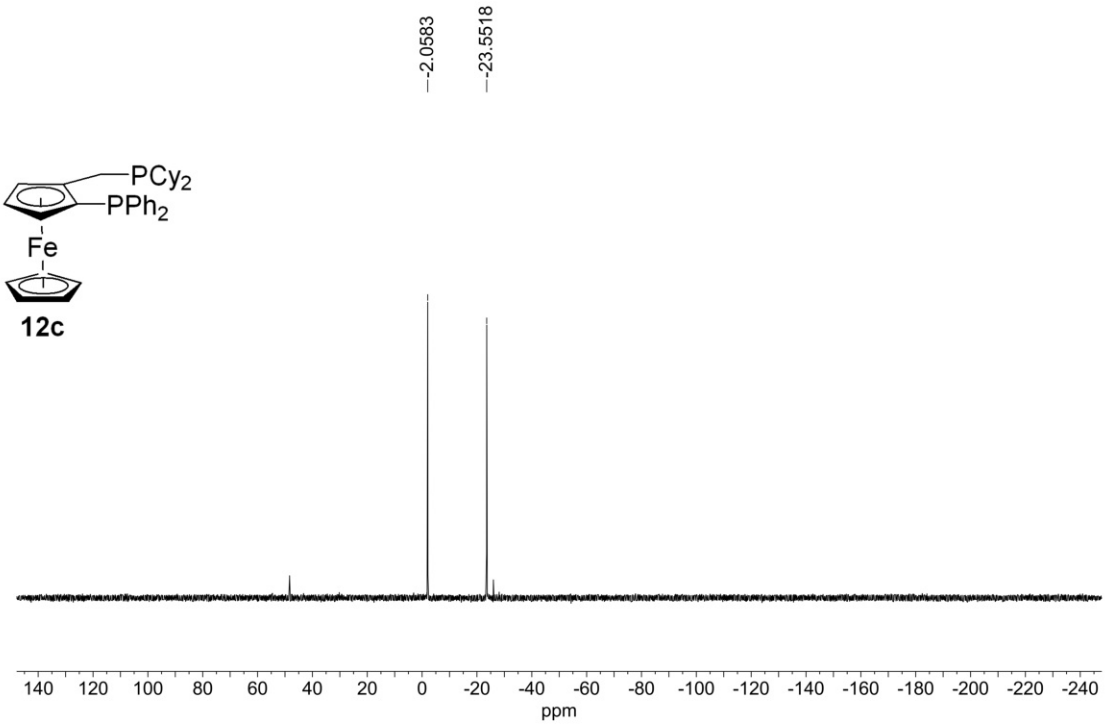

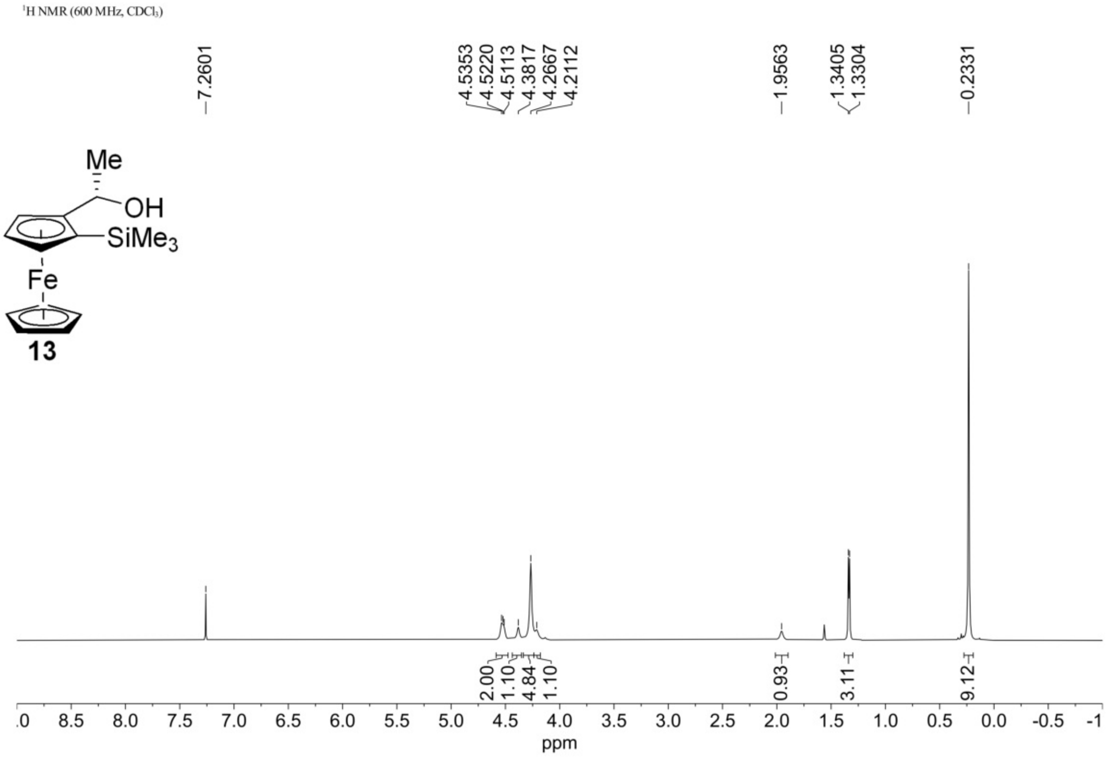
1H NMR (600 MHz, CDCl3)
7.2601
4.5353
4.5220
4.5113
4.3817
4.2667
4.2112
1.9563
1.3405
1.3304
0.2331
Me
OH
SiMe3
Fe
13
2.00
1.10
4.84
1.10
0.93
3.11
9.12
ppm

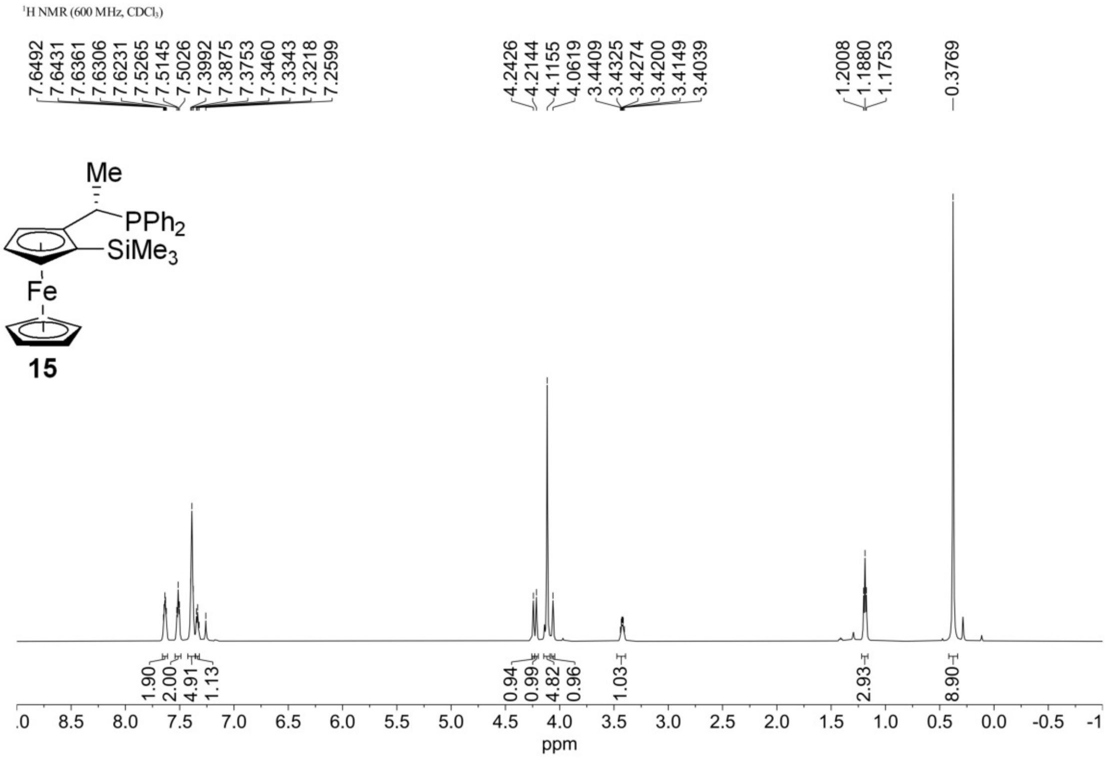
1H NMR (600 MHz, CDCl3)
7.6492
7.6431
7.6361
7.6306
7.6231
7.5265
7.5145
7.5026
7.3992
7.3875
7.3753
7.3460
7.3343
7.3218
7.2599
4.2426
4.2144
4.1155
4.0619
3.4409
3.4325
3.4274
3.4200
3.4149
3.4039
1.2008
1.1880
1.1753
0.3769
Me
PPh2
SiMe3
Fe
15
1.90
2.00
4.91
1.13
0.94
0.99
4.82
0.96
1.03
2.93
8.90
ppm

^{31}P NMR (243 MHz, $CDCl_3$)

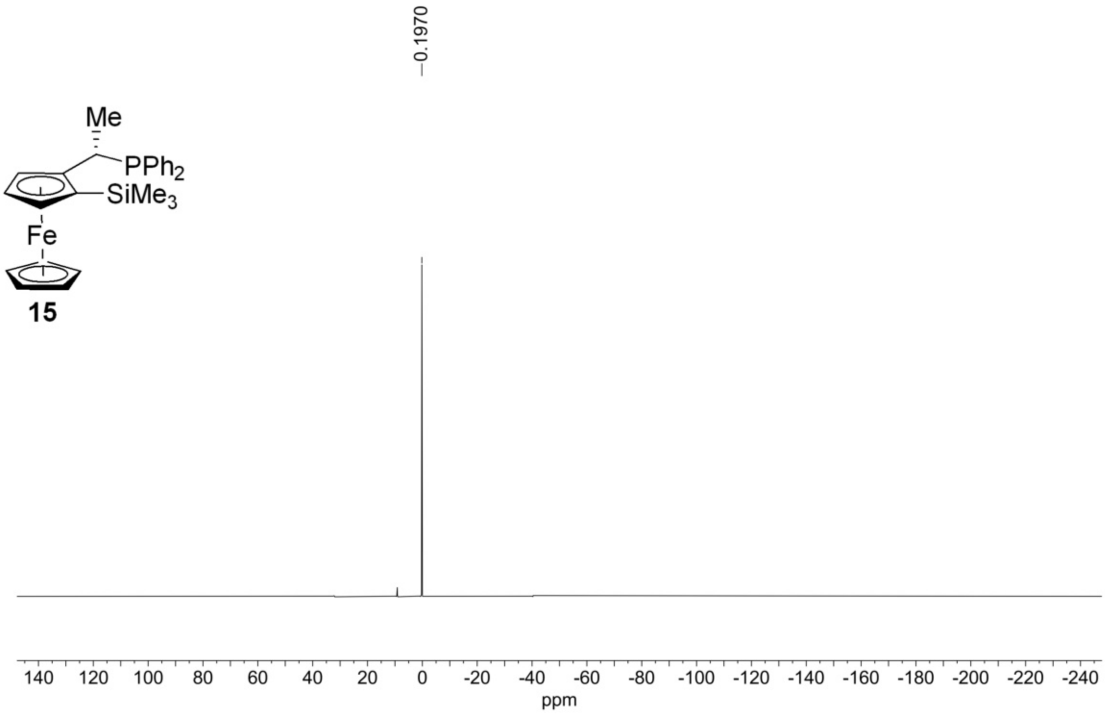

^{1}H NMR (600 MHz, $CDCl_3$)

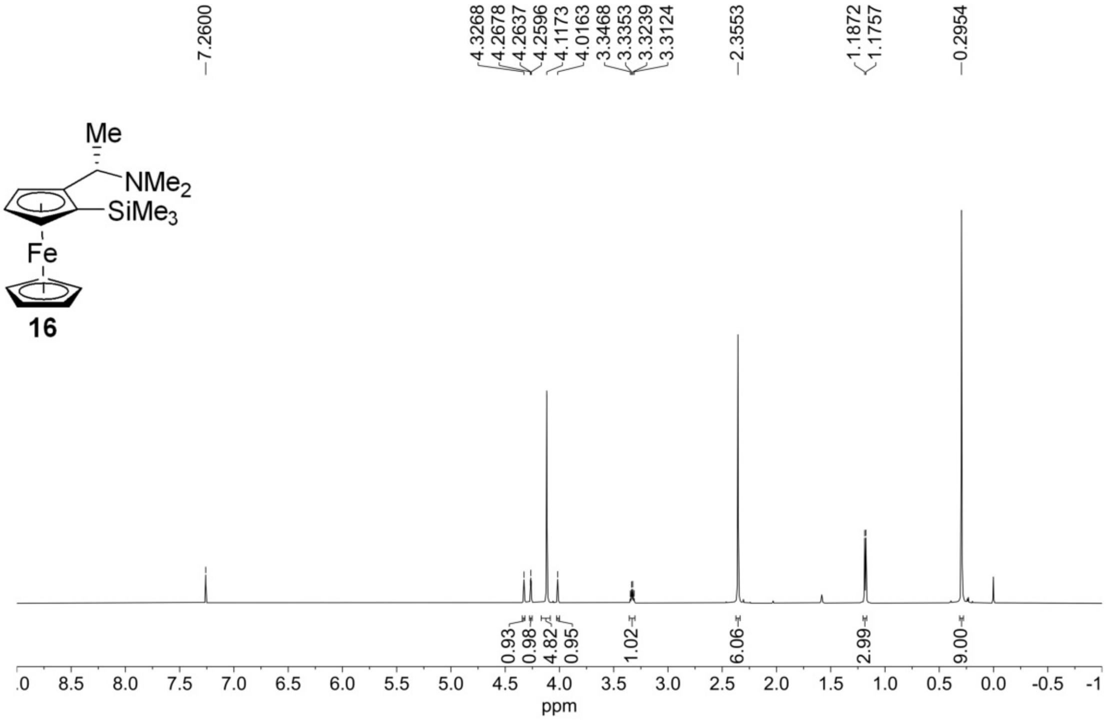

^{1}H NMR (600 MHz, $CDCl_3$)

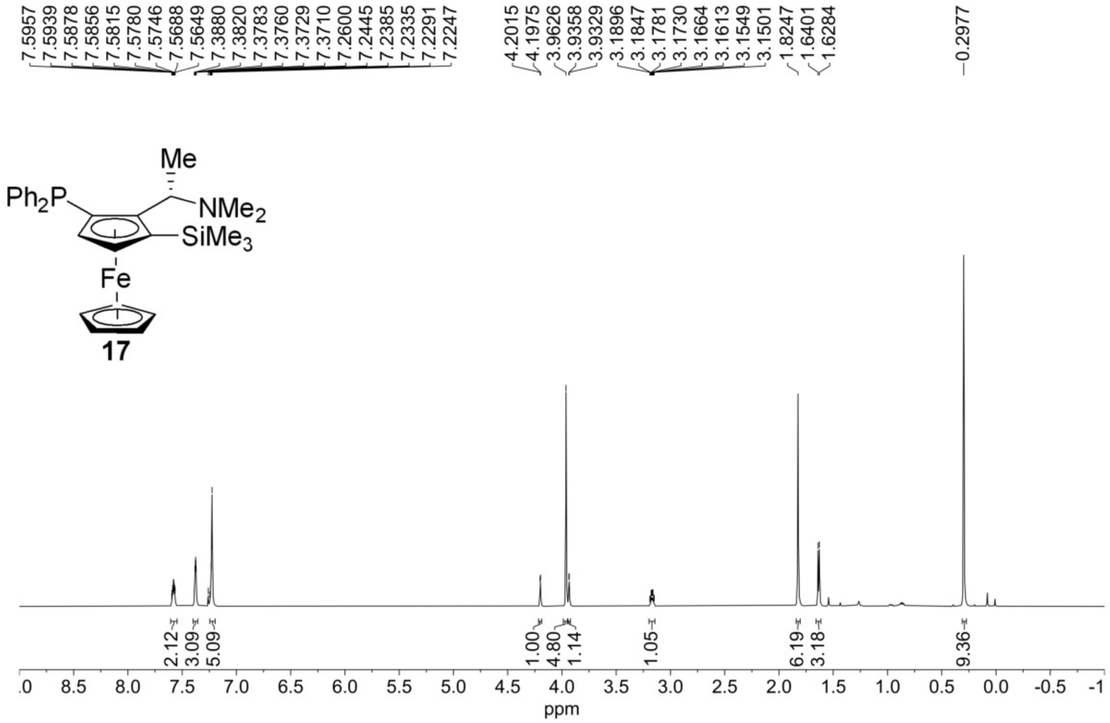

^{13}C NMR (150 MHz, $CDCl_3$)

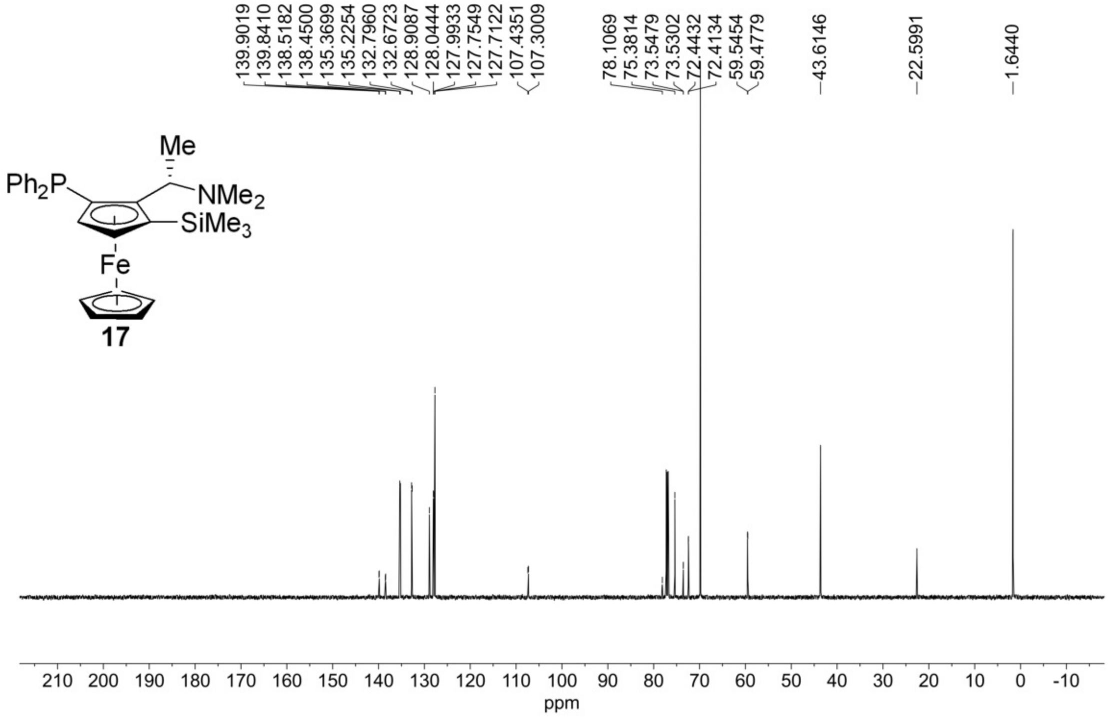

^{31}P NMR (243 MHz, $CDCl_3$)

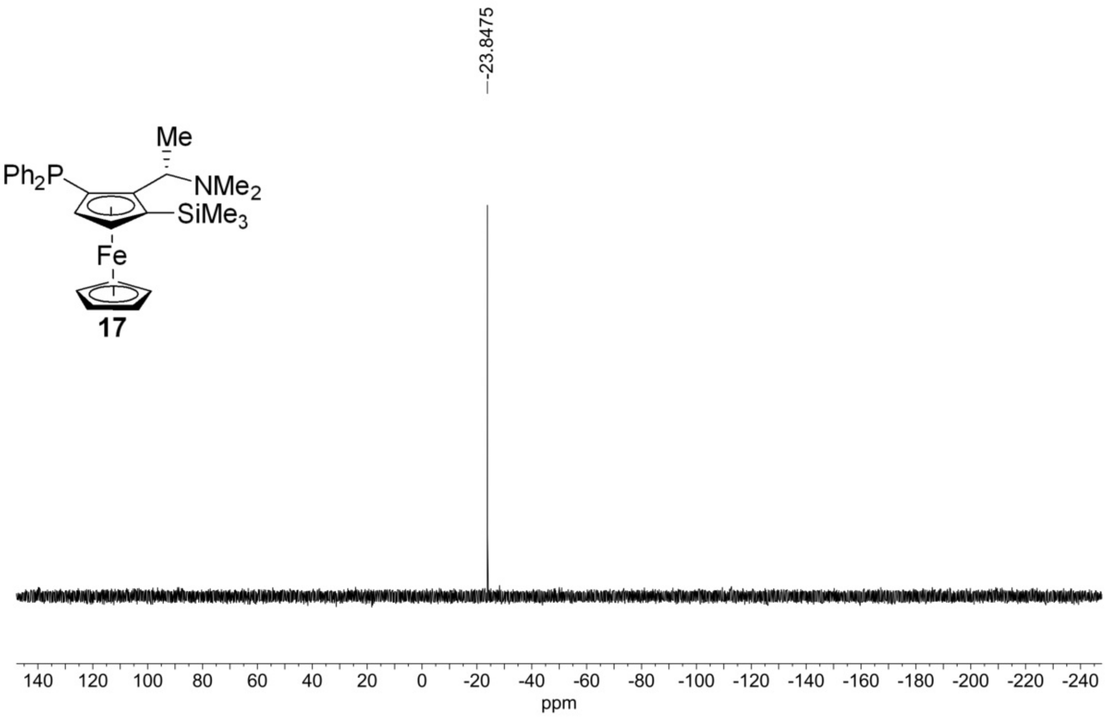

^{1}H NMR (600 MHz, $CDCl_3$)

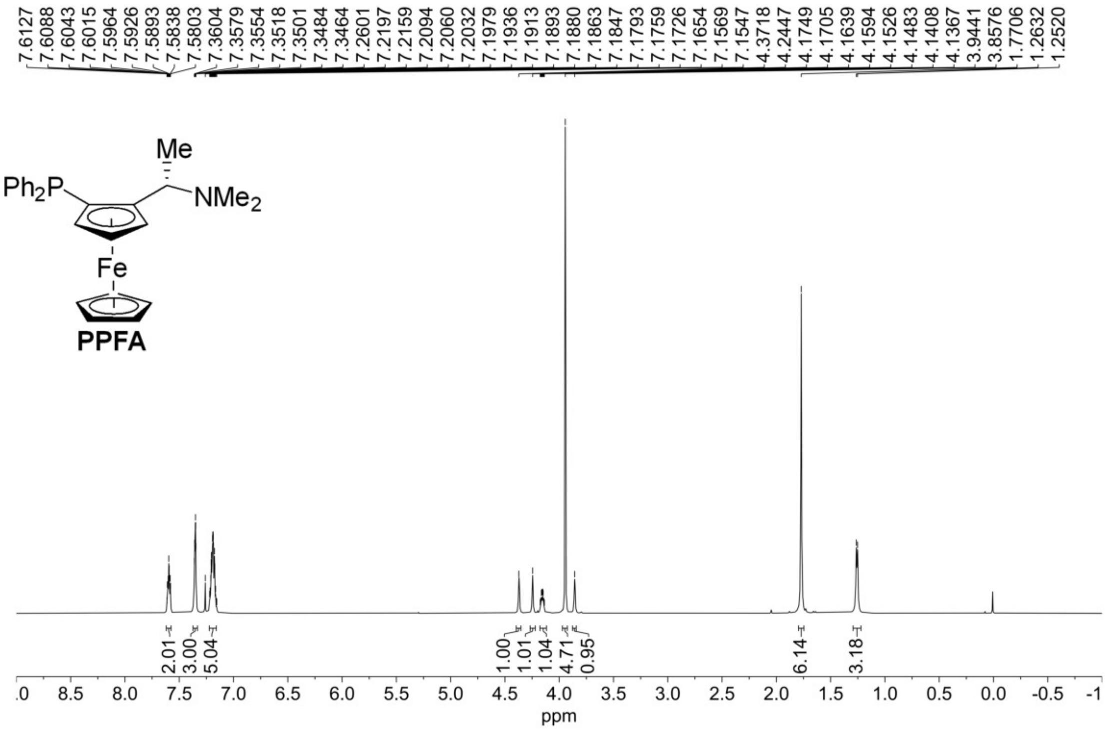

^{31}P NMR (243 MHz, $CDCl_3$)

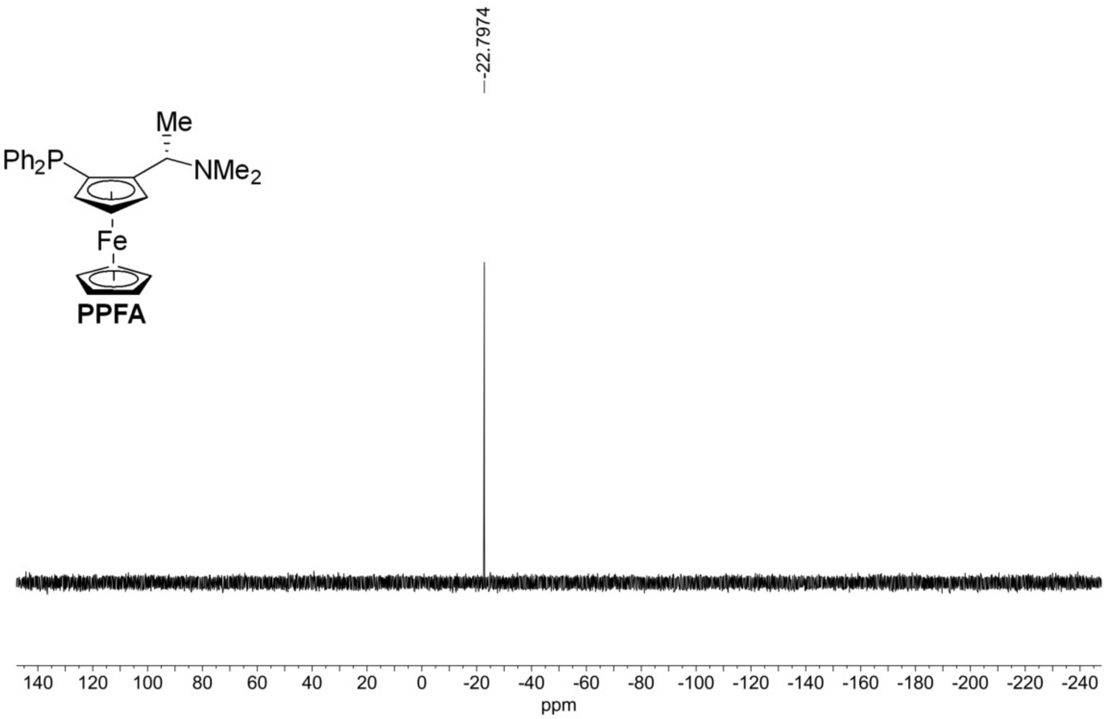

^1H NMR (600 MHz, $CDCl_3$)

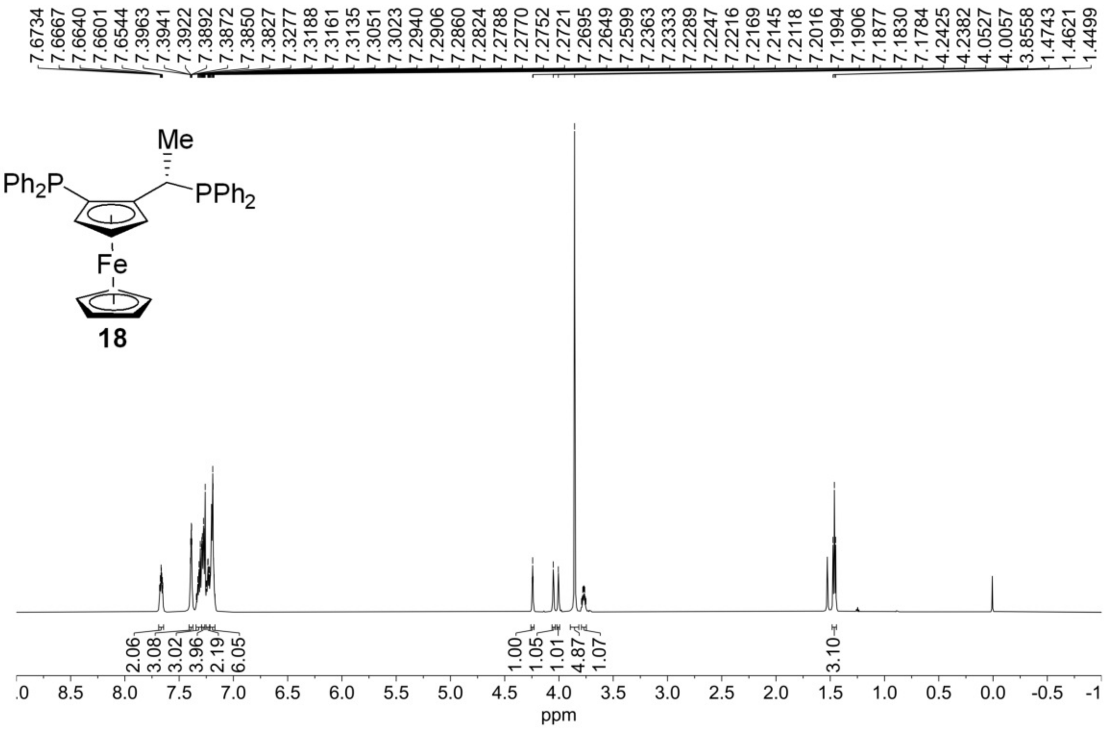

^{31}P NMR (243 MHz, $CDCl_3$)

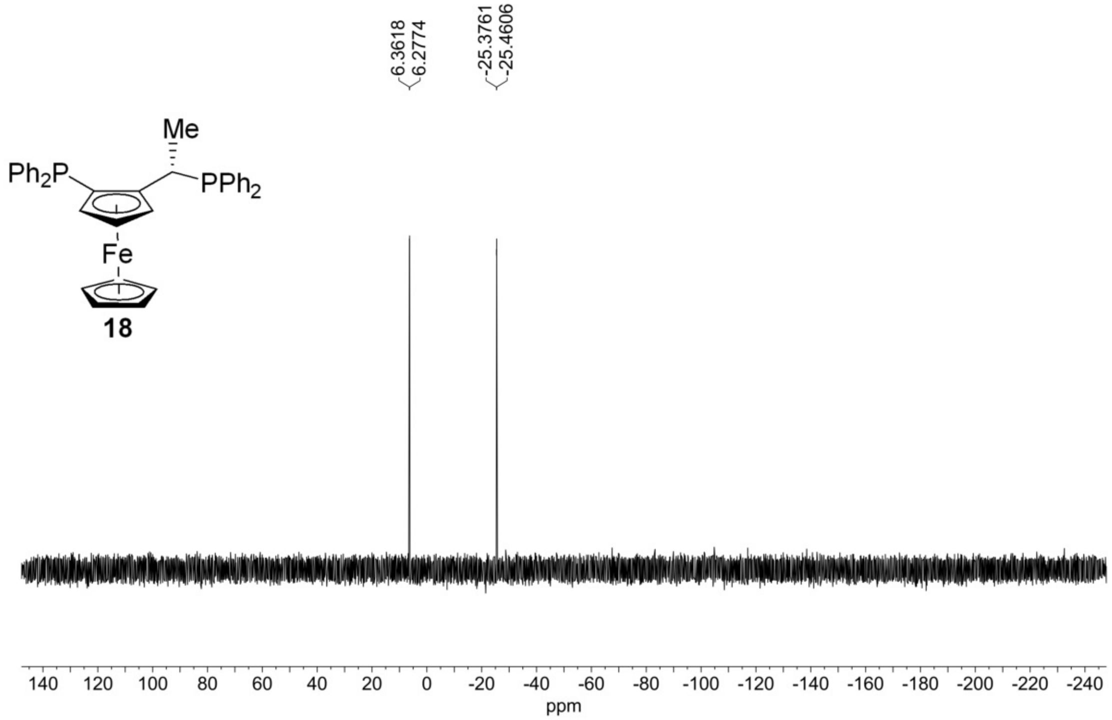

^{1}H NMR (600 MHz, Tol)

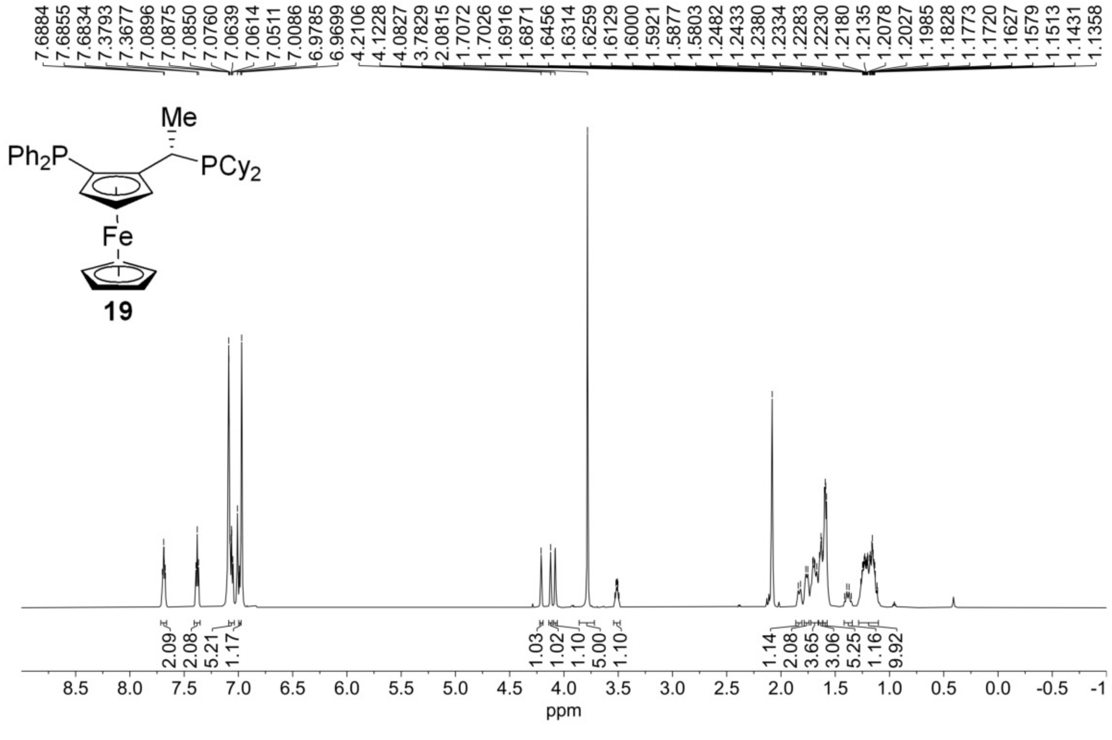

^{31}P NMR (243 MHz, Tol).

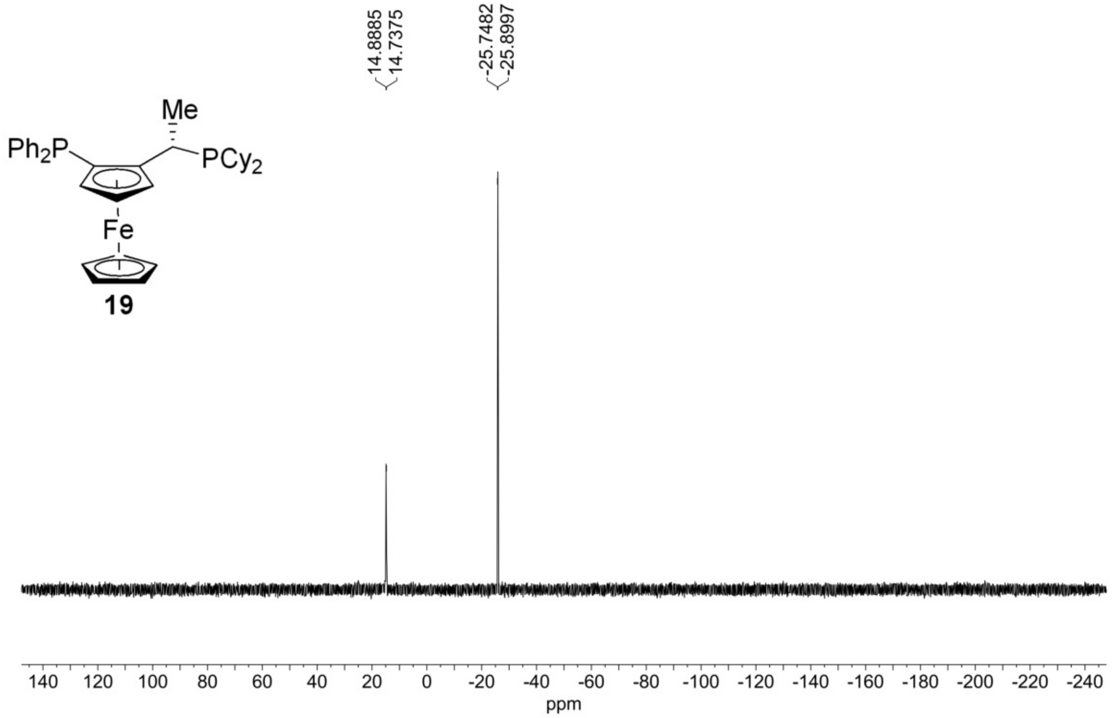

^{1}H NMR (600 MHz, $CDCl_3$)

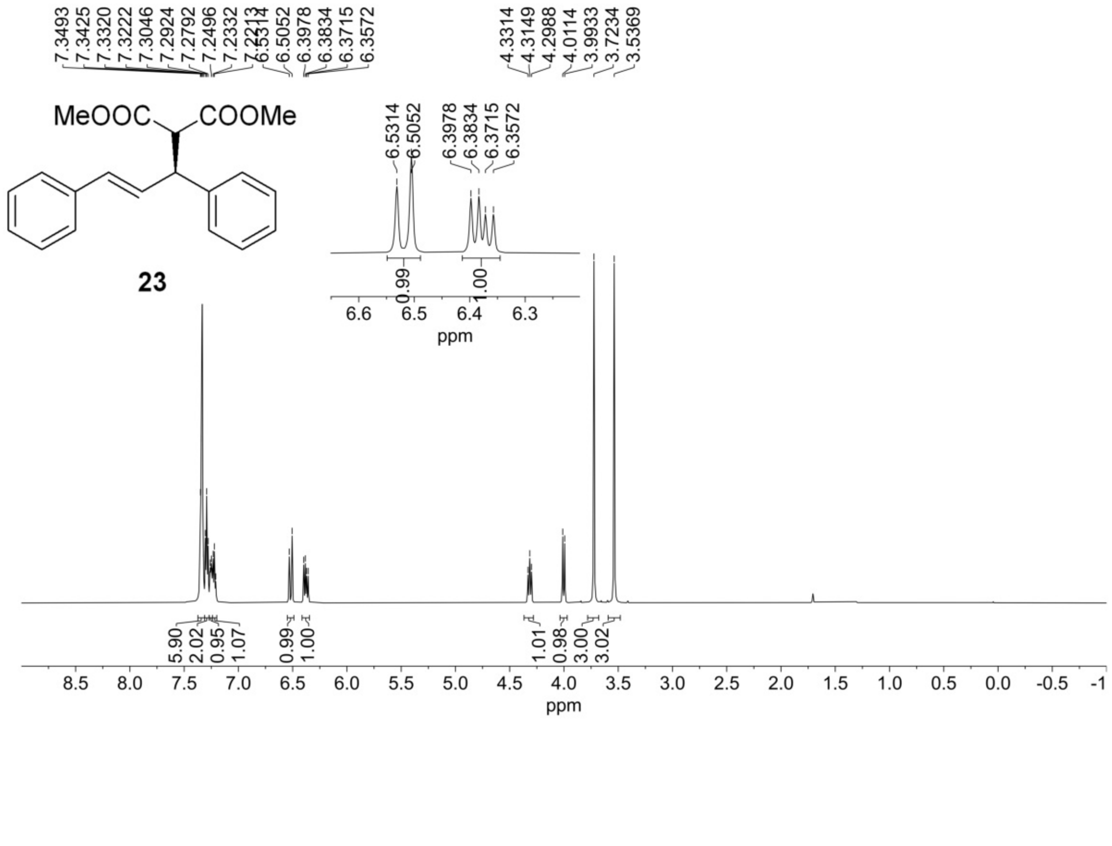

^{13}C NMR (150 MHz, $CDCl_3$)

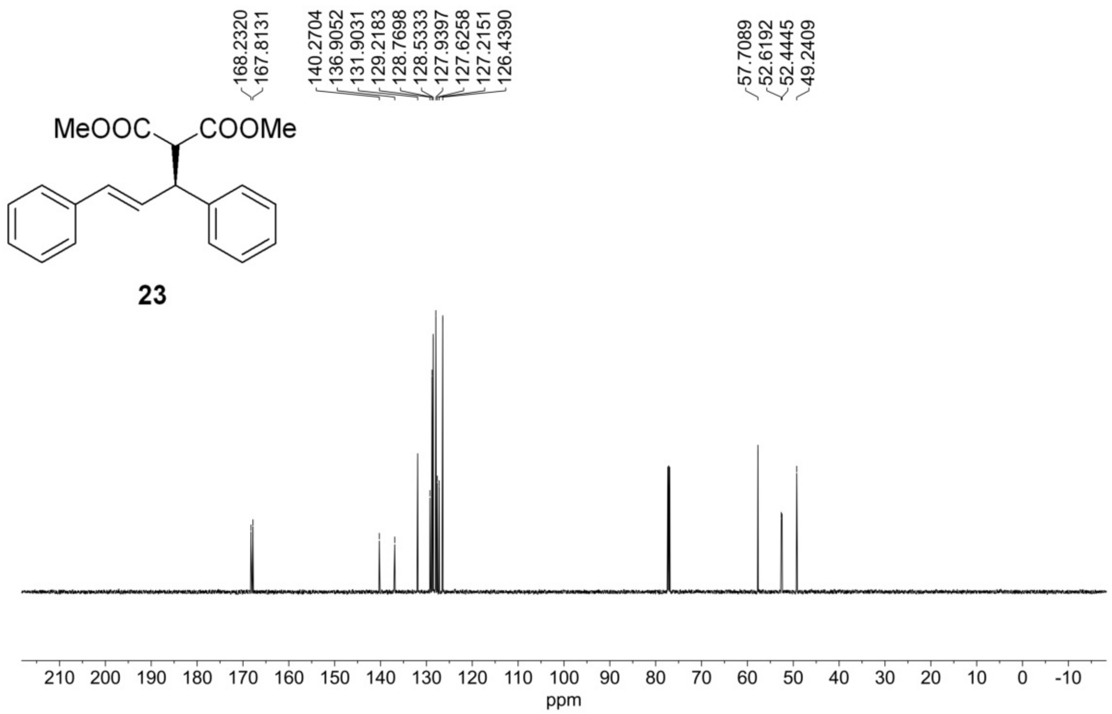

23
